# Screening of T Cell-Related Long Noncoding RNA-MicroRNA-mRNA Regulatory Networks in Non-Small-Cell Lung Cancer

**DOI:** 10.1155/2020/5816763

**Published:** 2020-11-14

**Authors:** Jinlong Duan, Yuefen Pan, Xi Yang, Liping Zhong, Yin Jin, Jiamin Xu, Jing Zhuang, Shuwen Han

**Affiliations:** ^1^Department of Oncology, Huzhou Hospital of Traditional Chinese Medicine, No. 315 South Street, Huzhou, Zhejiang Province, China 313000; ^2^Department of Oncology, Huzhou Central Hospital, Affiliated Central Hospital Huzhou University, No. 1558, Sanhuan North Road, Wuxing District, Huzhou, Zhejiang Province, China 313000; ^3^Department of Laboratory Medicine, Huzhou Central Hospital, Affiliated Central Hospital Huzhou University, No. 1558, Sanhuan North Road, Wuxing District, Huzhou, Zhejiang Province, China 313000; ^4^Graduate School of Nursing, Huzhou University, Huzhou, Zhejiang, No. 1 Bachelor Road, Huzhou, Zhejiang Province, China 313000

## Abstract

**Background:**

Lung cancer (LC) has the highest mortality rate among all the other types of cancer in the world. T cells are known to be the key factor in inducing the immune response during LC.

**Objective:**

In this study, we aimed to screen and analyze RNAs associated with CD8(+) T cells and activated memory CD4(+) T cells in lung adenocarcinomas, a subtype of non-small-cell lung cancer (NSCLC-LUAD).

**Methods:**

Gene expression RNA-seq data and clinical data of NSCLC-LUAD were downloaded from the XENA database. The data were divided into low scores and high scores based on the Stromal and Immune scores. Then, all the genes were screened for identifying those specifically associated with CD8(+) T cells and activated memory CD4(+) T cells. The screened genes were used for the construction of the protein-protein interaction (PPI) network and for Gene Ontology (GO) and Kyoto Encyclopedia of Genes and Genomes (KEGG) pathway enrichment analysis along with prognosis analysis. Based on the results of the prognostic analysis, the prognostic-related genes were used to analyze long noncoding (lnc)RNA-micro(mi)RNA-mRNA networks and to predict small chemical molecules.

**Results:**

According to the Immune and Stromal scores, a total of 885 upregulated and 29 downregulated RNAs were identified. A total of 90 differentially expressed genes (DEGs) were found to be related to CD8(+) T immune cells, and 48 DEGs were related to activated memory CD4(+) T cells. GPR174 and CD226 suggested a favorable prognosis. For CD8(+) and activated memory CD4(+) T cells, 112 and 113 PPI edges were obtained, respectively. GPR174 was found to be regulated by hsa-miR-19b-5p and hsa-miR-19b-2-5p, and both of these two miRNAs were regulated by lncRNA PCED1B-AS1. CD226 was regulated by hsa-miR-379-5p, which was in turn regulated by lncRNA RP11-81H14.2.

**Conclusion:**

Our findings provide a deeper understanding of the T cell-related ceRNA regulatory mechanism in NSCLC-LUAD pathogenesis.

## 1. Introduction

Lung cancer (LC) has the highest incidence (11.6% of total cancer cases) and highest mortality rate (18.4% of total cancer deaths) among cancers worldwide [1]. The 5-year overall survival (OS) rate of LC is about 15% [2]. Non-small-cell lung cancer (NSCLC) accounts for more than 80% of all LC cases, and lung adenocarcinomas (LUAD), which is a subtype of NSCLC, accounts for 50% of all LC cases. Inconspicuous clinical symptoms, difficult early diagnosis, different histological subtypes, and inadequate understanding of the biological characteristics of the tumor are the reasons for poor prognosis and high mortality in case of LC [3]. In recent years, immunotherapy has become a promising treatment for LC. T cells are known to be the key factor in the induction of immune response during LC [4]. Therefore, it is imperative to screen T cell-related molecular biomarkers and to understand the mechanism of the regulatory pathways for the early diagnosis and treatment of LC.

In LC, immune infiltration includes both, adaptive and innate immune cell populations [5, 6], and these immune cells are found to be present in a highly ordered state in the tumor tissue [7]. LC cells form an integral part of the tumor microenvironment. They express chemokine receptors and produce chemokines that regulate both, immune and tumor cells. Previous studies have shown that the chemokine receptor, CXCR4, is upregulated in the tumor cells when compared to the normal cells as it is actively involved in promoting the survival, proliferation, and metastasis of tumor cells [8]. Meanwhile, CXCR4-CXCL12 regulatory complex plays an important role in the metastasis of NSCLC. Other studies have shown that the regulatory complex, CCL20-CCR6, can promote the progression of NSCLC because of its proinflammatory and proliferative effects [9]. Several genes have been identified to mutate in LC, such as *FHIT*, *RB*, *TP53*, and *CDKN2*. Studies have shown that tumorigenic KRAS can potentially inhibit the T cell immunity-related genes in the pancreatic cancer models, thereby inhibiting the immune response against the tumor [10]. HOXD-AS1, a long noncoding RNA (lncRNA), which is known to be involved in the development of a variety of cancers, has been demonstrated to be upregulated in NSCLC tissues and to promote the growth of cancer cells by targeting miR-147a [11]. It was also shown that miR-199-5p can regulate NSCLC proliferation by downregulating the expression of hypoxia-inducible factor-1*α* (HIF-1*α*). It was also found that the plasmacytoma variant translocation 1 (PVT1) gene was overexpressed in hypoxic LC cells, which regulated the expression of HIF-1*α* through competitive inhibition of the binding of miR-199-5p [12].

However, there were few other reports on T cell-related RNAs in NSCLC-LUAD. Therefore, in this study, we aimed to complement this research domain by analyzing the characteristics of the RNA expression profile of T cells associated with NSCLC-LUAD. In the current study, the differentially expressed RNAs of CD8(+) T cells and activated memory CD4(+) T cells related to NSCLC-LUAD were analyzed by the Estimation of STromal and Immune cells in MAlignant Tumours using Expression data (ESTIMATE, version 1.0.13) algorithm, in order to screen the potential biomarkers related to the prognosis of NSCLC-LUAD. Thus, it may further provide clinical guidance for the prognosis and treatment of LC from the perspective of immunity.

## 2. Materials and Methods

### 2.1. Data Collection and Preprocessing

Gene expression RNA-seq data (including Counts and FPKM) and clinical data (including phenotype) for the lung adenocarcinoma, a subtype of NSCLC (NSCLC-LUAD, version07-20-2019), were downloaded from the TCGA dataset, which is included in the XENA database (https://xenabrowser.net/) [13]. The platform used for the sequencing was Illumina HiSeq 2000 RNA Sequencing platform.

RNA-seq data was annotated using the annotation files (gencode.v22.annotation.gene) present in the Gencode database (https://www.gencodegenes.org/) [14]. Genes whose annotation information was indicated as “protein_coding” were extracted as mRNAs. Meanwhile, genes whose annotation information was indicated as “processed_transcript,” “lincRNA,” “3prime_overlapping_ncrna,” “antisense,” “non_coding,” “sense_intronic,” “sense_overlapping,” “TEC,” “known_ncrna,” “macro_lncRNA,” “bidirectional_promoter_lncrna,” or “lncRNA” were extracted as long noncoding RNAs (lncRNAs). Other genes were considered undefined.

The log2 (count +1) value of the sequencing data of Counts was restored as the count value, and the log2 (FPKM +1) value of the sequencing data of FPKM was restored as the FPKM value. Expression data of tumor samples suffixed with “01A” were extracted. The “Ensembl ID” was converted to “Symbol ID,” and when multiple “Ensembl IDs” corresponded to the same “Symbol ID,” the mean of the expressions of these “Ensembl IDs” was taken as the expression of the resultant “Symbol ID.” Samples for which both expression data and survival information were available were extracted, and overall survival time (OS time) and overall survival status (OS status) were estimated. OS time was modified from days to months.

### 2.2. Tumor Microenvironment Analysis Based on ESTIMATE Algorithm

The Estimation of STromal and Immune cells in MAlignant Tumours using Expression data (ESTIMATE, version 1.0.13) algorithm present in the R package was used to calculate the Stromal and Immune scores of all the samples [15]. ESTIMATE is a tool for predicting the presence of infiltrating stromal/immune cells in tumor tissues by using the gene expression data.

### 2.3. Differential Gene Expression Analysis

Based on the median of Stromal and Immune scores and the classical Bayesian modified *t*-test method provided by the limma package (version 3.40.6) [16], the R package was used to divide the samples into the high-scoring and low-scoring groups. Differential RNAs (including both, lncRNAs and mRNAs) were screened from different samples and by estimating the significance levels, for which the threshold was set as follows: *P* value < 0.05 and ∣logFC | >1. The differential RNA volcano map was obtained by using the ggscatter of the ggpubr tools (version 0.2.2) of the R package [17]. The R heat map package [18] was used to draw the monolayer and bilayer clustering heat maps for all the differentially expressed RNAs, and simultaneously, the monolayer and bilayer clustering heat maps for the differentially expressed RNAs were drawn based on the classification of mRNA and lncRNA.

### 2.4. Screening of Differentially Expressed Genes (DEGs) and Enrichment Analysis

As two major nontumor components in the tumor microenvironment, immune and stromal cells have certain potential in the diagnosis and prognosis of tumor. Based on the results of the analysis in the last step, the intersections of the Stromal and Immune scores between the two groups of DEGs (with the same upregulated relationship) were considered the DEGs related to the tumor microenvironment.

ClusterProfiler (version 3.12.0) [19] was used to analyze the Gene Ontology (GO) [20] and Kyoto Encyclopedia of Genes and Genomes (KEGG) [21] pathway enrichment for both the upregulated and downregulated genes. The GO analysis included Biological Process (BP), Cellular Component (CC), and Molecular Function (MF). *P* value < 0.05 and count ≥ 2 were considered to indicate significant enrichment.

### 2.5. Immunocyte Infiltration Abundance Analysis and T Cell-Related Gene Screening

The CIBERSORT deconvolution algorithm [22] of R package and ClusterProfiler were applied to estimate the infiltration abundance of 22 immune cell types in all the samples, based on the expression matrix. The LM22 dataset provided on the CIBERSORT website was set as the gene expression characteristic template, and the parameters were set as follows: perm = 100 and QN = *F*. The related landscape was drawn using the R package.

The tool corrplot (version 0.84) of R package [22] was used to evaluate the Pearson's correlation between the differential RNAs obtained in the last step, and then, the infiltration abundance of CD8(+) T cells and activated memory CD4(+) T cells was estimated, respectively. Differentially expressed RNAs with the Bonferroni-corrected *P* value < 0.05 and correlation coefficient ∣*r* | >0.3 were screened, which were considered to be related to the DEGs of the two T cell types infiltrated in the LC patients. Then, the GO and KEGG pathway enrichment analysis of the immune-correlated DEGs was performed. According to the NSCLC-related pathways included in the Comparative Toxicogenomics Database and CTD Database (http://ctdbase.org/) [23], the KEGG pathways enriched in the immune-related DEGs of the two T cell types were screened further.

### 2.6. Survival Analysis

The survival (version 2.44-1.1) tool of R package [24] was used to analyze the relationship between the immune-related differential RNAs of the two T cell types and the OS of the samples. According to the median gene expression, samples were divided into two groups with high and low expression, respectively, and were further subjected to the log-rank test. The threshold was set as *P* value < 0.05. Prognostic-related RNAs were screened, and Kaplan-Meier (K-M) survival curve was plotted. The mRNAs that were related with the survival were also screened.

### 2.7. Establishment of Protein-Protein Interaction (PPI) Network

The interaction between the encoded proteins was predicted and analyzed using the STRING (version 11.0, https://www.string-db.org/) database [25]. The PPI score was set at 0.4. After the PPI edges were obtained, Cytoscape software [26] was used to construct a network, and the survival-related differential genes were identified from the resultant network. The CytoNCA [27] plug-in of the Cytoscape software was applied to analyze the topological properties (betweenness, closeness, and degree) of the network.

### 2.8. Competing Endogenous RNA (ceRNA) Network Analysis

MicroRNAs (miRNAs) that can bind the 3′UTR regions of the immune-related DEGs of the two T cell types were predicted using the relevant databases (miRWalk3.0 [28], TargetScan [29], MiRDB [30], and MirTarBase [31]), with a threshold score > 0.95. Combining the results from different databases, the validated (MirTarBase database) miRNAs that were also predicted in either TargetScan or MiRDB were selected as the final mRNA-miRNA edge. The HMDD V3.2 database [32] was used to retrieve the keywords “Carcinoma, Lung and non-small-cell” in order to further verify and screen the predicted miRNAs.

The Prediction Module of DIANA-LncBase v.2 database (http://carolina.imis.athena-innovation.gr/diana_tools/web/index.php?r=lncbasev2%2Findex) [33] was applied to predict the miRNAs that might be related to the immune-related differences of the two T cell types, and then, the lncRNA-miRNA regulatory complexes were screened based on the results of the last step.

The integrated mRNA-miRNA edges and the lncRNA-mRNA edges were used to construct the ceRNA network using the Cytoscape software. The differential mRNAs and lncRNAs and their upregulation status were labeled in the resultant network.

### 2.9. Small Chemical Molecule Prediction Analysis

Firstly, in the Comparative Toxicogenomics Database, “Carcinoma, non-small-cell Lung” were used as the keywords to search for genes directly related to this disease. Then, the disease-related genes were obtained by comparing with the ceRNA network gene, and further, the relevant small chemical molecules were obtained. Finally, the network was constructed using the Cytoscape software in order to obtain the small chemical molecules that might be associated with the disease treatment.

## 3. Results

### 3.1. Data Preprocessing

According to the description provided in Materials and Methods, a total of 510 tumor samples were included in this study. We calculated the counts and FPKM values of these 510 tumor samples, and in the end, we obtained 58,387 × 510 counts and FPKM expression matrix. There were 10,221 genes that matched, and 191 genes that did not match in the ESTIMATE algorithm.

### 3.2. Identification of Differentially Expressed mRNAs and lncRNAs

**Figure 1:**
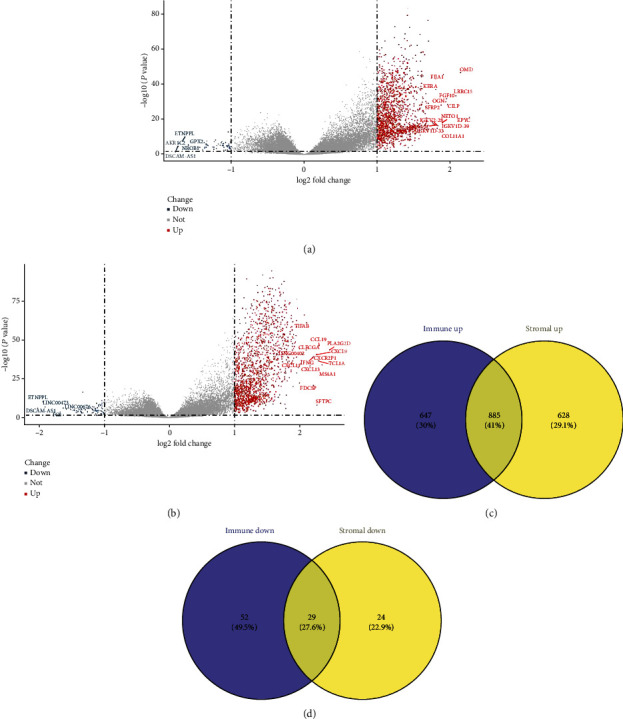
(a) Volcano plot of DEGs in the Stromal score group, (b) Immune score, and (c) Venn plots of DEGs that are upregulated and (d) down regulated. (a) To screen the DEGs between the high score group *vs.* the low score group, the gene expression RNA-seq data (counts, FPKM) and clinical data (phenotype) of NSCLC-LUAD were downloaded from XENA. In total, 510 tumor samples were obtained. In the Stromal score group, 1,031 mRNAs (998 upregulated, 33 downregulated) and 200 lncRNAs (184 upregulated, 16 downregulated) were obtained. (b) A total of 940 mRNAs (886 upregulated, 54 downregulated) and 272 lncRNAs (250 upregulated, 22 downregulated) were obtained according to the Immune Score result. Blue dots represent downregulated genes. Red dots represent upregulated genes. Grey dots represent nondifferentially expressed genes. DEGs represent differentially expressed genes. (c, d) In order to further analyze the DEGs, the intersection of the two groups of DEGs was screened. A total of 885 upregulated RNAs (88 lncRNAs, 797 mRNAs) and 29 downregulated RNAs (8 lncRNAs, 21 mRNAs) were obtained. Purple represents the Immune score group of DEGs. Yellow represents the Stromal score group of DEGs. DEGs represent differentially expressed genes.

Based on the results of the Stromal score, a total of 1,031 mRNAs (998 upregulated, 33 downregulated) and 200 lncRNAs (184 upregulated, 16 downregulated) were obtained (Figure 1(a)). However, based on the results of the Immune score, a total of 940 mRNAs (886 upregulated, 54 downregulated) and 272 lncRNAs (250 upregulated, 22 downregulated) were obtained (Figure 1(b)). The details are presented in the Supplementary Tables 1 and 2.

### 3.3. Screening of DEGs and Enrichment Analysis

The results of the DEG analysis of the two groups were taken as the intersection, and a total of 885 upregulated RNAs (88 lncRNAs, 797 mRNAs), and 29 downregulated RNAs (8 lncRNAs, 21 mRNAs) were obtained (Figures 1(c) and 1(d)).

**Figure 2:**
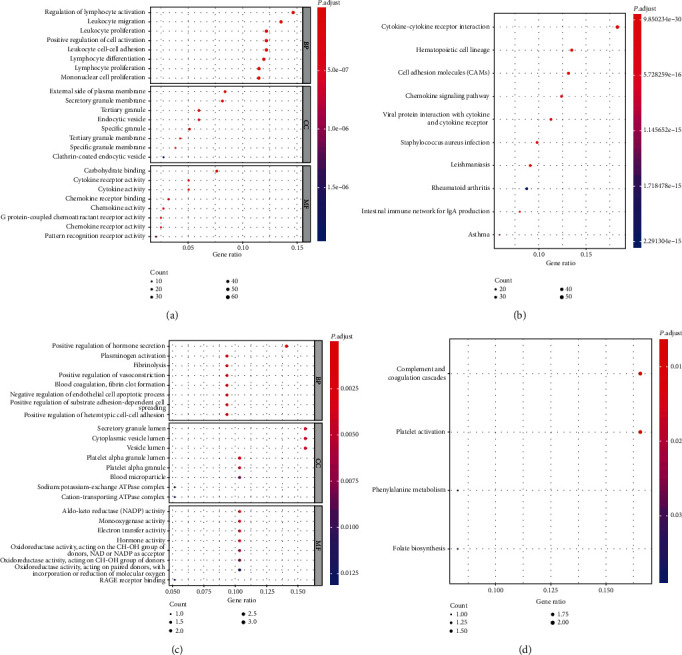
GO and KEGG enrichment analysis of (a, b) upregulated and (c, d) downregulated differentially expressed genes. ClusterProfiler was used to analyze the Gene Ontology (GO) and Kyoto Encyclopedia of Genes and Genomes (KEGG) pathway enrichment involving both, upregulated and downregulated genes. The GO analysis included Biological Process (BP), Cellular Component (CC), and Molecular Function (MF). *P* value < 0.05 and count ≥ 2 were considered significantly enriched. The downregulated DEGs were enriched in 251 GO terms and 4 KEGG pathways. The upregulated DEGs were enriched in 1128 GO terms and 53 KEGG pathways. DEGs represent differentially expressed genes.

The GO enrichment analysis of the 797 DEGs obtained in the previous step showed that the most enriched Biological Process (BP) of the upregulated genes included the regulation of lymphocyte activation and leukocyte migration. The Cellular Components (CC) mainly enriched were the external side of plasma membrane and secretory granule membrane, and the Molecular Functions (MF) mainly enriched were carbohydrate and cytokine receptor activity (Figure 2(a)). KEGG enrichment analysis results of the upregulated DEGs revealed that the most significant enriched pathway was cytokine-cytokine receptor interaction followed by hematopoietic cell lineage and cell adhesion molecules (Figure 2(b)).

For the downregulated DEGs, the BP found to be mainly enriched were positive regulation of hormone secretion and plasminogen activation. For the CC, the enrichment was observed for the platelet alpha granule lumen, while for the MF, the enrichment was observed for the aldo-keto reductase activity (Figure 2(c)). The major enrichment pathways of the downregulated DEGs mainly included complement and coagulation cascades and platelet activation (Figure 2(d)).

### 3.4. Immunocyte Infiltration Abundance Analysis and Screening of T Cell-Related Genes

**Figure 3:**
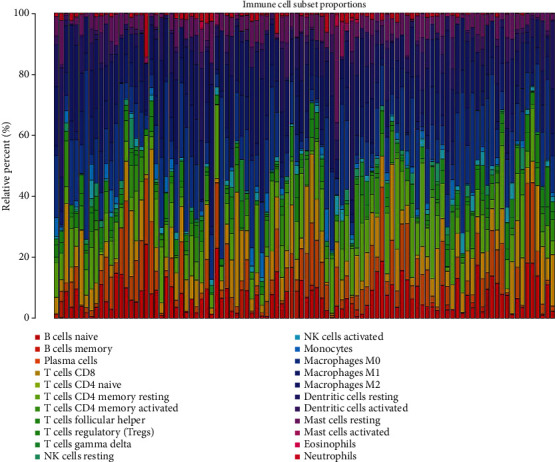
Landscape of the immune cells. To analyze the abundance of infiltration of the immune cells in the samples, RNA-seq expression profile data were used to target the differentially expressed genes, and the abundance matrix of the immune cells was evaluated by using the CIBERSORT deconvolution algorithm. The results showed that 488 of 510 cases were valid (*P* value < 0.05).

Out of the 547 related genes, 539 genes were found to be involved in the LM22 signal matrix, accounting for 98.54%. The infiltration abundance of the 22 types of immune cells was analyzed, and the results are shown in Figure 3. The obtained results showed that 488 samples out of the 510 samples were valid (*P* value < 0.05).

**Figure 4:**
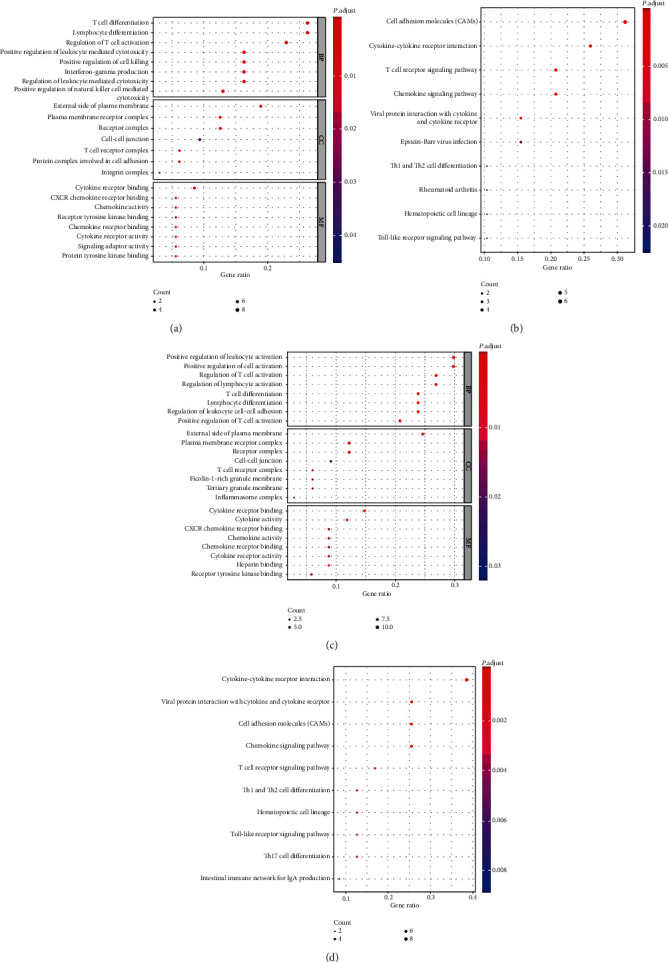
GO and KEGG enrichment analysis of (a, b) CD8(+) T cell-related DEGs and (c, d) memory-activated CD4(+) T cell-related DEGs. ClusterProfiler was used to analyze the Gene Ontology (GO) and Kyoto Encyclopedia of Genes and Genomes (KEGG) pathway enrichment involving both, upregulated and downregulated genes. The GO analysis included Biological Process (BP), Cellular Component (CC) and Molecular Function (MF). *P* value < 0.05 and count ≥ 2 were considered significant enrichment. The CD8(+) T cell-related DEGs were enriched in 338 GO terms and 369 KEGG pathways. The memory-activated CD4(+) T cell-related DEGs were enriched in 12 GO terms and 14 KEGG pathways. DEGs represent differentially expressed genes.

The correlation between the infiltration of CD8(+) T cells and activated memory CD4(+) T cells and the expression level of DEGs were analyzed. A total of 90 DEGs related to the CD8(+) T cell immune cells (Supplementary Table 3) and 48 DEGs related to the activated memory CD4(+) T cells were obtained (Supplementary Table 4). The results of the GO enrichment analysis of CD8(+) T cells related DEGs showed that the BP, CC, and MF mainly enriched were T cell differentiation, external side of plasma membrane, and cytokine receptor binding, respectively (Figure 4(a)). The pathways enriched for these DEGs mainly included cell adhesion molecules and cytokine-cytokine receptor interaction (Figure 4(b)). The GO enrichment analysis results of the activated memory CD4(+) T cell-related DEGs showed that the BP, CC, and MF mainly enriched were positive regulation of leukocyte activation, external side of plasma membrane, and cytokine receptor binding, respectively (Figure 4(c)). The pathways enriched for these DEGs mainly included cytokine-cytokine receptor interaction and viral protein interaction with cytokine and cytokine receptor (Figure 4(d)).

### 3.5. CD8(+) T Cells and Activated Memory CD4(+) T Cell-Related DEGs Are Associated with Prognosis of NSCLC-LUAD

**Figure 5:**
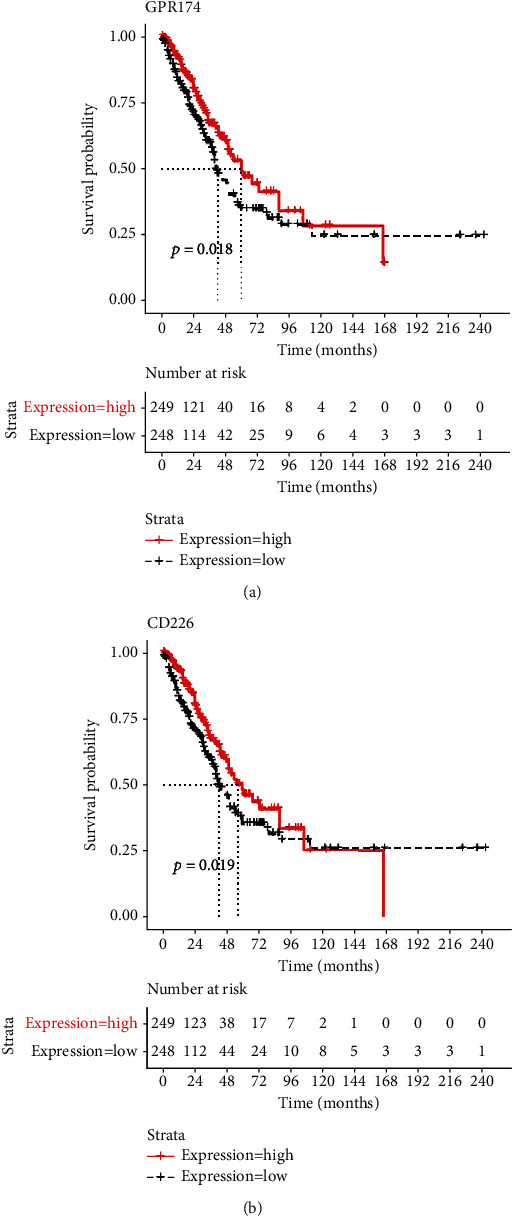
Kaplan-Meier survival curves of DEGs related with immune cells. Package survival (version 2.44-1.1) was used to analyze the relationship between immune-related DEGs of two types of T cells, and the survival of the samples was evaluated. According to the median gene expression, samples were divided into two groups with high and low expression and were tested by performing the log-rank test. The threshold was set as *P* value < 0.05. Patients with a high level of GPR174 and CD226 had a better overall survival than those with low expression levels.

The results of K-M survival curve analysis revealed that among the 90 DEGs related to the CD8(+) T cells, 23 DEGs were associated with the survival prognosis of patients with LUAD, while among the 48 DEGs related to the activated memory CD4(+) T cells, 4 DEGs were associated with the survival prognosis of patients with LUAD. GPR174 and CD226 were suggested as the potential candidates for favorable prognosis of NSCLC-LUAD (Figure 5).

### 3.6. PPI Network of the DEGs Related to CD8(+) T Cells and Activated Memory CD4(+) T Cells

**Figure 6:**
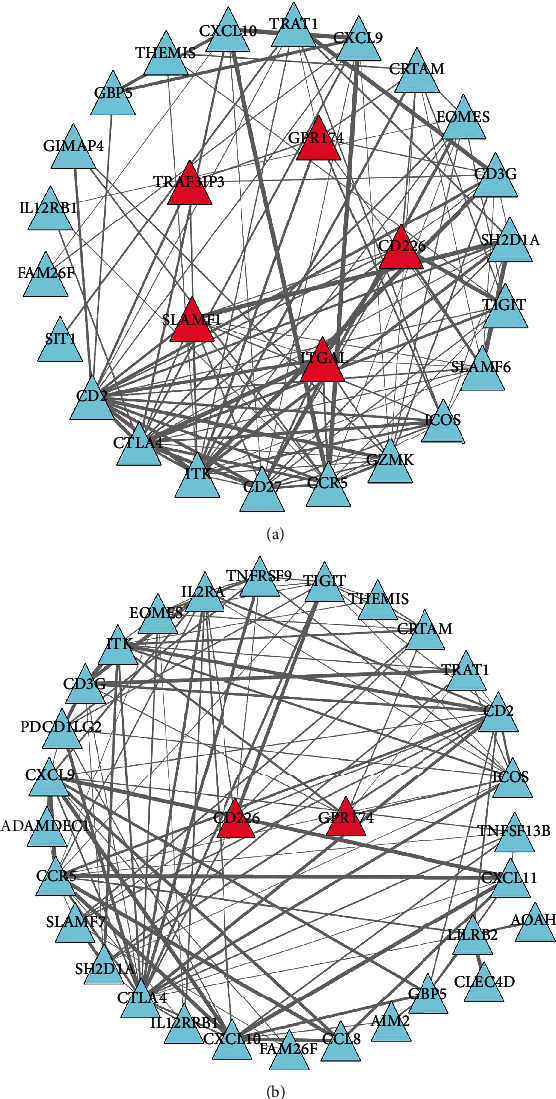
The PPI network of (a) CD8(+) T cell-related DEGs and (b) memory-activated CD4(+) T cell-related DEGs. The interaction between gene coding proteins was predicted and analyzed using the STRING database. The PPI score was set at 0.4. After the PPI edges were obtained, Cytoscape software was used to construct a network for them, and survivor-related differential genes were identified from the network. The CytoNCA plug-in of the Cytoscape software was applied to analyze the topological properties (betweenness, closeness, and degree) of the network. Red nodes represent the upregulated mRNAs while blue nodes represent the downregulated mRNAs. The size of nodes represents the corresponding value. Larger nodes indicate a larger value. DEGs represent differentially expressed genes.

For CD8(+) T cells, 112 PPI edges were obtained. A total of 27 nodes were obtained in the network, and all of them were upregulated. Among these 27 nodes, five (TRAF2IP3, GPR174, CD226, SLAMF1, and ITGAL) were identified as the survival-related genes (Figure 6(a)). For activated memory CD4(+) T cells, 113 PPI edges were obtained. A total of 31 nodes were obtained in the network, and all of them were upregulated. Among these, two (CD226 and GPR174) were identified as the survival-related genes (Figure 6(b)).

### 3.7. ceRNA Network Analysis of CD8(+) T Cells and Activated Memory CD4(+) T Cell-Related RNAs

**Figure 7:**
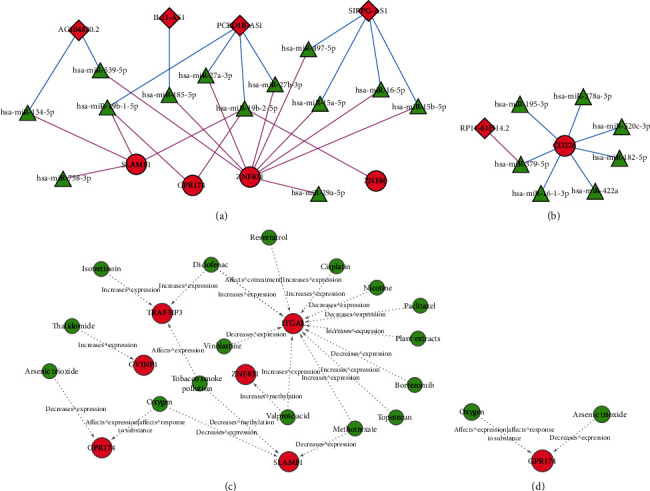
ceRNA network of (a) CD8(+) T cell-related DEGs and (b) memory-activated CD4(+) T cell-related DEGs and chemical small molecule-target network analysis of (c) CD8(+) T cell-related DEGs and (d) memory-activated CD4(+) T cell-related DEGs. The lncRNA-miRNA interaction of the immune-related genes was predicted by using the relevant database (miRWalk3.0, TargetScan, MiRDB, and MirTarBase), with a threshold score > 0.95. The prediction module of DIANA-LncBase v.2 database was used to predict the miRNAs that might be related to lncRNAs, which were related with CD8(+) T cell- and memory-activated CD4(+) T cells. Based on the lncRNA-miRNA and miRNA-target interaction edges, the lncRNA-miRNA-mRNA network was constructed utilizing the Cytoscape software. The red diamond represents the lncRNAs, the green triangle represents the miRNAs, and the red circle represents the mRNAs. The “Carcinoma, Non-Small-Cell Lung” was used as the keyword in the Comparative Toxicogenomics Database to search for lung cancer-related genes and chemicals. Besides, the overlapping genes that were associated with lung cancer and belonged to the T cells related genes in the ceRNA network were used to screen the chemical-target edges. Furthermore, the small chemical molecule-target network of the T cells was generated by using the Cytoscape software. The CD8(+) T cell-related DEG's group involved 6 mRNAs and 16 small chemical molecule drugs. The memory-activated CD4(+) T cell-related DEG's group involved 1 mRNAs and 2 small molecule drugs. Red represents mRNA, and green represents small molecule drugs.

According to the description in the material methods section, the ceRNA network was constructed. A total of 26 edges were obtained in the ceRNA network of the CD8(+) T cell-related RNAs. The network also included 4 lncRNAs, 11 miRNAs, and 4 mRNAs. In this network, GPR174 was found to be regulated by hsa-miR-19b-5p and hsa-miR-19b-2-5p, and both of these two miRNAs were regulated by the lncRNA, PCED1B-AS1. ZNF80 was also found to be regulated by hsa-miR-19b-2-5p, and hsa-miR-19b-2-5p was regulated by PCED1B-AS1 (Figure 7(a)).

In the ceRNA network of the activated memory CD4(+) T cell-related RNAs, there were 7 miRNAs, 1 lncRNA, and 1 mRNA. CD226 was found to be regulated by hsa-miR-379-5p, which was further regulated by the lncRNA, RP11-81H14.2 (Figure 7(b)).

### 3.8. Small Chemical Molecule Analysis of CD8(+) T Cells and Activated Memory CD4(+) T Cell-Related Genes

For CD8(+) T cells, 16 small chemical molecules and 6 mRNAs (associated with prognosis) were obtained from the drug-gene regulatory network (Figure 7(c)). There were two small chemical molecules associated with GPR174 that were identified from the activated memory CD4(+) T cell-related network. Arsenic trioxide has been shown to decrease the expression of GPR174. Moreover, oxygen could not only affect the expression of GPR174 but also affect its response to the substrates (Figure 7(d)).

## 4. Discussion

The immune response against cancer requires specific activation and amplification of T lymphocytes that are killed upon recognition by the antigenic targets expressed by cancer cells. Thus, T lymphocytes have become the current focus of tumor immunotherapy. However, the molecular mechanism of T cell-related RNAs in NSCLC remains unclear. In this study, we analyzed the RNA-seq data of NSCLC-LUAD downloaded from different databases, screened out the DEGs related to CD8(+) T cells and activated memory CD4(+) T cells, and finally constructed the ceRNA networks of the putative prognostic DEGs. Finally, the corresponding small chemical molecules associated with the target genes were predicted, and the regulation network was constructed. In this study, a total of 90 and 48 DEGs related to CD8(+) T cells and activated memory CD4(+) T cells were obtained, respectively. The survival analysis revealed that the T cell genes, CD226 and GPR174, were related with the prognosis of NSCLC-LUAD. PCED1B-AS1-hsa-miR-19b-1-5p/hsa-miR-19b-2-5p-GPR174 and RP11-81H14.2-hsa-miR-379-5p-CD226 were the two potential molecular complexes which were identified to be related to the prognosis of NSCLC-LUAD.

GPR174 is a G protein-coupled receptors, which is activated by the bioactive lipid lysophosphatidylserine (LysoPS) [34]. LysoPS inhibits T cell proliferation and the production of regulatory T cells (Treg) *in vitro* by activating GPR174. Previous studies have shown that an antagonist of GPR174 may have therapeutic potential to promote immune regulation in autoimmune diseases [35]. In this study, GPR174 was found to be regulated by hsa-miR-19b-5p and hsa-miR-19b-2-5p, and both of these miRNAs were regulated by the lncRNA, PCED1B-AS1 (lncRNA, PC-esterase domain containing protein 1B antisense RNA 1). PCED1B-AS1 has been shown to be closely related to tuberculosis. Studies have shown that the expression of PCED1B-AS1 is downregulated in patients with active tuberculosis, and in turn, the apoptosis of monocytes is significantly reduced, and autophagy is enhanced. Meanwhile, it has also been shown that the *in vitro* knockdown of PCED1B-AS1 can reduce apoptosis of macrophages and further promote autophagy. This study also demonstrated that the binding of PCED1B-AS1 with miR-155 can regulate the apoptosis and autophagy of macrophages during active tuberculosis [36]. Another study has shown that the silencing of PCED1B-AS1 leads to an effect that reduces the proliferation capacity of glioma cells, while the inhibition of the miR-194-5p expression can counteract this effect [37]. Therefore, we hypothesize that high expression of PCED1B-AS1 may lead to an increase on the expression of the mRNA, GPR174 by more binding to the target miRNA, including hsa-miR-19b-5p and hsa-miR-19b-2-5p. In this study, PCED1B-AS1 and GPR174 were found to be highly expressed in cancer tissues. Thus, this may be a potential NSCLC-LUAD-related ceRNA complex.

The immunoglobulin-like glycoprotein CD226 (DNAX accessory molecule-1), which is a transmembrane glycoprotein, plays a critical role in the detection of tumors and to study the autoimmune diseases. The level of soluble CD226 in serum of tumor patients was found to be significantly higher than that of healthy individuals [38]. Meanwhile, CD226 gene polymorphisms were identified as risk factors associated with NSCLC [39]. In this study, the ceRNA networks related to activated memory CD4(+) T cells revealed that CD226 was regulated by hsa-miR-379-5p, and at the same time, hsa-miR-379-5p was regulated by lncRNA, RP11-81H14.2. In a study on miR-379 and NSCLC, the researchers found that the miR-379 might be an inhibitory gene related to NSCLC, which could inhibit the cell growth and proliferation, and further promote cell apoptosis. In addition, it was found that miR-379 might also be inhibited in the process of tumor development, but it can reduce some of the malignant damages caused by tumors by inhibiting the expression of the conserved helix-loop-helix ubiquitous kinase (CHUK) [40]. It has been shown that the overexpression of miR-379-5p inhibits the migration, invasion, and metastasis of hepatocellular carcinoma (HCC) cells. However, the knockdown of miR-379-5p leads to an increase in the migration and invasion of hepatocellular carcinoma cells. miR-379-5p can inhibit the expression of focal adhesion ligase by targeting CHUK and further reverse the anticancer effect of miR-379-5p in HCC cells [41]. Wang et al. found that the lncRNA, RP11-81H14.2, was significantly upregulated in HCC cells [42]. Thus, the RP11-81H14.2-hsa-miR-379-5p-CD226 axis may act as a possible regulatory molecular pathway in case of NSCLC-LUAD.

In this study, we found that GPR174 and CD226 could act as favorable prognosis markers of NSCLC-LUAD. Groups with higher Stromal and Immune scores showed high levels of GPR174 and CD226, and higher scores are predicted as lower disease progression than those with lower scores. This suggested that the T cell-mediated immunity might be involved in the regulation of tumor. However, the prognosis depends on a variety of factors, such as the destruction and reconstruction of the immune system in patients with advanced tumors, the negative feedback of the immune system, the tolerance of immune cells, and the direct effects of the antitumor drugs. Therefore, the expression levels were not sufficient to determine the prognosis. We also identified several ceRNA axes that were associated with LC, but these axes have not been reported in the literature yet. Therefore, more experiments were required to perform in the future to verify the assumptions obtained from the bioinformatics analyses of this study. We predicted the small chemical molecules, which associated with T cell-related prognostic indicators. These results can further provide clinical guidance for prognosis judgment and treatment of LC from the perspective of immunity. In this study, multiple NSCLC-LUAD ceRNA networks associated with T cells were identified by bioinformatics analyses. Meanwhile, factors and drugs related to the prognosis of LC were identified, which will also provide clinical guidance for the prognosis judgment and the treatment of LC.

## 5. Conclusion

Our findings provide a deeper understanding of the T cell-related ceRNA regulatory mechanism in NSCLC-LUAD pathogenesis.

## Data Availability

The authors declared that data was available. Gene expression RNA-seq data and clinical data for the lung adenocarcinoma can be downloaded from TCGA dataset, which is included in the XENA database (https://xenabrowser.net/).

## Abbreviations

LC: Lung cancer
OS: Overall survival
NSCLC: Non-small-cell lung cancer
LUAD: Lung adenocarcinomas
CXCR4: C-X-C Motif Chemokine Receptor 4
CXCL12: C-X-C Motif Chemokine Ligand 12
CCL20: C-C motif Chemokine Ligand 20
CCR6: C-C motif Chemokine Receptor 6
FHIT: Fragile Histidine Triad Diadenosine Triphosphatase
RB: Retinoblastoma
TP53: Tumor Protein P53
CDKN2: Cyclin-Dependent Kinase Inhibitor 2
lncRNA: Long noncoding RNA
HIF-1*α*: Hypoxia-inducible factor-1*α*
PVT1: Plasmacytoma variant translocation 1
TCGA: The Cancer Genome Atlas
PPI: Protein-protein interaction
GO: Gene ontology
KEGG: Kyoto Encyclopedia of Genes and Genomes
ceRNA: Competing endogenous RNA
miRNA: MicroRNA
BP: Biological Process
CC: Cellular Component
MF: Molecular Function
CTD: Comparative Toxicogenomics Database
DEGs: Differentially expressed genes
GPR174: G protein-coupled receptor 174
LysoPS: Lysophosphatidylserine
Treg: Regulatory T cells
PCED1B-AS1: PC-esterase domain-containing protein 1B Antisense RNA 1
CD226: DNAX accessory molecule-1 (DNAM-1)
CHUK: Conserved helix-loop-helix ubiquitous kinase
HCC: Hepatocellular carcinoma.

## Additional Points

*Highlights*. A total of 90 differentially expressed genes (DEGs) were found to be related to CD8(+) T immune cells, and 48 DEGs were related to activated memory CD4(+) T cells. CD226 and GPR174 were related with the prognosis of non-small-cell lung cancer (NSCLC). The constructed ceRNA network provides a deeper understanding of the T cell-related ceRNA regulatory mechanism in NSCLC-LUAD.

## Supplementary Materials

Supplementary 1 Supplementary Table 1: genes sorted by Stromal score.

Supplementary 2 Supplementary Table 2: genes sorted by Immune score.

Supplementary 3 Supplementary Table 3: genes related with CD8 T cells.

Supplementary 4 Supplementary Table 4: genes related with memory-activated CD4 T cells.
