# Supplementary material for: Screening of T Cell-Related Long Noncoding RNA-MicroRNA-mRNA Regulatory Networks in Non-Small-Cell Lung Cancer
Source: Biomed Res Int. 2020 Nov 14;2020:5816763. doi: 10.1155/2020/5816763 (PMC7684158; doi:10.1155/2020/5816763)
Supplement: Supplementary 1 — Supplementary Table 1: genes sorted by Stromal score. [file 5816763.f1.docx]

Supplementary Table 1 Genes sorted by Stromal score.

| gene | type | change | logFC | AveExpr | t | P.Value | adj.P.Val | B |
| --- | --- | --- | --- | --- | --- | --- | --- | --- |
| DSCAM-AS1 | lncRNA | DOWN | -1.72327 | -3.19384 | -4.22755 | 2.80E-05 | 0.000199 | 1.96515 |
| LINC00473 | lncRNA | DOWN | -1.45558 | -2.43371 | -4.0599 | 5.67E-05 | 0.000379 | 1.280829 |
| CASC9 | lncRNA | DOWN | -1.35805 | -1.24871 | -4.01829 | 6.74E-05 | 0.000443 | 1.063799 |
| RP11-499O7.7 | lncRNA | DOWN | -1.33285 | -2.64007 | -5.64583 | 2.72E-08 | 3.39E-07 | 8.544551 |
| RP11-21L23.4 | lncRNA | DOWN | -1.23198 | -3.82811 | -5.49999 | 6.00E-08 | 7.06E-07 | 7.806707 |
| RP11-148B3.2 | lncRNA | DOWN | -1.14983 | -3.28114 | -4.53554 | 7.16E-06 | 5.77E-05 | 3.254859 |
| RP11-284F21.9 | lncRNA | DOWN | -1.1226 | 0.061945 | -4.42757 | 1.16E-05 | 8.99E-05 | 2.637188 |
| PART1 | lncRNA | DOWN | -1.10994 | -2.86034 | -4.36328 | 1.55E-05 | 0.000117 | 2.516054 |
| RP11-672A2.1 | lncRNA | DOWN | -1.10543 | -4.4762 | -5.00122 | 7.83E-07 | 7.57E-06 | 5.341648 |
| RP1-27K12.2 | lncRNA | DOWN | -1.10278 | -2.32715 | -2.9553 | 0.003267 | 0.013798 | -2.45821 |
| KCNMB2-AS1 | lncRNA | DOWN | -1.10081 | -1.28184 | -3.92505 | 9.85E-05 | 0.000624 | 0.710378 |
| RP11-874J12.4 | lncRNA | DOWN | -1.07805 | -3.90831 | -5.31364 | 1.60E-07 | 1.75E-06 | 6.868268 |
| AC011298.2 | lncRNA | DOWN | -1.05572 | -3.92517 | -4.3605 | 1.57E-05 | 0.000118 | 2.522757 |
| CTD-2139B15.5 | lncRNA | DOWN | -1.03153 | -4.07064 | -4.03593 | 6.27E-05 | 0.000415 | 1.221731 |
| RP11-53B5.1 | lncRNA | DOWN | -1.03048 | -3.9619 | -4.32466 | 1.84E-05 | 0.000136 | 2.374339 |
| NOVA1-AS1 | lncRNA | DOWN | -1.02715 | -3.42174 | -3.59105 | 0.000361 | 0.002004 | -0.40759 |
| RP11-713C5.1 | lncRNA | UP | 1.786249 | -2.46578 | 8.903077 | 9.35E-18 | 3.73E-16 | 29.64548 |
| AC093850.2 | lncRNA | UP | 1.718971 | -0.10845 | 11.48171 | 2.54E-27 | 2.22E-25 | 51.23029 |
| RP11-554A11.4 | lncRNA | UP | 1.527656 | -2.24414 | 14.1862 | 8.74E-39 | 2.13E-36 | 76.96493 |
| RP11-417E7.2 | lncRNA | UP | 1.516246 | -1.80523 | 9.513406 | 7.11E-20 | 3.42E-18 | 34.40788 |
| RP11-95H3.1 | lncRNA | UP | 1.507643 | -3.0886 | 12.77473 | 1.19E-32 | 1.60E-30 | 63.09627 |
| PTPRD-AS1 | lncRNA | UP | 1.494682 | -2.3568 | 13.0269 | 1.01E-33 | 1.44E-31 | 65.5557 |
| AC109642.1 | lncRNA | UP | 1.466816 | 0.8255 | 13.43645 | 1.73E-35 | 2.89E-33 | 69.76199 |
| RP11-449D8.1 | lncRNA | UP | 1.447413 | -2.74493 | 11.92754 | 4.01E-29 | 4.04E-27 | 55.18051 |
| WT1-AS | lncRNA | UP | 1.441344 | -3.44051 | 7.921029 | 1.47E-14 | 4.19E-13 | 22.49106 |
| RP11-276H19.1 | lncRNA | UP | 1.438006 | -2.37816 | 13.48951 | 1.02E-35 | 1.75E-33 | 70.04816 |
| RP11-401O9.4 | lncRNA | UP | 1.432201 | -4.29271 | 9.815332 | 5.86E-21 | 3.10E-19 | 36.82611 |
| RP11-513N24.1 | lncRNA | UP | 1.430051 | -1.95496 | 11.73295 | 2.48E-28 | 2.31E-26 | 53.43722 |
| IFNG-AS1 | lncRNA | UP | 1.428549 | -2.16298 | 9.016753 | 3.83E-18 | 1.58E-16 | 30.51561 |
| RP11-77A13.1 | lncRNA | UP | 1.419667 | -3.47351 | 6.084195 | 2.29E-09 | 3.40E-08 | 10.93511 |
| RP5-1172A22.1 | lncRNA | UP | 1.40202 | -2.92614 | 11.27845 | 1.64E-26 | 1.34E-24 | 49.30279 |
| RP11-1008C21.1 | lncRNA | UP | 1.401833 | 0.094057 | 10.13017 | 4.12E-22 | 2.40E-20 | 39.46163 |
| LINC00968 | lncRNA | UP | 1.394373 | -1.26684 | 11.28929 | 1.48E-26 | 1.22E-24 | 49.46342 |
| CTD-2171N6.1 | lncRNA | UP | 1.393624 | -1.67721 | 7.966086 | 1.06E-14 | 3.08E-13 | 22.79545 |
| LINC01266 | lncRNA | UP | 1.393268 | -2.64735 | 13.0995 | 4.92E-34 | 7.28E-32 | 66.23533 |
| LINC01561 | lncRNA | UP | 1.369641 | -3.59522 | 7.802444 | 3.42E-14 | 9.32E-13 | 21.6715 |
| TBX5-AS1 | lncRNA | UP | 1.369353 | 1.992724 | 15.50454 | 9.29E-45 | 4.30E-42 | 90.88283 |
| RP11-383H13.1 | lncRNA | UP | 1.365107 | 1.367762 | 16.03848 | 3.12E-47 | 2.05E-44 | 96.45723 |
| LINC01094 | lncRNA | UP | 1.347919 | 1.678844 | 14.61378 | 1.06E-40 | 3.06E-38 | 81.63651 |
| RP5-839B4.8 | lncRNA | UP | 1.347563 | -1.86483 | 6.576325 | 1.19E-10 | 2.12E-09 | 13.75611 |
| RP11-21C4.1 | lncRNA | UP | 1.346694 | -2.78523 | 12.75013 | 1.52E-32 | 1.99E-30 | 62.87638 |
| RP11-750H9.5 | lncRNA | UP | 1.340536 | 0.375362 | 15.21575 | 1.97E-43 | 7.32E-41 | 87.73868 |
| LINC01150 | lncRNA | UP | 1.338652 | -2.8705 | 12.46304 | 2.45E-31 | 2.92E-29 | 60.15411 |
| MEOX2-AS1 | lncRNA | UP | 1.327858 | -4.03585 | 10.34018 | 6.79E-23 | 4.23E-21 | 41.16339 |
| LINC00702 | lncRNA | UP | 1.32583 | -0.23663 | 13.31098 | 6.05E-35 | 9.65E-33 | 68.46757 |
| LINC00973 | lncRNA | UP | 1.320428 | -3.16806 | 4.779578 | 2.30E-06 | 2.03E-05 | 4.327523 |
| AF127936.5 | lncRNA | UP | 1.316709 | -2.49192 | 11.69179 | 3.64E-28 | 3.34E-26 | 53.03695 |
| RP11-290O12.2 | lncRNA | UP | 1.314831 | -3.89441 | 10.85388 | 7.49E-25 | 5.40E-23 | 45.555 |
| RP11-863P13.3 | lncRNA | UP | 1.314487 | -2.25179 | 9.023337 | 3.64E-18 | 1.51E-16 | 30.56561 |
| CTD-2033D15.2 | lncRNA | UP | 1.3081 | -1.69095 | 10.48089 | 2.00E-23 | 1.30E-21 | 42.39609 |
| AP000476.1 | lncRNA | UP | 1.302705 | -2.39024 | 12.96261 | 1.90E-33 | 2.64E-31 | 64.93396 |
| AC096579.13 | lncRNA | UP | 1.289451 | -2.41302 | 8.036293 | 6.39E-15 | 1.92E-13 | 23.29463 |
| RP4-564M11.2 | lncRNA | UP | 1.280767 | -3.26977 | 10.33055 | 7.38E-23 | 4.60E-21 | 41.08447 |
| GS1-600G8.5 | lncRNA | UP | 1.278354 | -2.24614 | 7.212488 | 1.98E-12 | 4.38E-11 | 17.72724 |
| RP11-180N14.1 | lncRNA | UP | 1.27717 | -2.81386 | 10.63984 | 4.98E-24 | 3.42E-22 | 43.72381 |
| AC112721.1 | lncRNA | UP | 1.2722 | -3.98078 | 9.486993 | 8.82E-20 | 4.21E-18 | 34.177 |
| RP11-89B16.1 | lncRNA | UP | 1.26951 | -4.05936 | 12.03618 | 1.44E-29 | 1.51E-27 | 56.15337 |
| RP11-401O9.3 | lncRNA | UP | 1.268597 | -4.6715 | 8.772223 | 2.58E-17 | 9.89E-16 | 28.65638 |
| RP11-542B15.1 | lncRNA | UP | 1.267282 | -2.45882 | 12.38467 | 5.20E-31 | 6.13E-29 | 59.44022 |
| RP11-92C4.6 | lncRNA | UP | 1.266902 | -3.40998 | 10.26403 | 1.31E-22 | 7.98E-21 | 40.52354 |
| LINC01013 | lncRNA | UP | 1.265038 | -3.71282 | 9.531473 | 6.13E-20 | 2.97E-18 | 34.53047 |
| LINC00402 | lncRNA | UP | 1.263941 | -2.9239 | 8.107442 | 3.81E-15 | 1.17E-13 | 23.79882 |
| RP11-733O18.1 | lncRNA | UP | 1.243771 | -2.49372 | 10.7672 | 1.62E-24 | 1.15E-22 | 44.8307 |
| RP11-693J15.5 | lncRNA | UP | 1.243657 | -2.19843 | 8.520931 | 1.76E-16 | 6.26E-15 | 26.78546 |
| RP11-354E11.2 | lncRNA | UP | 1.243473 | -1.39546 | 11.06867 | 1.09E-25 | 8.44E-24 | 47.50108 |
| AC112721.2 | lncRNA | UP | 1.240536 | -3.43599 | 8.596208 | 9.96E-17 | 3.63E-15 | 27.3386 |
| HAS2-AS1 | lncRNA | UP | 1.236158 | -1.79213 | 10.72507 | 2.35E-24 | 1.65E-22 | 44.48837 |
| LINC01352 | lncRNA | UP | 1.232183 | -2.94664 | 11.33352 | 9.90E-27 | 8.22E-25 | 49.79168 |
| RP11-247C2.2 | lncRNA | UP | 1.228399 | -1.5996 | 9.865198 | 3.86E-21 | 2.07E-19 | 37.25438 |
| RP11-472N13.3 | lncRNA | UP | 1.227538 | -3.18661 | 10.88714 | 5.57E-25 | 4.06E-23 | 45.85103 |
| RP5-887A10.1 | lncRNA | UP | 1.221907 | -2.09914 | 7.642213 | 1.05E-13 | 2.71E-12 | 20.56967 |
| RP11-626H12.2 | lncRNA | UP | 1.221254 | -3.36438 | 8.373962 | 5.33E-16 | 1.79E-14 | 25.70988 |
| INHBA-AS1 | lncRNA | UP | 1.217319 | -2.71265 | 11.33875 | 9.44E-27 | 7.87E-25 | 49.84698 |
| LINC00840 | lncRNA | UP | 1.216601 | -4.03382 | 7.570103 | 1.74E-13 | 4.36E-12 | 20.09383 |
| RP1-137D17.1 | lncRNA | UP | 1.216149 | -1.16255 | 9.771461 | 8.45E-21 | 4.42E-19 | 36.49463 |
| RP11-219E7.1 | lncRNA | UP | 1.215439 | -1.9738 | 12.98989 | 1.45E-33 | 2.05E-31 | 65.22563 |
| LINC01235 | lncRNA | UP | 1.215014 | -1.64786 | 6.882592 | 1.72E-11 | 3.40E-10 | 15.62174 |
| RP11-417E7.1 | lncRNA | UP | 1.213475 | -2.17011 | 8.834556 | 1.59E-17 | 6.22E-16 | 29.12685 |
| RP11-366L20.2 | lncRNA | UP | 1.208677 | -1.65802 | 7.83375 | 2.74E-14 | 7.56E-13 | 21.87457 |
| RP1-309I22.2 | lncRNA | UP | 1.206842 | 1.102605 | 9.741748 | 1.08E-20 | 5.59E-19 | 36.2429 |
| RP11-1018N14.5 | lncRNA | UP | 1.203538 | -2.32543 | 10.14049 | 3.77E-22 | 2.21E-20 | 39.51361 |
| OR7E47P | lncRNA | UP | 1.203112 | 0.033916 | 12.36113 | 6.52E-31 | 7.66E-29 | 59.35767 |
| AL928768.3 | lncRNA | UP | 1.202922 | -1.44972 | 7.691826 | 7.45E-14 | 1.95E-12 | 20.89718 |
| DNM3OS | lncRNA | UP | 1.195124 | 1.059603 | 14.37942 | 1.20E-39 | 3.19E-37 | 79.21078 |
| CTB-114C7.4 | lncRNA | UP | 1.193313 | -2.99808 | 11.20348 | 3.23E-26 | 2.58E-24 | 48.63386 |
| RP11-768B22.2 | lncRNA | UP | 1.191448 | -2.78947 | 8.204007 | 1.88E-15 | 5.93E-14 | 24.48587 |
| AC083949.1 | lncRNA | UP | 1.187618 | -2.01076 | 10.45036 | 2.61E-23 | 1.69E-21 | 42.12792 |
| RP11-428G5.5 | lncRNA | UP | 1.187573 | -3.13221 | 7.76658 | 4.40E-14 | 1.19E-12 | 21.42462 |
| RP11-758N13.1 | lncRNA | UP | 1.187297 | -3.75149 | 7.829297 | 2.82E-14 | 7.79E-13 | 21.85659 |
| RP11-121A8.1 | lncRNA | UP | 1.186143 | -1.06841 | 13.53862 | 6.22E-36 | 1.08E-33 | 70.63782 |
| MYHAS | lncRNA | UP | 1.185585 | -4.48437 | 9.418947 | 1.53E-19 | 7.18E-18 | 33.64567 |
| AC079767.4 | lncRNA | UP | 1.176296 | -0.79794 | 9.552347 | 5.17E-20 | 2.52E-18 | 34.72642 |
| RP4-598P13.1 | lncRNA | UP | 1.16606 | -4.42402 | 9.440643 | 1.29E-19 | 6.05E-18 | 33.81541 |
| AC090044.2 | lncRNA | UP | 1.163811 | -3.78363 | 9.402345 | 1.76E-19 | 8.16E-18 | 33.50513 |
| IL21-AS1 | lncRNA | UP | 1.161823 | -3.55145 | 7.897069 | 1.74E-14 | 4.91E-13 | 22.32496 |
| RP11-556E13.1 | lncRNA | UP | 1.161589 | -2.76052 | 9.359202 | 2.49E-19 | 1.14E-17 | 33.17377 |
| RP11-1070N10.3 | lncRNA | UP | 1.158894 | -2.14764 | 8.793013 | 2.20E-17 | 8.47E-16 | 28.81317 |
| RP11-514D23.3 | lncRNA | UP | 1.158702 | -4.05262 | 9.654797 | 2.22E-20 | 1.12E-18 | 35.51811 |
| RP11-33A14.1 | lncRNA | UP | 1.157933 | -4.31759 | 8.894211 | 1.00E-17 | 3.99E-16 | 29.57375 |
| RP11-307E17.8 | lncRNA | UP | 1.155178 | -2.96369 | 10.20711 | 2.13E-22 | 1.28E-20 | 40.05486 |
| LINC01281 | lncRNA | UP | 1.152603 | -2.48415 | 8.291087 | 9.87E-16 | 3.22E-14 | 25.11068 |
| KIAA0125 | lncRNA | UP | 1.152314 | 1.583567 | 7.407573 | 5.31E-13 | 1.26E-11 | 18.87196 |
| RP11-710C12.1 | lncRNA | UP | 1.148565 | -1.53992 | 13.94237 | 1.05E-37 | 2.24E-35 | 74.59391 |
| LINC01215 | lncRNA | UP | 1.147247 | -0.42621 | 8.746353 | 3.15E-17 | 1.20E-15 | 28.45303 |
| RP11-230G5.2 | lncRNA | UP | 1.147225 | -4.2562 | 5.851532 | 8.68E-09 | 1.17E-07 | 9.653362 |
| RP11-426C22.4 | lncRNA | UP | 1.146719 | -2.12543 | 10.97415 | 2.56E-25 | 1.91E-23 | 46.64459 |
| AF127936.3 | lncRNA | UP | 1.14645 | -2.83151 | 7.90353 | 1.66E-14 | 4.71E-13 | 22.36811 |
| RP11-283G6.3 | lncRNA | UP | 1.144759 | -1.89884 | 8.206186 | 1.85E-15 | 5.85E-14 | 24.4981 |
| CTC-378H22.2 | lncRNA | UP | 1.143745 | -1.85801 | 11.25535 | 2.02E-26 | 1.63E-24 | 49.13788 |
| LINC00582 | lncRNA | UP | 1.129976 | -2.39717 | 7.751753 | 4.89E-14 | 1.31E-12 | 21.31871 |
| AC069363.1 | lncRNA | UP | 1.127027 | -1.62321 | 8.58686 | 1.07E-16 | 3.88E-15 | 27.2714 |
| LINC00924 | lncRNA | UP | 1.126092 | -1.82963 | 9.735745 | 1.14E-20 | 5.86E-19 | 36.19624 |
| XXbac-BPG13B8.10 | lncRNA | UP | 1.12587 | -4.00811 | 7.50464 | 2.73E-13 | 6.69E-12 | 19.65649 |
| RP11-49G2.3 | lncRNA | UP | 1.122351 | -3.88027 | 8.61216 | 8.82E-17 | 3.23E-15 | 27.45598 |
| TRBV11-2 | lncRNA | UP | 1.121643 | -2.44138 | 9.241258 | 6.44E-19 | 2.87E-17 | 32.25208 |
| LINC00922 | lncRNA | UP | 1.116209 | -1.57278 | 7.256161 | 1.48E-12 | 3.31E-11 | 17.99795 |
| RP4-668J24.2 | lncRNA | UP | 1.113252 | -3.03711 | 6.807089 | 2.78E-11 | 5.37E-10 | 15.18044 |
| RP11-166D19.1 | lncRNA | UP | 1.109793 | 3.019071 | 10.60538 | 6.74E-24 | 4.58E-22 | 43.45635 |
| AC108142.1 | lncRNA | UP | 1.106911 | -2.6662 | 7.407264 | 5.32E-13 | 1.26E-11 | 19.00508 |
| AC020571.3 | lncRNA | UP | 1.106489 | -0.35061 | 12.89763 | 3.59E-33 | 4.91E-31 | 64.4491 |
| CTD-2532K18.2 | lncRNA | UP | 1.106488 | -4.20837 | 6.945713 | 1.14E-11 | 2.31E-10 | 16.04393 |
| RP1-34H18.1 | lncRNA | UP | 1.106486 | -4.88968 | 9.423184 | 1.48E-19 | 6.95E-18 | 33.6907 |
| RP11-672A2.4 | lncRNA | UP | 1.105538 | -1.59244 | 11.35038 | 8.48E-27 | 7.10E-25 | 49.99666 |
| RP11-815J21.4 | lncRNA | UP | 1.101049 | -3.08474 | 9.745884 | 1.05E-20 | 5.41E-19 | 36.2581 |
| LINC01055 | lncRNA | UP | 1.100619 | -2.98032 | 7.76012 | 4.61E-14 | 1.24E-12 | 21.37982 |
| RP11-8L8.2 | lncRNA | UP | 1.099789 | -2.228 | 11.60934 | 7.82E-28 | 7.03E-26 | 52.30044 |
| RP11-322E11.5 | lncRNA | UP | 1.098662 | -1.67706 | 11.80119 | 1.31E-28 | 1.24E-26 | 54.07368 |
| RP11-290F5.1 | lncRNA | UP | 1.09719 | 0.55303 | 8.172767 | 2.36E-15 | 7.41E-14 | 24.20972 |
| LBX1-AS1 | lncRNA | UP | 1.095749 | -3.84448 | 8.782987 | 2.38E-17 | 9.11E-16 | 28.73082 |
| AC104699.1 | lncRNA | UP | 1.093553 | -1.81714 | 7.389665 | 6.00E-13 | 1.41E-11 | 18.8772 |
| LINC00494 | lncRNA | UP | 1.093317 | -2.23155 | 6.5736 | 1.21E-10 | 2.16E-09 | 13.74893 |
| AC007750.5 | lncRNA | UP | 1.093186 | -1.76346 | 10.23086 | 1.74E-22 | 1.05E-20 | 40.28111 |
| RP11-536K7.5 | lncRNA | UP | 1.093173 | -4.16756 | 9.373151 | 2.22E-19 | 1.02E-17 | 33.27644 |
| RP11-13P5.2 | lncRNA | UP | 1.090948 | -2.5694 | 8.027018 | 6.84E-15 | 2.04E-13 | 23.23017 |
| CTC-296K1.4 | lncRNA | UP | 1.08965 | -4.26585 | 8.427823 | 3.56E-16 | 1.22E-14 | 26.10175 |
| LINC00996 | lncRNA | UP | 1.088397 | -0.32066 | 10.18567 | 2.56E-22 | 1.52E-20 | 39.92465 |
| DIO3OS | lncRNA | UP | 1.085398 | -0.20336 | 6.252741 | 8.49E-10 | 1.35E-08 | 11.79331 |
| MIR100HG | lncRNA | UP | 1.084041 | -1.76701 | 7.827367 | 2.86E-14 | 7.89E-13 | 21.83196 |
| RP5-907D15.4 | lncRNA | UP | 1.083058 | -2.52961 | 7.361453 | 7.27E-13 | 1.69E-11 | 18.70196 |
| LINC00619 | lncRNA | UP | 1.082975 | -4.26964 | 6.892942 | 1.61E-11 | 3.18E-10 | 15.7142 |
| CTD-2003C8.2 | lncRNA | UP | 1.082235 | -3.29826 | 9.03385 | 3.35E-18 | 1.39E-16 | 30.63839 |
| LINC00890 | lncRNA | UP | 1.079236 | -2.62027 | 5.095457 | 4.90E-07 | 4.91E-06 | 5.782891 |
| LINC00892 | lncRNA | UP | 1.079054 | -1.36298 | 8.663403 | 5.96E-17 | 2.20E-15 | 27.83979 |
| PIK3CD-AS1 | lncRNA | UP | 1.075004 | -3.80487 | 9.502482 | 7.77E-20 | 3.73E-18 | 34.29811 |
| RP11-18H21.1 | lncRNA | UP | 1.0746 | -1.74975 | 9.128685 | 1.58E-18 | 6.81E-17 | 31.38131 |
| LLNLR-470E3.1 | lncRNA | UP | 1.072075 | -1.45649 | 7.98469 | 9.28E-15 | 2.71E-13 | 22.92315 |
| LINC00861 | lncRNA | UP | 1.071 | 0.848173 | 8.715255 | 4.01E-17 | 1.51E-15 | 28.18956 |
| RP11-553L6.2 | lncRNA | UP | 1.068994 | -4.21916 | 8.571159 | 1.21E-16 | 4.34E-15 | 27.15342 |
| RP3-369A17.4 | lncRNA | UP | 1.065178 | -4.19981 | 9.123549 | 1.65E-18 | 7.07E-17 | 31.32849 |
| AL122127.25 | lncRNA | UP | 1.064659 | -3.61021 | 6.967603 | 9.92E-12 | 2.02E-10 | 16.18268 |
| AC013264.2 | lncRNA | UP | 1.064567 | -1.88355 | 5.808143 | 1.11E-08 | 1.47E-07 | 9.385246 |
| CTD-2353F22.1 | lncRNA | UP | 1.061545 | -1.92306 | 9.113012 | 1.79E-18 | 7.66E-17 | 31.25899 |
| RP11-20J15.3 | lncRNA | UP | 1.060868 | -2.44341 | 6.458975 | 2.45E-10 | 4.18E-09 | 13.07271 |
| RP11-598F7.3 | lncRNA | UP | 1.060565 | -1.8729 | 6.722622 | 4.76E-11 | 8.93E-10 | 14.64125 |
| AC011893.3 | lncRNA | UP | 1.058431 | -4.02051 | 8.521419 | 1.76E-16 | 6.24E-15 | 26.78628 |
| FLJ22447 | lncRNA | UP | 1.057455 | -0.64278 | 6.211086 | 1.09E-09 | 1.70E-08 | 11.57468 |
| AFAP1-AS1 | lncRNA | UP | 1.056756 | 4.588312 | 3.992784 | 7.48E-05 | 0.000487 | 0.498428 |
| RP11-567M16.1 | lncRNA | UP | 1.056696 | -1.91021 | 8.963075 | 5.84E-18 | 2.37E-16 | 30.10534 |
| RP11-284N8.3 | lncRNA | UP | 1.056315 | 2.725932 | 7.869823 | 2.12E-14 | 5.91E-13 | 21.95952 |
| RP11-445F6.2 | lncRNA | UP | 1.056198 | -4.48833 | 7.722597 | 6.00E-14 | 1.59E-12 | 21.12283 |
| RP11-756G20.1 | lncRNA | UP | 1.055284 | -3.12912 | 10.85894 | 7.16E-25 | 5.18E-23 | 45.60632 |
| RP11-145A3.1 | lncRNA | UP | 1.055243 | -2.14388 | 7.396981 | 5.71E-13 | 1.35E-11 | 18.93077 |
| AP001434.2 | lncRNA | UP | 1.054041 | -2.50029 | 8.865896 | 1.25E-17 | 4.90E-16 | 29.3627 |
| RP11-536O18.1 | lncRNA | UP | 1.052168 | -4.35376 | 8.043374 | 6.07E-15 | 1.82E-13 | 23.34682 |
| RP11-356N1.2 | lncRNA | UP | 1.051675 | -3.47328 | 8.421939 | 3.72E-16 | 1.27E-14 | 26.05849 |
| RP11-2N1.2 | lncRNA | UP | 1.050848 | -4.2209 | 5.916549 | 6.01E-09 | 8.36E-08 | 10.00957 |
| RP11-524D16__A.3 | lncRNA | UP | 1.050173 | 0.360454 | 9.518549 | 6.81E-20 | 3.29E-18 | 34.44961 |
| PCED1B-AS1 | lncRNA | UP | 1.04937 | 2.939548 | 13.63443 | 2.37E-36 | 4.34E-34 | 71.78338 |
| RP11-54A9.1 | lncRNA | UP | 1.047028 | -2.04783 | 8.815271 | 1.85E-17 | 7.18E-16 | 28.98138 |
| LINC01415 | lncRNA | UP | 1.046121 | -2.16278 | 9.497544 | 8.09E-20 | 3.89E-18 | 34.27705 |
| RP11-679B19.1 | lncRNA | UP | 1.044729 | -1.50568 | 12.9642 | 1.87E-33 | 2.60E-31 | 65.01201 |
| RP11-672L10.2 | lncRNA | UP | 1.042372 | -3.81661 | 6.01292 | 3.46E-09 | 5.01E-08 | 10.54257 |
| RP11-10J5.1 | lncRNA | UP | 1.042259 | -3.59899 | 6.681267 | 6.17E-11 | 1.14E-09 | 14.41739 |
| CH17-360D5.3 | lncRNA | UP | 1.040783 | -2.43404 | 4.854948 | 1.60E-06 | 1.46E-05 | 4.64996 |
| RP11-16K12.1 | lncRNA | UP | 1.035613 | -1.24371 | 6.766523 | 3.60E-11 | 6.85E-10 | 14.89268 |
| SIRPG-AS1 | lncRNA | UP | 1.033687 | -3.83043 | 7.351187 | 7.79E-13 | 1.81E-11 | 18.64315 |
| SRGAP3-AS2 | lncRNA | UP | 1.032531 | -1.96299 | 3.656111 | 0.000282 | 0.001607 | -0.23454 |
| LINC01197 | lncRNA | UP | 1.032507 | -1.25553 | 11.67863 | 4.11E-28 | 3.75E-26 | 52.97456 |
| RP11-340F14.6 | lncRNA | UP | 1.032236 | -3.25242 | 9.723102 | 1.26E-20 | 6.46E-19 | 36.0712 |
| RP11-400K9.4 | lncRNA | UP | 1.029629 | -0.13353 | 7.752673 | 4.86E-14 | 1.30E-12 | 21.28153 |
| LINC00877 | lncRNA | UP | 1.029066 | -1.78073 | 10.54012 | 1.19E-23 | 7.90E-22 | 42.89889 |
| LINC00944 | lncRNA | UP | 1.028412 | -1.30186 | 6.739183 | 4.29E-11 | 8.10E-10 | 14.72679 |
| MAGI2-AS3 | lncRNA | UP | 1.028348 | 3.237532 | 14.16702 | 1.06E-38 | 2.58E-36 | 77.13498 |
| AC104820.2 | lncRNA | UP | 1.027092 | -2.12403 | 8.827362 | 1.69E-17 | 6.56E-16 | 29.07256 |
| LINC01010 | lncRNA | UP | 1.026887 | -1.74227 | 7.428211 | 4.61E-13 | 1.10E-11 | 19.13073 |
| L3MBTL4-AS1 | lncRNA | UP | 1.019172 | -1.70217 | 11.41569 | 4.66E-27 | 3.98E-25 | 50.57744 |
| RP11-117D22.2 | lncRNA | UP | 1.018937 | -3.37779 | 8.509767 | 1.92E-16 | 6.78E-15 | 26.70123 |
| RP11-532F6.3 | lncRNA | UP | 1.018283 | -0.30029 | 10.2053 | 2.17E-22 | 1.29E-20 | 40.08954 |
| CTB-41I6.1 | lncRNA | UP | 1.016366 | -2.45025 | 9.191891 | 9.55E-19 | 4.19E-17 | 31.86708 |
| RP11-325F22.2 | lncRNA | UP | 1.01484 | -1.14832 | 9.765659 | 8.87E-21 | 4.63E-19 | 36.44753 |
| RP11-81H14.2 | lncRNA | UP | 1.010638 | -0.8157 | 7.938189 | 1.30E-14 | 3.72E-13 | 22.58626 |
| AC009784.3 | lncRNA | UP | 1.009973 | -4.47 | 7.066209 | 5.21E-12 | 1.10E-10 | 16.79982 |
| RP11-327F22.2 | lncRNA | UP | 1.009423 | -1.89012 | 10.41317 | 3.61E-23 | 2.30E-21 | 41.81526 |
| AP000695.6 | lncRNA | UP | 1.007683 | -1.37763 | 8.340809 | 6.82E-16 | 2.25E-14 | 25.46448 |
| MIR143HG | lncRNA | UP | 1.00755 | -0.04965 | 10.96376 | 2.81E-25 | 2.08E-23 | 46.61317 |
| MEG3 | lncRNA | UP | 1.006329 | 3.42923 | 6.252446 | 8.50E-10 | 1.35E-08 | 11.55535 |
| DKFZp434J0226 | lncRNA | UP | 1.004975 | -2.366 | 6.159693 | 1.47E-09 | 2.25E-08 | 11.3414 |
| CTC-378H22.1 | lncRNA | UP | 1.002826 | -3.29819 | 8.796994 | 2.13E-17 | 8.22E-16 | 28.83764 |
| RP5-1091N2.9 | lncRNA | UP | 1.001369 | -1.17409 | 11.5232 | 1.73E-27 | 1.53E-25 | 51.56795 |
| AC007386.4 | lncRNA | UP | 1.000489 | -1.61614 | 7.548571 | 2.02E-13 | 5.03E-12 | 19.93116 |
| AKR1C2 | mRNA | DOWN | -1.71691 | 4.153177 | -4.956 | 9.79E-07 | 9.30E-06 | 4.678136 |
| ETNPPL | mRNA | DOWN | -1.66107 | -3.70672 | -5.67707 | 2.29E-08 | 2.90E-07 | 8.721351 |
| GPX2 | mRNA | DOWN | -1.58313 | 3.235257 | -4.73357 | 2.86E-06 | 2.48E-05 | 3.730656 |
| NR0B1 | mRNA | DOWN | -1.581 | -3.50756 | -4.4846 | 9.02E-06 | 7.12E-05 | 3.036211 |
| CPLX2 | mRNA | DOWN | -1.42869 | -2.06387 | -3.94869 | 8.95E-05 | 0.000573 | 0.838776 |
| FGB | mRNA | DOWN | -1.39009 | 0.960311 | -3.04131 | 0.002476 | 0.010866 | -2.42859 |
| FXYD4 | mRNA | DOWN | -1.36837 | -1.59629 | -5.2825 | 1.89E-07 | 2.03E-06 | 6.656448 |
| FGA | mRNA | DOWN | -1.36264 | 3.480836 | -3.70755 | 0.000232 | 0.001349 | -0.46044 |
| WDR72 | mRNA | DOWN | -1.34223 | 1.265172 | -4.90534 | 1.25E-06 | 1.17E-05 | 4.674833 |
| AKR1C1 | mRNA | DOWN | -1.34194 | 4.806683 | -4.72552 | 2.97E-06 | 2.57E-05 | 3.551359 |
| INSL4 | mRNA | DOWN | -1.34065 | -3.62353 | -3.98124 | 7.84E-05 | 0.000509 | 1.009219 |
| PAH | mRNA | DOWN | -1.32707 | -1.60518 | -4.5607 | 6.38E-06 | 5.19E-05 | 3.303573 |
| S100P | mRNA | DOWN | -1.32268 | 5.409965 | -4.59887 | 5.35E-06 | 4.41E-05 | 2.927108 |
| MAGEA1 | mRNA | DOWN | -1.2525 | -4.21511 | -4.05271 | 5.85E-05 | 0.000389 | 1.275406 |
| AKR1B10 | mRNA | DOWN | -1.23766 | 1.136387 | -3.27583 | 0.001125 | 0.005462 | -1.72206 |
| CYP4F11 | mRNA | DOWN | -1.23669 | 0.793391 | -4.00409 | 7.14E-05 | 0.000467 | 0.871825 |
| CKMT1B | mRNA | DOWN | -1.21398 | 0.348259 | -5.8924 | 6.89E-09 | 9.48E-08 | 9.739815 |
| FGL1 | mRNA | DOWN | -1.20089 | 1.658485 | -3.58589 | 0.000368 | 0.00204 | -0.7323 |
| INHA | mRNA | DOWN | -1.19724 | -0.23072 | -4.43758 | 1.11E-05 | 8.64E-05 | 2.699076 |
| HGD | mRNA | DOWN | -1.1753 | 2.573824 | -4.8935 | 1.33E-06 | 1.23E-05 | 4.517413 |
| NPW | mRNA | DOWN | -1.1383 | -0.51441 | -5.21855 | 2.62E-07 | 2.75E-06 | 6.289086 |
| CKMT1A | mRNA | DOWN | -1.12933 | 0.106425 | -5.50233 | 5.92E-08 | 6.98E-07 | 7.681068 |
| KLK12 | mRNA | DOWN | -1.08029 | -0.99682 | -2.94752 | 0.003349 | 0.014096 | -2.55529 |
| F2 | mRNA | DOWN | -1.06324 | -3.90276 | -4.3214 | 1.86E-05 | 0.000138 | 2.361153 |
| BARX1 | mRNA | DOWN | -1.05457 | -0.0234 | -2.9996 | 0.002834 | 0.012216 | -2.47342 |
| DDC | mRNA | DOWN | -1.05039 | 0.156778 | -4.08369 | 5.14E-05 | 0.000346 | 1.228538 |
| MAGEA3 | mRNA | DOWN | -1.04389 | -2.83988 | -2.5515 | 0.011015 | 0.03918 | -3.51765 |
| LCN12 | mRNA | DOWN | -1.04297 | 0.361299 | -7.57589 | 1.67E-13 | 4.19E-12 | 20.05858 |
| AKR7A3 | mRNA | DOWN | -1.03486 | 1.316089 | -4.8772 | 1.44E-06 | 1.32E-05 | 4.539192 |
| AKR1C4 | mRNA | DOWN | -1.02239 | -3.0481 | -3.99344 | 7.46E-05 | 0.000486 | 1.047422 |
| HMGCS2 | mRNA | DOWN | -1.01783 | -1.89521 | -3.42605 | 0.000661 | 0.003424 | -1.02399 |
| CABYR | mRNA | DOWN | -1.01024 | 2.218812 | -5.39568 | 1.04E-07 | 1.18E-06 | 6.989403 |
| CYP4F3 | mRNA | DOWN | -1.00738 | -0.04917 | -3.62295 | 0.00032 | 0.001799 | -0.46779 |
| EPYC | mRNA | UP | 2.265065 | -2.09891 | 10.01905 | 1.06E-21 | 5.96E-20 | 38.5123 |
| OMD | mRNA | UP | 2.143981 | 0.96933 | 16.03079 | 3.39E-47 | 2.17E-44 | 96.33147 |
| LRRC15 | mRNA | UP | 2.081964 | 3.63784 | 13.07444 | 6.31E-34 | 9.23E-32 | 66.26046 |
| CILP | mRNA | UP | 1.967328 | 3.051632 | 11.89237 | 5.58E-29 | 5.56E-27 | 55.00806 |
| FGF10 | mRNA | UP | 1.953429 | -2.72706 | 12.67231 | 3.24E-32 | 4.14E-30 | 62.14495 |
| OGN | mRNA | UP | 1.944523 | 1.210693 | 12.28071 | 1.41E-30 | 1.59E-28 | 58.63678 |
| F13A1 | mRNA | UP | 1.912227 | 5.014533 | 15.79507 | 4.22E-46 | 2.28E-43 | 94.02931 |
| COL11A1 | mRNA | UP | 1.90714 | 4.055165 | 7.721238 | 6.06E-14 | 1.60E-12 | 20.84361 |
| NETO1 | mRNA | UP | 1.897044 | -1.15408 | 9.167535 | 1.16E-18 | 5.04E-17 | 31.68468 |
| SFRP2 | mRNA | UP | 1.880578 | 6.065783 | 11.81391 | 1.16E-28 | 1.11E-26 | 54.15581 |
| KERA | mRNA | UP | 1.801736 | -3.16002 | 13.87987 | 1.99E-37 | 4.11E-35 | 73.84641 |
| GREM1 | mRNA | UP | 1.754783 | 4.682837 | 9.309416 | 3.72E-19 | 1.69E-17 | 32.60377 |
| DPT | mRNA | UP | 1.746254 | 3.861688 | 15.38989 | 3.13E-44 | 1.36E-41 | 89.75269 |
| SGCD | mRNA | UP | 1.702337 | 2.61035 | 19.7794 | 3.62E-65 | 1.76E-61 | 137.3817 |
| CPA3 | mRNA | UP | 1.69892 | 3.732055 | 12.04984 | 1.27E-29 | 1.33E-27 | 56.45905 |
| DCN | mRNA | UP | 1.695708 | 8.159798 | 22.18749 | 5.23E-77 | 1.02E-72 | 164.7912 |
| P2RY12 | mRNA | UP | 1.67814 | -0.21705 | 12.10716 | 7.35E-30 | 7.85E-28 | 56.96713 |
| MFAP5 | mRNA | UP | 1.676583 | 1.283432 | 9.334293 | 3.05E-19 | 1.39E-17 | 32.96261 |
| MRC1 | mRNA | UP | 1.667029 | 5.688846 | 13.33713 | 4.66E-35 | 7.46E-33 | 68.78205 |
| PDZRN4 | mRNA | UP | 1.666237 | -2.09994 | 11.55811 | 1.26E-27 | 1.12E-25 | 51.84397 |
| SFRP4 | mRNA | UP | 1.665929 | 5.250751 | 12.32391 | 9.32E-31 | 1.08E-28 | 58.98447 |
| COL6A5 | mRNA | UP | 1.658753 | 1.289625 | 10.57917 | 8.48E-24 | 5.70E-22 | 43.26931 |
| PPAPDC1A | mRNA | UP | 1.65699 | -0.05359 | 8.74589 | 3.16E-17 | 1.20E-15 | 28.44348 |
| FGF7 | mRNA | UP | 1.651806 | 2.989262 | 18.15125 | 2.93E-57 | 6.34E-54 | 119.4144 |
| COMP | mRNA | UP | 1.644212 | 4.151816 | 8.679038 | 5.29E-17 | 1.97E-15 | 27.75538 |
| GPR1 | mRNA | UP | 1.635172 | -1.43474 | 10.81757 | 1.04E-24 | 7.40E-23 | 45.30118 |
| VCAM1 | mRNA | UP | 1.630745 | 4.824817 | 18.46392 | 9.07E-59 | 2.41E-55 | 122.9726 |
| COL10A1 | mRNA | UP | 1.629984 | 5.037764 | 11.03958 | 1.42E-25 | 1.09E-23 | 47.17834 |
| GAS1 | mRNA | UP | 1.629198 | 2.454138 | 15.59378 | 3.60E-45 | 1.72E-42 | 91.84505 |
| WSCD2 | mRNA | UP | 1.628858 | -1.50665 | 12.75472 | 1.45E-32 | 1.91E-30 | 63.00448 |
| C7 | mRNA | UP | 1.625371 | 5.278268 | 9.236976 | 6.66E-19 | 2.96E-17 | 31.98708 |
| SYNDIG1 | mRNA | UP | 1.623173 | 0.880814 | 12.92071 | 2.86E-33 | 3.94E-31 | 64.73286 |
| CCL11 | mRNA | UP | 1.620596 | 0.194633 | 10.95777 | 2.96E-25 | 2.19E-23 | 46.56408 |
| SCN2B | mRNA | UP | 1.619063 | -0.4569 | 12.2212 | 2.49E-30 | 2.73E-28 | 58.02124 |
| ISM1 | mRNA | UP | 1.614466 | 2.480781 | 14.00015 | 5.85E-38 | 1.28E-35 | 75.43657 |
| MMP3 | mRNA | UP | 1.61412 | -0.68078 | 8.030731 | 6.66E-15 | 1.99E-13 | 23.23364 |
| SVEP1 | mRNA | UP | 1.612819 | 3.810346 | 15.73792 | 7.76E-46 | 4.05E-43 | 93.41403 |
| CCDC80 | mRNA | UP | 1.611233 | 6.59162 | 18.44219 | 1.16E-58 | 2.93E-55 | 122.7577 |
| CLSTN2 | mRNA | UP | 1.610369 | 1.148844 | 11.922 | 4.22E-29 | 4.24E-27 | 55.28661 |
| FIBIN | mRNA | UP | 1.609171 | 2.439496 | 16.16254 | 8.22E-48 | 5.85E-45 | 97.85668 |
| COL3A1 | mRNA | UP | 1.608445 | 10.45144 | 14.66629 | 6.17E-41 | 1.80E-38 | 81.87111 |
| FAM180A | mRNA | UP | 1.607986 | -0.41723 | 15.49926 | 9.83E-45 | 4.45E-42 | 90.59609 |
| ADAM12 | mRNA | UP | 1.604596 | 3.982539 | 11.93233 | 3.83E-29 | 3.89E-27 | 55.3552 |
| ADAMTS16 | mRNA | UP | 1.603852 | 1.842875 | 11.77636 | 1.65E-28 | 1.56E-26 | 53.94673 |
| PLN | mRNA | UP | 1.603681 | 2.24566 | 14.05422 | 3.37E-38 | 7.63E-36 | 75.97493 |
| WNT2 | mRNA | UP | 1.60084 | 2.608878 | 15.62783 | 2.51E-45 | 1.22E-42 | 92.21096 |
| COL14A1 | mRNA | UP | 1.600244 | 4.973572 | 14.30526 | 2.58E-39 | 6.57E-37 | 78.5296 |
| CXCL12 | mRNA | UP | 1.59924 | 4.749869 | 18.40315 | 1.78E-58 | 4.34E-55 | 122.2986 |
| MFAP4 | mRNA | UP | 1.596325 | 5.769737 | 13.80776 | 4.13E-37 | 8.19E-35 | 73.46849 |
| SPON1 | mRNA | UP | 1.594305 | 5.07357 | 17.38459 | 1.39E-53 | 1.84E-50 | 111.1287 |
| LUM | mRNA | UP | 1.588186 | 8.682173 | 20.56182 | 5.29E-69 | 4.41E-65 | 146.4118 |
| MS4A2 | mRNA | UP | 1.587532 | 1.286192 | 11.39914 | 5.43E-27 | 4.60E-25 | 50.50663 |
| THBS2 | mRNA | UP | 1.587278 | 7.052729 | 13.94135 | 1.06E-37 | 2.25E-35 | 74.74829 |
| ALPK2 | mRNA | UP | 1.583235 | -0.1463 | 11.35092 | 8.44E-27 | 7.07E-25 | 50.05116 |
| C1QTNF7 | mRNA | UP | 1.580037 | 0.924848 | 11.80384 | 1.28E-28 | 1.21E-26 | 54.19222 |
| RSPO1 | mRNA | UP | 1.579615 | -1.55132 | 11.17375 | 4.24E-26 | 3.33E-24 | 48.42513 |
| MMP13 | mRNA | UP | 1.572941 | 2.349059 | 6.20521 | 1.13E-09 | 1.75E-08 | 11.36535 |
| COL12A1 | mRNA | UP | 1.562709 | 6.121261 | 11.00783 | 1.89E-25 | 1.43E-23 | 46.8268 |
| FMO2 | mRNA | UP | 1.562551 | 4.342738 | 14.13573 | 1.47E-38 | 3.44E-36 | 76.81668 |
| MEOX2 | mRNA | UP | 1.561476 | 1.715465 | 16.15968 | 8.48E-48 | 5.96E-45 | 97.77238 |
| ITGBL1 | mRNA | UP | 1.559004 | 3.938276 | 15.66856 | 1.63E-45 | 8.11E-43 | 92.68451 |
| FNDC1 | mRNA | UP | 1.555418 | 4.325987 | 11.18436 | 3.85E-26 | 3.04E-24 | 48.50741 |
| CTSG | mRNA | UP | 1.553925 | -0.75503 | 9.053779 | 2.86E-18 | 1.20E-16 | 30.80156 |
| CCL19 | mRNA | UP | 1.553917 | 3.528467 | 9.594355 | 3.66E-20 | 1.81E-18 | 34.95718 |
| MMP2 | mRNA | UP | 1.550391 | 8.031327 | 19.29403 | 8.47E-63 | 3.30E-59 | 132.2027 |
| ASPN | mRNA | UP | 1.547574 | 4.31449 | 13.53437 | 6.49E-36 | 1.12E-33 | 70.78036 |
| NTRK3 | mRNA | UP | 1.544571 | -0.06532 | 13.38847 | 2.79E-35 | 4.57E-33 | 69.23784 |
| GPR88 | mRNA | UP | 1.539122 | -1.50577 | 12.5706 | 8.67E-32 | 1.06E-29 | 61.25287 |
| BICC1 | mRNA | UP | 1.539017 | 3.701075 | 17.37744 | 1.50E-53 | 1.95E-50 | 111.0052 |
| SLIT3 | mRNA | UP | 1.536778 | 4.255769 | 13.66037 | 1.83E-36 | 3.39E-34 | 72.0362 |
| CCL13 | mRNA | UP | 1.533766 | 2.774279 | 10.03804 | 9.01E-22 | 5.11E-20 | 38.64086 |
| WT1 | mRNA | UP | 1.532598 | -1.54553 | 7.383209 | 6.27E-13 | 1.47E-11 | 18.82924 |
| MXRA5 | mRNA | UP | 1.53164 | 6.421348 | 14.3115 | 2.42E-39 | 6.24E-37 | 78.54268 |
| PRND | mRNA | UP | 1.531455 | -1.31506 | 8.251234 | 1.33E-15 | 4.26E-14 | 24.81617 |
| FBN1 | mRNA | UP | 1.525255 | 6.442516 | 18.07586 | 6.76E-57 | 1.41E-53 | 118.7124 |
| SCN7A | mRNA | UP | 1.523119 | 3.147817 | 10.94992 | 3.18E-25 | 2.35E-23 | 46.46631 |
| APELA | mRNA | UP | 1.522636 | -1.99619 | 7.757547 | 4.69E-14 | 1.26E-12 | 21.35417 |
| CBLN4 | mRNA | UP | 1.520308 | -1.46768 | 10.18374 | 2.60E-22 | 1.55E-20 | 39.89276 |
| WNT7A | mRNA | UP | 1.519061 | -0.66088 | 6.356103 | 4.57E-10 | 7.54E-09 | 12.41314 |
| TLR8 | mRNA | UP | 1.512963 | 2.275718 | 13.62272 | 2.67E-36 | 4.80E-34 | 71.65679 |
| RANBP3L | mRNA | UP | 1.511 | -0.80853 | 13.75685 | 6.90E-37 | 1.33E-34 | 72.81589 |
| MS4A1 | mRNA | UP | 1.51092 | 2.322726 | 7.801617 | 3.44E-14 | 9.37E-13 | 21.50943 |
| CCR6 | mRNA | UP | 1.50514 | -2.13517 | 11.6874 | 3.79E-28 | 3.46E-26 | 53.01411 |
| SEMA3D | mRNA | UP | 1.504563 | 1.058233 | 11.99088 | 2.21E-29 | 2.30E-27 | 55.924 |
| IL22RA2 | mRNA | UP | 1.498797 | -0.64435 | 8.584007 | 1.09E-16 | 3.96E-15 | 27.2416 |
| LRRN4CL | mRNA | UP | 1.498078 | -0.25035 | 15.28906 | 9.08E-44 | 3.63E-41 | 88.43085 |
| CCR2 | mRNA | UP | 1.495819 | 2.4672 | 15.35188 | 4.68E-44 | 1.97E-41 | 89.31154 |
| COLEC12 | mRNA | UP | 1.495608 | 4.839738 | 14.14692 | 1.31E-38 | 3.11E-36 | 76.92181 |
| SIGLEC6 | mRNA | UP | 1.49365 | -0.80469 | 11.02522 | 1.62E-25 | 1.23E-23 | 47.13663 |
| COL6A3 | mRNA | UP | 1.490011 | 9.061512 | 17.74074 | 2.75E-55 | 4.35E-52 | 114.9163 |
| DIO2 | mRNA | UP | 1.487341 | 2.867095 | 10.57909 | 8.49E-24 | 5.70E-22 | 43.23469 |
| IL7R | mRNA | UP | 1.484477 | 5.253268 | 15.50008 | 9.74E-45 | 4.44E-42 | 90.9118 |
| IVL | mRNA | UP | 1.481872 | -0.41471 | 4.458842 | 1.01E-05 | 7.93E-05 | 2.801146 |
| CLEC6A | mRNA | UP | 1.476151 | -2.43066 | 9.681858 | 1.78E-20 | 9.04E-19 | 35.75148 |
| COL8A2 | mRNA | UP | 1.474545 | 4.299294 | 14.3269 | 2.06E-39 | 5.40E-37 | 78.7603 |
| CYBB | mRNA | UP | 1.46959 | 6.287012 | 15.09957 | 6.68E-43 | 2.38E-40 | 86.68689 |
| CDK15 | mRNA | UP | 1.469529 | -1.03614 | 14.38322 | 1.15E-39 | 3.08E-37 | 79.06758 |
| KLHL4 | mRNA | UP | 1.467301 | -0.1136 | 13.58515 | 3.90E-36 | 6.89E-34 | 71.17084 |
| BNC2 | mRNA | UP | 1.466169 | 2.540058 | 19.3101 | 7.07E-63 | 2.95E-59 | 132.1599 |
| GCSAML | mRNA | UP | 1.465292 | -1.23857 | 10.2527 | 1.44E-22 | 8.79E-21 | 40.47523 |
| FAP | mRNA | UP | 1.463804 | 3.964985 | 14.69377 | 4.64E-41 | 1.37E-38 | 82.52038 |
| FOLR2 | mRNA | UP | 1.46286 | 3.848359 | 13.62825 | 2.53E-36 | 4.61E-34 | 71.72108 |
| TLR7 | mRNA | UP | 1.459975 | 2.289401 | 15.41271 | 2.46E-44 | 1.09E-41 | 89.93857 |
| SERPINA9 | mRNA | UP | 1.454806 | -3.77461 | 8.308891 | 8.65E-16 | 2.84E-14 | 25.23904 |
| MSR1 | mRNA | UP | 1.453774 | 5.368381 | 14.18623 | 8.74E-39 | 2.13E-36 | 77.30735 |
| ITGA11 | mRNA | UP | 1.452423 | 4.670526 | 11.22373 | 2.69E-26 | 2.16E-24 | 48.84404 |
| FDCSP | mRNA | UP | 1.452073 | 0.239663 | 6.150512 | 1.55E-09 | 2.37E-08 | 11.18614 |
| SLC1A7 | mRNA | UP | 1.448605 | 2.195015 | 5.862512 | 8.16E-09 | 1.11E-07 | 9.454856 |
| GPR34 | mRNA | UP | 1.448514 | 2.537562 | 15.27009 | 1.11E-43 | 4.38E-41 | 88.46113 |
| RP11-812E19.9 | mRNA | UP | 1.4482 | -1.60224 | 8.014526 | 7.48E-15 | 2.22E-13 | 23.13458 |
| MARCO | mRNA | UP | 1.447745 | 4.733901 | 8.589991 | 1.04E-16 | 3.80E-15 | 27.04471 |
| ISLR | mRNA | UP | 1.447296 | 5.807443 | 16.24375 | 3.43E-48 | 2.57E-45 | 98.79894 |
| APCDD1L | mRNA | UP | 1.445987 | -0.5066 | 7.028072 | 6.69E-12 | 1.39E-10 | 16.50156 |
| PDGFRA | mRNA | UP | 1.44587 | 4.99937 | 19.21325 | 2.10E-62 | 7.65E-59 | 131.291 |
| TIMP3 | mRNA | UP | 1.445479 | 2.923061 | 13.4263 | 1.91E-35 | 3.19E-33 | 69.71898 |
| GAS7 | mRNA | UP | 1.445354 | 4.74032 | 21.36343 | 6.03E-73 | 7.05E-69 | 155.3605 |
| COL6A6 | mRNA | UP | 1.444667 | 1.836501 | 8.893393 | 1.01E-17 | 4.01E-16 | 29.50792 |
| NALCN | mRNA | UP | 1.443947 | 1.235095 | 14.21416 | 6.56E-39 | 1.62E-36 | 77.54612 |
| CTSK | mRNA | UP | 1.443276 | 5.767949 | 16.51427 | 1.85E-49 | 1.66E-46 | 101.702 |
| FCRLA | mRNA | UP | 1.443241 | 1.118011 | 9.210733 | 8.22E-19 | 3.64E-17 | 31.99263 |
| SHISA3 | mRNA | UP | 1.441984 | 1.247205 | 5.896592 | 6.73E-09 | 9.27E-08 | 9.707164 |
| CXCL14 | mRNA | UP | 1.438641 | 4.805184 | 5.9646 | 4.57E-09 | 6.47E-08 | 9.807478 |
| TMEM119 | mRNA | UP | 1.437022 | 4.562542 | 17.5343 | 2.68E-54 | 3.64E-51 | 112.7506 |
| PTGIS | mRNA | UP | 1.433349 | 4.117819 | 13.89913 | 1.63E-37 | 3.41E-35 | 74.4301 |
| COL1A2 | mRNA | UP | 1.431182 | 10.33699 | 13.9928 | 6.30E-38 | 1.37E-35 | 74.98344 |
| PDPN | mRNA | UP | 1.43113 | 4.356067 | 14.45614 | 5.44E-40 | 1.48E-37 | 80.07996 |
| HPGDS | mRNA | UP | 1.428516 | 1.381306 | 11.00267 | 1.98E-25 | 1.50E-23 | 46.96536 |
| SCGB1A1 | mRNA | UP | 1.427465 | 3.155609 | 3.863819 | 0.000126 | 0.000778 | 0.142509 |
| CD1E | mRNA | UP | 1.42435 | 0.836368 | 8.355796 | 6.10E-16 | 2.03E-14 | 25.52455 |
| FAM196B | mRNA | UP | 1.424295 | -2.7732 | 10.79823 | 1.23E-24 | 8.76E-23 | 45.09126 |
| CHIT1 | mRNA | UP | 1.42365 | 4.636324 | 7.049464 | 5.81E-12 | 1.21E-10 | 16.32472 |
| VSIG4 | mRNA | UP | 1.423408 | 5.017339 | 12.60383 | 6.29E-32 | 7.79E-30 | 61.66534 |
| TPSAB1 | mRNA | UP | 1.421842 | 3.160662 | 10.70519 | 2.80E-24 | 1.96E-22 | 44.3187 |
| ADH1B | mRNA | UP | 1.421487 | 3.783381 | 5.911454 | 6.18E-09 | 8.58E-08 | 9.600696 |
| FPR1 | mRNA | UP | 1.419175 | 3.249288 | 13.9986 | 5.94E-38 | 1.30E-35 | 75.43309 |
| CPED1 | mRNA | UP | 1.417888 | 2.90059 | 17.10767 | 2.89E-52 | 3.13E-49 | 108.0301 |
| PLXDC2 | mRNA | UP | 1.417494 | 6.070436 | 23.6597 | 2.88E-84 | 1.68E-79 | 181.3595 |
| COL1A1 | mRNA | UP | 1.416794 | 10.87392 | 11.25354 | 2.05E-26 | 1.66E-24 | 48.55658 |
| SULF1 | mRNA | UP | 1.416133 | 6.756387 | 11.69103 | 3.66E-28 | 3.36E-26 | 52.96949 |
| OLFML1 | mRNA | UP | 1.415609 | 2.926852 | 22.73087 | 1.09E-79 | 3.19E-75 | 170.4839 |
| PRELP | mRNA | UP | 1.415272 | 5.794357 | 13.90729 | 1.50E-37 | 3.16E-35 | 74.46854 |
| ABCA6 | mRNA | UP | 1.414671 | 2.120277 | 17.21282 | 9.15E-53 | 1.03E-49 | 109.104 |
| ECM2 | mRNA | UP | 1.41397 | 3.114197 | 19.84047 | 1.82E-65 | 1.06E-61 | 138.1283 |
| MRGPRF | mRNA | UP | 1.413232 | 2.161401 | 16.72062 | 1.97E-50 | 1.88E-47 | 103.8011 |
| SLCO2B1 | mRNA | UP | 1.412799 | 6.123005 | 16.53617 | 1.46E-49 | 1.33E-46 | 101.9321 |
| FMO1 | mRNA | UP | 1.412613 | 1.042949 | 12.54593 | 1.10E-31 | 1.34E-29 | 61.14423 |
| PIK3CG | mRNA | UP | 1.411395 | 2.642917 | 15.80652 | 3.73E-46 | 2.04E-43 | 94.09641 |
| CLMP | mRNA | UP | 1.410953 | 3.697232 | 13.86263 | 2.37E-37 | 4.80E-35 | 74.06627 |
| ADAMTS12 | mRNA | UP | 1.407442 | 4.242748 | 13.90322 | 1.57E-37 | 3.28E-35 | 74.46978 |
| CD1A | mRNA | UP | 1.406565 | 1.427164 | 6.436316 | 2.81E-10 | 4.77E-09 | 12.7739 |
| WNT10A | mRNA | UP | 1.40292 | 1.529325 | 9.262026 | 5.45E-19 | 2.44E-17 | 32.38365 |
| PRRX1 | mRNA | UP | 1.399854 | 5.04099 | 17.55459 | 2.14E-54 | 2.98E-51 | 112.9841 |
| GLT8D2 | mRNA | UP | 1.39947 | 2.761011 | 17.7092 | 3.90E-55 | 5.99E-52 | 114.5572 |
| CORIN | mRNA | UP | 1.397245 | 1.154894 | 13.10018 | 4.89E-34 | 7.25E-32 | 66.48404 |
| INHBA | mRNA | UP | 1.397134 | 5.026097 | 13.00384 | 1.26E-33 | 1.79E-31 | 65.53584 |
| BHLHE22 | mRNA | UP | 1.39619 | 0.902466 | 16.44549 | 3.89E-49 | 3.24E-46 | 100.7311 |
| FCER1A | mRNA | UP | 1.395242 | 1.122552 | 7.246632 | 1.58E-12 | 3.52E-11 | 17.8366 |
| TEX26 | mRNA | UP | 1.395119 | -3.37404 | 7.259824 | 1.44E-12 | 3.24E-11 | 18.04529 |
| TENM3 | mRNA | UP | 1.394225 | 1.956818 | 11.24439 | 2.23E-26 | 1.79E-24 | 49.11223 |
| COL15A1 | mRNA | UP | 1.393205 | 5.407218 | 13.45079 | 1.50E-35 | 2.52E-33 | 69.91861 |
| CILP2 | mRNA | UP | 1.39279 | 2.775069 | 9.736798 | 1.13E-20 | 5.81E-19 | 36.15116 |
| PLA2G2D | mRNA | UP | 1.39249 | 2.105743 | 7.669646 | 8.70E-14 | 2.25E-12 | 20.61201 |
| CCR4 | mRNA | UP | 1.392271 | 1.676388 | 11.87886 | 6.34E-29 | 6.28E-27 | 54.89246 |
| CD209 | mRNA | UP | 1.392073 | 2.691917 | 12.98071 | 1.59E-33 | 2.22E-31 | 65.35168 |
| ZCCHC5 | mRNA | UP | 1.391812 | -4.17904 | 11.85602 | 7.85E-29 | 7.69E-27 | 54.5081 |
| CLEC4G | mRNA | UP | 1.390804 | -2.04957 | 9.534839 | 5.96E-20 | 2.90E-18 | 34.57617 |
| MNDA | mRNA | UP | 1.38982 | 3.763549 | 14.97847 | 2.38E-42 | 8.17E-40 | 85.46162 |
| COL5A1 | mRNA | UP | 1.389596 | 7.829498 | 13.78603 | 5.14E-37 | 1.01E-34 | 73.12861 |
| HHLA2 | mRNA | UP | 1.389079 | 1.346433 | 4.546184 | 6.82E-06 | 5.51E-05 | 3.052073 |
| MMP7 | mRNA | UP | 1.388981 | 5.22698 | 7.306172 | 1.06E-12 | 2.41E-11 | 17.94566 |
| AMPD1 | mRNA | UP | 1.387931 | -0.77493 | 9.572765 | 4.37E-20 | 2.15E-18 | 34.89036 |
| CDH11 | mRNA | UP | 1.38762 | 6.36794 | 16.39906 | 6.42E-49 | 5.21E-46 | 100.4521 |
| MOXD1 | mRNA | UP | 1.387442 | 4.994873 | 15.7379 | 7.77E-46 | 4.05E-43 | 93.42397 |
| PLA2G5 | mRNA | UP | 1.385099 | 0.714497 | 18.82332 | 1.65E-60 | 5.06E-57 | 126.4937 |
| MYLK | mRNA | UP | 1.383948 | 6.315399 | 20.612 | 3.00E-69 | 2.92E-65 | 146.995 |
| TCF21 | mRNA | UP | 1.382033 | 1.815383 | 10.91817 | 4.22E-25 | 3.09E-23 | 46.21619 |
| CD1B | mRNA | UP | 1.380807 | -0.94864 | 8.01847 | 7.27E-15 | 2.16E-13 | 23.15251 |
| NAV3 | mRNA | UP | 1.380201 | 1.42749 | 14.02714 | 4.44E-38 | 9.86E-36 | 75.67086 |
| PTPRC | mRNA | UP | 1.379767 | 5.924206 | 15.84383 | 2.51E-46 | 1.39E-43 | 94.53361 |
| MYH11 | mRNA | UP | 1.379162 | 5.862953 | 10.98027 | 2.42E-25 | 1.81E-23 | 46.59889 |
| SAA2-SAA4 | mRNA | UP | 1.376758 | -2.26038 | 5.913139 | 6.12E-09 | 8.50E-08 | 9.966178 |
| PTPRO | mRNA | UP | 1.376441 | 2.302235 | 14.8961 | 5.63E-42 | 1.79E-39 | 84.56951 |
| HTR2A | mRNA | UP | 1.375773 | -1.55277 | 12.21556 | 2.62E-30 | 2.87E-28 | 57.91071 |
| CLEC4C | mRNA | UP | 1.375727 | -3.36498 | 8.938499 | 7.09E-18 | 2.85E-16 | 29.90951 |
| CSMD2 | mRNA | UP | 1.375723 | 0.970484 | 11.94802 | 3.31E-29 | 3.38E-27 | 55.52487 |
| FPR3 | mRNA | UP | 1.375335 | 4.709268 | 16.06688 | 2.30E-47 | 1.54E-44 | 96.91624 |
| TLR4 | mRNA | UP | 1.375241 | 3.836471 | 17.36854 | 1.66E-53 | 2.10E-50 | 110.9155 |
| MSRB3 | mRNA | UP | 1.375184 | 4.177094 | 20.07858 | 1.24E-66 | 8.06E-63 | 140.8946 |
| POSTN | mRNA | UP | 1.374061 | 7.479072 | 12.57989 | 7.93E-32 | 9.74E-30 | 61.28736 |
| VGLL3 | mRNA | UP | 1.37344 | 3.865688 | 14.79533 | 1.61E-41 | 4.93E-39 | 83.56722 |
| GLB1L3 | mRNA | UP | 1.371992 | 1.318329 | 4.442653 | 1.09E-05 | 8.46E-05 | 2.609505 |
| COL5A2 | mRNA | UP | 1.371804 | 7.134616 | 13.49523 | 9.61E-36 | 1.65E-33 | 70.26855 |
| ITGA8 | mRNA | UP | 1.37002 | 3.516455 | 14.16402 | 1.10E-38 | 2.65E-36 | 77.10647 |
| CD163 | mRNA | UP | 1.369192 | 6.036404 | 12.60118 | 6.45E-32 | 7.98E-30 | 61.58962 |
| PDCD1LG2 | mRNA | UP | 1.369126 | 2.069928 | 14.30817 | 2.50E-39 | 6.43E-37 | 78.53849 |
| SPOCK1 | mRNA | UP | 1.36652 | 2.40582 | 7.513186 | 2.58E-13 | 6.35E-12 | 19.5323 |
| C3AR1 | mRNA | UP | 1.366225 | 4.142783 | 15.94938 | 8.10E-47 | 4.88E-44 | 95.66025 |
| FPR2 | mRNA | UP | 1.365813 | 0.658788 | 10.35 | 6.24E-23 | 3.90E-21 | 41.31295 |
| SLC24A2 | mRNA | UP | 1.364938 | -1.00628 | 7.797234 | 3.54E-14 | 9.66E-13 | 21.61125 |
| WISP1 | mRNA | UP | 1.363611 | 3.211194 | 14.1055 | 2.00E-38 | 4.64E-36 | 76.51187 |
| TMEM200A | mRNA | UP | 1.362261 | 2.895082 | 17.84807 | 8.42E-56 | 1.45E-52 | 116.0858 |
| RGS13 | mRNA | UP | 1.359627 | -0.47438 | 11.39821 | 5.48E-27 | 4.63E-25 | 50.46672 |
| TCL1A | mRNA | UP | 1.35801 | -0.83999 | 7.297702 | 1.12E-12 | 2.54E-11 | 18.25032 |
| ALOX5AP | mRNA | UP | 1.358002 | 4.870301 | 14.00701 | 5.45E-38 | 1.21E-35 | 75.50456 |
| CLEC5A | mRNA | UP | 1.35774 | 2.86035 | 11.66774 | 4.55E-28 | 4.13E-26 | 52.93861 |
| SSPN | mRNA | UP | 1.355595 | 3.672626 | 17.09137 | 3.46E-52 | 3.67E-49 | 107.8985 |
| ZNF521 | mRNA | UP | 1.354238 | 2.732096 | 17.28941 | 3.95E-53 | 4.52E-50 | 109.9871 |
| SMOC2 | mRNA | UP | 1.354115 | 4.113764 | 12.20191 | 2.99E-30 | 3.26E-28 | 57.87503 |
| HLA-DQA1 | mRNA | UP | 1.35381 | 7.560981 | 12.28433 | 1.36E-30 | 1.54E-28 | 58.45819 |
| VCAN | mRNA | UP | 1.353283 | 7.341465 | 12.34548 | 7.58E-31 | 8.87E-29 | 59.05669 |
| GZMK | mRNA | UP | 1.350493 | 1.770558 | 10.88645 | 5.61E-25 | 4.08E-23 | 45.93788 |
| AOC3 | mRNA | UP | 1.349801 | 5.229148 | 15.09549 | 6.97E-43 | 2.47E-40 | 86.67357 |
| LILRA4 | mRNA | UP | 1.349718 | -0.601 | 10.60372 | 6.84E-24 | 4.64E-22 | 43.47184 |
| DACT1 | mRNA | UP | 1.349698 | 2.857061 | 14.97227 | 2.54E-42 | 8.62E-40 | 85.37812 |
| GFRA1 | mRNA | UP | 1.349111 | 1.09425 | 7.960776 | 1.10E-14 | 3.20E-13 | 22.68281 |
| CR1 | mRNA | UP | 1.348311 | 2.089709 | 12.28532 | 1.35E-30 | 1.53E-28 | 58.69093 |
| CD84 | mRNA | UP | 1.34633 | 4.355668 | 15.28793 | 9.19E-44 | 3.65E-41 | 88.68988 |
| AEBP1 | mRNA | UP | 1.345786 | 8.126533 | 15.31947 | 6.59E-44 | 2.69E-41 | 88.8904 |
| GALNT15 | mRNA | UP | 1.344737 | 1.437863 | 11.25539 | 2.02E-26 | 1.63E-24 | 49.21402 |
| MCEMP1 | mRNA | UP | 1.34457 | 1.945023 | 7.782305 | 3.94E-14 | 1.07E-12 | 21.39588 |
| LSAMP | mRNA | UP | 1.343792 | 3.113976 | 15.38444 | 3.32E-44 | 1.43E-41 | 89.6783 |
| TIFAB | mRNA | UP | 1.341895 | -0.48236 | 10.19505 | 2.36E-22 | 1.41E-20 | 40.00197 |
| CLEC10A | mRNA | UP | 1.34184 | 2.527272 | 11.76308 | 1.87E-28 | 1.76E-26 | 53.8201 |
| RTN1 | mRNA | UP | 1.341793 | 2.267019 | 13.28029 | 8.21E-35 | 1.30E-32 | 68.27394 |
| FN1 | mRNA | UP | 1.340237 | 11.08529 | 13.79625 | 4.64E-37 | 9.18E-35 | 72.90865 |
| UNC5C | mRNA | UP | 1.340028 | 2.452917 | 18.05612 | 8.41E-57 | 1.64E-53 | 118.3219 |
| LPAR4 | mRNA | UP | 1.33645 | -2.76196 | 11.19682 | 3.44E-26 | 2.72E-24 | 48.58358 |
| COL8A1 | mRNA | UP | 1.336316 | 6.685121 | 14.09987 | 2.11E-38 | 4.90E-36 | 76.37372 |
| CXCL13 | mRNA | UP | 1.336017 | 3.889416 | 7.387272 | 6.10E-13 | 1.44E-11 | 18.5899 |
| CSF1R | mRNA | UP | 1.335874 | 6.017411 | 16.49703 | 2.22E-49 | 1.97E-46 | 101.5128 |
| CLC | mRNA | UP | 1.335519 | -3.72873 | 6.587572 | 1.11E-10 | 1.99E-09 | 13.85202 |
| CXCL6 | mRNA | UP | 1.333639 | 0.25264 | 5.815015 | 1.07E-08 | 1.42E-07 | 9.324402 |
| TSHZ3 | mRNA | UP | 1.331262 | 2.591086 | 18.99976 | 2.29E-61 | 7.87E-58 | 128.7289 |
| FCRL5 | mRNA | UP | 1.329981 | 2.925236 | 8.699735 | 4.51E-17 | 1.69E-15 | 27.98399 |
| ACTG2 | mRNA | UP | 1.329215 | 3.401176 | 11.45589 | 3.22E-27 | 2.80E-25 | 50.99172 |
| FAM101A | mRNA | UP | 1.328615 | 1.338853 | 8.382998 | 4.98E-16 | 1.68E-14 | 25.70402 |
| LYZ | mRNA | UP | 1.328397 | 8.107004 | 9.986322 | 1.39E-21 | 7.77E-20 | 37.8358 |
| FGL2 | mRNA | UP | 1.326823 | 4.968058 | 14.07485 | 2.73E-38 | 6.25E-36 | 76.18824 |
| DRD5 | mRNA | UP | 1.326799 | -3.46998 | 8.607708 | 9.13E-17 | 3.33E-15 | 27.4238 |
| MMP11 | mRNA | UP | 1.325848 | 5.251452 | 7.686021 | 7.76E-14 | 2.02E-12 | 20.50453 |
| P2RY13 | mRNA | UP | 1.325719 | 2.293813 | 13.24103 | 1.21E-34 | 1.88E-32 | 67.88909 |
| IGJ | mRNA | UP | 1.324891 | 8.257303 | 8.638768 | 7.20E-17 | 2.65E-15 | 27.09153 |
| KRT14 | mRNA | UP | 1.324823 | -1.30784 | 5.011219 | 7.45E-07 | 7.25E-06 | 5.330725 |
| XPNPEP2 | mRNA | UP | 1.324736 | -0.68339 | 11.85107 | 8.22E-29 | 8.03E-27 | 54.57764 |
| PGR | mRNA | UP | 1.324597 | 0.76748 | 13.12968 | 3.65E-34 | 5.49E-32 | 66.75552 |
| IL5RA | mRNA | UP | 1.322918 | -0.1545 | 8.618996 | 8.38E-17 | 3.07E-15 | 27.4938 |
| NUDT10 | mRNA | UP | 1.32283 | -2.52144 | 9.58787 | 3.86E-20 | 1.90E-18 | 34.99409 |
| TMEM130 | mRNA | UP | 1.321544 | 2.759254 | 7.931803 | 1.36E-14 | 3.89E-13 | 22.3928 |
| ITGAM | mRNA | UP | 1.319447 | 4.568202 | 14.02853 | 4.38E-38 | 9.78E-36 | 75.72822 |
| MS4A6A | mRNA | UP | 1.31901 | 5.372063 | 15.31168 | 7.15E-44 | 2.90E-41 | 88.93086 |
| FCER2 | mRNA | UP | 1.318978 | -1.05415 | 8.059889 | 5.39E-15 | 1.62E-13 | 23.44685 |
| PLA2G2A | mRNA | UP | 1.318871 | 0.670189 | 6.770363 | 3.52E-11 | 6.70E-10 | 14.8362 |
| SIGLEC14 | mRNA | UP | 1.318489 | 1.557858 | 7.813909 | 3.15E-14 | 8.64E-13 | 21.63495 |
| HTRA3 | mRNA | UP | 1.318409 | 5.549553 | 12.31982 | 9.69E-31 | 1.12E-28 | 58.92971 |
| CCBE1 | mRNA | UP | 1.317275 | 1.601919 | 8.543564 | 1.49E-16 | 5.30E-15 | 26.87757 |
| CD28 | mRNA | UP | 1.316888 | 2.044185 | 15.34796 | 4.88E-44 | 2.03E-41 | 89.24864 |
| IGSF21 | mRNA | UP | 1.316667 | 0.261669 | 10.20826 | 2.11E-22 | 1.27E-20 | 40.11641 |
| TFEC | mRNA | UP | 1.315143 | 2.947564 | 14.35782 | 1.50E-39 | 3.96E-37 | 79.06867 |
| EVI2A | mRNA | UP | 1.31475 | 3.389148 | 16.76951 | 1.16E-50 | 1.13E-47 | 104.4082 |
| GPR174 | mRNA | UP | 1.314621 | -0.51968 | 10.72581 | 2.33E-24 | 1.64E-22 | 44.52731 |
| LPPR4 | mRNA | UP | 1.313875 | 1.20611 | 13.19057 | 2.00E-34 | 3.05E-32 | 67.36739 |
| PDZRN3 | mRNA | UP | 1.313546 | 2.956599 | 17.34658 | 2.11E-53 | 2.57E-50 | 110.6247 |
| ANTXR1 | mRNA | UP | 1.312469 | 6.931189 | 15.71668 | 9.74E-46 | 5.03E-43 | 93.15072 |
| DES | mRNA | UP | 1.311958 | 2.353672 | 8.136161 | 3.09E-15 | 9.59E-14 | 23.86692 |
| FBLN1 | mRNA | UP | 1.311928 | 6.909893 | 15.39979 | 2.82E-44 | 1.24E-41 | 89.80577 |
| IL21R | mRNA | UP | 1.311151 | 2.526557 | 14.30535 | 2.57E-39 | 6.57E-37 | 78.52502 |
| FCRL3 | mRNA | UP | 1.311084 | 1.104777 | 9.638744 | 2.54E-20 | 1.27E-18 | 35.40559 |
| ST6GALNAC5 | mRNA | UP | 1.310676 | 2.250014 | 12.34115 | 7.90E-31 | 9.21E-29 | 59.21852 |
| CPZ | mRNA | UP | 1.310574 | -0.40345 | 13.06465 | 6.95E-34 | 1.01E-31 | 66.05864 |
| FCGR3A | mRNA | UP | 1.309194 | 6.24516 | 13.44548 | 1.58E-35 | 2.65E-33 | 69.82704 |
| S100B | mRNA | UP | 1.308759 | 2.382948 | 8.875274 | 1.16E-17 | 4.57E-16 | 29.34521 |
| ZNF385D | mRNA | UP | 1.307682 | -0.11566 | 13.39503 | 2.62E-35 | 4.29E-33 | 69.29912 |
| EPHA3 | mRNA | UP | 1.307626 | 1.873534 | 9.757735 | 9.48E-21 | 4.92E-19 | 36.35427 |
| DIO3 | mRNA | UP | 1.306571 | -1.76369 | 7.909586 | 1.59E-14 | 4.53E-13 | 22.40199 |
| CTD-2370N5.3 | mRNA | UP | 1.30581 | -3.75069 | 13.45562 | 1.43E-35 | 2.41E-33 | 69.64236 |
| SLC1A3 | mRNA | UP | 1.304825 | 3.43522 | 14.07189 | 2.81E-38 | 6.42E-36 | 76.17386 |
| CYTL1 | mRNA | UP | 1.304451 | -0.38888 | 11.33287 | 9.96E-27 | 8.26E-25 | 49.88267 |
| ABI3BP | mRNA | UP | 1.304451 | 4.149407 | 11.73556 | 2.42E-28 | 2.25E-26 | 53.52612 |
| CD79A | mRNA | UP | 1.303238 | 4.087478 | 8.846629 | 1.45E-17 | 5.67E-16 | 29.03179 |
| CD180 | mRNA | UP | 1.303213 | 2.928176 | 15.68976 | 1.30E-45 | 6.64E-43 | 92.87834 |
| IL2RA | mRNA | UP | 1.303005 | 2.812887 | 12.23562 | 2.17E-30 | 2.40E-28 | 58.21993 |
| TWIST1 | mRNA | UP | 1.302493 | 0.276129 | 7.722353 | 6.01E-14 | 1.59E-12 | 21.05934 |
| TLR10 | mRNA | UP | 1.302087 | 1.250911 | 10.10181 | 5.24E-22 | 3.02E-20 | 39.21522 |
| FBLN2 | mRNA | UP | 1.301952 | 5.695793 | 14.27817 | 3.40E-39 | 8.56E-37 | 78.23222 |
| GXYLT2 | mRNA | UP | 1.300934 | 2.847602 | 15.17952 | 2.88E-43 | 1.05E-40 | 87.53005 |
| UBE2QL1 | mRNA | UP | 1.300908 | -0.48003 | 9.079349 | 2.34E-18 | 9.85E-17 | 30.9973 |
| GPR84 | mRNA | UP | 1.299468 | 1.091919 | 11.43498 | 3.91E-27 | 3.35E-25 | 50.82947 |
| TNFAIP6 | mRNA | UP | 1.299373 | 1.957685 | 12.39577 | 4.68E-31 | 5.53E-29 | 59.73435 |
| FCRL1 | mRNA | UP | 1.299314 | -0.39679 | 7.796106 | 3.57E-14 | 9.73E-13 | 21.58868 |
| PRKG1 | mRNA | UP | 1.297416 | 3.050298 | 19.3918 | 2.83E-63 | 1.27E-59 | 133.1286 |
| RGS18 | mRNA | UP | 1.297144 | 1.053448 | 14.09495 | 2.22E-38 | 5.13E-36 | 76.33326 |
| CCIN | mRNA | UP | 1.296365 | -2.70285 | 12.20177 | 2.99E-30 | 3.26E-28 | 57.71778 |
| LAX1 | mRNA | UP | 1.295762 | 1.974454 | 10.97387 | 2.57E-25 | 1.91E-23 | 46.70503 |
| GPC6 | mRNA | UP | 1.29519 | 3.46978 | 9.661138 | 2.11E-20 | 1.07E-18 | 35.50187 |
| OLR1 | mRNA | UP | 1.294682 | 4.419551 | 10.84949 | 7.79E-25 | 5.60E-23 | 45.52822 |
| IGFL2 | mRNA | UP | 1.293595 | -0.01673 | 6.961405 | 1.03E-11 | 2.10E-10 | 16.06041 |
| MS4A4A | mRNA | UP | 1.292985 | 4.099357 | 14.26287 | 3.98E-39 | 9.98E-37 | 78.10966 |
| DDR2 | mRNA | UP | 1.292396 | 4.515255 | 17.6567 | 6.96E-55 | 1.04E-51 | 114.0869 |
| CHRDL1 | mRNA | UP | 1.290677 | 3.195887 | 7.592293 | 1.49E-13 | 3.77E-12 | 20.01723 |
| PODN | mRNA | UP | 1.289483 | 5.038709 | 15.21661 | 1.95E-43 | 7.30E-41 | 87.94068 |
| CCL18 | mRNA | UP | 1.289423 | 5.82432 | 8.761362 | 2.81E-17 | 1.07E-15 | 28.25306 |
| PPBP | mRNA | UP | 1.288787 | -1.71101 | 6.106634 | 2.01E-09 | 3.01E-08 | 11.02278 |
| GAP43 | mRNA | UP | 1.287704 | -1.98912 | 7.013151 | 7.37E-12 | 1.52E-10 | 16.44846 |
| LRFN5 | mRNA | UP | 1.285897 | -1.22116 | 12.23636 | 2.15E-30 | 2.38E-28 | 58.12342 |
| NCKAP1L | mRNA | UP | 1.285883 | 5.264012 | 16.27395 | 2.48E-48 | 1.90E-45 | 99.12752 |
| HSD11B1 | mRNA | UP | 1.285808 | 2.30271 | 13.60543 | 3.18E-36 | 5.67E-34 | 71.48581 |
| BOC | mRNA | UP | 1.285151 | 2.356537 | 16.21609 | 4.62E-48 | 3.41E-45 | 98.42208 |
| HGF | mRNA | UP | 1.284975 | 3.158102 | 13.68191 | 1.47E-36 | 2.74E-34 | 72.2576 |
| CMKLR1 | mRNA | UP | 1.283953 | 3.985424 | 14.81722 | 1.28E-41 | 3.99E-39 | 83.79388 |
| CCR8 | mRNA | UP | 1.283524 | -0.77493 | 11.92929 | 3.94E-29 | 3.98E-27 | 55.29408 |
| THBS1 | mRNA | UP | 1.283089 | 8.013335 | 13.27365 | 8.77E-35 | 1.38E-32 | 68.00598 |
| CD80 | mRNA | UP | 1.282169 | 0.736028 | 14.90281 | 5.25E-42 | 1.68E-39 | 84.5388 |
| MPEG1 | mRNA | UP | 1.281876 | 5.185987 | 16.04174 | 3.01E-47 | 2.00E-44 | 96.6486 |
| CLEC17A | mRNA | UP | 1.281863 | -1.35972 | 8.990772 | 4.70E-18 | 1.92E-16 | 30.3184 |
| HPSE2 | mRNA | UP | 1.281293 | -1.22185 | 7.908499 | 1.60E-14 | 4.56E-13 | 22.38665 |
| CASS4 | mRNA | UP | 1.28114 | 2.569425 | 16.01188 | 4.15E-47 | 2.60E-44 | 96.26593 |
| FCN1 | mRNA | UP | 1.279681 | 2.964349 | 10.65456 | 4.38E-24 | 3.02E-22 | 43.88483 |
| EMILIN1 | mRNA | UP | 1.278091 | 6.140152 | 16.11466 | 1.38E-47 | 9.34E-45 | 97.41191 |
| DOCK2 | mRNA | UP | 1.277661 | 4.788888 | 16.4666 | 3.09E-49 | 2.66E-46 | 101.1911 |
| SCIMP | mRNA | UP | 1.274629 | 2.597194 | 14.06407 | 3.05E-38 | 6.92E-36 | 76.0838 |
| FAT3 | mRNA | UP | 1.273795 | -0.57159 | 8.504202 | 2.00E-16 | 7.05E-15 | 26.6498 |
| NRK | mRNA | UP | 1.273272 | 1.316076 | 6.039135 | 2.97E-09 | 4.35E-08 | 10.4924 |
| DTHD1 | mRNA | UP | 1.273198 | -0.04961 | 7.353385 | 7.68E-13 | 1.78E-11 | 18.58963 |
| SNAI2 | mRNA | UP | 1.271532 | 2.856279 | 12.76538 | 1.31E-32 | 1.74E-30 | 63.26806 |
| ADAMTS2 | mRNA | UP | 1.271119 | 4.753677 | 14.14239 | 1.37E-38 | 3.25E-36 | 76.87741 |
| GAPT | mRNA | UP | 1.27079 | 0.975627 | 12.38708 | 5.09E-31 | 6.00E-29 | 59.63564 |
| NPTX2 | mRNA | UP | 1.269171 | 1.005565 | 7.360388 | 7.32E-13 | 1.70E-11 | 18.58954 |
| CD300C | mRNA | UP | 1.269114 | 1.345535 | 13.62709 | 2.56E-36 | 4.63E-34 | 71.67099 |
| NLRP3 | mRNA | UP | 1.269079 | 2.012798 | 15.06649 | 9.45E-43 | 3.31E-40 | 86.31877 |
| TNFSF8 | mRNA | UP | 1.266474 | 1.360394 | 15.03271 | 1.35E-42 | 4.68E-40 | 85.92813 |
| BRINP3 | mRNA | UP | 1.264988 | -1.38269 | 8.86527 | 1.26E-17 | 4.92E-16 | 29.36027 |
| FAT4 | mRNA | UP | 1.26425 | 3.454303 | 13.20095 | 1.80E-34 | 2.77E-32 | 67.4997 |
| PTPRD | mRNA | UP | 1.264111 | 2.251542 | 12.62574 | 5.08E-32 | 6.40E-30 | 61.92633 |
| BTK | mRNA | UP | 1.264086 | 3.444703 | 16.12243 | 1.27E-47 | 8.69E-45 | 97.48132 |
| CD1C | mRNA | UP | 1.263577 | 1.568691 | 8.503721 | 2.01E-16 | 7.07E-15 | 26.58331 |
| TNC | mRNA | UP | 1.263548 | 7.352844 | 8.081695 | 4.60E-15 | 1.40E-13 | 23.08803 |
| LMOD1 | mRNA | UP | 1.263541 | 3.682869 | 14.90537 | 5.11E-42 | 1.65E-39 | 84.7032 |
| ROR2 | mRNA | UP | 1.262811 | 2.74743 | 14.46844 | 4.79E-40 | 1.31E-37 | 80.19244 |
| HEPH | mRNA | UP | 1.262656 | 3.797504 | 15.66006 | 1.78E-45 | 8.81E-43 | 92.59238 |
| LY86 | mRNA | UP | 1.262396 | 2.871709 | 13.98623 | 6.74E-38 | 1.46E-35 | 75.30445 |
| C2orf40 | mRNA | UP | 1.262341 | -0.56603 | 6.224088 | 1.01E-09 | 1.58E-08 | 11.64571 |
| MMP9 | mRNA | UP | 1.261773 | 5.568187 | 9.464156 | 1.06E-19 | 5.03E-18 | 33.77553 |
| PIEZO2 | mRNA | UP | 1.261539 | 3.067819 | 13.23208 | 1.33E-34 | 2.05E-32 | 67.80677 |
| HLA-DOA | mRNA | UP | 1.260841 | 5.566762 | 11.71288 | 2.99E-28 | 2.76E-26 | 53.24926 |
| KCNMB1 | mRNA | UP | 1.258595 | 1.975857 | 16.20545 | 5.18E-48 | 3.78E-45 | 98.28138 |
| LAMP5 | mRNA | UP | 1.258247 | 1.97355 | 10.83457 | 8.90E-25 | 6.38E-23 | 45.47966 |
| LTBP2 | mRNA | UP | 1.257545 | 7.43046 | 15.22041 | 1.87E-43 | 7.06E-41 | 87.89484 |
| GBP6 | mRNA | UP | 1.257335 | -0.36342 | 6.554573 | 1.36E-10 | 2.41E-09 | 13.57396 |
| EMR1 | mRNA | UP | 1.256124 | 0.369877 | 9.745691 | 1.05E-20 | 5.42E-19 | 36.28596 |
| PLAU | mRNA | UP | 1.254829 | 6.651114 | 9.270221 | 5.10E-19 | 2.29E-17 | 32.137 |
| PLEK | mRNA | UP | 1.254333 | 4.927038 | 14.13082 | 1.54E-38 | 3.60E-36 | 76.75638 |
| LBX1 | mRNA | UP | 1.253664 | -3.46952 | 10.15643 | 3.29E-22 | 1.93E-20 | 39.62416 |
| MAMDC2 | mRNA | UP | 1.253631 | 2.376253 | 8.39124 | 4.68E-16 | 1.58E-14 | 25.71613 |
| CLEC4D | mRNA | UP | 1.253189 | -1.74009 | 10.36471 | 5.49E-23 | 3.44E-21 | 41.40881 |
| CXCL9 | mRNA | UP | 1.250605 | 5.471115 | 7.269236 | 1.35E-12 | 3.05E-11 | 17.68029 |
| CPXM1 | mRNA | UP | 1.249877 | 3.320449 | 11.73124 | 2.52E-28 | 2.34E-26 | 53.51209 |
| OLFML2B | mRNA | UP | 1.249586 | 4.730769 | 15.38166 | 3.41E-44 | 1.47E-41 | 89.67169 |
| ZEB2 | mRNA | UP | 1.247233 | 5.180289 | 21.47053 | 1.79E-73 | 2.61E-69 | 156.603 |
| CNN1 | mRNA | UP | 1.24721 | 3.205187 | 12.70312 | 2.40E-32 | 3.09E-30 | 62.66637 |
| CCL23 | mRNA | UP | 1.247188 | -0.59996 | 9.518476 | 6.82E-20 | 3.29E-18 | 34.45529 |
| FMO3 | mRNA | UP | 1.246877 | 2.936108 | 13.07439 | 6.31E-34 | 9.23E-32 | 66.26391 |
| MYOCD | mRNA | UP | 1.245364 | -0.07048 | 8.01343 | 7.54E-15 | 2.23E-13 | 23.09617 |
| LILRB5 | mRNA | UP | 1.244737 | 2.28259 | 11.20037 | 3.33E-26 | 2.65E-24 | 48.71364 |
| CD4 | mRNA | UP | 1.244695 | 6.516338 | 17.89087 | 5.24E-56 | 9.57E-53 | 116.6747 |
| P2RY10 | mRNA | UP | 1.244648 | 1.178562 | 11.81604 | 1.14E-28 | 1.10E-26 | 54.30848 |
| SHISA6 | mRNA | UP | 1.244592 | -1.7019 | 7.473683 | 3.38E-13 | 8.19E-12 | 19.43194 |
| CSF2RB | mRNA | UP | 1.244492 | 4.30811 | 15.51314 | 8.48E-45 | 3.96E-42 | 91.05212 |
| KIAA0226L | mRNA | UP | 1.243738 | 1.940761 | 16.80979 | 7.46E-51 | 7.51E-48 | 104.7406 |
| SPSB4 | mRNA | UP | 1.24313 | -1.82217 | 9.88349 | 3.31E-21 | 1.79E-19 | 37.40067 |
| CNR2 | mRNA | UP | 1.242007 | -0.87773 | 8.333172 | 7.22E-16 | 2.38E-14 | 25.40288 |
| CPEB1 | mRNA | UP | 1.241845 | -0.65288 | 11.03937 | 1.42E-25 | 1.09E-23 | 47.26548 |
| PLXNC1 | mRNA | UP | 1.241264 | 4.923461 | 20.18141 | 3.89E-67 | 2.84E-63 | 142.1008 |
| MAGEL2 | mRNA | UP | 1.240788 | -1.02529 | 12.47248 | 2.24E-31 | 2.68E-29 | 60.3535 |
| PTGDS | mRNA | UP | 1.240383 | 5.358395 | 10.01022 | 1.14E-21 | 6.40E-20 | 38.26942 |
| WISP2 | mRNA | UP | 1.239635 | 2.836279 | 10.97571 | 2.52E-25 | 1.88E-23 | 46.70299 |
| SIGLEC8 | mRNA | UP | 1.23926 | 0.565757 | 9.666618 | 2.02E-20 | 1.02E-18 | 35.64089 |
| LAMA2 | mRNA | UP | 1.239205 | 4.471569 | 15.26364 | 1.19E-43 | 4.65E-41 | 88.43593 |
| AOAH | mRNA | UP | 1.23899 | 3.178966 | 13.72081 | 9.94E-37 | 1.88E-34 | 72.64597 |
| SHANK1 | mRNA | UP | 1.238842 | -1.34004 | 13.20741 | 1.69E-34 | 2.61E-32 | 67.37712 |
| GLP2R | mRNA | UP | 1.238742 | -2.86106 | 6.478871 | 2.17E-10 | 3.73E-09 | 13.19782 |
| SYNPO2 | mRNA | UP | 1.238274 | 3.581773 | 14.89581 | 5.65E-42 | 1.79E-39 | 84.60298 |
| HAVCR2 | mRNA | UP | 1.237801 | 4.316803 | 15.08537 | 7.75E-43 | 2.73E-40 | 86.57579 |
| SH2D1A | mRNA | UP | 1.237513 | 1.557154 | 11.15916 | 4.83E-26 | 3.79E-24 | 48.35345 |
| C1QC | mRNA | UP | 1.237451 | 7.213885 | 12.68106 | 2.97E-32 | 3.82E-30 | 62.28099 |
| GPR68 | mRNA | UP | 1.237017 | 3.467307 | 13.2957 | 7.04E-35 | 1.11E-32 | 68.4306 |
| TMEM236 | mRNA | UP | 1.236982 | 0.122491 | 10.08718 | 5.93E-22 | 3.41E-20 | 39.10273 |
| SPARC | mRNA | UP | 1.236799 | 9.959426 | 17.32304 | 2.73E-53 | 3.19E-50 | 110.2619 |
| STAC | mRNA | UP | 1.23515 | 2.024828 | 8.467992 | 2.63E-16 | 9.17E-15 | 26.29856 |
| TDO2 | mRNA | UP | 1.234142 | 1.89478 | 10.55312 | 1.07E-23 | 7.10E-22 | 43.03551 |
| CX3CR1 | mRNA | UP | 1.233838 | 1.487567 | 8.875824 | 1.16E-17 | 4.55E-16 | 29.38646 |
| LRRC4C | mRNA | UP | 1.233286 | -0.60933 | 12.40324 | 4.35E-31 | 5.16E-29 | 59.72341 |
| GSTM5 | mRNA | UP | 1.233009 | 1.136595 | 13.74769 | 7.58E-37 | 1.45E-34 | 72.86038 |
| IRF4 | mRNA | UP | 1.232757 | 3.108478 | 10.38858 | 4.47E-23 | 2.83E-21 | 41.58863 |
| MSC | mRNA | UP | 1.23275 | 2.494132 | 13.58401 | 3.94E-36 | 6.95E-34 | 71.27668 |
| CYP1B1 | mRNA | UP | 1.232348 | 5.706825 | 12.99774 | 1.34E-33 | 1.90E-31 | 65.44679 |
| HMCN1 | mRNA | UP | 1.231874 | 4.588099 | 12.25415 | 1.82E-30 | 2.02E-28 | 58.3512 |
| XIRP1 | mRNA | UP | 1.230634 | -0.62116 | 7.77198 | 4.24E-14 | 1.15E-12 | 21.42839 |
| IGLL5 | mRNA | UP | 1.23057 | 6.081315 | 7.67385 | 8.45E-14 | 2.19E-12 | 20.3473 |
| TRHDE | mRNA | UP | 1.230522 | -1.62209 | 6.019251 | 3.34E-09 | 4.84E-08 | 10.53123 |
| MRVI1 | mRNA | UP | 1.230345 | 3.793071 | 17.7522 | 2.43E-55 | 3.94E-52 | 115.0997 |
| STMN2 | mRNA | UP | 1.230039 | -1.66622 | 6.201023 | 1.15E-09 | 1.79E-08 | 11.55599 |
| TPSB2 | mRNA | UP | 1.2299 | 3.244319 | 7.563673 | 1.82E-13 | 4.55E-12 | 19.81957 |
| AIM2 | mRNA | UP | 1.229818 | 2.160245 | 7.398867 | 5.64E-13 | 1.33E-11 | 18.78149 |
| TNFRSF17 | mRNA | UP | 1.229595 | 1.071137 | 8.23073 | 1.54E-15 | 4.92E-14 | 24.60756 |
| PLXNA4 | mRNA | UP | 1.229388 | 1.919123 | 11.21011 | 3.05E-26 | 2.43E-24 | 48.8056 |
| DCSTAMP | mRNA | UP | 1.22872 | 0.047337 | 8.886541 | 1.06E-17 | 4.21E-16 | 29.50827 |
| GPR87 | mRNA | UP | 1.228517 | 1.049491 | 4.28196 | 2.21E-05 | 0.000162 | 1.958352 |
| PDLIM3 | mRNA | UP | 1.227808 | 4.632032 | 14.79099 | 1.69E-41 | 5.13E-39 | 83.52108 |
| KRT5 | mRNA | UP | 1.22744 | 1.350672 | 5.031334 | 6.74E-07 | 6.60E-06 | 5.261485 |
| IL13RA2 | mRNA | UP | 1.227308 | -0.7651 | 6.857597 | 2.02E-11 | 3.95E-10 | 15.43969 |
| LOX | mRNA | UP | 1.226932 | 4.893364 | 13.08907 | 5.46E-34 | 8.03E-32 | 66.37288 |
| KCNA3 | mRNA | UP | 1.226816 | 1.391547 | 9.103263 | 1.93E-18 | 8.23E-17 | 31.14418 |
| MYL3 | mRNA | UP | 1.226612 | -1.76579 | 11.72802 | 2.60E-28 | 2.40E-26 | 53.40102 |
| CCL21 | mRNA | UP | 1.226432 | 4.756085 | 8.801286 | 2.06E-17 | 7.96E-16 | 28.63966 |
| HHIP | mRNA | UP | 1.226212 | 1.866224 | 5.154787 | 3.63E-07 | 3.71E-06 | 5.817779 |
| COL6A2 | mRNA | UP | 1.226051 | 8.377781 | 13.90711 | 1.51E-37 | 3.16E-35 | 74.30468 |
| EDIL3 | mRNA | UP | 1.22508 | 5.061653 | 11.04925 | 1.30E-25 | 1.00E-23 | 47.26174 |
| BRINP1 | mRNA | UP | 1.22452 | -0.17706 | 4.438176 | 1.11E-05 | 8.62E-05 | 2.698165 |
| PRDM6 | mRNA | UP | 1.224166 | 2.295798 | 14.40817 | 8.93E-40 | 2.39E-37 | 79.56422 |
| KLK4 | mRNA | UP | 1.223978 | -3.4302 | 6.570517 | 1.23E-10 | 2.20E-09 | 13.74939 |
| FAM26E | mRNA | UP | 1.223658 | 2.428505 | 17.62812 | 9.54E-55 | 1.39E-51 | 113.6441 |
| SLAMF8 | mRNA | UP | 1.223399 | 4.500572 | 13.95001 | 9.74E-38 | 2.08E-35 | 74.93686 |
| IRF8 | mRNA | UP | 1.223382 | 4.350919 | 14.5745 | 1.60E-40 | 4.58E-38 | 81.29324 |
| CSDC2 | mRNA | UP | 1.221882 | 1.714188 | 13.04173 | 8.71E-34 | 1.26E-31 | 65.9323 |
| FCGR2B | mRNA | UP | 1.221634 | 3.561064 | 13.48019 | 1.12E-35 | 1.91E-33 | 70.25195 |
| LAIR1 | mRNA | UP | 1.220425 | 4.960787 | 14.92905 | 3.99E-42 | 1.32E-39 | 84.9468 |
| FABP4 | mRNA | UP | 1.218989 | 0.811198 | 6.841903 | 2.23E-11 | 4.34E-10 | 15.27174 |
| LRRTM1 | mRNA | UP | 1.218596 | -3.04445 | 4.624793 | 4.75E-06 | 3.95E-05 | 3.636796 |
| SAA1 | mRNA | UP | 1.218414 | 2.188131 | 5.512751 | 5.60E-08 | 6.63E-07 | 7.592922 |
| CD86 | mRNA | UP | 1.217894 | 3.975144 | 16.02439 | 3.63E-47 | 2.30E-44 | 96.45394 |
| C1QB | mRNA | UP | 1.217251 | 7.431861 | 11.81606 | 1.14E-28 | 1.10E-26 | 54.07251 |
| MS4A7 | mRNA | UP | 1.216813 | 4.631635 | 14.29361 | 2.90E-39 | 7.34E-37 | 78.41688 |
| ZNF423 | mRNA | UP | 1.21677 | 1.442769 | 15.87144 | 1.87E-46 | 1.06E-43 | 94.6996 |
| IGF1 | mRNA | UP | 1.216522 | 0.757499 | 11.56863 | 1.14E-27 | 1.02E-25 | 52.03762 |
| ANGPTL2 | mRNA | UP | 1.216493 | 5.451738 | 15.13541 | 4.58E-43 | 1.64E-40 | 87.08547 |
| TAGLN | mRNA | UP | 1.21609 | 7.195814 | 15.46271 | 1.45E-44 | 6.45E-42 | 90.45384 |
| EVI2B | mRNA | UP | 1.215806 | 4.362101 | 16.28958 | 2.09E-48 | 1.63E-45 | 99.28899 |
| ENTHD1 | mRNA | UP | 1.215323 | -2.40679 | 8.255477 | 1.28E-15 | 4.14E-14 | 24.85413 |
| IGLL1 | mRNA | UP | 1.214537 | -2.72965 | 7.219158 | 1.89E-12 | 4.20E-11 | 17.77698 |
| DLG2 | mRNA | UP | 1.214235 | 1.722879 | 15.68594 | 1.35E-45 | 6.86E-43 | 92.76861 |
| HAS2 | mRNA | UP | 1.214233 | 2.22737 | 11.27345 | 1.71E-26 | 1.40E-24 | 49.36951 |
| PNOC | mRNA | UP | 1.213603 | 0.049134 | 8.96754 | 5.64E-18 | 2.29E-16 | 30.1283 |
| ICAM3 | mRNA | UP | 1.213431 | 1.492857 | 13.3506 | 4.08E-35 | 6.56E-33 | 68.94586 |
| C1QTNF1 | mRNA | UP | 1.213111 | 4.164835 | 15.34292 | 5.14E-44 | 2.13E-41 | 89.26449 |
| BTLA | mRNA | UP | 1.212499 | 0.306135 | 11.50171 | 2.11E-27 | 1.85E-25 | 51.42185 |
| NID2 | mRNA | UP | 1.212423 | 3.349026 | 12.28362 | 1.37E-30 | 1.55E-28 | 58.665 |
| P2RX7 | mRNA | UP | 1.212088 | 2.944564 | 13.76282 | 6.50E-37 | 1.26E-34 | 73.064 |
| CYS1 | mRNA | UP | 1.211731 | 1.609243 | 8.667398 | 5.79E-17 | 2.14E-15 | 27.80232 |
| PABPC5 | mRNA | UP | 1.211407 | -0.92278 | 14.29549 | 2.85E-39 | 7.23E-37 | 78.19298 |
| ACTA2 | mRNA | UP | 1.211148 | 7.167858 | 15.65404 | 1.90E-45 | 9.31E-43 | 92.47583 |
| CADM3 | mRNA | UP | 1.210782 | 0.751076 | 8.44816 | 3.05E-16 | 1.06E-14 | 26.20411 |
| TAGAP | mRNA | UP | 1.209459 | 3.442452 | 15.48488 | 1.14E-44 | 5.14E-42 | 90.74098 |
| FCRL4 | mRNA | UP | 1.209127 | -2.03518 | 7.287096 | 1.20E-12 | 2.72E-11 | 18.20821 |
| CCL14 | mRNA | UP | 1.209119 | -0.48114 | 8.049875 | 5.79E-15 | 1.74E-13 | 23.36449 |
| SV2B | mRNA | UP | 1.209009 | 0.024369 | 9.796408 | 6.87E-21 | 3.61E-19 | 36.70221 |
| DACT3 | mRNA | UP | 1.209009 | 2.286733 | 16.84963 | 4.84E-51 | 4.95E-48 | 105.1994 |
| CD53 | mRNA | UP | 1.208947 | 5.793371 | 15.96287 | 7.01E-47 | 4.27E-44 | 95.8014 |
| NCAM2 | mRNA | UP | 1.20832 | 2.272045 | 10.89155 | 5.36E-25 | 3.91E-23 | 45.97423 |
| ELN | mRNA | UP | 1.207768 | 6.026481 | 9.556408 | 5.00E-20 | 2.44E-18 | 34.48406 |
| FAM216B | mRNA | UP | 1.207009 | 0.140287 | 4.909275 | 1.23E-06 | 1.15E-05 | 4.771295 |
| CPXM2 | mRNA | UP | 1.206986 | 3.743532 | 9.19859 | 9.05E-19 | 3.98E-17 | 31.78386 |
| VSTM1 | mRNA | UP | 1.206859 | -2.93331 | 7.051189 | 5.75E-12 | 1.20E-10 | 16.70407 |
| MROH9 | mRNA | UP | 1.206329 | -3.00048 | 6.293405 | 6.66E-10 | 1.07E-08 | 12.11808 |
| SIGLEC7 | mRNA | UP | 1.205866 | 1.217967 | 13.36212 | 3.63E-35 | 5.88E-33 | 69.04896 |
| GPR65 | mRNA | UP | 1.205841 | 2.329736 | 15.26224 | 1.21E-43 | 4.69E-41 | 88.3702 |
| NAP1L3 | mRNA | UP | 1.205163 | 0.604373 | 14.35716 | 1.51E-39 | 3.97E-37 | 78.95278 |
| SAA2 | mRNA | UP | 1.204802 | 0.829566 | 5.274258 | 1.97E-07 | 2.11E-06 | 6.480684 |
| HTR7 | mRNA | UP | 1.204783 | -1.38133 | 11.03564 | 1.47E-25 | 1.12E-23 | 47.21047 |
| GJA5 | mRNA | UP | 1.204668 | 4.379748 | 15.37924 | 3.50E-44 | 1.49E-41 | 89.64631 |
| HS3ST3A1 | mRNA | UP | 1.204663 | -0.27268 | 11.02911 | 1.56E-25 | 1.19E-23 | 47.18431 |
| HDC | mRNA | UP | 1.203804 | 0.794748 | 9.010225 | 4.03E-18 | 1.66E-16 | 30.44067 |
| HLA-DPA1 | mRNA | UP | 1.202718 | 8.615367 | 11.80526 | 1.26E-28 | 1.20E-26 | 53.86513 |
| CXCL10 | mRNA | UP | 1.20253 | 4.265596 | 7.972906 | 1.01E-14 | 2.94E-13 | 22.58298 |
| TSPAN2 | mRNA | UP | 1.202351 | 2.077777 | 14.78659 | 1.77E-41 | 5.34E-39 | 83.43086 |
| PVALB | mRNA | UP | 1.201907 | -2.15466 | 7.993589 | 8.71E-15 | 2.55E-13 | 22.99288 |
| NXPE4 | mRNA | UP | 1.20089 | -3.70271 | 7.692994 | 7.39E-14 | 1.93E-12 | 20.9239 |
| ABCD2 | mRNA | UP | 1.200126 | 0.159227 | 11.8291 | 1.01E-28 | 9.77E-27 | 54.40683 |
| CXCR5 | mRNA | UP | 1.200048 | -2.43354 | 7.836363 | 2.68E-14 | 7.43E-13 | 21.90068 |
| TNN | mRNA | UP | 1.200014 | -1.01419 | 9.575773 | 4.26E-20 | 2.10E-18 | 34.91357 |
| ADORA3 | mRNA | UP | 1.199569 | 2.6768 | 13.03932 | 8.92E-34 | 1.28E-31 | 65.92175 |
| RARRES2 | mRNA | UP | 1.199451 | 5.853584 | 13.70011 | 1.22E-36 | 2.31E-34 | 72.38431 |
| HLA-DRA | mRNA | UP | 1.19887 | 10.13486 | 12.19397 | 3.22E-30 | 3.50E-28 | 57.34743 |
| MMRN1 | mRNA | UP | 1.198856 | 2.45519 | 9.737546 | 1.12E-20 | 5.78E-19 | 36.16942 |
| MZB1 | mRNA | UP | 1.197665 | 4.593297 | 8.034572 | 6.47E-15 | 1.94E-13 | 22.99614 |
| ZNF365 | mRNA | UP | 1.197634 | 0.463922 | 11.70409 | 3.25E-28 | 2.98E-26 | 53.26786 |
| ITGB2 | mRNA | UP | 1.196398 | 7.264514 | 13.05752 | 7.45E-34 | 1.08E-31 | 65.93691 |
| GFRA2 | mRNA | UP | 1.195639 | -0.24537 | 10.86866 | 6.57E-25 | 4.76E-23 | 45.77585 |
| GPR141 | mRNA | UP | 1.193405 | -0.27852 | 12.09192 | 8.49E-30 | 9.00E-28 | 56.82292 |
| ITGA4 | mRNA | UP | 1.193398 | 3.863098 | 15.76675 | 5.71E-46 | 3.06E-43 | 93.72032 |
| IQSEC3 | mRNA | UP | 1.192695 | 0.297966 | 10.63859 | 5.04E-24 | 3.45E-22 | 43.78358 |
| CLEC4A | mRNA | UP | 1.192491 | 2.101804 | 14.04457 | 3.72E-38 | 8.38E-36 | 75.87395 |
| GDNF | mRNA | UP | 1.19212 | -1.69186 | 7.520267 | 2.45E-13 | 6.07E-12 | 19.74272 |
| CD33 | mRNA | UP | 1.191929 | 2.148703 | 13.56455 | 4.79E-36 | 8.38E-34 | 71.07692 |
| IKZF1 | mRNA | UP | 1.190646 | 4.232161 | 14.76687 | 2.17E-41 | 6.53E-39 | 83.27426 |
| PIK3AP1 | mRNA | UP | 1.19059 | 4.678523 | 14.13686 | 1.45E-38 | 3.41E-36 | 76.82275 |
| PEG3 | mRNA | UP | 1.190451 | 0.820064 | 11.25364 | 2.05E-26 | 1.66E-24 | 49.19632 |
| SUCNR1 | mRNA | UP | 1.190356 | 1.9772 | 9.123584 | 1.65E-18 | 7.07E-17 | 31.28145 |
| ICOS | mRNA | UP | 1.18963 | 1.021886 | 11.24668 | 2.18E-26 | 1.76E-24 | 49.13535 |
| HLA-DRB5 | mRNA | UP | 1.189374 | 7.572159 | 9.084441 | 2.25E-18 | 9.47E-17 | 30.58621 |
| ARHGAP20 | mRNA | UP | 1.188621 | 2.381479 | 9.190653 | 9.65E-19 | 4.23E-17 | 31.78972 |
| SIRPB2 | mRNA | UP | 1.188294 | 2.208359 | 13.88625 | 1.86E-37 | 3.87E-35 | 74.28609 |
| CCDC170 | mRNA | UP | 1.187737 | 2.074536 | 9.291075 | 4.31E-19 | 1.95E-17 | 32.59336 |
| KCNS2 | mRNA | UP | 1.187349 | -2.38968 | 9.900754 | 2.87E-21 | 1.56E-19 | 37.5319 |
| IL16 | mRNA | UP | 1.186669 | 4.201745 | 15.94333 | 8.65E-47 | 5.10E-44 | 95.59711 |
| SIGLEC12 | mRNA | UP | 1.186377 | -0.04511 | 7.311457 | 1.02E-12 | 2.33E-11 | 18.31364 |
| CXCL11 | mRNA | UP | 1.186089 | 1.901769 | 7.292779 | 1.16E-12 | 2.62E-11 | 18.09533 |
| FLRT2 | mRNA | UP | 1.185445 | 3.032529 | 10.67522 | 3.65E-24 | 2.53E-22 | 44.06192 |
| TNFRSF9 | mRNA | UP | 1.18452 | 2.267816 | 10.23469 | 1.68E-22 | 1.02E-20 | 40.30899 |
| SFTPC | mRNA | UP | 1.183866 | 6.128711 | 2.995829 | 0.002869 | 0.012349 | -3.05503 |
| HMGA2 | mRNA | UP | 1.183747 | 0.470311 | 3.896711 | 0.00011 | 0.000692 | 0.487757 |
| AICDA | mRNA | UP | 1.183458 | -3.18629 | 7.577514 | 1.65E-13 | 4.15E-12 | 20.14243 |
| SIGLEC9 | mRNA | UP | 1.183369 | 2.006213 | 13.96386 | 8.46E-38 | 1.82E-35 | 75.05881 |
| LILRB4 | mRNA | UP | 1.182917 | 4.522942 | 11.41954 | 4.50E-27 | 3.85E-25 | 50.61901 |
| VWC2 | mRNA | UP | 1.182595 | -3.66504 | 8.211385 | 1.78E-15 | 5.64E-14 | 24.53863 |
| THY1 | mRNA | UP | 1.182402 | 6.132604 | 13.86476 | 2.32E-37 | 4.71E-35 | 74.02468 |
| L1CAM | mRNA | UP | 1.182293 | 0.254098 | 7.036753 | 6.32E-12 | 1.31E-10 | 16.52568 |
| ZNF831 | mRNA | UP | 1.181888 | 0.867545 | 10.34224 | 6.67E-23 | 4.16E-21 | 41.24585 |
| SLIT2 | mRNA | UP | 1.181851 | 4.092676 | 13.60926 | 3.06E-36 | 5.48E-34 | 71.52886 |
| THEMIS | mRNA | UP | 1.181623 | 1.097931 | 10.32019 | 8.07E-23 | 5.00E-21 | 41.05661 |
| CMA1 | mRNA | UP | 1.181118 | -3.23722 | 7.91313 | 1.55E-14 | 4.42E-13 | 22.43601 |
| PTGER4 | mRNA | UP | 1.180729 | 3.440622 | 14.44412 | 6.16E-40 | 1.67E-37 | 79.95623 |
| PI16 | mRNA | UP | 1.179668 | -1.33603 | 6.547404 | 1.42E-10 | 2.51E-09 | 13.5678 |
| SLN | mRNA | UP | 1.179497 | -3.02588 | 7.001988 | 7.93E-12 | 1.63E-10 | 16.39418 |
| TBX5 | mRNA | UP | 1.179348 | 2.716589 | 14.04322 | 3.77E-38 | 8.47E-36 | 75.87606 |
| KLHL6 | mRNA | UP | 1.178561 | 3.751464 | 13.41725 | 2.10E-35 | 3.47E-33 | 69.62729 |
| NTM | mRNA | UP | 1.178155 | 3.389455 | 11.23852 | 2.35E-26 | 1.89E-24 | 49.02877 |
| HLA-DQA2 | mRNA | UP | 1.178145 | 4.777955 | 6.406652 | 3.36E-10 | 5.64E-09 | 12.34762 |
| ITIH5 | mRNA | UP | 1.175927 | 3.378033 | 9.114901 | 1.76E-18 | 7.55E-17 | 31.14793 |
| GRP | mRNA | UP | 1.175494 | -1.13408 | 5.383194 | 1.11E-07 | 1.25E-06 | 7.139626 |
| APOC4-APOC2 | mRNA | UP | 1.174694 | -3.01109 | 8.475039 | 2.50E-16 | 8.73E-15 | 26.4475 |
| TRAT1 | mRNA | UP | 1.173623 | 0.463114 | 9.615863 | 3.07E-20 | 1.53E-18 | 35.23149 |
| GADL1 | mRNA | UP | 1.17328 | -3.53871 | 7.363609 | 7.16E-13 | 1.67E-11 | 18.72374 |
| DSE | mRNA | UP | 1.172561 | 4.729995 | 14.81898 | 1.26E-41 | 3.93E-39 | 83.80945 |
| HCK | mRNA | UP | 1.172211 | 4.726956 | 13.13525 | 3.46E-34 | 5.20E-32 | 66.83043 |
| GUCA1A | mRNA | UP | 1.172166 | -2.97347 | 6.642244 | 7.88E-11 | 1.44E-09 | 14.17493 |
| RNASE6 | mRNA | UP | 1.171643 | 3.763135 | 16.34835 | 1.11E-48 | 8.88E-46 | 99.90285 |
| ABCA8 | mRNA | UP | 1.171225 | 2.188981 | 8.376877 | 5.21E-16 | 1.75E-14 | 25.62003 |
| FCGR1A | mRNA | UP | 1.170934 | 2.43329 | 12.23252 | 2.23E-30 | 2.46E-28 | 58.1933 |
| LAPTM5 | mRNA | UP | 1.170663 | 7.965147 | 15.96776 | 6.65E-47 | 4.09E-44 | 95.7642 |
| STAP1 | mRNA | UP | 1.170501 | 0.199087 | 10.71803 | 2.50E-24 | 1.75E-22 | 44.47039 |
| SLAMF7 | mRNA | UP | 1.16957 | 4.990381 | 10.30423 | 9.26E-23 | 5.70E-21 | 40.77425 |
| TRPA1 | mRNA | UP | 1.16932 | -1.6994 | 6.807922 | 2.77E-11 | 5.35E-10 | 15.16027 |
| FHL1 | mRNA | UP | 1.168477 | 4.393428 | 10.69202 | 3.15E-24 | 2.19E-22 | 44.15017 |
| MATN3 | mRNA | UP | 1.168411 | 2.617113 | 10.29156 | 1.03E-22 | 6.33E-21 | 40.77972 |
| SSC5D | mRNA | UP | 1.167928 | 4.179984 | 12.9304 | 2.60E-33 | 3.59E-31 | 64.84652 |
| TNFSF13B | mRNA | UP | 1.167868 | 3.907231 | 13.87525 | 2.08E-37 | 4.27E-35 | 74.19188 |
| MAP1LC3C | mRNA | UP | 1.167767 | 0.145474 | 7.473721 | 3.38E-13 | 8.19E-12 | 19.38072 |
| CDH13 | mRNA | UP | 1.167722 | 3.005505 | 12.97605 | 1.66E-33 | 2.32E-31 | 65.30633 |
| CD22 | mRNA | UP | 1.167659 | 2.540133 | 9.51059 | 7.27E-20 | 3.50E-18 | 34.32557 |
| PPP2R2B | mRNA | UP | 1.16742 | 0.228778 | 12.60009 | 6.52E-32 | 8.05E-30 | 61.63045 |
| NEGR1 | mRNA | UP | 1.167353 | 1.611528 | 9.965638 | 1.66E-21 | 9.18E-20 | 38.07357 |
| SIRPB1 | mRNA | UP | 1.166766 | 1.699833 | 9.956213 | 1.80E-21 | 9.91E-20 | 37.99327 |
| IGDCC4 | mRNA | UP | 1.166253 | 1.457164 | 12.94992 | 2.15E-33 | 2.98E-31 | 65.03514 |
| ADAMTSL1 | mRNA | UP | 1.165435 | 1.224471 | 10.12402 | 4.34E-22 | 2.52E-20 | 39.40145 |
| CLEC12A | mRNA | UP | 1.164903 | 1.478186 | 9.380701 | 2.09E-19 | 9.67E-18 | 33.32447 |
| DKK3 | mRNA | UP | 1.164894 | 5.906287 | 14.49844 | 3.52E-40 | 9.73E-38 | 80.47769 |
| GBP5 | mRNA | UP | 1.164064 | 4.448181 | 9.392414 | 1.90E-19 | 8.82E-18 | 33.27802 |
| RGS4 | mRNA | UP | 1.163078 | 1.655655 | 9.949707 | 1.90E-21 | 1.05E-19 | 37.94046 |
| DOK2 | mRNA | UP | 1.162662 | 3.673649 | 13.57659 | 4.25E-36 | 7.47E-34 | 71.20812 |
| SELL | mRNA | UP | 1.162598 | 3.857362 | 10.7978 | 1.23E-24 | 8.78E-23 | 45.10026 |
| ANGPTL1 | mRNA | UP | 1.162112 | 0.539777 | 11.6325 | 6.31E-28 | 5.69E-26 | 52.61555 |
| CACNA1C | mRNA | UP | 1.161818 | 2.891901 | 14.69876 | 4.41E-41 | 1.31E-38 | 82.55668 |
| CRTAM | mRNA | UP | 1.161196 | 0.636702 | 10.97965 | 2.44E-25 | 1.82E-23 | 46.76141 |
| CCL24 | mRNA | UP | 1.159311 | -0.72664 | 7.510262 | 2.63E-13 | 6.47E-12 | 19.6542 |
| NKX3-2 | mRNA | UP | 1.158276 | -2.65862 | 6.234341 | 9.47E-10 | 1.49E-08 | 11.77272 |
| MEDAG | mRNA | UP | 1.158163 | 2.306267 | 9.75089 | 1.00E-20 | 5.20E-19 | 36.28389 |
| FAM69C | mRNA | UP | 1.157954 | -4.21123 | 6.809382 | 2.74E-11 | 5.30E-10 | 15.19717 |
| LAMA4 | mRNA | UP | 1.157726 | 5.716543 | 17.142 | 1.99E-52 | 2.19E-49 | 108.4915 |
| PAMR1 | mRNA | UP | 1.157244 | 1.970542 | 11.48106 | 2.56E-27 | 2.23E-25 | 51.24666 |
| RNASE2 | mRNA | UP | 1.157072 | 0.032993 | 9.92898 | 2.26E-21 | 1.24E-19 | 37.79078 |
| CCL17 | mRNA | UP | 1.156847 | 0.440489 | 6.705921 | 5.29E-11 | 9.87E-10 | 14.45201 |
| ITK | mRNA | UP | 1.156568 | 2.739451 | 11.21925 | 2.80E-26 | 2.24E-24 | 48.87366 |
| KLF17 | mRNA | UP | 1.156513 | -3.36134 | 9.895776 | 2.99E-21 | 1.62E-19 | 37.47424 |
| SAMSN1 | mRNA | UP | 1.15641 | 3.620444 | 13.76169 | 6.58E-37 | 1.27E-34 | 73.05478 |
| GPR183 | mRNA | UP | 1.154834 | 3.903849 | 12.34394 | 7.69E-31 | 8.98E-29 | 59.2233 |
| SPN | mRNA | UP | 1.154177 | 4.156939 | 12.58038 | 7.89E-32 | 9.72E-30 | 61.46979 |
| BLK | mRNA | UP | 1.153442 | 0.731882 | 7.256627 | 1.47E-12 | 3.31E-11 | 17.9209 |
| C1QA | mRNA | UP | 1.152199 | 7.20234 | 11.77967 | 1.60E-28 | 1.52E-26 | 53.75332 |
| BHLHE41 | mRNA | UP | 1.151785 | 3.986943 | 12.29603 | 1.22E-30 | 1.39E-28 | 58.76724 |
| PCDH7 | mRNA | UP | 1.151258 | 3.861096 | 7.69531 | 7.27E-14 | 1.90E-12 | 20.67559 |
| IGSF6 | mRNA | UP | 1.151224 | 4.260031 | 12.84914 | 5.77E-33 | 7.84E-31 | 64.05611 |
| HLA-DQB2 | mRNA | UP | 1.150644 | 5.202848 | 7.296809 | 1.12E-12 | 2.56E-11 | 17.88485 |
| FAM92B | mRNA | UP | 1.149845 | 0.061734 | 5.982488 | 4.12E-09 | 5.90E-08 | 10.25231 |
| FCGR1B | mRNA | UP | 1.149764 | -0.14135 | 11.87083 | 6.83E-29 | 6.74E-27 | 54.78102 |
| CASQ2 | mRNA | UP | 1.149397 | -1.05368 | 7.590137 | 1.51E-13 | 3.82E-12 | 20.19949 |
| ACTBL2 | mRNA | UP | 1.149193 | -2.48028 | 6.621764 | 8.96E-11 | 1.62E-09 | 14.04325 |
| IL10RA | mRNA | UP | 1.147855 | 5.067911 | 15.50385 | 9.36E-45 | 4.30E-42 | 90.95338 |
| TM6SF1 | mRNA | UP | 1.147592 | 1.603962 | 14.62779 | 9.21E-41 | 2.66E-38 | 81.7764 |
| SPIB | mRNA | UP | 1.14719 | 1.343298 | 7.557614 | 1.90E-13 | 4.74E-12 | 19.89035 |
| ADAM33 | mRNA | UP | 1.147141 | 0.614915 | 9.29228 | 4.27E-19 | 1.93E-17 | 32.6458 |
| IBSP | mRNA | UP | 1.146663 | -1.07971 | 4.942502 | 1.05E-06 | 9.89E-06 | 4.996948 |
| CA10 | mRNA | UP | 1.145887 | -2.70599 | 3.639089 | 0.000301 | 0.001703 | -0.26222 |
| ACSM5 | mRNA | UP | 1.145884 | -0.79893 | 8.417809 | 3.84E-16 | 1.31E-14 | 26.0186 |
| ANXA8 | mRNA | UP | 1.144906 | -0.96509 | 5.053065 | 6.05E-07 | 5.96E-06 | 5.512608 |
| AIF1 | mRNA | UP | 1.144735 | 4.645725 | 14.24241 | 4.91E-39 | 1.23E-36 | 77.8953 |
| CD79B | mRNA | UP | 1.144716 | 2.370248 | 9.905 | 2.77E-21 | 1.51E-19 | 37.54923 |
| LY6H | mRNA | UP | 1.14461 | -0.91392 | 8.607663 | 9.13E-17 | 3.33E-15 | 27.42079 |
| SIGLEC10 | mRNA | UP | 1.144199 | 3.177992 | 11.50802 | 2.00E-27 | 1.75E-25 | 51.4717 |
| FYB | mRNA | UP | 1.144128 | 4.690461 | 12.79945 | 9.38E-33 | 1.27E-30 | 63.56203 |
| PPFIA2 | mRNA | UP | 1.143028 | -0.97349 | 10.00245 | 1.22E-21 | 6.81E-20 | 38.39228 |
| SLAMF6 | mRNA | UP | 1.142439 | 2.702468 | 11.5106 | 1.95E-27 | 1.71E-25 | 51.50566 |
| ADRB3 | mRNA | UP | 1.142348 | -3.14173 | 7.257605 | 1.46E-12 | 3.28E-11 | 18.02997 |
| VAT1L | mRNA | UP | 1.142168 | -0.55638 | 8.515324 | 1.84E-16 | 6.50E-15 | 26.73165 |
| SGCA | mRNA | UP | 1.141986 | 1.417013 | 9.316843 | 3.51E-19 | 1.59E-17 | 32.81965 |
| SASH3 | mRNA | UP | 1.141636 | 4.478052 | 14.73773 | 2.94E-41 | 8.75E-39 | 82.97241 |
| DAZL | mRNA | UP | 1.14161 | -4.29655 | 7.284319 | 1.22E-12 | 2.77E-11 | 18.20344 |
| SELPLG | mRNA | UP | 1.141554 | 4.962333 | 15.5279 | 7.25E-45 | 3.41E-42 | 91.20742 |
| SNX20 | mRNA | UP | 1.140615 | 3.271972 | 14.13754 | 1.44E-38 | 3.40E-36 | 76.83669 |
| FAM13C | mRNA | UP | 1.140139 | 0.93661 | 13.25394 | 1.07E-34 | 1.67E-32 | 67.97635 |
| DOK5 | mRNA | UP | 1.139393 | 0.903745 | 8.70302 | 4.40E-17 | 1.65E-15 | 28.09578 |
| FHOD3 | mRNA | UP | 1.139012 | 1.958189 | 7.14703 | 3.06E-12 | 6.62E-11 | 17.14202 |
| CLECL1 | mRNA | UP | 1.139004 | -0.40737 | 11.11687 | 7.09E-26 | 5.52E-24 | 47.95702 |
| FCGR3B | mRNA | UP | 1.138944 | 0.318079 | 7.528324 | 2.32E-13 | 5.76E-12 | 19.73983 |
| CTSE | mRNA | UP | 1.138389 | 6.14413 | 4.404942 | 1.29E-05 | 9.87E-05 | 2.011482 |
| A2M | mRNA | UP | 1.138239 | 9.679451 | 13.38697 | 2.84E-35 | 4.63E-33 | 68.97227 |
| PTPN22 | mRNA | UP | 1.137795 | 3.093733 | 12.30154 | 1.15E-30 | 1.32E-28 | 58.83844 |
| RASGRP4 | mRNA | UP | 1.137606 | 1.239594 | 14.419 | 7.98E-40 | 2.16E-37 | 79.62442 |
| CCR5 | mRNA | UP | 1.13739 | 3.368036 | 12.66065 | 3.62E-32 | 4.63E-30 | 62.25622 |
| ADAMDEC1 | mRNA | UP | 1.136948 | 2.480006 | 6.709407 | 5.17E-11 | 9.67E-10 | 14.35077 |
| BEND6 | mRNA | UP | 1.136893 | 0.74799 | 9.552639 | 5.15E-20 | 2.52E-18 | 34.71743 |
| NXPH3 | mRNA | UP | 1.136805 | 0.904754 | 12.77623 | 1.18E-32 | 1.58E-30 | 63.34264 |
| ALDH1A3 | mRNA | UP | 1.136692 | 3.738225 | 10.24185 | 1.58E-22 | 9.64E-21 | 40.31311 |
| MCHR1 | mRNA | UP | 1.136334 | -0.89705 | 9.032525 | 3.38E-18 | 1.41E-16 | 30.63835 |
| NFAM1 | mRNA | UP | 1.135941 | 3.662301 | 13.09131 | 5.34E-34 | 7.87E-32 | 66.42409 |
| C1orf186 | mRNA | UP | 1.135683 | 1.084744 | 8.749523 | 3.08E-17 | 1.17E-15 | 28.44052 |
| XCR1 | mRNA | UP | 1.135609 | 0.291832 | 9.02863 | 3.49E-18 | 1.45E-16 | 30.5943 |
| CD40LG | mRNA | UP | 1.135438 | 0.312214 | 9.539662 | 5.73E-20 | 2.79E-18 | 34.61967 |
| DNASE2B | mRNA | UP | 1.135335 | -1.80122 | 6.875877 | 1.79E-11 | 3.53E-10 | 15.58375 |
| CD69 | mRNA | UP | 1.135262 | 2.925357 | 10.52537 | 1.36E-23 | 8.93E-22 | 42.76861 |
| TESPA1 | mRNA | UP | 1.134463 | 1.66253 | 12.61985 | 5.38E-32 | 6.74E-30 | 61.86359 |
| KCNK13 | mRNA | UP | 1.13301 | 0.323466 | 11.50095 | 2.13E-27 | 1.86E-25 | 51.41538 |
| PILRA | mRNA | UP | 1.132221 | 3.159546 | 14.32412 | 2.12E-39 | 5.53E-37 | 78.72916 |
| ANGPT4 | mRNA | UP | 1.131978 | -2.15258 | 8.154156 | 2.71E-15 | 8.45E-14 | 24.12804 |
| MAFB | mRNA | UP | 1.131632 | 5.212747 | 15.87767 | 1.75E-46 | 9.99E-44 | 94.90379 |
| LTF | mRNA | UP | 1.130915 | 5.080837 | 5.047202 | 6.23E-07 | 6.13E-06 | 5.022964 |
| SLAMF1 | mRNA | UP | 1.130903 | 1.934935 | 12.25382 | 1.82E-30 | 2.02E-28 | 58.39353 |
| FCGR2A | mRNA | UP | 1.130736 | 5.730486 | 14.6878 | 4.94E-41 | 1.45E-38 | 82.43247 |
| CDO1 | mRNA | UP | 1.129312 | 0.206013 | 10.22004 | 1.91E-22 | 1.15E-20 | 40.21543 |
| AMICA1 | mRNA | UP | 1.129256 | 4.179974 | 12.32217 | 9.47E-31 | 1.10E-28 | 59.00897 |
| AXL | mRNA | UP | 1.128762 | 5.543594 | 15.30465 | 7.70E-44 | 3.10E-41 | 88.85361 |
| OSCAR | mRNA | UP | 1.128731 | 3.086682 | 11.8423 | 8.93E-29 | 8.70E-27 | 54.54245 |
| MMP16 | mRNA | UP | 1.12841 | 0.711505 | 10.13568 | 3.93E-22 | 2.30E-20 | 39.50543 |
| CEACAM4 | mRNA | UP | 1.128223 | -0.04303 | 8.928949 | 7.64E-18 | 3.07E-16 | 29.83392 |
| BGN | mRNA | UP | 1.128095 | 8.956115 | 15.15114 | 3.88E-43 | 1.41E-40 | 87.05965 |
| SIGLEC1 | mRNA | UP | 1.127658 | 4.48755 | 10.4125 | 3.63E-23 | 2.31E-21 | 41.72878 |
| C8orf88 | mRNA | UP | 1.127527 | -1.37384 | 11.11409 | 7.27E-26 | 5.65E-24 | 47.90281 |
| METTL24 | mRNA | UP | 1.127414 | -1.41566 | 10.72734 | 2.30E-24 | 1.62E-22 | 44.51891 |
| FLT3 | mRNA | UP | 1.126374 | -0.12706 | 11.19445 | 3.51E-26 | 2.78E-24 | 48.65287 |
| SERPINF1 | mRNA | UP | 1.125309 | 6.693913 | 12.1708 | 4.02E-30 | 4.34E-28 | 57.44878 |
| LRRC17 | mRNA | UP | 1.125261 | 1.715357 | 11.96545 | 2.81E-29 | 2.90E-27 | 55.6953 |
| MYH1 | mRNA | UP | 1.125232 | -3.78539 | 6.980509 | 9.12E-12 | 1.87E-10 | 16.26394 |
| HMCN2 | mRNA | UP | 1.125103 | 1.232372 | 8.483541 | 2.34E-16 | 8.21E-15 | 26.44736 |
| HRH2 | mRNA | UP | 1.124371 | -0.08659 | 10.37951 | 4.83E-23 | 3.04E-21 | 41.56217 |
| RETN | mRNA | UP | 1.124027 | -0.51044 | 6.110373 | 1.97E-09 | 2.95E-08 | 10.99657 |
| FRMD6 | mRNA | UP | 1.12371 | 4.283506 | 11.83116 | 9.91E-29 | 9.59E-27 | 54.40433 |
| CEACAM21 | mRNA | UP | 1.1236 | 1.049802 | 11.85184 | 8.16E-29 | 7.98E-27 | 54.63696 |
| FAM26F | mRNA | UP | 1.12313 | 2.484675 | 9.106113 | 1.89E-18 | 8.06E-17 | 31.1237 |
| TGFBI | mRNA | UP | 1.122948 | 7.602229 | 9.981782 | 1.45E-21 | 8.06E-20 | 37.84613 |
| SLC18A2 | mRNA | UP | 1.12288 | 0.262135 | 8.385123 | 4.90E-16 | 1.65E-14 | 25.7568 |
| GPR31 | mRNA | UP | 1.122821 | -4.1954 | 8.266702 | 1.18E-15 | 3.82E-14 | 24.93525 |
| PTAFR | mRNA | UP | 1.122249 | 4.773803 | 12.09823 | 8.00E-30 | 8.52E-28 | 56.87502 |
| LRRC25 | mRNA | UP | 1.121754 | 3.031514 | 13.6875 | 1.39E-36 | 2.61E-34 | 72.31269 |
| FGF14 | mRNA | UP | 1.121589 | 1.082938 | 10.54112 | 1.18E-23 | 7.84E-22 | 42.94328 |
| SLC2A5 | mRNA | UP | 1.121566 | 2.649261 | 9.318953 | 3.45E-19 | 1.57E-17 | 32.79023 |
| AQP9 | mRNA | UP | 1.121187 | 2.975528 | 8.789305 | 2.26E-17 | 8.70E-16 | 28.65936 |
| SPRR1B | mRNA | UP | 1.120592 | -1.50166 | 3.381511 | 0.000776 | 0.003931 | -1.19172 |
| CYP26A1 | mRNA | UP | 1.120283 | -2.42959 | 5.248144 | 2.25E-07 | 2.38E-06 | 6.5178 |
| GHR | mRNA | UP | 1.119201 | 0.583515 | 11.43393 | 3.95E-27 | 3.38E-25 | 50.81421 |
| EPB41L2 | mRNA | UP | 1.118917 | 5.165965 | 18.15545 | 2.80E-57 | 6.28E-54 | 119.5807 |
| PKNOX2 | mRNA | UP | 1.118773 | 1.175854 | 10.23521 | 1.68E-22 | 1.02E-20 | 40.33682 |
| CSF2RA | mRNA | UP | 1.118715 | 2.750779 | 11.94682 | 3.34E-29 | 3.41E-27 | 55.51755 |
| GFPT2 | mRNA | UP | 1.118217 | 3.603407 | 11.77112 | 1.74E-28 | 1.64E-26 | 53.87198 |
| FCRL2 | mRNA | UP | 1.11658 | 1.010253 | 7.169617 | 2.63E-12 | 5.73E-11 | 17.34124 |
| SAMD3 | mRNA | UP | 1.116459 | 0.099549 | 12.23269 | 2.23E-30 | 2.46E-28 | 58.15326 |
| LCP1 | mRNA | UP | 1.116437 | 7.3677 | 13.62546 | 2.60E-36 | 4.69E-34 | 71.55069 |
| CCR1 | mRNA | UP | 1.11635 | 3.953071 | 12.87947 | 4.29E-33 | 5.84E-31 | 64.35725 |
| KCNA5 | mRNA | UP | 1.115665 | -1.23449 | 9.486939 | 8.82E-20 | 4.21E-18 | 34.20146 |
| IL10 | mRNA | UP | 1.114885 | -0.39373 | 11.65533 | 5.11E-28 | 4.62E-26 | 52.79724 |
| CXorf21 | mRNA | UP | 1.114261 | 1.679244 | 13.94722 | 1.00E-37 | 2.14E-35 | 74.87987 |
| FILIP1L | mRNA | UP | 1.113882 | 5.294494 | 14.00454 | 5.59E-38 | 1.23E-35 | 75.46762 |
| ARSI | mRNA | UP | 1.113526 | 1.634403 | 12.92028 | 2.87E-33 | 3.95E-31 | 64.75263 |
| EFCAB1 | mRNA | UP | 1.113415 | 0.042097 | 5.213844 | 2.68E-07 | 2.81E-06 | 6.23344 |
| GDF5 | mRNA | UP | 1.112813 | -0.76557 | 6.628755 | 8.57E-11 | 1.56E-09 | 14.03673 |
| GIMAP6 | mRNA | UP | 1.112577 | 4.184262 | 15.67639 | 1.50E-45 | 7.53E-43 | 92.77115 |
| KCTD12 | mRNA | UP | 1.112122 | 6.609788 | 15.74343 | 7.32E-46 | 3.89E-43 | 93.44703 |
| JPH2 | mRNA | UP | 1.11179 | 0.804394 | 10.46048 | 2.39E-23 | 1.55E-21 | 42.25356 |
| RSPO2 | mRNA | UP | 1.111612 | -1.11611 | 5.508241 | 5.74E-08 | 6.79E-07 | 7.775828 |
| SLC37A2 | mRNA | UP | 1.11151 | 3.691203 | 12.4869 | 1.95E-31 | 2.34E-29 | 60.58778 |
| GAL3ST4 | mRNA | UP | 1.110936 | 3.346193 | 14.92826 | 4.02E-42 | 1.32E-39 | 84.93507 |
| CD37 | mRNA | UP | 1.110811 | 4.806635 | 13.6274 | 2.55E-36 | 4.63E-34 | 71.69486 |
| CPVL | mRNA | UP | 1.110695 | 4.715204 | 11.11233 | 7.38E-26 | 5.73E-24 | 47.84293 |
| CNRIP1 | mRNA | UP | 1.110654 | 2.61062 | 19.79466 | 3.05E-65 | 1.62E-61 | 137.5562 |
| RUNX1T1 | mRNA | UP | 1.110568 | 1.911053 | 13.30814 | 6.22E-35 | 9.90E-33 | 68.54066 |
| CPN2 | mRNA | UP | 1.109655 | -3.91411 | 7.712525 | 6.44E-14 | 1.70E-12 | 21.05693 |
| SIT1 | mRNA | UP | 1.10957 | 1.580437 | 10.6013 | 6.99E-24 | 4.73E-22 | 43.45637 |
| CD19 | mRNA | UP | 1.109264 | 1.34768 | 7.59896 | 1.42E-13 | 3.61E-12 | 20.16996 |
| LILRB2 | mRNA | UP | 1.109163 | 3.288701 | 12.52098 | 1.40E-31 | 1.69E-29 | 60.92012 |
| TIMP2 | mRNA | UP | 1.108744 | 8.022542 | 15.94666 | 8.34E-47 | 4.97E-44 | 95.53539 |
| CCR7 | mRNA | UP | 1.108509 | 2.472255 | 10.04815 | 8.27E-22 | 4.70E-20 | 38.73527 |
| PTGDR | mRNA | UP | 1.107992 | -0.94826 | 11.41197 | 4.83E-27 | 4.11E-25 | 50.57418 |
| NOX4 | mRNA | UP | 1.107437 | 1.917754 | 11.2859 | 1.53E-26 | 1.25E-24 | 49.48498 |
| ZPLD1 | mRNA | UP | 1.10709 | -1.93131 | 5.853412 | 8.59E-09 | 1.16E-07 | 9.63137 |
| MUC16 | mRNA | UP | 1.105789 | 3.734802 | 3.810393 | 0.000156 | 0.00094 | -0.10974 |
| PCDHGA12 | mRNA | UP | 1.105535 | 0.462271 | 12.55175 | 1.04E-31 | 1.27E-29 | 61.18035 |
| CTGF | mRNA | UP | 1.105409 | 7.105827 | 12.0804 | 9.47E-30 | 9.99E-28 | 56.56715 |
| TNFSF4 | mRNA | UP | 1.104913 | 1.870667 | 13.01653 | 1.12E-33 | 1.59E-31 | 65.69077 |
| CYTH4 | mRNA | UP | 1.104611 | 4.285658 | 15.60866 | 3.07E-45 | 1.48E-42 | 92.05774 |
| C10orf128 | mRNA | UP | 1.104544 | 3.182785 | 15.2502 | 1.37E-43 | 5.29E-41 | 88.27767 |
| LST1 | mRNA | UP | 1.104482 | 3.710808 | 11.90783 | 4.83E-29 | 4.82E-27 | 55.13488 |
| ZNF366 | mRNA | UP | 1.104131 | 0.529702 | 11.80746 | 1.24E-28 | 1.18E-26 | 54.21794 |
| BCHE | mRNA | UP | 1.103931 | 0.544411 | 8.502658 | 2.03E-16 | 7.13E-15 | 26.61248 |
| NCF1 | mRNA | UP | 1.102282 | 1.934061 | 11.92481 | 4.11E-29 | 4.13E-27 | 55.31868 |
| CYTIP | mRNA | UP | 1.102213 | 4.069103 | 13.71628 | 1.04E-36 | 1.97E-34 | 72.597 |
| RARRES1 | mRNA | UP | 1.101704 | 4.312683 | 9.123041 | 1.65E-18 | 7.09E-17 | 31.15593 |
| SYT16 | mRNA | UP | 1.101458 | -0.80109 | 5.371351 | 1.19E-07 | 1.32E-06 | 7.063955 |
| NAIP | mRNA | UP | 1.101412 | -0.06884 | 12.67826 | 3.05E-32 | 3.92E-30 | 62.36162 |
| HK3 | mRNA | UP | 1.101146 | 3.387272 | 9.941478 | 2.04E-21 | 1.12E-19 | 37.8103 |
| TNFAIP8L2 | mRNA | UP | 1.101089 | 2.403636 | 13.55481 | 5.29E-36 | 9.21E-34 | 70.9855 |
| COL6A1 | mRNA | UP | 1.101023 | 7.883571 | 12.29205 | 1.26E-30 | 1.44E-28 | 58.50328 |
| GDF6 | mRNA | UP | 1.101013 | -2.80162 | 6.919631 | 1.35E-11 | 2.71E-10 | 15.87502 |
| C16orf54 | mRNA | UP | 1.100749 | 2.34961 | 11.26638 | 1.83E-26 | 1.48E-24 | 49.30407 |
| SPI1 | mRNA | UP | 1.100606 | 5.0177 | 13.25072 | 1.10E-34 | 1.72E-32 | 67.95505 |
| PMP22 | mRNA | UP | 1.100484 | 5.943748 | 18.07144 | 7.10E-57 | 1.43E-53 | 118.6653 |
| PTGS1 | mRNA | UP | 1.10028 | 4.138375 | 13.79047 | 4.92E-37 | 9.70E-35 | 73.33875 |
| FMOD | mRNA | UP | 1.100239 | 5.846937 | 13.81559 | 3.81E-37 | 7.60E-35 | 73.54278 |
| PDGFRB | mRNA | UP | 1.100053 | 6.721469 | 17.35889 | 1.84E-53 | 2.29E-50 | 110.8406 |
| POU2AF1 | mRNA | UP | 1.097803 | 3.882988 | 7.653926 | 9.71E-14 | 2.50E-12 | 20.38988 |
| GLIPR1 | mRNA | UP | 1.097522 | 4.273643 | 16.32198 | 1.48E-48 | 1.16E-45 | 99.63398 |
| PODNL1 | mRNA | UP | 1.097259 | 3.103345 | 7.83782 | 2.66E-14 | 7.35E-13 | 21.71312 |
| CTHRC1 | mRNA | UP | 1.097138 | 5.567076 | 8.935073 | 7.28E-18 | 2.93E-16 | 29.60448 |
| CLEC7A | mRNA | UP | 1.097116 | 4.209694 | 11.76518 | 1.84E-28 | 1.73E-26 | 53.79674 |
| RYR2 | mRNA | UP | 1.096274 | 1.254696 | 9.012573 | 3.96E-18 | 1.63E-16 | 30.44516 |
| HLA-DPB1 | mRNA | UP | 1.096236 | 8.336155 | 11.31367 | 1.19E-26 | 9.80E-25 | 49.38298 |
| COL19A1 | mRNA | UP | 1.095871 | -2.93033 | 7.094108 | 4.34E-12 | 9.22E-11 | 16.97692 |
| TREM2 | mRNA | UP | 1.095464 | 4.090404 | 10.38563 | 4.58E-23 | 2.89E-21 | 41.51968 |
| GIMAP5 | mRNA | UP | 1.094946 | 0.791855 | 11.39082 | 5.86E-27 | 4.94E-25 | 50.42812 |
| C1S | mRNA | UP | 1.094264 | 8.222909 | 14.23933 | 5.07E-39 | 1.26E-36 | 77.68968 |
| CALB2 | mRNA | UP | 1.094142 | -0.24866 | 5.918728 | 5.93E-09 | 8.26E-08 | 9.916995 |
| NEXN | mRNA | UP | 1.093909 | 3.237109 | 14.9757 | 2.45E-42 | 8.36E-40 | 85.42423 |
| PTCRA | mRNA | UP | 1.093858 | -1.64571 | 9.07722 | 2.38E-18 | 1.00E-16 | 30.98351 |
| LRRN4 | mRNA | UP | 1.092972 | 2.824497 | 5.204122 | 2.82E-07 | 2.94E-06 | 5.983994 |
| CCL8 | mRNA | UP | 1.092414 | 1.260174 | 7.368957 | 6.91E-13 | 1.61E-11 | 18.63288 |
| FSTL1 | mRNA | UP | 1.09193 | 7.522976 | 15.32744 | 6.06E-44 | 2.49E-41 | 89.01192 |
| GPR124 | mRNA | UP | 1.091572 | 4.540357 | 16.03182 | 3.35E-47 | 2.17E-44 | 96.54186 |
| FCAR | mRNA | UP | 1.091349 | -1.5451 | 7.485085 | 3.13E-13 | 7.62E-12 | 19.50508 |
| PRKCB | mRNA | UP | 1.091257 | 3.688015 | 12.23109 | 2.26E-30 | 2.49E-28 | 58.16175 |
| ANGPT1 | mRNA | UP | 1.090724 | 2.856142 | 9.59778 | 3.56E-20 | 1.76E-18 | 35.01569 |
| C10orf55 | mRNA | UP | 1.090683 | -0.25395 | 8.456709 | 2.86E-16 | 9.93E-15 | 26.29424 |
| EGR2 | mRNA | UP | 1.090078 | 3.421667 | 12.169 | 4.09E-30 | 4.40E-28 | 57.58315 |
| GPNMB | mRNA | UP | 1.089758 | 7.690998 | 10.94408 | 3.35E-25 | 2.46E-23 | 46.13088 |
| ROS1 | mRNA | UP | 1.089239 | 5.157195 | 5.792775 | 1.21E-08 | 1.59E-07 | 8.829212 |
| GPR176 | mRNA | UP | 1.089101 | 3.126572 | 11.95719 | 3.03E-29 | 3.12E-27 | 55.60794 |
| HS3ST2 | mRNA | UP | 1.088087 | 2.133082 | 7.930187 | 1.37E-14 | 3.93E-13 | 22.41637 |
| CACNG6 | mRNA | UP | 1.087588 | -1.90647 | 4.447646 | 1.06E-05 | 8.30E-05 | 2.833413 |
| TIMD4 | mRNA | UP | 1.087422 | -1.00972 | 8.147677 | 2.84E-15 | 8.84E-14 | 24.06987 |
| SIRPA | mRNA | UP | 1.085602 | 5.567868 | 14.82745 | 1.15E-41 | 3.62E-39 | 83.88084 |
| CYSLTR2 | mRNA | UP | 1.085505 | 0.739856 | 13.10198 | 4.81E-34 | 7.14E-32 | 66.48511 |
| FGF2 | mRNA | UP | 1.085221 | 1.463491 | 11.1677 | 4.47E-26 | 3.52E-24 | 48.43003 |
| FOXF2 | mRNA | UP | 1.08461 | 2.365964 | 11.10531 | 7.87E-26 | 6.09E-24 | 47.86367 |
| FBXL7 | mRNA | UP | 1.084562 | 2.829906 | 15.57384 | 4.45E-45 | 2.11E-42 | 91.65473 |
| DNAJC5B | mRNA | UP | 1.084087 | 0.842845 | 9.828235 | 5.26E-21 | 2.79E-19 | 36.95506 |
| ART4 | mRNA | UP | 1.084086 | -0.4966 | 7.791673 | 3.69E-14 | 1.00E-12 | 21.56081 |
| CD200R1 | mRNA | UP | 1.084058 | 0.983245 | 12.61627 | 5.57E-32 | 6.95E-30 | 61.81346 |
| TMEM255A | mRNA | UP | 1.084024 | 1.066052 | 8.809197 | 1.94E-17 | 7.51E-16 | 28.89323 |
| SGIP1 | mRNA | UP | 1.08388 | 1.899491 | 15.14954 | 3.95E-43 | 1.42E-40 | 87.17465 |
| SLA | mRNA | UP | 1.083608 | 4.712483 | 14.18798 | 8.58E-39 | 2.11E-36 | 77.34078 |
| LILRB1 | mRNA | UP | 1.083544 | 3.204866 | 12.90573 | 3.32E-33 | 4.54E-31 | 64.62266 |
| LCP2 | mRNA | UP | 1.08347 | 5.066325 | 15.36809 | 3.94E-44 | 1.67E-41 | 89.52672 |
| ANKRD33B | mRNA | UP | 1.083253 | 1.442382 | 10.2264 | 1.81E-22 | 1.09E-20 | 40.25827 |
| CD27 | mRNA | UP | 1.082881 | 2.804762 | 10.08409 | 6.09E-22 | 3.49E-20 | 39.02387 |
| LILRA1 | mRNA | UP | 1.082612 | -0.03138 | 12.57763 | 8.10E-32 | 9.94E-30 | 61.40452 |
| PTGFR | mRNA | UP | 1.0824 | 0.237564 | 6.832888 | 2.36E-11 | 4.59E-10 | 15.24453 |
| ATP8B4 | mRNA | UP | 1.081685 | 1.913217 | 14.96356 | 2.78E-42 | 9.28E-40 | 85.24833 |
| CD48 | mRNA | UP | 1.080817 | 4.010516 | 10.59383 | 7.46E-24 | 5.04E-22 | 43.31568 |
| MN1 | mRNA | UP | 1.080056 | 2.579324 | 11.05409 | 1.25E-25 | 9.61E-24 | 47.40385 |
| RCSD1 | mRNA | UP | 1.079947 | 3.981633 | 14.75609 | 2.43E-41 | 7.26E-39 | 83.16284 |
| P2RX1 | mRNA | UP | 1.079873 | 1.480525 | 11.38693 | 6.07E-27 | 5.11E-25 | 50.39673 |
| HLA-DQB1 | mRNA | UP | 1.07905 | 7.802975 | 9.008511 | 4.09E-18 | 1.68E-16 | 29.96996 |
| GLI1 | mRNA | UP | 1.078938 | 0.489148 | 9.182552 | 1.03E-18 | 4.49E-17 | 31.78648 |
| KCNA2 | mRNA | UP | 1.0788 | -2.75678 | 7.971431 | 1.02E-14 | 2.97E-13 | 22.84109 |
| HLA-DMB | mRNA | UP | 1.078402 | 6.216289 | 11.70582 | 3.19E-28 | 2.94E-26 | 53.14283 |
| NTNG1 | mRNA | UP | 1.07803 | -0.57095 | 5.422215 | 9.07E-08 | 1.03E-06 | 7.309331 |
| SGCG | mRNA | UP | 1.077976 | -2.19563 | 7.754154 | 4.81E-14 | 1.29E-12 | 21.3333 |
| SLITRK3 | mRNA | UP | 1.077643 | -3.30527 | 6.112227 | 1.94E-09 | 2.92E-08 | 11.09216 |
| ARHGEF6 | mRNA | UP | 1.07671 | 4.372848 | 16.77461 | 1.09E-50 | 1.08E-47 | 104.4992 |
| SEMA5A | mRNA | UP | 1.075314 | 3.869189 | 10.29847 | 9.73E-23 | 5.99E-21 | 40.78727 |
| GLYATL2 | mRNA | UP | 1.075108 | -3.07671 | 4.33239 | 1.77E-05 | 0.000132 | 2.394794 |
| NLRP12 | mRNA | UP | 1.075077 | 0.141039 | 7.229834 | 1.76E-12 | 3.92E-11 | 17.77279 |
| FEZ1 | mRNA | UP | 1.074736 | 2.523077 | 16.19209 | 5.98E-48 | 4.31E-45 | 98.17802 |
| ATP6V0D2 | mRNA | UP | 1.074481 | 0.939381 | 7.688993 | 7.60E-14 | 1.98E-12 | 20.80299 |
| TIGIT | mRNA | UP | 1.074028 | 2.723938 | 10.32178 | 7.96E-23 | 4.94E-21 | 41.03276 |
| TCEAL7 | mRNA | UP | 1.073555 | 0.115651 | 13.6034 | 3.24E-36 | 5.77E-34 | 71.36782 |
| CADPS | mRNA | UP | 1.073408 | -1.25795 | 6.906847 | 1.47E-11 | 2.93E-10 | 15.76256 |
| KCNJ5 | mRNA | UP | 1.073307 | 2.197202 | 8.457092 | 2.86E-16 | 9.91E-15 | 26.20949 |
| SULF2 | mRNA | UP | 1.072871 | 5.395958 | 12.43802 | 3.12E-31 | 3.71E-29 | 60.06115 |
| FFAR4 | mRNA | UP | 1.072829 | 0.661187 | 7.941062 | 1.27E-14 | 3.65E-13 | 22.56175 |
| ABCA9 | mRNA | UP | 1.072802 | 1.147171 | 11.57382 | 1.09E-27 | 9.70E-26 | 52.08952 |
| MEOX1 | mRNA | UP | 1.07239 | -0.53241 | 8.447619 | 3.07E-16 | 1.06E-14 | 26.23278 |
| LTA | mRNA | UP | 1.072231 | -0.18691 | 10.63888 | 5.02E-24 | 3.45E-22 | 43.78132 |
| ILDR2 | mRNA | UP | 1.071902 | 0.335233 | 8.443349 | 3.17E-16 | 1.09E-14 | 26.18148 |
| DAB2 | mRNA | UP | 1.071788 | 5.829098 | 16.43762 | 4.23E-49 | 3.48E-46 | 100.8771 |
| GIMAP4 | mRNA | UP | 1.071611 | 4.658219 | 15.93932 | 9.03E-47 | 5.22E-44 | 95.55924 |
| SPARCL1 | mRNA | UP | 1.071608 | 7.000253 | 13.87737 | 2.04E-37 | 4.19E-35 | 74.10388 |
| LPPR5 | mRNA | UP | 1.071578 | -4.5363 | 7.451414 | 3.94E-13 | 9.49E-12 | 19.29774 |
| CH25H | mRNA | UP | 1.071129 | 2.673023 | 7.372132 | 6.76E-13 | 1.58E-11 | 18.5718 |
| LRRC18 | mRNA | UP | 1.070624 | -2.34882 | 5.903839 | 6.46E-09 | 8.92E-08 | 9.918265 |
| TYROBP | mRNA | UP | 1.070612 | 5.903455 | 12.09297 | 8.41E-30 | 8.93E-28 | 56.76744 |
| PLD4 | mRNA | UP | 1.070192 | 1.978944 | 9.200002 | 8.95E-19 | 3.95E-17 | 31.87927 |
| NUGGC | mRNA | UP | 1.07013 | 0.654709 | 8.792161 | 2.21E-17 | 8.52E-16 | 28.77641 |
| SCARF2 | mRNA | UP | 1.069756 | 3.296693 | 13.42068 | 2.03E-35 | 3.36E-33 | 69.6641 |
| EDNRA | mRNA | UP | 1.069587 | 4.39437 | 13.03086 | 9.69E-34 | 1.38E-31 | 65.81912 |
| APOC2 | mRNA | UP | 1.068908 | -2.39523 | 8.248281 | 1.36E-15 | 4.34E-14 | 24.80241 |
| ST6GAL2 | mRNA | UP | 1.068899 | -0.15499 | 6.1672 | 1.41E-09 | 2.16E-08 | 11.30128 |
| TNFRSF13B | mRNA | UP | 1.06817 | -0.34874 | 7.031224 | 6.55E-12 | 1.36E-10 | 16.51566 |
| CRHBP | mRNA | UP | 1.067858 | -2.4997 | 9.723358 | 1.26E-20 | 6.45E-19 | 36.08529 |
| GNG2 | mRNA | UP | 1.067217 | 3.817575 | 18.94844 | 4.07E-61 | 1.32E-57 | 128.28 |
| TMEM47 | mRNA | UP | 1.067147 | 3.907216 | 14.41438 | 8.37E-40 | 2.25E-37 | 79.65469 |
| PPAPDC3 | mRNA | UP | 1.067113 | -0.11965 | 14.96753 | 2.67E-42 | 9.00E-40 | 85.12369 |
| ZFPM2 | mRNA | UP | 1.066485 | 2.185133 | 14.8004 | 1.53E-41 | 4.70E-39 | 83.57822 |
| TGFB3 | mRNA | UP | 1.065443 | 4.261959 | 16.48224 | 2.61E-49 | 2.28E-46 | 101.3509 |
| CHRNA1 | mRNA | UP | 1.065306 | -0.20145 | 6.836756 | 2.30E-11 | 4.48E-10 | 15.28833 |
| CD3G | mRNA | UP | 1.065193 | 2.291467 | 10.76457 | 1.66E-24 | 1.18E-22 | 44.86101 |
| GRIP2 | mRNA | UP | 1.064329 | -0.66298 | 9.470588 | 1.01E-19 | 4.78E-18 | 34.07275 |
| CD14 | mRNA | UP | 1.063958 | 6.281314 | 12.31931 | 9.74E-31 | 1.12E-28 | 58.88184 |
| ISLR2 | mRNA | UP | 1.063892 | 0.421017 | 9.062365 | 2.67E-18 | 1.12E-16 | 30.85253 |
| CD300E | mRNA | UP | 1.063418 | 1.395591 | 9.308628 | 3.75E-19 | 1.70E-17 | 32.75522 |
| CYSLTR1 | mRNA | UP | 1.063411 | 2.391707 | 10.32542 | 7.71E-23 | 4.79E-21 | 41.07417 |
| MMP19 | mRNA | UP | 1.062965 | 3.724736 | 11.95769 | 3.02E-29 | 3.11E-27 | 55.59824 |
| DOCK10 | mRNA | UP | 1.062643 | 4.344475 | 13.77281 | 5.88E-37 | 1.15E-34 | 73.1587 |
| CST6 | mRNA | UP | 1.062234 | 2.44356 | 5.783092 | 1.27E-08 | 1.68E-07 | 9.003547 |
| ST8SIA2 | mRNA | UP | 1.062115 | -2.63223 | 5.554509 | 4.47E-08 | 5.38E-07 | 8.069371 |
| CHSY3 | mRNA | UP | 1.061884 | 1.675895 | 11.45567 | 3.23E-27 | 2.80E-25 | 51.01793 |
| TCF4 | mRNA | UP | 1.06079 | 5.479695 | 17.8841 | 5.65E-56 | 1.00E-52 | 116.6014 |
| RASSF2 | mRNA | UP | 1.060085 | 4.864918 | 15.22865 | 1.72E-43 | 6.55E-41 | 88.0683 |
| NCF2 | mRNA | UP | 1.059157 | 5.069843 | 12.7993 | 9.39E-33 | 1.27E-30 | 63.54636 |
| HEG1 | mRNA | UP | 1.058719 | 5.917439 | 14.90669 | 5.04E-42 | 1.64E-39 | 84.6917 |
| CD52 | mRNA | UP | 1.058375 | 4.864918 | 10.74218 | 2.02E-24 | 1.43E-22 | 44.56233 |
| FCER1G | mRNA | UP | 1.058281 | 5.403529 | 12.64357 | 4.28E-32 | 5.43E-30 | 62.02934 |
| FGF1 | mRNA | UP | 1.057199 | 1.682505 | 10.30803 | 8.96E-23 | 5.54E-21 | 40.94415 |
| EOMES | mRNA | UP | 1.056706 | 0.786432 | 9.179432 | 1.06E-18 | 4.60E-17 | 31.75553 |
| PIK3R5 | mRNA | UP | 1.056171 | 3.55936 | 14.07724 | 2.66E-38 | 6.12E-36 | 76.22831 |
| UNC45B | mRNA | UP | 1.055369 | -2.32073 | 9.387638 | 1.98E-19 | 9.15E-18 | 33.40323 |
| CD93 | mRNA | UP | 1.055079 | 6.145001 | 14.80473 | 1.46E-41 | 4.52E-39 | 83.62628 |
| GP5 | mRNA | UP | 1.055051 | -2.67977 | 9.201902 | 8.82E-19 | 3.89E-17 | 31.94277 |
| KAL1 | mRNA | UP | 1.054288 | 4.431388 | 9.311915 | 3.65E-19 | 1.66E-17 | 32.63733 |
| SIRPD | mRNA | UP | 1.053966 | -3.63286 | 9.023521 | 3.63E-18 | 1.51E-16 | 30.55727 |
| PCDHGB7 | mRNA | UP | 1.053777 | 1.416154 | 11.87209 | 6.75E-29 | 6.67E-27 | 54.82832 |
| CHRNA6 | mRNA | UP | 1.053507 | -2.45022 | 9.287593 | 4.44E-19 | 2.00E-17 | 32.61451 |
| FUT7 | mRNA | UP | 1.052963 | -0.40182 | 9.76136 | 9.19E-21 | 4.78E-19 | 36.41675 |
| LSP1 | mRNA | UP | 1.051939 | 5.973726 | 13.63552 | 2.35E-36 | 4.31E-34 | 71.73256 |
| DMBT1 | mRNA | UP | 1.051759 | 5.096665 | 3.748848 | 0.000198 | 0.001167 | -0.46726 |
| PCOLCE | mRNA | UP | 1.051531 | 5.877397 | 12.73973 | 1.68E-32 | 2.19E-30 | 62.9323 |
| HLA-DRB1 | mRNA | UP | 1.051111 | 9.28988 | 9.886674 | 3.23E-21 | 1.74E-19 | 36.8763 |
| ALOX5 | mRNA | UP | 1.051069 | 5.268287 | 11.66983 | 4.46E-28 | 4.06E-26 | 52.86884 |
| GATA5 | mRNA | UP | 1.05077 | -0.87371 | 7.442818 | 4.18E-13 | 1.00E-11 | 19.2083 |
| ITGB3 | mRNA | UP | 1.050468 | 1.647423 | 7.804593 | 3.36E-14 | 9.19E-13 | 21.56514 |
| COL16A1 | mRNA | UP | 1.050113 | 5.395229 | 13.03921 | 8.93E-34 | 1.28E-31 | 65.86554 |
| SUGCT | mRNA | UP | 1.04968 | 1.504197 | 9.000761 | 4.35E-18 | 1.78E-16 | 30.34541 |
| SSTR3 | mRNA | UP | 1.048298 | -2.37785 | 8.28041 | 1.07E-15 | 3.47E-14 | 25.03351 |
| HTRA1 | mRNA | UP | 1.048266 | 6.177572 | 14.15871 | 1.16E-38 | 2.77E-36 | 76.99604 |
| TMEM158 | mRNA | UP | 1.047361 | 1.286736 | 8.116731 | 3.56E-15 | 1.09E-13 | 23.7795 |
| LAYN | mRNA | UP | 1.047164 | 2.869572 | 12.71919 | 2.05E-32 | 2.66E-30 | 62.82333 |
| LRMP | mRNA | UP | 1.047116 | 2.58112 | 10.94534 | 3.31E-25 | 2.44E-23 | 46.44113 |
| FREM1 | mRNA | UP | 1.04659 | 1.021601 | 7.210241 | 2.01E-12 | 4.44E-11 | 17.60393 |
| BCL2A1 | mRNA | UP | 1.045139 | 2.892457 | 10.66185 | 4.10E-24 | 2.84E-22 | 43.95014 |
| PNMAL1 | mRNA | UP | 1.045025 | 2.577397 | 6.06896 | 2.50E-09 | 3.69E-08 | 10.57168 |
| TPSD1 | mRNA | UP | 1.044994 | -0.29718 | 4.600366 | 5.32E-06 | 4.39E-05 | 3.404501 |
| KBTBD8 | mRNA | UP | 1.044652 | 1.430319 | 13.86989 | 2.20E-37 | 4.49E-35 | 74.09446 |
| KIAA1755 | mRNA | UP | 1.044561 | 1.898787 | 13.97783 | 7.34E-38 | 1.58E-35 | 75.19568 |
| APLNR | mRNA | UP | 1.044516 | 3.338124 | 11.03643 | 1.46E-25 | 1.12E-23 | 47.22561 |
| LRRC55 | mRNA | UP | 1.044288 | -1.08049 | 6.223317 | 1.01E-09 | 1.59E-08 | 11.66275 |
| BIN2 | mRNA | UP | 1.043543 | 3.558206 | 13.83383 | 3.17E-37 | 6.38E-35 | 73.77761 |
| ANGPTL7 | mRNA | UP | 1.042999 | -3.61537 | 7.003239 | 7.86E-12 | 1.62E-10 | 16.40682 |
| WIPF1 | mRNA | UP | 1.042727 | 5.947554 | 18.25957 | 8.80E-58 | 2.06E-54 | 120.7401 |
| PLD5 | mRNA | UP | 1.040883 | 0.622043 | 4.385044 | 1.41E-05 | 0.000107 | 2.418016 |
| SERPINE1 | mRNA | UP | 1.040478 | 6.052162 | 8.253657 | 1.30E-15 | 4.19E-14 | 24.45282 |
| TMEM52B | mRNA | UP | 1.040283 | 0.03244 | 10.97996 | 2.43E-25 | 1.82E-23 | 46.75708 |
| SERPING1 | mRNA | UP | 1.039579 | 8.021584 | 16.24897 | 3.24E-48 | 2.46E-45 | 98.76747 |
| ITGAL | mRNA | UP | 1.038924 | 4.915919 | 11.66468 | 4.68E-28 | 4.24E-26 | 52.83976 |
| SLC9A9 | mRNA | UP | 1.038381 | 2.781706 | 14.96562 | 2.72E-42 | 9.13E-40 | 85.30733 |
| PYHIN1 | mRNA | UP | 1.038286 | 1.005574 | 9.978105 | 1.50E-21 | 8.30E-20 | 38.18876 |
| KCNMA1 | mRNA | UP | 1.037901 | 3.306054 | 10.04854 | 8.24E-22 | 4.68E-20 | 38.70588 |
| PAX5 | mRNA | UP | 1.0379 | 1.361435 | 6.228538 | 9.80E-10 | 1.54E-08 | 11.5643 |
| PTGIR | mRNA | UP | 1.037627 | 2.079002 | 16.67322 | 3.29E-50 | 3.10E-47 | 103.2871 |
| STON1-GTF2A1L | mRNA | UP | 1.036795 | -4.08102 | 8.23197 | 1.53E-15 | 4.87E-14 | 24.6859 |
| CD200 | mRNA | UP | 1.035964 | 2.988172 | 16.9253 | 2.12E-51 | 2.21E-48 | 106.0662 |
| ZEB1 | mRNA | UP | 1.035689 | 4.239452 | 18.70491 | 6.18E-60 | 1.80E-56 | 125.6108 |
| CD207 | mRNA | UP | 1.035464 | 1.486999 | 4.371666 | 1.49E-05 | 0.000113 | 2.295533 |
| FGD2 | mRNA | UP | 1.035128 | 3.607881 | 12.54148 | 1.15E-31 | 1.39E-29 | 61.11045 |
| KCNQ5 | mRNA | UP | 1.034526 | 1.118138 | 5.984422 | 4.08E-09 | 5.84E-08 | 10.19935 |
| RGS1 | mRNA | UP | 1.033927 | 6.035089 | 9.417699 | 1.55E-19 | 7.25E-18 | 33.3648 |
| MYH13 | mRNA | UP | 1.033904 | -3.62824 | 7.020846 | 7.01E-12 | 1.45E-10 | 16.5179 |
| MILR1 | mRNA | UP | 1.033904 | 2.487554 | 11.3312 | 1.01E-26 | 8.37E-25 | 49.88477 |
| COL5A3 | mRNA | UP | 1.033681 | 3.77871 | 9.812847 | 5.99E-21 | 3.16E-19 | 36.72752 |
| CTLA4 | mRNA | UP | 1.033545 | 1.586602 | 9.875444 | 3.55E-21 | 1.90E-19 | 37.32832 |
| SRPX | mRNA | UP | 1.033157 | 2.521063 | 10.58536 | 8.04E-24 | 5.41E-22 | 43.2986 |
| LAT2 | mRNA | UP | 1.03306 | 4.024713 | 14.31347 | 2.37E-39 | 6.14E-37 | 78.62506 |
| C5AR1 | mRNA | UP | 1.032886 | 4.330984 | 12.31625 | 1.00E-30 | 1.15E-28 | 58.94786 |
| DKK2 | mRNA | UP | 1.032731 | 1.483878 | 9.677201 | 1.85E-20 | 9.37E-19 | 35.70826 |
| ADCYAP1R1 | mRNA | UP | 1.032696 | -1.16959 | 8.359521 | 5.93E-16 | 1.98E-14 | 25.59823 |
| KLHL33 | mRNA | UP | 1.032421 | -3.76466 | 8.816194 | 1.84E-17 | 7.13E-16 | 28.98061 |
| GLIS1 | mRNA | UP | 1.031879 | -2.19285 | 7.5506 | 1.99E-13 | 4.97E-12 | 19.95344 |
| P2RY8 | mRNA | UP | 1.031682 | 1.807055 | 11.54608 | 1.40E-27 | 1.24E-25 | 51.83851 |
| PRG4 | mRNA | UP | 1.030722 | 1.483378 | 5.430055 | 8.70E-08 | 9.96E-07 | 7.218677 |
| RECK | mRNA | UP | 1.030667 | 3.166682 | 17.91007 | 4.24E-56 | 7.99E-53 | 116.7872 |
| CLEC4E | mRNA | UP | 1.030595 | 1.21527 | 8.473777 | 2.52E-16 | 8.80E-15 | 26.37582 |
| SIGLEC11 | mRNA | UP | 1.030389 | -0.62035 | 8.589848 | 1.05E-16 | 3.80E-15 | 27.28467 |
| IL4I1 | mRNA | UP | 1.030304 | 3.610117 | 8.85587 | 1.35E-17 | 5.29E-16 | 29.13125 |
| S100Z | mRNA | UP | 1.028661 | -2.45982 | 9.589637 | 3.80E-20 | 1.87E-18 | 35.00888 |
| CD300LF | mRNA | UP | 1.028587 | 2.706478 | 10.40846 | 3.76E-23 | 2.39E-21 | 41.7724 |
| PRSS35 | mRNA | UP | 1.028114 | -1.38834 | 8.110708 | 3.72E-15 | 1.14E-13 | 23.81212 |
| TXLNB | mRNA | UP | 1.027899 | 0.407381 | 9.878501 | 3.46E-21 | 1.86E-19 | 37.37309 |
| PRR5L | mRNA | UP | 1.027786 | 3.5356 | 12.40697 | 4.20E-31 | 4.99E-29 | 59.83005 |
| PLAT | mRNA | UP | 1.027627 | 5.991512 | 6.771464 | 3.49E-11 | 6.66E-10 | 14.44791 |
| DPEP2 | mRNA | UP | 1.02749 | 2.01754 | 11.8149 | 1.15E-28 | 1.11E-26 | 54.30166 |
| CCDC8 | mRNA | UP | 1.026703 | 2.614687 | 10.49313 | 1.80E-23 | 1.18E-21 | 42.50105 |
| ASXL3 | mRNA | UP | 1.026374 | -0.72326 | 7.730345 | 5.68E-14 | 1.51E-12 | 21.14509 |
| CRISPLD2 | mRNA | UP | 1.025886 | 5.534109 | 14.54955 | 2.07E-40 | 5.87E-38 | 81.01496 |
| C19orf35 | mRNA | UP | 1.025353 | -0.65406 | 12.76103 | 1.36E-32 | 1.81E-30 | 63.11944 |
| EGR3 | mRNA | UP | 1.02503 | 2.514271 | 9.455638 | 1.14E-19 | 5.38E-18 | 33.88526 |
| MFAP2 | mRNA | UP | 1.024854 | 4.430138 | 10.47192 | 2.17E-23 | 1.40E-21 | 42.24191 |
| FBN2 | mRNA | UP | 1.024212 | 0.39697 | 6.295503 | 6.58E-10 | 1.06E-08 | 12.00903 |
| IL1A | mRNA | UP | 1.023888 | -0.68811 | 6.090306 | 2.21E-09 | 3.29E-08 | 10.89167 |
| GYPC | mRNA | UP | 1.022611 | 3.988339 | 15.98775 | 5.37E-47 | 3.34E-44 | 96.06511 |
| HS3ST3B1 | mRNA | UP | 1.022342 | 2.06738 | 12.63537 | 4.63E-32 | 5.84E-30 | 62.0173 |
| ANXA8L1 | mRNA | UP | 1.022272 | -0.91644 | 5.392148 | 1.06E-07 | 1.20E-06 | 7.174561 |
| RASGRF2 | mRNA | UP | 1.021964 | 3.006893 | 13.82393 | 3.50E-37 | 7.00E-35 | 73.6762 |
| CD2 | mRNA | UP | 1.02186 | 3.955194 | 10.37049 | 5.22E-23 | 3.28E-21 | 41.39684 |
| ZFP57 | mRNA | UP | 1.021791 | -1.46794 | 4.565978 | 6.23E-06 | 5.07E-05 | 3.3202 |
| ROBO2 | mRNA | UP | 1.021359 | 1.8893 | 6.50249 | 1.87E-10 | 3.25E-09 | 13.13642 |
| RGS22 | mRNA | UP | 1.021064 | -0.16823 | 6.699372 | 5.51E-11 | 1.03E-09 | 14.44104 |
| ZNF469 | mRNA | UP | 1.020894 | 2.720663 | 11.04906 | 1.31E-25 | 1.00E-23 | 47.35576 |
| FGD5 | mRNA | UP | 1.020828 | 4.114007 | 16.12721 | 1.20E-47 | 8.36E-45 | 97.55141 |
| ROR1 | mRNA | UP | 1.020621 | 2.727517 | 10.21106 | 2.06E-22 | 1.24E-20 | 40.09429 |
| CD5 | mRNA | UP | 1.020396 | 3.032488 | 10.41534 | 3.54E-23 | 2.26E-21 | 41.81987 |
| ADAMTS8 | mRNA | UP | 1.020059 | 1.144494 | 6.094816 | 2.15E-09 | 3.21E-08 | 10.81593 |
| THSD7B | mRNA | UP | 1.020039 | -0.68129 | 7.06682 | 5.19E-12 | 1.09E-10 | 16.75419 |
| CDH23 | mRNA | UP | 1.019443 | 1.949241 | 10.56091 | 9.96E-24 | 6.64E-22 | 43.10136 |
| VNN2 | mRNA | UP | 1.018264 | 1.980139 | 8.889085 | 1.04E-17 | 4.14E-16 | 29.46813 |
| PCDHGC3 | mRNA | UP | 1.018221 | 3.507547 | 11.57917 | 1.03E-27 | 9.25E-26 | 52.1115 |
| FOXI2 | mRNA | UP | 1.018135 | -3.83062 | 6.281116 | 7.17E-10 | 1.15E-08 | 12.05604 |
| NTF3 | mRNA | UP | 1.017926 | -1.26655 | 8.012902 | 7.57E-15 | 2.24E-13 | 23.11872 |
| CD226 | mRNA | UP | 1.017626 | 1.850206 | 11.86437 | 7.26E-29 | 7.13E-27 | 54.75891 |
| C20orf85 | mRNA | UP | 1.01735 | -0.25449 | 3.24816 | 0.001237 | 0.005935 | -1.7007 |
| FERMT3 | mRNA | UP | 1.017223 | 5.014987 | 13.65757 | 1.88E-36 | 3.47E-34 | 71.98993 |
| MEI1 | mRNA | UP | 1.016802 | 2.433365 | 9.961693 | 1.72E-21 | 9.48E-20 | 38.01661 |
| LOXL1 | mRNA | UP | 1.016536 | 4.504624 | 12.75821 | 1.40E-32 | 1.85E-30 | 63.16948 |
| MGP | mRNA | UP | 1.016395 | 7.257195 | 10.22879 | 1.77E-22 | 1.07E-20 | 39.95834 |
| TRIM61 | mRNA | UP | 1.016386 | -1.95019 | 7.902553 | 1.67E-14 | 4.74E-13 | 22.35519 |
| ARHGAP31 | mRNA | UP | 1.01627 | 4.917871 | 13.40788 | 2.30E-35 | 3.79E-33 | 69.50983 |
| SLITRK4 | mRNA | UP | 1.015962 | -0.50012 | 6.582535 | 1.14E-10 | 2.05E-09 | 13.74757 |
| NKD2 | mRNA | UP | 1.015577 | 2.942719 | 9.690511 | 1.66E-20 | 8.43E-19 | 35.76527 |
| TRAF3IP3 | mRNA | UP | 1.01501 | 2.949594 | 11.53626 | 1.54E-27 | 1.36E-25 | 51.73419 |
| XYLT1 | mRNA | UP | 1.01477 | 3.77754 | 10.65092 | 4.52E-24 | 3.11E-22 | 43.82154 |
| ZNF80 | mRNA | UP | 1.014362 | -2.74477 | 7.09962 | 4.18E-12 | 8.92E-11 | 17.00995 |
| FLNC | mRNA | UP | 1.014333 | 3.613549 | 6.804164 | 2.84E-11 | 5.47E-10 | 14.85468 |
| RAI2 | mRNA | UP | 1.014012 | 2.943265 | 12.63861 | 4.49E-32 | 5.67E-30 | 62.04914 |
| PTX3 | mRNA | UP | 1.013868 | 0.452578 | 8.490631 | 2.22E-16 | 7.79E-15 | 26.52632 |
| TMEM150B | mRNA | UP | 1.013608 | 1.21623 | 10.01846 | 1.06E-21 | 5.99E-20 | 38.5204 |
| CCL2 | mRNA | UP | 1.013573 | 5.036655 | 10.32406 | 7.80E-23 | 4.85E-21 | 40.9399 |
| ZFR2 | mRNA | UP | 1.013465 | -2.1639 | 4.58118 | 5.81E-06 | 4.76E-05 | 3.417298 |
| GPR78 | mRNA | UP | 1.012798 | -2.16605 | 7.033385 | 6.46E-12 | 1.34E-10 | 16.58008 |
| ENPP6 | mRNA | UP | 1.012664 | -1.92123 | 9.62184 | 2.92E-20 | 1.46E-18 | 35.27507 |
| SLC7A7 | mRNA | UP | 1.011892 | 4.882571 | 13.38666 | 2.84E-35 | 4.63E-33 | 69.30096 |
| DCHS2 | mRNA | UP | 1.010883 | -1.04148 | 6.14778 | 1.58E-09 | 2.40E-08 | 11.23104 |
| C1R | mRNA | UP | 1.010388 | 7.956906 | 15.86309 | 2.04E-46 | 1.15E-43 | 94.64929 |
| C11orf88 | mRNA | UP | 1.010333 | -1.10027 | 4.651378 | 4.20E-06 | 3.54E-05 | 3.675422 |
| LILRA5 | mRNA | UP | 1.010296 | 1.40491 | 9.860934 | 4.00E-21 | 2.14E-19 | 37.21309 |
| LILRA6 | mRNA | UP | 1.009842 | 1.526034 | 10.29408 | 1.01E-22 | 6.20E-21 | 40.82885 |
| FLI1 | mRNA | UP | 1.009659 | 4.173689 | 16.4648 | 3.15E-49 | 2.67E-46 | 101.1618 |
| CRLF2 | mRNA | UP | 1.009646 | -2.20998 | 6.042942 | 2.91E-09 | 4.26E-08 | 10.68154 |
| TRPV2 | mRNA | UP | 1.008937 | 4.398231 | 14.98497 | 2.22E-42 | 7.68E-40 | 85.53193 |
| MEGF10 | mRNA | UP | 1.008609 | -1.17277 | 5.805355 | 1.13E-08 | 1.49E-07 | 9.343518 |
| IL12RB1 | mRNA | UP | 1.008429 | 2.354867 | 11.95028 | 3.24E-29 | 3.32E-27 | 55.55365 |
| AOX1 | mRNA | UP | 1.008109 | 2.426361 | 8.088402 | 4.38E-15 | 1.33E-13 | 23.52064 |
| SPP1 | mRNA | UP | 1.008082 | 8.062684 | 5.539119 | 4.86E-08 | 5.81E-07 | 7.172004 |
| KIRREL | mRNA | UP | 1.00794 | 5.24904 | 13.41121 | 2.23E-35 | 3.67E-33 | 69.53147 |
| INMT | mRNA | UP | 1.007336 | 3.797954 | 6.975167 | 9.44E-12 | 1.93E-10 | 15.91533 |
| ALDH1L2 | mRNA | UP | 1.006859 | 3.145391 | 9.532657 | 6.07E-20 | 2.94E-18 | 34.47607 |
| CALD1 | mRNA | UP | 1.006474 | 7.240538 | 15.23443 | 1.62E-43 | 6.21E-41 | 88.05177 |
| SLC2A9 | mRNA | UP | 1.006101 | 1.898322 | 15.84149 | 2.57E-46 | 1.42E-43 | 94.42069 |
| MEIS3 | mRNA | UP | 1.005868 | 2.215894 | 12.6428 | 4.31E-32 | 5.46E-30 | 62.08958 |
| MYBPC2 | mRNA | UP | 1.004139 | -0.13847 | 7.045195 | 5.98E-12 | 1.25E-10 | 16.59614 |
| ARHGAP15 | mRNA | UP | 1.004135 | 2.946776 | 13.74434 | 7.84E-37 | 1.50E-34 | 72.87942 |
| GIMAP8 | mRNA | UP | 1.003837 | 3.696533 | 13.6844 | 1.43E-36 | 2.68E-34 | 72.28199 |
| DHRS9 | mRNA | UP | 1.003821 | 2.332067 | 6.529451 | 1.59E-10 | 2.78E-09 | 13.26828 |
| ZBED2 | mRNA | UP | 1.003678 | 1.412989 | 7.713837 | 6.38E-14 | 1.69E-12 | 20.95062 |
| CACNG4 | mRNA | UP | 1.003571 | 2.30784 | 4.239737 | 2.65E-05 | 0.00019 | 1.683316 |
| P2RY14 | mRNA | UP | 1.003025 | 1.513262 | 12.09669 | 8.12E-30 | 8.63E-28 | 56.91648 |
| PM20D1 | mRNA | UP | 1.003011 | -2.22634 | 8.583864 | 1.09E-16 | 3.96E-15 | 27.25022 |
| TM4SF18 | mRNA | UP | 1.002683 | 2.687171 | 10.03162 | 9.51E-22 | 5.37E-20 | 38.58951 |
| IL1B | mRNA | UP | 1.001867 | 2.396903 | 8.515776 | 1.83E-16 | 6.48E-15 | 26.63375 |
| ACKR1 | mRNA | UP | 1.001782 | 2.727079 | 5.961074 | 4.66E-09 | 6.60E-08 | 9.957035 |
| C1orf162 | mRNA | UP | 1.001709 | 4.020011 | 13.05038 | 8.00E-34 | 1.16E-31 | 66.01833 |
| RAB44 | mRNA | UP | 1.001682 | -1.53258 | 8.358895 | 5.96E-16 | 1.99E-14 | 25.59721 |
| GPR110 | mRNA | UP | 1.000977 | 4.50636 | 3.937204 | 9.38E-05 | 0.000598 | 0.292809 |
| CAMK1G | mRNA | UP | 1.000968 | -0.17492 | 13.73159 | 8.91E-37 | 1.70E-34 | 72.61668 |
| LYVE1 | mRNA | UP | 1.000855 | 2.225187 | 9.090911 | 2.13E-18 | 9.03E-17 | 31.01634 |
