# Supplementary material for: Screening of T Cell-Related Long Noncoding RNA-MicroRNA-mRNA Regulatory Networks in Non-Small-Cell Lung Cancer
Source: Biomed Res Int. 2020 Nov 14;2020:5816763. doi: 10.1155/2020/5816763 (PMC7684158; doi:10.1155/2020/5816763)
Supplement: Supplementary 2 — Supplementary Table 2: genes sorted by Immune score. [file 5816763.f2.docx]

Supplementary Table 2 Genes sorted by Immune score.

| gene | type | change | logFC | AveExpr | t | P.Value | adj.P.Val | B |
| --- | --- | --- | --- | --- | --- | --- | --- | --- |
| DSCAM-AS1 | lncRNA | DOWN | -2.0099 | -3.19384 | -4.93104 | 1.11E-06 | 1.28E-05 | 4.756227 |
| LINC00473 | lncRNA | DOWN | -1.90003 | -2.43371 | -5.33522 | 1.43E-07 | 1.95E-06 | 6.698325 |
| LINC00676 | lncRNA | DOWN | -1.62193 | -4.45313 | -4.99152 | 8.22E-07 | 9.77E-06 | 4.998555 |
| RP11-116O18.1 | lncRNA | DOWN | -1.45515 | -2.47535 | -4.41362 | 1.24E-05 | 0.000115 | 2.432696 |
| RP11-148B3.2 | lncRNA | DOWN | -1.41532 | -3.28114 | -5.63539 | 2.88E-08 | 4.36E-07 | 8.273487 |
| NOVA1-AS1 | lncRNA | DOWN | -1.38352 | -3.42174 | -4.88514 | 1.38E-06 | 1.57E-05 | 4.554525 |
| CASC9 | lncRNA | DOWN | -1.36124 | -1.24871 | -4.0357 | 6.27E-05 | 0.000492 | 0.831851 |
| RP11-169F17.1 | lncRNA | DOWN | -1.34229 | -0.489 | -3.68344 | 0.000254 | 0.001701 | -0.53555 |
| RP11-279F6.1 | lncRNA | DOWN | -1.25263 | 0.720504 | -4.95957 | 9.62E-07 | 1.13E-05 | 4.672638 |
| RP11-474D1.3 | lncRNA | DOWN | -1.25029 | -3.70359 | -3.82329 | 0.000148 | 0.001052 | 0.128782 |
| PART1 | lncRNA | DOWN | -1.23687 | -2.86034 | -4.87947 | 1.42E-06 | 1.61E-05 | 4.514551 |
| H19 | lncRNA | DOWN | -1.13582 | 3.058942 | -4.77144 | 2.39E-06 | 2.58E-05 | 3.598253 |
| CTD-2021H9.3 | lncRNA | DOWN | -1.12732 | -3.20299 | -4.08497 | 5.11E-05 | 0.000409 | 1.119474 |
| LINC01194 | lncRNA | DOWN | -1.11303 | -4.51699 | -4.215 | 2.95E-05 | 0.000251 | 1.622124 |
| KCNMB2-AS1 | lncRNA | DOWN | -1.10943 | -1.28184 | -3.95916 | 8.58E-05 | 0.000649 | 0.538776 |
| BARX1-AS1 | lncRNA | DOWN | -1.09496 | -3.77335 | -4.23621 | 2.69E-05 | 0.000231 | 1.734785 |
| CTD-2139B15.5 | lncRNA | DOWN | -1.08461 | -4.07064 | -4.25986 | 2.43E-05 | 0.000211 | 1.828047 |
| RP11-98G7.1 | lncRNA | DOWN | -1.06141 | -2.36417 | -6.0148 | 3.42E-09 | 6.00E-08 | 10.30717 |
| TUSC8 | lncRNA | DOWN | -1.05804 | -4.03573 | -4.10848 | 4.63E-05 | 0.000375 | 1.222109 |
| HOXC13-AS | lncRNA | DOWN | -1.03371 | -3.63559 | -3.98634 | 7.68E-05 | 0.000588 | 0.746064 |
| ST8SIA6-AS1 | lncRNA | DOWN | -1.02927 | -3.71973 | -3.87731 | 0.000119 | 0.000868 | 0.333511 |
| RP11-663N22.1 | lncRNA | DOWN | -1.02881 | -3.33012 | -5.57918 | 3.91E-08 | 5.80E-07 | 7.982478 |
| LINC00402 | lncRNA | UP | 2.051041 | -2.9239 | 14.79804 | 1.57E-41 | 1.94E-39 | 83.53483 |
| RP11-291B21.2 | lncRNA | UP | 1.957555 | -0.86163 | 11.97923 | 2.47E-29 | 1.76E-27 | 55.81401 |
| LINC01281 | lncRNA | UP | 1.918545 | -2.48415 | 15.83503 | 2.76E-46 | 4.25E-44 | 94.35823 |
| RP5-839B4.8 | lncRNA | UP | 1.901805 | -1.86483 | 9.708081 | 1.43E-20 | 6.98E-19 | 35.93229 |
| RP11-428G5.5 | lncRNA | UP | 1.878596 | -3.13221 | 13.54993 | 5.55E-36 | 5.32E-34 | 70.91982 |
| RP11-10J5.1 | lncRNA | UP | 1.86594 | -3.59899 | 13.35033 | 4.09E-35 | 3.75E-33 | 68.94792 |
| RP11-598F7.3 | lncRNA | UP | 1.832269 | -1.8729 | 12.81864 | 7.78E-33 | 6.39E-31 | 63.7804 |
| IL21-AS1 | lncRNA | UP | 1.817194 | -3.55145 | 13.62166 | 2.70E-36 | 2.62E-34 | 71.6283 |
| AC104820.2 | lncRNA | UP | 1.805784 | -2.12403 | 18.99144 | 2.52E-61 | 9.87E-59 | 128.5861 |
| AC079767.4 | lncRNA | UP | 1.797267 | -0.79794 | 16.54386 | 1.34E-49 | 2.54E-47 | 101.9679 |
| LINC00861 | lncRNA | UP | 1.782881 | 0.848173 | 16.72496 | 1.88E-50 | 3.80E-48 | 103.9645 |
| LINC00892 | lncRNA | UP | 1.755709 | -1.36298 | 16.2499 | 3.21E-48 | 5.50E-46 | 98.8023 |
| LINC01215 | lncRNA | UP | 1.748516 | -0.42621 | 14.79603 | 1.60E-41 | 1.98E-39 | 83.56843 |
| AC069363.1 | lncRNA | UP | 1.735418 | -1.62321 | 14.76551 | 2.20E-41 | 2.69E-39 | 83.23086 |
| AC002331.1 | lncRNA | UP | 1.719479 | -2.44635 | 12.08768 | 8.85E-30 | 6.37E-28 | 56.83471 |
| LINC00973 | lncRNA | UP | 1.71088 | -3.16806 | 6.262351 | 8.02E-10 | 1.53E-08 | 11.73804 |
| RP11-693J15.5 | lncRNA | UP | 1.704682 | -2.19843 | 12.49704 | 1.77E-31 | 1.37E-29 | 60.69685 |
| RP5-887A10.1 | lncRNA | UP | 1.691021 | -2.09914 | 11.17055 | 4.36E-26 | 2.76E-24 | 48.44798 |
| SIRPG-AS1 | lncRNA | UP | 1.665848 | -3.83043 | 12.98978 | 1.45E-33 | 1.23E-31 | 65.42508 |
| LINC00158 | lncRNA | UP | 1.664976 | -4.22024 | 14.14414 | 1.35E-38 | 1.46E-36 | 76.86669 |
| TRBV11-2 | lncRNA | UP | 1.660219 | -2.44138 | 15.24358 | 1.47E-43 | 1.99E-41 | 88.15778 |
| AC083949.1 | lncRNA | UP | 1.655608 | -2.01076 | 16.3935 | 6.82E-49 | 1.24E-46 | 100.3056 |
| LINC00944 | lncRNA | UP | 1.654125 | -1.30186 | 11.78881 | 1.47E-28 | 1.01E-26 | 54.05412 |
| RP11-553L6.2 | lncRNA | UP | 1.650416 | -4.21916 | 14.8167 | 1.29E-41 | 1.60E-39 | 83.72556 |
| IFNG-AS1 | lncRNA | UP | 1.635316 | -2.16298 | 10.59346 | 7.49E-24 | 4.24E-22 | 43.37753 |
| RP5-1028K7.2 | lncRNA | UP | 1.628793 | -0.19003 | 15.93721 | 9.24E-47 | 1.48E-44 | 95.5174 |
| CTD-2313F11.1 | lncRNA | UP | 1.622374 | -3.63335 | 13.0697 | 6.61E-34 | 5.75E-32 | 66.20086 |
| CTD-2506J14.1 | lncRNA | UP | 1.614194 | -3.63855 | 14.87344 | 7.14E-42 | 9.05E-40 | 84.29842 |
| AC092580.4 | lncRNA | UP | 1.606621 | -0.78296 | 14.4059 | 9.15E-40 | 1.06E-37 | 79.56111 |
| PIK3CD-AS1 | lncRNA | UP | 1.604893 | -3.80487 | 16.04677 | 2.86E-47 | 4.72E-45 | 96.56559 |
| RP11-148O21.2 | lncRNA | UP | 1.598555 | -2.76837 | 9.898023 | 2.93E-21 | 1.49E-19 | 37.50791 |
| RP11-18H21.1 | lncRNA | UP | 1.596727 | -1.74975 | 15.19379 | 2.48E-43 | 3.33E-41 | 87.66084 |
| RP11-222K16.2 | lncRNA | UP | 1.590737 | -1.68214 | 15.32141 | 6.46E-44 | 8.90E-42 | 88.99325 |
| USP30-AS1 | lncRNA | UP | 1.588328 | -0.21061 | 19.11407 | 6.38E-62 | 2.62E-59 | 130.095 |
| AC006129.2 | lncRNA | UP | 1.579301 | 0.113109 | 17.30914 | 3.19E-53 | 7.53E-51 | 110.2689 |
| LINC00494 | lncRNA | UP | 1.571273 | -2.23155 | 9.895418 | 3.00E-21 | 1.52E-19 | 37.47805 |
| AP000476.1 | lncRNA | UP | 1.569208 | -2.39024 | 17.0133 | 8.12E-52 | 1.74E-49 | 106.9397 |
| TRG-AS1 | lncRNA | UP | 1.538186 | 0.889822 | 21.78413 | 5.11E-75 | 7.28E-72 | 160.0718 |
| RP11-202G18.1 | lncRNA | UP | 1.537899 | -4.51161 | 11.61865 | 7.18E-28 | 4.80E-26 | 52.49877 |
| RP11-476D10.1 | lncRNA | UP | 1.535194 | -1.59281 | 6.708811 | 5.19E-11 | 1.16E-09 | 14.34402 |
| RP11-327F22.2 | lncRNA | UP | 1.534933 | -1.89012 | 18.6671 | 9.44E-60 | 3.22E-57 | 125.0227 |
| RP11-1094M14.5 | lncRNA | UP | 1.527241 | -1.29791 | 16.56932 | 1.02E-49 | 1.95E-47 | 102.2192 |
| LINC00996 | lncRNA | UP | 1.522383 | -0.32066 | 15.79857 | 4.07E-46 | 6.19E-44 | 94.04617 |
| CHRM3-AS2 | lncRNA | UP | 1.513339 | -1.1288 | 12.66358 | 3.52E-32 | 2.80E-30 | 62.28869 |
| RP11-117D22.2 | lncRNA | UP | 1.500391 | -3.37779 | 13.76289 | 6.50E-37 | 6.54E-35 | 73.03229 |
| MIR155HG | lncRNA | UP | 1.499262 | 0.572713 | 19.31635 | 6.60E-63 | 2.97E-60 | 132.3944 |
| GS1-600G8.5 | lncRNA | UP | 1.494772 | -2.24614 | 8.589915 | 1.05E-16 | 4.01E-15 | 27.19991 |
| RP1-167O22.1 | lncRNA | UP | 1.483696 | -4.38147 | 12.61919 | 5.42E-32 | 4.27E-30 | 61.85965 |
| AC013264.2 | lncRNA | UP | 1.483636 | -1.88355 | 8.361501 | 5.85E-16 | 2.12E-14 | 25.50013 |
| RP11-212I21.2 | lncRNA | UP | 1.47309 | -0.89806 | 10.15408 | 3.36E-22 | 1.78E-20 | 39.60287 |
| AC009133.17 | lncRNA | UP | 1.467574 | -2.60721 | 12.26496 | 1.64E-30 | 1.22E-28 | 58.49787 |
| RP11-77A13.1 | lncRNA | UP | 1.467248 | -3.47351 | 6.310393 | 6.01E-10 | 1.17E-08 | 12.02409 |
| RP11-1008C21.1 | lncRNA | UP | 1.458177 | 0.094057 | 10.67052 | 3.80E-24 | 2.19E-22 | 43.98978 |
| XXYLT1-AS2 | lncRNA | UP | 1.45306 | -2.8776 | 10.27844 | 1.16E-22 | 6.23E-21 | 40.69124 |
| RP11-223C24.1 | lncRNA | UP | 1.449391 | -4.17593 | 10.82146 | 1.00E-24 | 5.92E-23 | 45.36895 |
| RP13-452N2.1 | lncRNA | UP | 1.445716 | -3.51634 | 12.4293 | 3.39E-31 | 2.60E-29 | 60.0474 |
| RP5-1091N2.9 | lncRNA | UP | 1.443303 | -1.17409 | 19.62946 | 1.96E-64 | 9.95E-62 | 135.7427 |
| AC006369.2 | lncRNA | UP | 1.442946 | -2.96445 | 13.0182 | 1.10E-33 | 9.38E-32 | 65.70384 |
| RP11-750H9.5 | lncRNA | UP | 1.439832 | 0.375362 | 16.86575 | 4.06E-51 | 8.35E-49 | 105.473 |
| RP11-445F6.2 | lncRNA | UP | 1.438974 | -4.48833 | 11.22901 | 2.57E-26 | 1.64E-24 | 48.97421 |
| KIAA0125 | lncRNA | UP | 1.421064 | 1.583567 | 9.36514 | 2.37E-19 | 1.08E-17 | 33.01437 |
| RP11-81H14.2 | lncRNA | UP | 1.419876 | -0.8157 | 11.89513 | 5.44E-29 | 3.82E-27 | 55.03167 |
| AF127936.5 | lncRNA | UP | 1.418721 | -2.49192 | 12.83367 | 6.72E-33 | 5.54E-31 | 63.92142 |
| PCED1B-AS1 | lncRNA | UP | 1.407779 | 2.939548 | 21.73071 | 9.38E-75 | 1.30E-71 | 159.5987 |
| RP11-327F22.1 | lncRNA | UP | 1.406353 | -3.99195 | 12.81226 | 8.28E-33 | 6.79E-31 | 63.7079 |
| AL928768.3 | lncRNA | UP | 1.405112 | -1.44972 | 9.177246 | 1.07E-18 | 4.65E-17 | 31.67377 |
| RP11-238K6.1 | lncRNA | UP | 1.404042 | -2.40206 | 5.98248 | 4.12E-09 | 7.14E-08 | 10.12805 |
| LINC00426 | lncRNA | UP | 1.400478 | 0.599241 | 17.79497 | 1.52E-55 | 4.11E-53 | 115.5895 |
| RP11-731F5.2 | lncRNA | UP | 1.400153 | -1.92917 | 8.906957 | 9.07E-18 | 3.71E-16 | 29.59131 |
| LINC00943 | lncRNA | UP | 1.399109 | -2.76298 | 9.606643 | 3.31E-20 | 1.58E-18 | 35.1261 |
| LINC01010 | lncRNA | UP | 1.397284 | -1.74227 | 10.59065 | 7.67E-24 | 4.34E-22 | 43.34688 |
| OSTN-AS1 | lncRNA | UP | 1.396313 | -4.00154 | 9.887926 | 3.19E-21 | 1.62E-19 | 37.43129 |
| RP5-1171I10.5 | lncRNA | UP | 1.390255 | 0.305267 | 10.4857 | 1.92E-23 | 1.07E-21 | 42.38206 |
| RP11-148O21.4 | lncRNA | UP | 1.389227 | -4.26809 | 9.123295 | 1.65E-18 | 7.04E-17 | 31.2893 |
| RP11-367G6.3 | lncRNA | UP | 1.384006 | -1.76485 | 14.98563 | 2.21E-42 | 2.85E-40 | 85.49945 |
| RP11-8L8.2 | lncRNA | UP | 1.382055 | -2.228 | 15.86291 | 2.05E-46 | 3.22E-44 | 94.66065 |
| RP11-126O1.6 | lncRNA | UP | 1.374948 | -4.09297 | 11.48384 | 2.49E-27 | 1.65E-25 | 51.27309 |
| RP11-1018N14.5 | lncRNA | UP | 1.371923 | -2.32543 | 11.9645 | 2.83E-29 | 2.01E-27 | 55.6867 |
| IL21R-AS1 | lncRNA | UP | 1.368149 | -3.49128 | 12.28962 | 1.29E-30 | 9.74E-29 | 58.72691 |
| LINC01094 | lncRNA | UP | 1.356679 | 1.678844 | 14.78664 | 1.77E-41 | 2.18E-39 | 83.4666 |
| RP11-145M4.3 | lncRNA | UP | 1.354698 | -4.13831 | 11.9805 | 2.44E-29 | 1.74E-27 | 55.83418 |
| RP11-121A8.1 | lncRNA | UP | 1.354447 | -1.06841 | 16.33003 | 1.35E-48 | 2.37E-46 | 99.66882 |
| LINC01146 | lncRNA | UP | 1.353425 | -2.15286 | 11.87203 | 6.76E-29 | 4.73E-27 | 54.82875 |
| RP3-492J12.2 | lncRNA | UP | 1.351016 | -4.38492 | 12.06881 | 1.06E-29 | 7.61E-28 | 56.65778 |
| LLNLR-470E3.1 | lncRNA | UP | 1.348735 | -1.45649 | 10.41277 | 3.62E-23 | 1.99E-21 | 41.81207 |
| RP11-342D11.3 | lncRNA | UP | 1.347501 | -4.78526 | 11.76478 | 1.84E-28 | 1.26E-26 | 53.8371 |
| HLA-DQB1-AS1 | lncRNA | UP | 1.345437 | 0.553674 | 10.56685 | 9.45E-24 | 5.34E-22 | 43.0715 |
| CH17-360D5.2 | lncRNA | UP | 1.340933 | -0.8104 | 6.521341 | 1.67E-10 | 3.48E-09 | 13.16614 |
| RP11-47L3.1 | lncRNA | UP | 1.339191 | -0.84468 | 15.76867 | 5.60E-46 | 8.45E-44 | 93.71575 |
| RP4-555D20.2 | lncRNA | UP | 1.338372 | -0.74778 | 7.300167 | 1.10E-12 | 3.01E-11 | 18.06547 |
| U62631.5 | lncRNA | UP | 1.337527 | -3.84698 | 9.561972 | 4.78E-20 | 2.25E-18 | 34.7742 |
| RP11-1070N10.3 | lncRNA | UP | 1.337168 | -2.14764 | 10.404 | 3.91E-23 | 2.14E-21 | 41.74995 |
| LINC01150 | lncRNA | UP | 1.328961 | -2.8705 | 12.33159 | 8.66E-31 | 6.56E-29 | 59.12531 |
| ITGB2-AS1 | lncRNA | UP | 1.316291 | 1.741193 | 10.68743 | 3.28E-24 | 1.89E-22 | 44.06002 |
| RP11-701P16.5 | lncRNA | UP | 1.307466 | -1.81904 | 10.95854 | 2.94E-25 | 1.79E-23 | 46.56222 |
| RP11-81H14.1 | lncRNA | UP | 1.305786 | -3.63359 | 9.264855 | 5.33E-19 | 2.35E-17 | 32.40532 |
| C9orf139 | lncRNA | UP | 1.303602 | -0.51026 | 12.92118 | 2.85E-33 | 2.39E-31 | 64.77099 |
| RP11-472N13.3 | lncRNA | UP | 1.30167 | -3.18661 | 11.68588 | 3.85E-28 | 2.60E-26 | 53.11521 |
| LY86-AS1 | lncRNA | UP | 1.301197 | -3.67261 | 10.75815 | 1.75E-24 | 1.03E-22 | 44.8177 |
| RP11-212I21.5 | lncRNA | UP | 1.300976 | -3.78296 | 9.381945 | 2.07E-19 | 9.44E-18 | 33.3329 |
| RP11-16K12.1 | lncRNA | UP | 1.299434 | -1.24371 | 8.756623 | 2.91E-17 | 1.15E-15 | 28.42209 |
| AC079630.2 | lncRNA | UP | 1.294196 | -2.66612 | 5.454581 | 7.64E-08 | 1.09E-06 | 7.316348 |
| CTB-114C7.4 | lncRNA | UP | 1.290645 | -2.99808 | 12.41002 | 4.08E-31 | 3.12E-29 | 59.86669 |
| RP11-325F22.2 | lncRNA | UP | 1.290391 | -1.14832 | 13.20365 | 1.76E-34 | 1.57E-32 | 67.52711 |
| AC021188.4 | lncRNA | UP | 1.284959 | -2.56608 | 14.56659 | 1.74E-40 | 2.05E-38 | 81.16569 |
| AC007278.3 | lncRNA | UP | 1.281411 | -3.47508 | 9.103896 | 1.92E-18 | 8.17E-17 | 31.14341 |
| LINC01358 | lncRNA | UP | 1.277185 | -1.93665 | 13.63948 | 2.26E-36 | 2.20E-34 | 71.8236 |
| RP11-297B17.3 | lncRNA | UP | 1.276143 | -4.84477 | 9.537887 | 5.82E-20 | 2.72E-18 | 34.55037 |
| AC092484.1 | lncRNA | UP | 1.26673 | -4.44878 | 6.524923 | 1.63E-10 | 3.42E-09 | 13.27626 |
| CTC-378H22.2 | lncRNA | UP | 1.265936 | -1.85801 | 12.85977 | 5.20E-33 | 4.31E-31 | 64.17747 |
| AC004988.1 | lncRNA | UP | 1.264154 | -1.77262 | 10.78137 | 1.43E-24 | 8.42E-23 | 45.00515 |
| RP11-404F10.2 | lncRNA | UP | 1.262619 | -2.38843 | 12.211 | 2.74E-30 | 2.02E-28 | 57.99062 |
| RP1-111C20.3 | lncRNA | UP | 1.260148 | -4.02896 | 10.34654 | 6.43E-23 | 3.50E-21 | 41.27368 |
| RP5-899E9.1 | lncRNA | UP | 1.25579 | -1.64742 | 15.12349 | 5.20E-43 | 6.90E-41 | 86.93248 |
| CTC-378H22.1 | lncRNA | UP | 1.254958 | -3.29819 | 11.52011 | 1.79E-27 | 1.18E-25 | 51.6024 |
| CTC-231O11.1 | lncRNA | UP | 1.253672 | 0.081356 | 10.1385 | 3.83E-22 | 2.02E-20 | 39.43625 |
| LINC01229 | lncRNA | UP | 1.25253 | -3.82349 | 10.37183 | 5.16E-23 | 2.82E-21 | 41.48944 |
| RP11-284N8.3 | lncRNA | UP | 1.251529 | 2.725932 | 9.563907 | 4.70E-20 | 2.22E-18 | 34.53732 |
| RP11-542M13.3 | lncRNA | UP | 1.24588 | -4.50942 | 10.91576 | 4.32E-25 | 2.60E-23 | 46.19421 |
| LINC01550 | lncRNA | UP | 1.244688 | 0.081036 | 8.725273 | 3.71E-17 | 1.46E-15 | 28.12443 |
| RP11-340F14.6 | lncRNA | UP | 1.243531 | -3.25242 | 12.22159 | 2.48E-30 | 1.83E-28 | 58.08729 |
| RP11-61F12.1 | lncRNA | UP | 1.24172 | -3.58207 | 9.375068 | 2.19E-19 | 9.97E-18 | 33.278 |
| RP6-159A1.4 | lncRNA | UP | 1.240616 | 0.038065 | 11.0507 | 1.29E-25 | 7.95E-24 | 47.33593 |
| RP3-455J7.4 | lncRNA | UP | 1.239916 | -3.17776 | 10.01064 | 1.14E-21 | 5.87E-20 | 38.44643 |
| LINC00528 | lncRNA | UP | 1.238968 | -1.17923 | 14.28553 | 3.16E-39 | 3.54E-37 | 78.33005 |
| AC022182.3 | lncRNA | UP | 1.237611 | -2.88543 | 11.70649 | 3.17E-28 | 2.15E-26 | 53.30425 |
| CTD-2353F22.1 | lncRNA | UP | 1.233257 | -1.92306 | 10.93742 | 3.56E-25 | 2.15E-23 | 46.37724 |
| LINC00877 | lncRNA | UP | 1.227703 | -1.78073 | 13.23102 | 1.34E-34 | 1.20E-32 | 67.79195 |
| RP11-131H24.4 | lncRNA | UP | 1.225878 | -4.33855 | 9.546942 | 5.40E-20 | 2.54E-18 | 34.6489 |
| RP11-489O18.1 | lncRNA | UP | 1.225326 | -4.19347 | 9.348194 | 2.72E-19 | 1.23E-17 | 33.06286 |
| RP4-647J21.1 | lncRNA | UP | 1.222267 | 1.240285 | 8.071559 | 4.95E-15 | 1.68E-13 | 23.24142 |
| AC131056.3 | lncRNA | UP | 1.220828 | -2.81966 | 8.772069 | 2.59E-17 | 1.03E-15 | 28.58448 |
| AC011899.9 | lncRNA | UP | 1.219608 | 1.005101 | 14.49225 | 3.75E-40 | 4.39E-38 | 80.44597 |
| RP11-290F5.1 | lncRNA | UP | 1.219323 | 0.55303 | 9.204448 | 8.64E-19 | 3.77E-17 | 31.80042 |
| CTD-2341M24.1 | lncRNA | UP | 1.216472 | -1.11695 | 13.0529 | 7.80E-34 | 6.74E-32 | 66.05389 |
| AL122127.25 | lncRNA | UP | 1.210876 | -3.61021 | 8.051045 | 5.74E-15 | 1.94E-13 | 23.30111 |
| SRGAP3-AS2 | lncRNA | UP | 1.210746 | -1.96299 | 4.298379 | 2.06E-05 | 0.000181 | 1.927331 |
| AP003774.1 | lncRNA | UP | 1.209991 | -2.43343 | 12.69189 | 2.68E-32 | 2.14E-30 | 62.55769 |
| MIAT | lncRNA | UP | 1.209121 | 3.26837 | 8.211856 | 1.77E-15 | 6.23E-14 | 24.10145 |
| CTD-2288O8.1 | lncRNA | UP | 1.209109 | -3.24505 | 8.846503 | 1.45E-17 | 5.84E-16 | 29.1576 |
| AC147651.4 | lncRNA | UP | 1.201394 | -0.0957 | 17.1078 | 2.89E-52 | 6.44E-50 | 108.0757 |
| RP11-219E7.1 | lncRNA | UP | 1.199227 | -1.9738 | 12.77906 | 1.14E-32 | 9.35E-31 | 63.39827 |
| RP11-960L18.1 | lncRNA | UP | 1.196237 | -1.55928 | 12.37599 | 5.66E-31 | 4.32E-29 | 59.54759 |
| LINC00968 | lncRNA | UP | 1.19328 | -1.26684 | 9.303271 | 3.91E-19 | 1.75E-17 | 32.66137 |
| RP11-118B22.4 | lncRNA | UP | 1.191212 | -4.40149 | 10.74239 | 2.02E-24 | 1.18E-22 | 44.67795 |
| RP11-365O16.3 | lncRNA | UP | 1.190192 | -2.98502 | 8.478084 | 2.44E-16 | 9.13E-15 | 26.38696 |
| RP5-1073O3.2 | lncRNA | UP | 1.188045 | -4.0682 | 9.676894 | 1.85E-20 | 8.96E-19 | 35.70461 |
| RP13-297E16.4 | lncRNA | UP | 1.187149 | -2.72186 | 10.5426 | 1.17E-23 | 6.56E-22 | 42.94571 |
| LINC01260 | lncRNA | UP | 1.1858 | -2.27488 | 10.88625 | 5.62E-25 | 3.37E-23 | 45.93089 |
| RP3-340N1.2 | lncRNA | UP | 1.181761 | 1.724559 | 5.029233 | 6.82E-07 | 8.23E-06 | 4.919822 |
| RP11-24F11.2 | lncRNA | UP | 1.17751 | -0.57115 | 13.00375 | 1.27E-33 | 1.08E-31 | 65.57373 |
| CADM3-AS1 | lncRNA | UP | 1.175996 | -2.04816 | 7.320408 | 9.59E-13 | 2.64E-11 | 18.25802 |
| RP11-164H13.1 | lncRNA | UP | 1.17477 | -5.04952 | 8.505955 | 1.98E-16 | 7.46E-15 | 26.54139 |
| AC011893.3 | lncRNA | UP | 1.172302 | -4.02051 | 9.583199 | 4.01E-20 | 1.91E-18 | 34.94607 |
| AC096558.1 | lncRNA | UP | 1.170763 | -4.16213 | 9.019876 | 3.74E-18 | 1.57E-16 | 30.49149 |
| RP11-20G13.3 | lncRNA | UP | 1.165047 | -4.26137 | 8.394793 | 4.56E-16 | 1.67E-14 | 25.77897 |
| RP11-473M20.9 | lncRNA | UP | 1.164616 | -0.34666 | 12.63821 | 4.51E-32 | 3.56E-30 | 62.03897 |
| BFSP2-AS1 | lncRNA | UP | 1.164267 | -4.51694 | 9.673908 | 1.90E-20 | 9.18E-19 | 35.6728 |
| AC133644.2 | lncRNA | UP | 1.159844 | -0.36796 | 14.15132 | 1.25E-38 | 1.36E-36 | 76.9768 |
| CH17-360D5.3 | lncRNA | UP | 1.159141 | -2.43404 | 5.444344 | 8.07E-08 | 1.14E-06 | 7.255015 |
| LINC01480 | lncRNA | UP | 1.157165 | -0.26961 | 11.73912 | 2.34E-28 | 1.60E-26 | 53.5787 |
| RP11-756G20.1 | lncRNA | UP | 1.156436 | -3.12912 | 12.20848 | 2.81E-30 | 2.06E-28 | 57.96453 |
| AP002954.4 | lncRNA | UP | 1.155833 | -2.10785 | 9.443895 | 1.25E-19 | 5.77E-18 | 33.80442 |
| ZEB2-AS1 | lncRNA | UP | 1.153949 | -2.32106 | 13.27811 | 8.39E-35 | 7.63E-33 | 68.24842 |
| RP11-79H23.3 | lncRNA | UP | 1.149144 | -1.16132 | 9.829108 | 5.23E-21 | 2.61E-19 | 36.9058 |
| RP11-356N1.2 | lncRNA | UP | 1.14697 | -3.47328 | 9.290251 | 4.34E-19 | 1.93E-17 | 32.60545 |
| AC109826.1 | lncRNA | UP | 1.144889 | -0.80498 | 14.91759 | 4.50E-42 | 5.74E-40 | 84.81828 |
| RP11-44K6.4 | lncRNA | UP | 1.143075 | -3.3664 | 7.003272 | 7.86E-12 | 1.94E-10 | 16.24393 |
| RP11-768B22.2 | lncRNA | UP | 1.141375 | -2.78947 | 7.824178 | 2.93E-14 | 9.30E-13 | 21.69319 |
| RP11-536K7.5 | lncRNA | UP | 1.139349 | -4.16756 | 9.839494 | 4.79E-21 | 2.40E-19 | 37.0333 |
| RP11-67C2.2 | lncRNA | UP | 1.137876 | -1.55434 | 10.44569 | 2.72E-23 | 1.50E-21 | 42.096 |
| RP3-369A17.4 | lncRNA | UP | 1.135752 | -4.19981 | 9.830895 | 5.15E-21 | 2.57E-19 | 36.96241 |
| DBH-AS1 | lncRNA | UP | 1.134148 | 0.300222 | 10.28782 | 1.07E-22 | 5.77E-21 | 40.68966 |
| RP11-452C13.1 | lncRNA | UP | 1.127836 | -2.79784 | 10.72073 | 2.44E-24 | 1.42E-22 | 44.48818 |
| LINC01272 | lncRNA | UP | 1.126803 | 2.964438 | 11.87656 | 6.48E-29 | 4.54E-27 | 54.71739 |
| CTD-2547L24.3 | lncRNA | UP | 1.126718 | -0.22102 | 12.22273 | 2.45E-30 | 1.82E-28 | 58.08546 |
| RP11-109E24.1 | lncRNA | UP | 1.125107 | -4.6797 | 8.543715 | 1.48E-16 | 5.65E-15 | 26.86135 |
| RP11-532F6.3 | lncRNA | UP | 1.12456 | -0.30029 | 11.52946 | 1.64E-27 | 1.09E-25 | 51.65764 |
| RP11-162J8.3 | lncRNA | UP | 1.121958 | -4.2856 | 9.518582 | 6.81E-20 | 3.18E-18 | 34.42308 |
| RP11-138I18.2 | lncRNA | UP | 1.119549 | -4.29772 | 7.797984 | 3.53E-14 | 1.11E-12 | 21.52136 |
| CTD-2616J11.3 | lncRNA | UP | 1.119231 | -3.43484 | 10.07913 | 6.36E-22 | 3.31E-20 | 39.01963 |
| RP11-44K6.2 | lncRNA | UP | 1.118937 | -3.71804 | 6.775131 | 3.41E-11 | 7.82E-10 | 14.81885 |
| AC106801.1 | lncRNA | UP | 1.117395 | -4.29055 | 9.138148 | 1.47E-18 | 6.27E-17 | 31.40893 |
| CTD-2531D15.4 | lncRNA | UP | 1.116297 | -4.15438 | 7.224064 | 1.83E-12 | 4.89E-11 | 17.66672 |
| CTD-2003C8.2 | lncRNA | UP | 1.115315 | -3.29826 | 9.34392 | 2.82E-19 | 1.27E-17 | 33.02873 |
| RP11-624C23.1 | lncRNA | UP | 1.114577 | -4.16322 | 9.339118 | 2.93E-19 | 1.32E-17 | 32.99239 |
| ZBTB20-AS1 | lncRNA | UP | 1.110131 | -3.71969 | 9.751763 | 9.96E-21 | 4.90E-19 | 36.31569 |
| AC104654.2 | lncRNA | UP | 1.102746 | -2.27338 | 9.437588 | 1.32E-19 | 6.07E-18 | 33.75774 |
| AC104699.1 | lncRNA | UP | 1.102013 | -1.81714 | 7.42858 | 4.60E-13 | 1.30E-11 | 18.96667 |
| CTD-2054N24.2 | lncRNA | UP | 1.099793 | -1.92453 | 11.52341 | 1.73E-27 | 1.15E-25 | 51.62804 |
| AC064834.3 | lncRNA | UP | 1.099126 | -4.25641 | 7.85689 | 2.32E-14 | 7.44E-13 | 21.93254 |
| MEF2C-AS1 | lncRNA | UP | 1.095281 | -1.48346 | 9.457281 | 1.12E-19 | 5.18E-18 | 33.89523 |
| LINC00299 | lncRNA | UP | 1.091768 | -2.15974 | 9.287424 | 4.44E-19 | 1.97E-17 | 32.56135 |
| RP11-511B23.1 | lncRNA | UP | 1.091453 | -4.49595 | 8.503246 | 2.02E-16 | 7.60E-15 | 26.57281 |
| AFAP1-AS1 | lncRNA | UP | 1.090978 | 4.588312 | 4.129579 | 4.24E-05 | 0.000346 | 0.703827 |
| LA16c-390H2.4 | lncRNA | UP | 1.089798 | -1.65316 | 9.697819 | 1.56E-20 | 7.58E-19 | 35.84407 |
| CTA-126B4.7 | lncRNA | UP | 1.089161 | -2.44833 | 8.40386 | 4.26E-16 | 1.57E-14 | 25.82798 |
| LINC00840 | lncRNA | UP | 1.088646 | -4.03382 | 6.698488 | 5.54E-11 | 1.24E-09 | 14.34728 |
| XXbac-BPG13B8.10 | lncRNA | UP | 1.08158 | -4.00811 | 7.16507 | 2.71E-12 | 7.09E-11 | 17.28588 |
| HCP5 | lncRNA | UP | 1.07976 | 5.474662 | 12.93566 | 2.47E-33 | 2.07E-31 | 64.6482 |
| CTB-138E5.1 | lncRNA | UP | 1.079739 | -4.85817 | 8.737228 | 3.38E-17 | 1.33E-15 | 28.30251 |
| BZRAP1-AS1 | lncRNA | UP | 1.079271 | -0.20065 | 16.00931 | 4.27E-47 | 6.98E-45 | 96.28249 |
| RP11-169D4.2 | lncRNA | UP | 1.078936 | -2.156 | 12.76969 | 1.25E-32 | 1.02E-30 | 63.307 |
| RP11-23P13.6 | lncRNA | UP | 1.072762 | -1.1321 | 6.774626 | 3.42E-11 | 7.84E-10 | 14.72755 |
| RP11-95H3.1 | lncRNA | UP | 1.072618 | -3.0886 | 8.43959 | 3.26E-16 | 1.21E-14 | 26.10523 |
| RP11-664D1.1 | lncRNA | UP | 1.070998 | -4.15423 | 9.665682 | 2.03E-20 | 9.81E-19 | 35.61389 |
| RP11-33A14.1 | lncRNA | UP | 1.067679 | -4.31759 | 8.082265 | 4.58E-15 | 1.56E-13 | 23.52019 |
| RP1-207H1.3 | lncRNA | UP | 1.066397 | -4.94455 | 8.964496 | 5.78E-18 | 2.40E-16 | 30.02867 |
| FLJ38122 | lncRNA | UP | 1.065447 | -3.91656 | 8.955732 | 6.19E-18 | 2.56E-16 | 29.99945 |
| AC007386.4 | lncRNA | UP | 1.06371 | -1.61614 | 8.062489 | 5.29E-15 | 1.79E-13 | 23.332 |
| RP5-906C1.1 | lncRNA | UP | 1.063622 | -3.31542 | 10.59963 | 7.09E-24 | 4.02E-22 | 43.44175 |
| RP11-556E13.1 | lncRNA | UP | 1.063571 | -2.76052 | 8.436266 | 3.34E-16 | 1.24E-14 | 26.07404 |
| RP11-455F5.5 | lncRNA | UP | 1.062472 | -1.51321 | 9.736931 | 1.13E-20 | 5.52E-19 | 36.15969 |
| LINC00582 | lncRNA | UP | 1.059292 | -2.39717 | 7.202652 | 2.11E-12 | 5.59E-11 | 17.49949 |
| RP5-943J3.1 | lncRNA | UP | 1.058817 | -4.45641 | 8.379618 | 5.11E-16 | 1.86E-14 | 25.66365 |
| LINC01506 | lncRNA | UP | 1.058707 | -3.75771 | 9.067579 | 2.57E-18 | 1.08E-16 | 30.86364 |
| RP11-733O18.1 | lncRNA | UP | 1.058495 | -2.49372 | 8.871046 | 1.20E-17 | 4.87E-16 | 29.33126 |
| RP11-655C2.3 | lncRNA | UP | 1.058075 | -4.53534 | 9.932969 | 2.19E-21 | 1.12E-19 | 37.79866 |
| RP11-49G2.3 | lncRNA | UP | 1.052334 | -3.88027 | 7.995302 | 8.60E-15 | 2.86E-13 | 22.90853 |
| C5orf64 | lncRNA | UP | 1.050406 | -4.60338 | 9.531226 | 6.14E-20 | 2.87E-18 | 34.51743 |
| RP11-1399P15.1 | lncRNA | UP | 1.050246 | -2.37482 | 8.319891 | 7.97E-16 | 2.87E-14 | 25.21164 |
| LINC00544 | lncRNA | UP | 1.047622 | -4.88663 | 9.347282 | 2.74E-19 | 1.24E-17 | 33.02907 |
| RP11-713N11.6 | lncRNA | UP | 1.046172 | -2.63246 | 8.259264 | 1.25E-15 | 4.46E-14 | 24.7779 |
| AC096579.13 | lncRNA | UP | 1.045507 | -2.41302 | 6.375394 | 4.07E-10 | 8.09E-09 | 12.37646 |
| RP4-547N15.3 | lncRNA | UP | 1.043809 | -4.08902 | 4.819799 | 1.89E-06 | 2.10E-05 | 4.26183 |
| RP11-429E11.3 | lncRNA | UP | 1.042292 | -4.81309 | 8.67574 | 5.43E-17 | 2.11E-15 | 27.84185 |
| RP11-524N5.1 | lncRNA | UP | 1.041878 | -4.5673 | 8.785891 | 2.32E-17 | 9.24E-16 | 28.68991 |
| AC019117.2 | lncRNA | UP | 1.039901 | 1.562596 | 5.390596 | 1.07E-07 | 1.49E-06 | 6.717555 |
| RP11-493L12.5 | lncRNA | UP | 1.037119 | -1.95864 | 7.135836 | 3.29E-12 | 8.54E-11 | 17.05022 |
| AC021218.2 | lncRNA | UP | 1.037053 | -2.4863 | 4.874275 | 1.46E-06 | 1.65E-05 | 4.476725 |
| AC079630.4 | lncRNA | UP | 1.032543 | 1.193841 | 5.156863 | 3.59E-07 | 4.56E-06 | 5.581218 |
| LINC01127 | lncRNA | UP | 1.026779 | -2.52657 | 8.368385 | 5.55E-16 | 2.02E-14 | 25.57 |
| AC017060.1 | lncRNA | UP | 1.025159 | 0.007831 | 5.285471 | 1.86E-07 | 2.47E-06 | 6.309961 |
| RP5-998N21.4 | lncRNA | UP | 1.024825 | -3.75211 | 9.12887 | 1.58E-18 | 6.74E-17 | 31.34078 |
| AC009784.3 | lncRNA | UP | 1.02374 | -4.47 | 7.169984 | 2.63E-12 | 6.88E-11 | 17.30714 |
| RP11-275I4.2 | lncRNA | UP | 1.023156 | -4.40713 | 7.588172 | 1.54E-13 | 4.57E-12 | 20.08084 |
| RP11-276H19.1 | lncRNA | UP | 1.021949 | -2.37816 | 8.828692 | 1.67E-17 | 6.68E-16 | 29.00525 |
| AC007386.2 | lncRNA | UP | 1.020177 | -4.34118 | 7.70034 | 7.02E-14 | 2.15E-12 | 20.84848 |
| RP3-477O4.14 | lncRNA | UP | 1.017796 | -1.91431 | 15.1623 | 3.46E-43 | 4.63E-41 | 87.32773 |
| AC097713.4 | lncRNA | UP | 1.014345 | -5.15241 | 9.176007 | 1.08E-18 | 4.70E-17 | 31.65171 |
| RP11-214O1.2 | lncRNA | UP | 1.011395 | -1.24505 | 10.10999 | 4.89E-22 | 2.57E-20 | 39.24246 |
| RP11-203B7.2 | lncRNA | UP | 1.010546 | -4.92992 | 8.05837 | 5.45E-15 | 1.84E-13 | 23.31013 |
| RP11-356I2.4 | lncRNA | UP | 1.010202 | 0.68693 | 14.30717 | 2.53E-39 | 2.86E-37 | 78.55793 |
| CACNA1C-AS2 | lncRNA | UP | 1.009455 | -3.23305 | 9.17557 | 1.09E-18 | 4.71E-17 | 31.70135 |
| RP1-153P14.8 | lncRNA | UP | 1.009253 | -4.86353 | 8.397535 | 4.47E-16 | 1.64E-14 | 25.77096 |
| CTD-2020K17.1 | lncRNA | UP | 1.008753 | 1.761933 | 8.73543 | 3.43E-17 | 1.35E-15 | 28.09786 |
| RP4-728D4.2 | lncRNA | UP | 1.008543 | -1.09708 | 13.69514 | 1.29E-36 | 1.27E-34 | 72.38696 |
| AF127936.3 | lncRNA | UP | 1.008405 | -2.83151 | 6.874764 | 1.81E-11 | 4.27E-10 | 15.42097 |
| AP001055.6 | lncRNA | UP | 1.006088 | -3.39153 | 8.964475 | 5.78E-18 | 2.40E-16 | 30.06406 |
| RP11-354E11.2 | lncRNA | UP | 1.004189 | -1.39546 | 8.560559 | 1.31E-16 | 4.99E-15 | 26.95387 |
| ETNPPL | mRNA | DOWN | -1.9328 | -3.70672 | -6.69637 | 5.61E-11 | 1.25E-09 | 14.31849 |
| FGB | mRNA | DOWN | -1.67031 | 0.960311 | -3.64289 | 0.000297 | 0.00195 | -0.79374 |
| INHA | mRNA | DOWN | -1.58912 | -0.23072 | -5.93905 | 5.28E-09 | 9.02E-08 | 9.771001 |
| FGL1 | mRNA | DOWN | -1.57357 | 1.658485 | -4.69976 | 3.35E-06 | 3.52E-05 | 3.399078 |
| PAH | mRNA | DOWN | -1.51855 | -1.60518 | -5.24112 | 2.33E-07 | 3.06E-06 | 6.191323 |
| HOXC13 | mRNA | DOWN | -1.50675 | -2.06095 | -4.54648 | 6.81E-06 | 6.69E-05 | 2.98312 |
| BARX1 | mRNA | DOWN | -1.47484 | -0.0234 | -4.21122 | 3.00E-05 | 0.000254 | 1.444912 |
| AKR1C2 | mRNA | DOWN | -1.45587 | 4.153177 | -4.2152 | 2.95E-05 | 0.000251 | 1.094457 |
| GPX2 | mRNA | DOWN | -1.44631 | 3.235257 | -4.32425 | 1.84E-05 | 0.000163 | 1.630882 |
| MAGEA3 | mRNA | DOWN | -1.42222 | -2.83988 | -3.47522 | 0.000554 | 0.003364 | -1.12437 |
| TMEM59L | mRNA | DOWN | -1.42115 | 2.073298 | -5.77802 | 1.31E-08 | 2.10E-07 | 8.713124 |
| MSMB | mRNA | DOWN | -1.41812 | 1.135764 | -4.12511 | 4.32E-05 | 0.000352 | 1.004995 |
| TFF1 | mRNA | DOWN | -1.39973 | -0.41344 | -3.68957 | 0.000249 | 0.001666 | -0.51911 |
| NR0B1 | mRNA | DOWN | -1.39671 | -3.50756 | -3.95483 | 8.73E-05 | 0.000659 | 0.618883 |
| CRLF1 | mRNA | DOWN | -1.38571 | 4.350519 | -5.40923 | 9.72E-08 | 1.36E-06 | 6.567501 |
| MAGEA1 | mRNA | DOWN | -1.3814 | -4.21511 | -4.52523 | 7.50E-06 | 7.31E-05 | 2.928079 |
| S100P | mRNA | DOWN | -1.3516 | 5.409965 | -4.69883 | 3.36E-06 | 3.53E-05 | 3.042783 |
| FXYD4 | mRNA | DOWN | -1.35102 | -1.59629 | -5.21905 | 2.61E-07 | 3.40E-06 | 6.082083 |
| FGA | mRNA | DOWN | -1.34879 | 3.480836 | -3.67124 | 0.000267 | 0.001774 | -0.92187 |
| CALCA | mRNA | DOWN | -1.34304 | -1.02654 | -3.25029 | 0.001228 | 0.006727 | -1.95941 |
| COL25A1 | mRNA | DOWN | -1.33855 | -0.93883 | -4.48667 | 8.93E-06 | 8.59E-05 | 2.660409 |
| MAGEA6 | mRNA | DOWN | -1.32401 | -3.29527 | -3.31268 | 0.000989 | 0.005568 | -1.6458 |
| CASKIN1 | mRNA | DOWN | -1.32396 | -0.78265 | -8.65704 | 6.26E-17 | 2.43E-15 | 27.65134 |
| WDR72 | mRNA | DOWN | -1.30534 | 1.265172 | -4.76868 | 2.42E-06 | 2.62E-05 | 3.742922 |
| PITX2 | mRNA | DOWN | -1.28522 | 0.35361 | -4.48336 | 9.07E-06 | 8.71E-05 | 2.552985 |
| KIF1A | mRNA | DOWN | -1.25156 | 0.691585 | -3.64358 | 0.000296 | 0.001946 | -0.77067 |
| BPIFB4 | mRNA | DOWN | -1.18618 | -3.27798 | -4.81538 | 1.94E-06 | 2.13E-05 | 4.232143 |
| CPLX2 | mRNA | DOWN | -1.18154 | -2.06387 | -3.26251 | 0.001178 | 0.006483 | -1.85707 |
| NPW | mRNA | DOWN | -1.17841 | -0.51441 | -5.39895 | 1.03E-07 | 1.43E-06 | 6.918897 |
| KCNU1 | mRNA | DOWN | -1.17421 | -4.54356 | -4.60468 | 5.21E-06 | 5.26E-05 | 3.255 |
| INSL4 | mRNA | DOWN | -1.16567 | -3.62353 | -3.45903 | 0.000587 | 0.003539 | -1.15619 |
| DSCR8 | mRNA | DOWN | -1.15354 | -4.97171 | -5.2967 | 1.75E-07 | 2.34E-06 | 6.456202 |
| PCK1 | mRNA | DOWN | -1.15105 | -3.48591 | -4.06893 | 5.47E-05 | 0.000435 | 1.063009 |
| CELF3 | mRNA | DOWN | -1.13079 | -2.28588 | -4.43688 | 1.12E-05 | 0.000105 | 2.523719 |
| BPIFA1 | mRNA | DOWN | -1.12461 | 3.446522 | -2.86451 | 0.004347 | 0.019637 | -3.50015 |
| RPS4Y1 | mRNA | DOWN | -1.11162 | 1.098458 | -2.19286 | 0.028765 | 0.09029 | -4.95742 |
| LGSN | mRNA | DOWN | -1.10866 | 2.545465 | -3.95585 | 8.70E-05 | 0.000657 | 0.217816 |
| B4GALNT4 | mRNA | DOWN | -1.09931 | 2.055179 | -5.10544 | 4.66E-07 | 5.79E-06 | 5.258917 |
| CPS1 | mRNA | DOWN | -1.09537 | 2.683377 | -3.28718 | 0.001081 | 0.006023 | -2.15265 |
| DLL3 | mRNA | DOWN | -1.09295 | -1.05856 | -4.11662 | 4.48E-05 | 0.000364 | 1.138231 |
| HOXC12 | mRNA | DOWN | -1.08944 | -4.22928 | -3.659 | 0.000279 | 0.001851 | -0.47075 |
| CABYR | mRNA | DOWN | -1.08214 | 2.218812 | -5.81718 | 1.05E-08 | 1.71E-07 | 8.912273 |
| TRPM5 | mRNA | DOWN | -1.08044 | -2.49321 | -5.4613 | 7.37E-08 | 1.05E-06 | 7.34428 |
| FGG | mRNA | DOWN | -1.04694 | 5.000051 | -2.92087 | 0.003644 | 0.016941 | -3.49486 |
| MAGEA12 | mRNA | DOWN | -1.03633 | -3.53608 | -2.88276 | 0.004107 | 0.018745 | -2.93712 |
| LRRC26 | mRNA | DOWN | -1.03316 | -2.51007 | -5.0289 | 6.83E-07 | 8.25E-06 | 5.204253 |
| SLC16A14 | mRNA | DOWN | -1.03289 | 3.762576 | -5.93354 | 5.45E-09 | 9.28E-08 | 9.417507 |
| C19orf45 | mRNA | DOWN | -1.03179 | -1.26874 | -6.88554 | 1.69E-11 | 4.00E-10 | 15.42573 |
| PODXL2 | mRNA | DOWN | -1.02984 | 4.916925 | -6.3094 | 6.05E-10 | 1.18E-08 | 11.45253 |
| AKR7A3 | mRNA | DOWN | -1.02164 | 1.316089 | -4.78432 | 2.25E-06 | 2.44E-05 | 3.808681 |
| FOXI3 | mRNA | DOWN | -1.0112 | -1.74663 | -3.44264 | 0.000623 | 0.003723 | -1.28641 |
| PRAME | mRNA | DOWN | -1.00777 | 1.058783 | -2.85167 | 0.004524 | 0.020313 | -3.31636 |
| POU6F2 | mRNA | DOWN | -1.00524 | -0.84178 | -3.43229 | 0.000647 | 0.003849 | -1.3791 |
| WNK2 | mRNA | DOWN | -1.00069 | 2.981062 | -6.47197 | 2.26E-10 | 4.63E-09 | 12.58997 |
| PLA2G2D | mRNA | UP | 2.463732 | 2.105743 | 15.39285 | 3.04E-44 | 4.27E-42 | 89.77898 |
| MS4A1 | mRNA | UP | 2.346965 | 2.322726 | 13.19407 | 1.93E-34 | 1.72E-32 | 67.36932 |
| CCL19 | mRNA | UP | 2.301106 | 3.528467 | 15.97918 | 5.89E-47 | 9.51E-45 | 95.94668 |
| TCL1A | mRNA | UP | 2.292277 | -0.83999 | 13.80074 | 4.43E-37 | 4.49E-35 | 73.44394 |
| SFTPC | mRNA | UP | 2.269723 | 6.128711 | 5.836653 | 9.44E-09 | 1.55E-07 | 8.658984 |
| CXCL9 | mRNA | UP | 2.262405 | 5.471115 | 14.72309 | 3.42E-41 | 4.15E-39 | 82.6486 |
| FDCSP | mRNA | UP | 2.217248 | 0.239663 | 9.896611 | 2.97E-21 | 1.51E-19 | 37.41138 |
| IFNG | mRNA | UP | 2.205863 | -1.36598 | 14.30027 | 2.71E-39 | 3.06E-37 | 78.4772 |
| CXCL13 | mRNA | UP | 2.162986 | 3.889416 | 13.05637 | 7.54E-34 | 6.52E-32 | 65.93999 |
| TIFAB | mRNA | UP | 2.11191 | -0.48236 | 19.00733 | 2.11E-61 | 8.31E-59 | 128.8916 |
| CLEC6A | mRNA | UP | 2.104617 | -2.43066 | 15.33171 | 5.79E-44 | 8.00E-42 | 89.07824 |
| GZMK | mRNA | UP | 2.07882 | 1.770558 | 20.04097 | 1.90E-66 | 1.16E-63 | 140.5441 |
| CXCL11 | mRNA | UP | 2.036032 | 1.901769 | 13.82841 | 3.35E-37 | 3.42E-35 | 73.6899 |
| FCRL1 | mRNA | UP | 2.024062 | -0.39679 | 13.26416 | 9.64E-35 | 8.73E-33 | 68.12012 |
| AIM2 | mRNA | UP | 2.022562 | 2.160245 | 13.41928 | 2.06E-35 | 1.92E-33 | 69.59772 |
| P2RY12 | mRNA | UP | 2.008216 | -0.21705 | 15.50671 | 9.09E-45 | 1.30E-42 | 90.97235 |
| CHIT1 | mRNA | UP | 2.006695 | 4.636324 | 10.33504 | 7.10E-23 | 3.86E-21 | 40.82364 |
| CLEC10A | mRNA | UP | 1.977422 | 2.527272 | 20.99348 | 4.01E-71 | 3.49E-68 | 151.272 |
| SH2D1A | mRNA | UP | 1.962655 | 1.557154 | 22.17506 | 6.04E-77 | 1.18E-73 | 164.5298 |
| GPR174 | mRNA | UP | 1.958672 | -0.51968 | 18.48554 | 7.14E-59 | 2.37E-56 | 123.1211 |
| CXCL10 | mRNA | UP | 1.952765 | 4.265596 | 14.33186 | 1.96E-39 | 2.24E-37 | 78.69448 |
| TRAT1 | mRNA | UP | 1.952098 | 0.463114 | 19.30574 | 7.44E-63 | 3.31E-60 | 132.2684 |
| CNR2 | mRNA | UP | 1.909395 | -0.87773 | 14.18643 | 8.73E-39 | 9.60E-37 | 77.32858 |
| TLR8 | mRNA | UP | 1.895713 | 2.275718 | 19.15294 | 4.13E-62 | 1.76E-59 | 130.6421 |
| SPIB | mRNA | UP | 1.893374 | 1.343298 | 13.79213 | 4.84E-37 | 4.89E-35 | 73.34012 |
| FCRL3 | mRNA | UP | 1.888619 | 1.104777 | 15.361 | 4.25E-44 | 5.94E-42 | 89.45429 |
| FAM26F | mRNA | UP | 1.886257 | 2.484675 | 18.04008 | 1.01E-56 | 2.87E-54 | 118.3183 |
| HLA-DQA1 | mRNA | UP | 1.873316 | 7.560981 | 19.98907 | 3.41E-66 | 2.01E-63 | 139.8682 |
| THEMIS | mRNA | UP | 1.870678 | 1.097931 | 19.612 | 2.38E-64 | 1.20E-61 | 135.717 |
| UBD | mRNA | UP | 1.870249 | 2.66059 | 12.41216 | 4.00E-31 | 3.06E-29 | 59.78039 |
| FCER2 | mRNA | UP | 1.869034 | -1.05415 | 12.17915 | 3.71E-30 | 2.71E-28 | 57.68687 |
| CRTAM | mRNA | UP | 1.851007 | 0.636702 | 21.7065 | 1.23E-74 | 1.68E-71 | 159.1728 |
| CD1B | mRNA | UP | 1.840805 | -0.94864 | 11.21507 | 2.91E-26 | 1.86E-24 | 48.82967 |
| ICOS | mRNA | UP | 1.839674 | 1.021886 | 21.56157 | 6.39E-74 | 8.29E-71 | 157.5769 |
| ZNF683 | mRNA | UP | 1.839335 | 1.039888 | 13.74118 | 8.09E-37 | 8.09E-35 | 72.83588 |
| TTC24 | mRNA | UP | 1.827734 | -1.85721 | 14.24992 | 4.55E-39 | 5.07E-37 | 77.9569 |
| GBP5 | mRNA | UP | 1.820794 | 4.448181 | 16.66519 | 3.60E-50 | 7.12E-48 | 103.2732 |
| IL22RA2 | mRNA | UP | 1.810084 | -0.64435 | 10.66839 | 3.88E-24 | 2.22E-22 | 43.99552 |
| LYZ | mRNA | UP | 1.801559 | 8.107004 | 14.69615 | 4.53E-41 | 5.46E-39 | 82.14888 |
| SIRPG | mRNA | UP | 1.798261 | 1.445676 | 20.30899 | 9.23E-68 | 6.06E-65 | 143.5325 |
| ZNF831 | mRNA | UP | 1.797411 | 0.867545 | 18.41865 | 1.50E-58 | 4.82E-56 | 122.4575 |
| RTP5 | mRNA | UP | 1.796179 | -3.56682 | 13.93767 | 1.11E-37 | 1.15E-35 | 74.78156 |
| CYBB | mRNA | UP | 1.792893 | 6.287012 | 20.81817 | 2.92E-70 | 2.30E-67 | 149.2803 |
| P2RY13 | mRNA | UP | 1.791501 | 2.293813 | 21.15577 | 6.37E-72 | 6.30E-69 | 153.0904 |
| SCGB1A1 | mRNA | UP | 1.789237 | 3.155609 | 4.861658 | 1.55E-06 | 1.74E-05 | 4.007831 |
| FCRLA | mRNA | UP | 1.786792 | 1.118011 | 11.954 | 3.13E-29 | 2.22E-27 | 55.5288 |
| CXCR5 | mRNA | UP | 1.783795 | -2.43354 | 12.61234 | 5.79E-32 | 4.55E-30 | 61.796 |
| CLEC4C | mRNA | UP | 1.782538 | -3.36498 | 12.24159 | 2.05E-30 | 1.53E-28 | 58.27574 |
| CD48 | mRNA | UP | 1.77826 | 4.010516 | 21.94525 | 8.21E-76 | 1.37E-72 | 162.0468 |
| EREG | mRNA | UP | 1.77699 | 1.562427 | 6.070395 | 2.48E-09 | 4.45E-08 | 10.37451 |
| CCL5 | mRNA | UP | 1.77661 | 5.144697 | 18.72842 | 4.76E-60 | 1.65E-57 | 125.8907 |
| CCL17 | mRNA | UP | 1.77479 | 0.440489 | 10.90083 | 4.93E-25 | 2.96E-23 | 45.99461 |
| ADAMDEC1 | mRNA | UP | 1.773728 | 2.480006 | 11.13206 | 6.18E-26 | 3.87E-24 | 47.94994 |
| FCRL4 | mRNA | UP | 1.773128 | -2.03518 | 11.37408 | 6.83E-27 | 4.44E-25 | 50.27509 |
| GZMA | mRNA | UP | 1.771767 | 2.861677 | 17.50112 | 3.87E-54 | 9.69E-52 | 112.4027 |
| TLR10 | mRNA | UP | 1.770971 | 1.250911 | 15.07531 | 8.62E-43 | 1.14E-40 | 86.4681 |
| BLK | mRNA | UP | 1.769468 | 0.731882 | 11.98657 | 2.30E-29 | 1.64E-27 | 55.8461 |
| PYHIN1 | mRNA | UP | 1.769286 | 1.005574 | 21.33728 | 8.14E-73 | 8.96E-70 | 155.0525 |
| ITK | mRNA | UP | 1.765278 | 2.739451 | 20.83141 | 2.51E-70 | 2.01E-67 | 149.4554 |
| PTPRC | mRNA | UP | 1.763092 | 5.924206 | 24.46735 | 3.05E-88 | 2.97E-84 | 190.5648 |
| FASLG | mRNA | UP | 1.754744 | 0.080897 | 16.11259 | 1.41E-47 | 2.34E-45 | 97.38731 |
| CD2 | mRNA | UP | 1.754239 | 3.955194 | 22.98935 | 5.82E-81 | 1.70E-77 | 173.8471 |
| CD8A | mRNA | UP | 1.749251 | 3.605892 | 17.43757 | 7.78E-54 | 1.90E-51 | 111.6961 |
| SCML4 | mRNA | UP | 1.749065 | 0.477931 | 18.21045 | 1.52E-57 | 4.63E-55 | 120.146 |
| GZMH | mRNA | UP | 1.748346 | 1.57006 | 15.90665 | 1.28E-46 | 2.04E-44 | 95.21132 |
| MARCO | mRNA | UP | 1.746436 | 4.733901 | 10.71373 | 2.60E-24 | 1.51E-22 | 44.09123 |
| NKG7 | mRNA | UP | 1.746115 | 2.896098 | 17.07396 | 4.19E-52 | 9.19E-50 | 107.7456 |
| KLRC4-KLRK1 | mRNA | UP | 1.741039 | -2.90123 | 13.67716 | 1.54E-36 | 1.52E-34 | 72.18485 |
| BTLA | mRNA | UP | 1.738529 | 0.306135 | 19.3944 | 2.75E-63 | 1.30E-60 | 133.2454 |
| TIGIT | mRNA | UP | 1.738438 | 2.723938 | 20.44194 | 2.06E-68 | 1.43E-65 | 145.0755 |
| NCR3 | mRNA | UP | 1.735515 | -0.47993 | 18.00301 | 1.52E-56 | 4.30E-54 | 117.8175 |
| DTHD1 | mRNA | UP | 1.732502 | -0.04961 | 10.56058 | 9.99E-24 | 5.63E-22 | 43.042 |
| CCR6 | mRNA | UP | 1.730858 | -2.13517 | 14.06121 | 3.14E-38 | 3.35E-36 | 76.04362 |
| SIT1 | mRNA | UP | 1.729581 | 1.580437 | 19.84394 | 1.75E-65 | 9.65E-63 | 138.332 |
| PLEK | mRNA | UP | 1.726292 | 4.927038 | 24.10661 | 1.82E-86 | 1.52E-82 | 186.4889 |
| LILRA4 | mRNA | UP | 1.7229 | -0.601 | 14.46422 | 5.01E-40 | 5.84E-38 | 80.15872 |
| HLA-DQA2 | mRNA | UP | 1.720192 | 4.777955 | 9.747681 | 1.03E-20 | 5.06E-19 | 35.88278 |
| HLA-DOA | mRNA | UP | 1.717567 | 5.566762 | 18.13584 | 3.48E-57 | 1.02E-54 | 119.3038 |
| CCR2 | mRNA | UP | 1.716527 | 2.4672 | 19.10006 | 7.46E-62 | 3.05E-59 | 130.057 |
| CD69 | mRNA | UP | 1.715505 | 2.925357 | 18.82014 | 1.71E-60 | 6.12E-58 | 126.9483 |
| SCIMP | mRNA | UP | 1.714138 | 2.597194 | 22.82456 | 3.78E-80 | 1.00E-76 | 171.929 |
| CCR4 | mRNA | UP | 1.710929 | 1.676388 | 15.59744 | 3.47E-45 | 5.10E-43 | 91.93744 |
| EOMES | mRNA | UP | 1.704285 | 0.786432 | 17.16897 | 1.48E-52 | 3.39E-50 | 108.7672 |
| CD3E | mRNA | UP | 1.702936 | 4.192568 | 22.61507 | 4.08E-79 | 9.54E-76 | 169.6212 |
| CLEC12A | mRNA | UP | 1.699502 | 1.478186 | 15.21626 | 1.96E-43 | 2.64E-41 | 87.9364 |
| IRG1 | mRNA | UP | 1.698815 | -3.90302 | 11.02907 | 1.56E-25 | 9.61E-24 | 47.19665 |
| P2RY10 | mRNA | UP | 1.69802 | 1.178562 | 18.48111 | 7.50E-59 | 2.48E-56 | 123.1592 |
| LTA | mRNA | UP | 1.694568 | -0.18691 | 20.38213 | 4.04E-68 | 2.78E-65 | 144.2317 |
| CLECL1 | mRNA | UP | 1.691545 | -0.40737 | 19.69739 | 9.12E-65 | 4.84E-62 | 136.5661 |
| TFEC | mRNA | UP | 1.687079 | 2.947564 | 21.3248 | 9.37E-73 | 1.01E-69 | 155.0198 |
| CD40LG | mRNA | UP | 1.686386 | 0.312214 | 16.01488 | 4.02E-47 | 6.59E-45 | 96.35262 |
| CCR5 | mRNA | UP | 1.685977 | 3.368036 | 23.58369 | 6.84E-84 | 3.63E-80 | 180.5385 |
| PDCD1 | mRNA | UP | 1.683721 | 1.439055 | 17.05802 | 4.98E-52 | 1.08E-49 | 107.5749 |
| SERPINA9 | mRNA | UP | 1.681982 | -3.77461 | 9.843228 | 4.65E-21 | 2.32E-19 | 37.06207 |
| CCL18 | mRNA | UP | 1.680122 | 5.82432 | 12.04924 | 1.27E-29 | 9.13E-28 | 56.13202 |
| MRC1 | mRNA | UP | 1.67982 | 5.688846 | 13.43356 | 1.78E-35 | 1.67E-33 | 69.53791 |
| ZNF80 | mRNA | UP | 1.679033 | -2.74477 | 12.89281 | 3.76E-33 | 3.12E-31 | 64.49081 |
| AICDA | mRNA | UP | 1.675894 | -3.18629 | 11.40929 | 4.95E-27 | 3.23E-25 | 50.5979 |
| SELL | mRNA | UP | 1.671302 | 3.857362 | 17.88835 | 5.40E-56 | 1.50E-53 | 116.6347 |
| ABCD2 | mRNA | UP | 1.6698 | 0.159227 | 19.14746 | 4.39E-62 | 1.84E-59 | 130.4907 |
| AOAH | mRNA | UP | 1.667075 | 3.178966 | 21.97638 | 5.77E-76 | 1.02E-72 | 162.3795 |
| EMR1 | mRNA | UP | 1.666898 | 0.369877 | 13.89483 | 1.71E-37 | 1.76E-35 | 74.38676 |
| TNFRSF13B | mRNA | UP | 1.666063 | -0.34874 | 11.78088 | 1.59E-28 | 1.09E-26 | 53.96576 |
| IL7R | mRNA | UP | 1.664808 | 5.253268 | 18.56754 | 2.86E-59 | 9.61E-57 | 124.0989 |
| PDCD1LG2 | mRNA | UP | 1.663271 | 2.069928 | 19.14468 | 4.53E-62 | 1.89E-59 | 130.5459 |
| TNFRSF9 | mRNA | UP | 1.657838 | 2.267816 | 15.95592 | 7.56E-47 | 1.21E-44 | 95.7281 |
| C1QB | mRNA | UP | 1.654576 | 7.431861 | 18.31263 | 4.89E-58 | 1.53E-55 | 121.1456 |
| CD3D | mRNA | UP | 1.654006 | 2.873746 | 19.38696 | 2.99E-63 | 1.40E-60 | 133.2605 |
| CCL13 | mRNA | UP | 1.653346 | 2.774279 | 11.00053 | 2.02E-25 | 1.23E-23 | 46.75878 |
| CCR7 | mRNA | UP | 1.650118 | 2.472255 | 17.09372 | 3.37E-52 | 7.46E-50 | 107.9646 |
| IL21R | mRNA | UP | 1.644721 | 2.526557 | 20.33798 | 6.66E-68 | 4.47E-65 | 143.9028 |
| HLA-DPA1 | mRNA | UP | 1.641755 | 8.615367 | 18.4138 | 1.59E-58 | 5.01E-56 | 122.1698 |
| SLAMF6 | mRNA | UP | 1.638938 | 2.702468 | 19.38164 | 3.17E-63 | 1.47E-60 | 133.1991 |
| TBX21 | mRNA | UP | 1.637302 | 0.63551 | 16.20737 | 5.08E-48 | 8.57E-46 | 98.40839 |
| CD247 | mRNA | UP | 1.633879 | 2.701474 | 23.35116 | 9.57E-83 | 3.73E-79 | 177.8782 |
| MNDA | mRNA | UP | 1.633742 | 3.763549 | 19.38026 | 3.22E-63 | 1.48E-60 | 133.1869 |
| CD52 | mRNA | UP | 1.630105 | 4.864918 | 19.92853 | 6.75E-66 | 3.87E-63 | 139.3105 |
| SAMD3 | mRNA | UP | 1.622114 | 0.099549 | 21.49055 | 1.43E-73 | 1.71E-70 | 156.6917 |
| UBASH3A | mRNA | UP | 1.618941 | 1.096788 | 19.97229 | 4.12E-66 | 2.41E-63 | 139.7432 |
| SAA2-SAA4 | mRNA | UP | 1.617864 | -2.26038 | 7.023083 | 6.91E-12 | 1.72E-10 | 16.33746 |
| DCSTAMP | mRNA | UP | 1.616802 | 0.047337 | 12.36168 | 6.49E-31 | 4.94E-29 | 59.39424 |
| HLA-DRA | mRNA | UP | 1.615799 | 10.13486 | 18.88484 | 8.30E-61 | 3.05E-58 | 127.2606 |
| ZAP70 | mRNA | UP | 1.615046 | 2.813434 | 17.41323 | 1.02E-53 | 2.47E-51 | 111.4425 |
| CD96 | mRNA | UP | 1.613073 | 3.18922 | 18.41379 | 1.59E-58 | 5.01E-56 | 122.4425 |
| CD19 | mRNA | UP | 1.612053 | 1.34768 | 11.77384 | 1.69E-28 | 1.16E-26 | 53.84663 |
| SLAMF1 | mRNA | UP | 1.612046 | 1.934935 | 20.93507 | 7.76E-71 | 6.57E-68 | 150.59 |
| CLEC17A | mRNA | UP | 1.610977 | -1.35972 | 11.80533 | 1.26E-28 | 8.71E-27 | 54.20718 |
| SSTR3 | mRNA | UP | 1.610415 | -2.37785 | 14.11912 | 1.74E-38 | 1.87E-36 | 76.62296 |
| CD1E | mRNA | UP | 1.607347 | 0.836368 | 9.577935 | 4.19E-20 | 1.99E-18 | 34.76995 |
| ITGAL | mRNA | UP | 1.606121 | 4.915919 | 22.59415 | 5.18E-79 | 1.16E-75 | 169.3903 |
| FGL2 | mRNA | UP | 1.606085 | 4.968058 | 18.92481 | 5.31E-61 | 1.97E-58 | 128.0819 |
| SPN | mRNA | UP | 1.604746 | 4.156939 | 20.69541 | 1.17E-69 | 8.87E-67 | 147.9433 |
| CD3G | mRNA | UP | 1.603002 | 2.291467 | 19.18513 | 2.88E-62 | 1.25E-59 | 131.0013 |
| VSIG4 | mRNA | UP | 1.601446 | 5.017339 | 14.8269 | 1.16E-41 | 1.45E-39 | 83.75012 |
| C1QA | mRNA | UP | 1.601263 | 7.20234 | 18.84685 | 1.27E-60 | 4.60E-58 | 127.0994 |
| ADH1B | mRNA | UP | 1.600921 | 3.783381 | 6.763957 | 3.66E-11 | 8.37E-10 | 14.30141 |
| FAM129C | mRNA | UP | 1.598763 | -0.20591 | 11.72217 | 2.74E-28 | 1.87E-26 | 53.42117 |
| LILRB4 | mRNA | UP | 1.597653 | 4.522942 | 17.11407 | 2.70E-52 | 6.04E-50 | 108.1383 |
| CD84 | mRNA | UP | 1.596923 | 4.355668 | 20.01412 | 2.57E-66 | 1.53E-63 | 140.2801 |
| PVALB | mRNA | UP | 1.596921 | -2.15466 | 11.17443 | 4.21E-26 | 2.66E-24 | 48.48316 |
| SNX20 | mRNA | UP | 1.595462 | 3.271972 | 24.95033 | 1.29E-90 | 2.52E-86 | 195.9279 |
| AMICA1 | mRNA | UP | 1.594662 | 4.179974 | 20.6865 | 1.29E-69 | 9.69E-67 | 147.8429 |
| IGSF6 | mRNA | UP | 1.593424 | 4.260031 | 21.18002 | 4.84E-72 | 4.96E-69 | 153.4068 |
| SLC12A3 | mRNA | UP | 1.589189 | -1.84714 | 13.67107 | 1.64E-36 | 1.61E-34 | 72.13923 |
| GIMAP5 | mRNA | UP | 1.588477 | 0.791855 | 19.37986 | 3.24E-63 | 1.48E-60 | 133.1137 |
| COL6A5 | mRNA | UP | 1.587102 | 1.289625 | 9.978891 | 1.49E-21 | 7.63E-20 | 38.04072 |
| TESPA1 | mRNA | UP | 1.585942 | 1.66253 | 21.01436 | 3.16E-71 | 2.88E-68 | 151.4671 |
| CTLA4 | mRNA | UP | 1.585905 | 1.586602 | 17.63583 | 8.77E-55 | 2.28E-52 | 113.8728 |
| NCKAP1L | mRNA | UP | 1.584895 | 5.264012 | 23.49247 | 1.92E-83 | 8.03E-80 | 179.5521 |
| FUT7 | mRNA | UP | 1.584417 | -0.40182 | 16.69597 | 2.58E-50 | 5.14E-48 | 103.6171 |
| LCK | mRNA | UP | 1.580624 | 3.562527 | 20.78905 | 4.05E-70 | 3.12E-67 | 148.9954 |
| IL2RG | mRNA | UP | 1.575511 | 5.196534 | 21.23869 | 2.49E-72 | 2.59E-69 | 154.0606 |
| MS4A6A | mRNA | UP | 1.573891 | 5.372063 | 20.36232 | 5.06E-68 | 3.43E-65 | 144.1736 |
| SAA1 | mRNA | UP | 1.57361 | 2.188131 | 7.259996 | 1.44E-12 | 3.88E-11 | 17.60398 |
| SASH3 | mRNA | UP | 1.573473 | 4.478052 | 25.8419 | 5.55E-95 | 3.24E-90 | 206.0011 |
| SLAMF8 | mRNA | UP | 1.573368 | 4.500572 | 20.52679 | 7.88E-69 | 5.54E-66 | 146.0422 |
| CXCR6 | mRNA | UP | 1.57331 | 2.67998 | 19.67277 | 1.20E-64 | 6.22E-62 | 136.4515 |
| HLA-DRB5 | mRNA | UP | 1.573209 | 7.572159 | 12.78577 | 1.07E-32 | 8.77E-31 | 63.00708 |
| GZMB | mRNA | UP | 1.573196 | 2.701243 | 12.55726 | 9.87E-32 | 7.68E-30 | 61.16442 |
| TRAF3IP3 | mRNA | UP | 1.572605 | 2.949594 | 22.24475 | 2.74E-77 | 5.51E-74 | 165.4014 |
| IRF8 | mRNA | UP | 1.571592 | 4.350919 | 21.91645 | 1.14E-75 | 1.85E-72 | 161.7247 |
| S100B | mRNA | UP | 1.570365 | 2.382948 | 10.96445 | 2.79E-25 | 1.70E-23 | 46.46142 |
| HLA-DPB1 | mRNA | UP | 1.570331 | 8.336155 | 18.86342 | 1.05E-60 | 3.85E-58 | 127.1967 |
| PTGDS | mRNA | UP | 1.570235 | 5.358395 | 13.54359 | 5.92E-36 | 5.66E-34 | 70.65773 |
| CCL4 | mRNA | UP | 1.569878 | 3.06082 | 17.67689 | 5.58E-55 | 1.48E-52 | 114.3253 |
| IL2RA | mRNA | UP | 1.568587 | 2.812887 | 15.79575 | 4.19E-46 | 6.36E-44 | 94.01567 |
| GPR18 | mRNA | UP | 1.568136 | -0.34329 | 18.7962 | 2.23E-60 | 7.95E-58 | 126.5639 |
| NCF1 | mRNA | UP | 1.564728 | 1.934061 | 19.73505 | 5.97E-65 | 3.23E-62 | 137.1287 |
| LTB | mRNA | UP | 1.562142 | 3.660355 | 15.80306 | 3.88E-46 | 5.91E-44 | 94.06711 |
| TLR7 | mRNA | UP | 1.560832 | 2.289401 | 17.09075 | 3.48E-52 | 7.68E-50 | 107.9334 |
| SIGLEC10 | mRNA | UP | 1.558776 | 3.177992 | 17.63287 | 9.06E-55 | 2.34E-52 | 113.8413 |
| HK3 | mRNA | UP | 1.558647 | 3.387272 | 15.48824 | 1.11E-44 | 1.58E-42 | 90.74678 |
| SAA2 | mRNA | UP | 1.554443 | 0.829566 | 6.932277 | 1.25E-11 | 3.01E-10 | 15.59218 |
| SLAMF7 | mRNA | UP | 1.553707 | 4.990381 | 14.85706 | 8.47E-42 | 1.07E-39 | 84.06461 |
| RTN1 | mRNA | UP | 1.553334 | 2.267019 | 16.36181 | 9.61E-49 | 1.71E-46 | 100.0639 |
| CARD17 | mRNA | UP | 1.552815 | -3.01845 | 12.60689 | 6.11E-32 | 4.79E-30 | 61.74031 |
| CLEC4D | mRNA | UP | 1.552174 | -1.74009 | 13.64758 | 2.08E-36 | 2.03E-34 | 71.90683 |
| GPR65 | mRNA | UP | 1.551495 | 2.329736 | 23.52867 | 1.28E-83 | 5.73E-80 | 179.8547 |
| C1QC | mRNA | UP | 1.551132 | 7.213885 | 17.5098 | 3.51E-54 | 8.92E-52 | 112.308 |
| XCL2 | mRNA | UP | 1.550477 | -1.08959 | 13.5771 | 4.23E-36 | 4.07E-34 | 71.21228 |
| CD53 | mRNA | UP | 1.549543 | 5.793371 | 24.88968 | 2.57E-90 | 3.75E-86 | 195.3279 |
| IKZF1 | mRNA | UP | 1.5469 | 4.232161 | 22.83406 | 3.39E-80 | 9.44E-77 | 172.098 |
| IL12RB1 | mRNA | UP | 1.546104 | 2.354867 | 23.16946 | 7.53E-82 | 2.75E-78 | 175.8058 |
| BTK | mRNA | UP | 1.544487 | 3.444703 | 22.75237 | 8.59E-80 | 2.09E-76 | 171.154 |
| PTCRA | mRNA | UP | 1.539311 | -1.64571 | 13.86418 | 2.33E-37 | 2.39E-35 | 74.07108 |
| KLRB1 | mRNA | UP | 1.539242 | 1.895462 | 18.44797 | 1.09E-58 | 3.52E-56 | 122.8125 |
| FCN1 | mRNA | UP | 1.537999 | 2.964349 | 13.37858 | 3.09E-35 | 2.85E-33 | 69.15949 |
| HLA-DMB | mRNA | UP | 1.536281 | 6.216289 | 19.63772 | 1.79E-64 | 9.14E-62 | 135.9994 |
| FCGR1A | mRNA | UP | 1.534573 | 2.43329 | 17.94566 | 2.86E-56 | 8.08E-54 | 117.2793 |
| HLA-DRB1 | mRNA | UP | 1.531195 | 9.28988 | 16.33485 | 1.29E-48 | 2.26E-46 | 99.34545 |
| RGS18 | mRNA | UP | 1.530751 | 1.053448 | 18.14905 | 3.01E-57 | 8.91E-55 | 119.4931 |
| GBP6 | mRNA | UP | 1.530697 | -0.36342 | 8.143043 | 2.94E-15 | 1.02E-13 | 23.85151 |
| PTPN7 | mRNA | UP | 1.529062 | 3.762159 | 23.53914 | 1.13E-83 | 5.51E-80 | 180.0526 |
| FYB | mRNA | UP | 1.528459 | 4.690461 | 19.86624 | 1.36E-65 | 7.65E-63 | 138.6151 |
| IL4I1 | mRNA | UP | 1.528198 | 3.610117 | 14.37903 | 1.21E-39 | 1.39E-37 | 79.20895 |
| CD5 | mRNA | UP | 1.528195 | 3.032488 | 18.16589 | 2.49E-57 | 7.47E-55 | 119.7042 |
| GBP4 | mRNA | UP | 1.527686 | 5.437542 | 17.38824 | 1.34E-53 | 3.23E-51 | 111.0889 |
| LST1 | mRNA | UP | 1.527012 | 3.710808 | 18.95409 | 3.82E-61 | 1.47E-58 | 128.4349 |
| CD8B | mRNA | UP | 1.526883 | 1.30664 | 14.14429 | 1.34E-38 | 1.46E-36 | 76.89335 |
| GPR84 | mRNA | UP | 1.526118 | 1.091919 | 14.11919 | 1.74E-38 | 1.87E-36 | 76.64267 |
| CD79B | mRNA | UP | 1.52403 | 2.370248 | 14.23282 | 5.42E-39 | 6.01E-37 | 77.76537 |
| CXCR3 | mRNA | UP | 1.523468 | 2.22236 | 16.55736 | 1.16E-49 | 2.21E-47 | 102.1655 |
| HAVCR2 | mRNA | UP | 1.521143 | 4.316803 | 21.11054 | 1.06E-71 | 1.03E-68 | 152.6227 |
| SIGLEC14 | mRNA | UP | 1.52073 | 1.557858 | 9.175503 | 1.09E-18 | 4.71E-17 | 31.51395 |
| CSF2RB | mRNA | UP | 1.520181 | 4.30811 | 21.49038 | 1.43E-73 | 1.71E-70 | 156.9105 |
| KLRD1 | mRNA | UP | 1.518299 | 1.677442 | 15.28839 | 9.16E-44 | 1.25E-41 | 88.68862 |
| DOK2 | mRNA | UP | 1.518291 | 3.673649 | 20.53128 | 7.49E-69 | 5.33E-66 | 146.0948 |
| FOLR2 | mRNA | UP | 1.516817 | 3.848359 | 14.32888 | 2.02E-39 | 2.30E-37 | 78.68438 |
| TAGAP | mRNA | UP | 1.516541 | 3.442452 | 22.76661 | 7.30E-80 | 1.85E-76 | 171.315 |
| STAP1 | mRNA | UP | 1.514581 | 0.199087 | 15.03056 | 1.38E-42 | 1.79E-40 | 86.00311 |
| F13A1 | mRNA | UP | 1.514382 | 5.014533 | 11.4686 | 2.87E-27 | 1.89E-25 | 50.81892 |
| CR1 | mRNA | UP | 1.513307 | 2.089709 | 14.28064 | 3.32E-39 | 3.72E-37 | 78.26119 |
| CD28 | mRNA | UP | 1.51245 | 2.044185 | 19.05622 | 1.22E-61 | 4.88E-59 | 129.5612 |
| CD79A | mRNA | UP | 1.510848 | 4.087478 | 10.5213 | 1.41E-23 | 7.85E-22 | 42.46579 |
| DNAJC5B | mRNA | UP | 1.510422 | 0.842845 | 15.05157 | 1.11E-42 | 1.45E-40 | 86.22317 |
| IDO1 | mRNA | UP | 1.510082 | 4.423147 | 10.56291 | 9.79E-24 | 5.52E-22 | 42.80036 |
| EVI2B | mRNA | UP | 1.507542 | 4.362101 | 23.82165 | 4.60E-85 | 2.98E-81 | 183.261 |
| CD22 | mRNA | UP | 1.506437 | 2.540133 | 13.01239 | 1.16E-33 | 9.91E-32 | 65.57706 |
| TNFSF13B | mRNA | UP | 1.505814 | 3.907231 | 20.66427 | 1.66E-69 | 1.23E-66 | 147.5926 |
| CCR8 | mRNA | UP | 1.505174 | -0.77493 | 14.67882 | 5.42E-41 | 6.50E-39 | 82.35621 |
| DOCK2 | mRNA | UP | 1.504672 | 4.788888 | 21.69509 | 1.40E-74 | 1.86E-71 | 159.2225 |
| CD300LF | mRNA | UP | 1.504649 | 2.706478 | 17.49887 | 3.96E-54 | 9.89E-52 | 112.3792 |
| NCR1 | mRNA | UP | 1.503624 | -2.22466 | 12.60323 | 6.33E-32 | 4.96E-30 | 61.70977 |
| CD163 | mRNA | UP | 1.50281 | 6.036404 | 14.3183 | 2.25E-39 | 2.56E-37 | 78.43759 |
| TBC1D10C | mRNA | UP | 1.500112 | 3.31383 | 19.55647 | 4.45E-64 | 2.20E-61 | 135.1572 |
| CD70 | mRNA | UP | 1.499437 | -0.48093 | 12.96918 | 1.78E-33 | 1.50E-31 | 65.23685 |
| GAPT | mRNA | UP | 1.499249 | 0.975627 | 15.56475 | 4.91E-45 | 7.18E-43 | 91.59568 |
| HCK | mRNA | UP | 1.499175 | 4.726956 | 18.97505 | 3.02E-61 | 1.17E-58 | 128.6487 |
| CD37 | mRNA | UP | 1.498985 | 4.806635 | 21.8931 | 1.48E-75 | 2.28E-72 | 161.4609 |
| PRF1 | mRNA | UP | 1.49841 | 3.498868 | 15.97938 | 5.88E-47 | 9.51E-45 | 95.94792 |
| SIRPB1 | mRNA | UP | 1.498038 | 1.699833 | 13.67456 | 1.59E-36 | 1.56E-34 | 72.15278 |
| OLR1 | mRNA | UP | 1.497615 | 4.419551 | 13.03993 | 8.87E-34 | 7.63E-32 | 65.74343 |
| ITGB2 | mRNA | UP | 1.497512 | 7.264514 | 18.1163 | 4.32E-57 | 1.25E-54 | 118.9837 |
| C11orf21 | mRNA | UP | 1.496247 | 0.482371 | 14.97189 | 2.55E-42 | 3.27E-40 | 85.39533 |
| HLA-DQB2 | mRNA | UP | 1.495823 | 5.202848 | 9.907583 | 2.71E-21 | 1.38E-19 | 37.16757 |
| PAX5 | mRNA | UP | 1.49374 | 1.361435 | 9.313745 | 3.59E-19 | 1.61E-17 | 32.61901 |
| IL2RB | mRNA | UP | 1.49371 | 4.256347 | 18.4755 | 7.99E-59 | 2.62E-56 | 123.1095 |
| KLRK1 | mRNA | UP | 1.49344 | -1.32136 | 11.76765 | 1.80E-28 | 1.23E-26 | 53.8592 |
| CLEC4A | mRNA | UP | 1.492372 | 2.101804 | 19.86887 | 1.32E-65 | 7.50E-63 | 138.6313 |
| AIF1 | mRNA | UP | 1.491852 | 4.645725 | 21.79445 | 4.55E-75 | 6.64E-72 | 160.3462 |
| PTPN22 | mRNA | UP | 1.491311 | 3.093733 | 18.30543 | 5.30E-58 | 1.65E-55 | 121.2447 |
| CD27 | mRNA | UP | 1.49063 | 2.804762 | 15.31682 | 6.78E-44 | 9.29E-42 | 88.96285 |
| GNLY | mRNA | UP | 1.489808 | 3.038944 | 10.88531 | 5.67E-25 | 3.39E-23 | 45.72065 |
| CD244 | mRNA | UP | 1.489022 | 0.600084 | 18.23751 | 1.13E-57 | 3.44E-55 | 120.4499 |
| CD80 | mRNA | UP | 1.488508 | 0.736028 | 18.78874 | 2.43E-60 | 8.59E-58 | 126.545 |
| CD1C | mRNA | UP | 1.487632 | 1.568691 | 10.26284 | 1.32E-22 | 7.12E-21 | 40.41465 |
| EVI2A | mRNA | UP | 1.485762 | 3.389148 | 20.59463 | 3.66E-69 | 2.64E-66 | 146.805 |
| SP140 | mRNA | UP | 1.485085 | 2.557444 | 19.09789 | 7.65E-62 | 3.10E-59 | 130.0342 |
| SIGLEC8 | mRNA | UP | 1.48439 | 0.565757 | 11.96901 | 2.72E-29 | 1.93E-27 | 55.68718 |
| IRF4 | mRNA | UP | 1.483514 | 3.108478 | 13.13631 | 3.42E-34 | 3.01E-32 | 66.76343 |
| CD226 | mRNA | UP | 1.483492 | 1.850206 | 20.95298 | 6.34E-71 | 5.44E-68 | 150.7875 |
| GPR31 | mRNA | UP | 1.483354 | -4.1954 | 11.52488 | 1.71E-27 | 1.14E-25 | 51.64561 |
| APOC4-APOC2 | mRNA | UP | 1.483205 | -3.01109 | 11.17587 | 4.16E-26 | 2.63E-24 | 48.50109 |
| ZBED2 | mRNA | UP | 1.482871 | 1.412989 | 12.36923 | 6.04E-31 | 4.60E-29 | 59.42565 |
| CTSE | mRNA | UP | 1.482716 | 6.14413 | 5.805851 | 1.12E-08 | 1.81E-07 | 8.48241 |
| C3AR1 | mRNA | UP | 1.481894 | 4.142783 | 18.1573 | 2.74E-57 | 8.17E-55 | 119.5922 |
| PIK3CG | mRNA | UP | 1.481655 | 2.642917 | 17.06825 | 4.46E-52 | 9.71E-50 | 107.6864 |
| PRKCB | mRNA | UP | 1.478907 | 3.688015 | 19.16822 | 3.48E-62 | 1.49E-59 | 130.8207 |
| LAIR1 | mRNA | UP | 1.478342 | 4.960787 | 20.21681 | 2.61E-67 | 1.66E-64 | 142.5468 |
| PTPRO | mRNA | UP | 1.47614 | 2.302235 | 16.57054 | 1.00E-49 | 1.93E-47 | 102.3067 |
| SAMSN1 | mRNA | UP | 1.475683 | 3.620444 | 20.07087 | 1.36E-66 | 8.34E-64 | 140.921 |
| CLC | mRNA | UP | 1.475427 | -3.72873 | 7.334247 | 8.74E-13 | 2.42E-11 | 18.38738 |
| LY86 | mRNA | UP | 1.471447 | 2.871709 | 17.50841 | 3.57E-54 | 9.02E-52 | 112.4821 |
| FCGR3A | mRNA | UP | 1.471314 | 6.24516 | 15.90297 | 1.33E-46 | 2.12E-44 | 94.99015 |
| IL10RA | mRNA | UP | 1.47074 | 5.067911 | 23.67294 | 2.48E-84 | 1.45E-80 | 181.5918 |
| CCL23 | mRNA | UP | 1.47069 | -0.59996 | 11.63342 | 6.26E-28 | 4.19E-26 | 52.61464 |
| CD74 | mRNA | UP | 1.470116 | 11.51128 | 17.73655 | 2.89E-55 | 7.74E-53 | 114.3565 |
| CD33 | mRNA | UP | 1.469645 | 2.148703 | 18.57892 | 2.52E-59 | 8.57E-57 | 124.2657 |
| CD200R1 | mRNA | UP | 1.468254 | 0.983245 | 19.93959 | 5.96E-66 | 3.45E-63 | 139.3712 |
| HSD11B1 | mRNA | UP | 1.46754 | 2.30271 | 16.33913 | 1.23E-48 | 2.17E-46 | 99.82033 |
| ICAM3 | mRNA | UP | 1.466131 | 1.492857 | 17.66049 | 6.68E-55 | 1.76E-52 | 114.1413 |
| HLA-DQB1 | mRNA | UP | 1.465386 | 7.802975 | 13.18403 | 2.14E-34 | 1.89E-32 | 66.87932 |
| LCP1 | mRNA | UP | 1.465278 | 7.3677 | 20.86307 | 1.75E-70 | 1.42E-67 | 149.7308 |
| P2RX7 | mRNA | UP | 1.463818 | 2.944564 | 18.18724 | 1.97E-57 | 5.92E-55 | 119.9402 |
| SLC6A20 | mRNA | UP | 1.462976 | 1.181197 | 6.589013 | 1.10E-10 | 2.36E-09 | 13.44055 |
| TNFAIP8L2 | mRNA | UP | 1.462125 | 2.403636 | 21.03848 | 2.41E-71 | 2.23E-68 | 151.7752 |
| GBP1 | mRNA | UP | 1.460079 | 5.842452 | 15.46662 | 1.39E-44 | 1.98E-42 | 90.39106 |
| HTRA4 | mRNA | UP | 1.459837 | 0.325881 | 13.19766 | 1.87E-34 | 1.66E-32 | 67.45935 |
| IGJ | mRNA | UP | 1.4592 | 8.257303 | 9.606195 | 3.32E-20 | 1.59E-18 | 34.37765 |
| PSTPIP1 | mRNA | UP | 1.459047 | 2.752633 | 22.01849 | 3.58E-76 | 6.52E-73 | 162.839 |
| FCGR1B | mRNA | UP | 1.458223 | -0.14135 | 16.50734 | 1.99E-49 | 3.72E-47 | 101.6 |
| C16orf54 | mRNA | UP | 1.456661 | 2.34961 | 16.73047 | 1.77E-50 | 3.59E-48 | 104.0307 |
| CD6 | mRNA | UP | 1.455997 | 3.58809 | 19.203 | 2.35E-62 | 1.04E-59 | 131.2092 |
| CLEC4E | mRNA | UP | 1.455202 | 1.21527 | 12.73238 | 1.80E-32 | 1.45E-30 | 62.90987 |
| HHLA2 | mRNA | UP | 1.455183 | 1.346433 | 4.762605 | 2.49E-06 | 2.69E-05 | 3.708113 |
| CD274 | mRNA | UP | 1.454969 | 3.271571 | 13.26062 | 9.99E-35 | 9.03E-33 | 67.97842 |
| WNT10A | mRNA | UP | 1.453751 | 1.529325 | 9.719669 | 1.30E-20 | 6.35E-19 | 35.88449 |
| ARHGAP9 | mRNA | UP | 1.453573 | 3.699319 | 22.58713 | 5.61E-79 | 1.21E-75 | 169.295 |
| CASP5 | mRNA | UP | 1.452718 | -1.52807 | 13.25895 | 1.02E-34 | 9.15E-33 | 68.06783 |
| CD1A | mRNA | UP | 1.451951 | 1.427164 | 6.663902 | 6.88E-11 | 1.52E-09 | 13.87804 |
| PIK3AP1 | mRNA | UP | 1.450645 | 4.678523 | 19.1517 | 4.18E-62 | 1.77E-59 | 130.6196 |
| TMIGD2 | mRNA | UP | 1.450571 | -1.27487 | 14.10254 | 2.06E-38 | 2.21E-36 | 76.47473 |
| HLA-DOB | mRNA | UP | 1.450118 | 2.638067 | 15.66445 | 1.70E-45 | 2.53E-43 | 92.62905 |
| CMKLR1 | mRNA | UP | 1.447693 | 3.985424 | 17.68689 | 5.00E-55 | 1.33E-52 | 114.4175 |
| LAX1 | mRNA | UP | 1.447291 | 1.974454 | 12.67006 | 3.31E-32 | 2.63E-30 | 62.28123 |
| DNASE2B | mRNA | UP | 1.447015 | -1.80122 | 8.987713 | 4.82E-18 | 2.01E-16 | 30.20921 |
| FPR3 | mRNA | UP | 1.446637 | 4.709268 | 17.37853 | 1.49E-53 | 3.57E-51 | 111.0157 |
| CD300C | mRNA | UP | 1.445356 | 1.345535 | 16.47056 | 2.97E-49 | 5.48E-47 | 101.2336 |
| NFAM1 | mRNA | UP | 1.444252 | 3.662301 | 18.5709 | 2.76E-59 | 9.31E-57 | 124.1781 |
| IL9R | mRNA | UP | 1.442732 | -2.11534 | 16.50911 | 1.95E-49 | 3.66E-47 | 101.536 |
| CLEC9A | mRNA | UP | 1.439027 | -1.67669 | 11.70391 | 3.25E-28 | 2.20E-26 | 53.27611 |
| SLFN14 | mRNA | UP | 1.438802 | -3.54208 | 11.63549 | 6.14E-28 | 4.12E-26 | 52.65389 |
| EBI3 | mRNA | UP | 1.438296 | 1.093261 | 16.50398 | 2.07E-49 | 3.84E-47 | 101.5904 |
| LCP2 | mRNA | UP | 1.438112 | 5.066325 | 25.31268 | 2.16E-92 | 6.31E-88 | 200.0808 |
| IL18RAP | mRNA | UP | 1.438037 | 0.179571 | 15.03532 | 1.31E-42 | 1.71E-40 | 86.05251 |
| ACSM5 | mRNA | UP | 1.432601 | -0.79893 | 10.94842 | 3.22E-25 | 1.95E-23 | 46.45367 |
| IL16 | mRNA | UP | 1.431271 | 4.201745 | 21.79674 | 4.43E-75 | 6.63E-72 | 160.371 |
| BIN2 | mRNA | UP | 1.430967 | 3.558206 | 23.11907 | 1.33E-81 | 4.58E-78 | 175.3011 |
| BCL2A1 | mRNA | UP | 1.429154 | 2.892457 | 16.25087 | 3.18E-48 | 5.46E-46 | 98.86457 |
| GPR25 | mRNA | UP | 1.428767 | -1.75233 | 11.06613 | 1.12E-25 | 6.95E-24 | 47.51412 |
| FPR2 | mRNA | UP | 1.424881 | 0.658788 | 10.89015 | 5.43E-25 | 3.26E-23 | 45.891 |
| FGD2 | mRNA | UP | 1.424243 | 3.607881 | 20.17429 | 4.23E-67 | 2.62E-64 | 142.0819 |
| LILRB1 | mRNA | UP | 1.424132 | 3.204866 | 19.33074 | 5.62E-63 | 2.54E-60 | 132.6351 |
| MPEG1 | mRNA | UP | 1.423537 | 5.185987 | 18.92251 | 5.44E-61 | 2.01E-58 | 128.0484 |
| MS4A4A | mRNA | UP | 1.422166 | 4.099357 | 16.44625 | 3.86E-49 | 7.08E-47 | 100.9255 |
| ITGAM | mRNA | UP | 1.421946 | 4.568202 | 15.54024 | 6.37E-45 | 9.27E-43 | 91.24395 |
| ASB2 | mRNA | UP | 1.421313 | 1.356153 | 18.09028 | 5.77E-57 | 1.66E-54 | 118.8554 |
| C15orf53 | mRNA | UP | 1.42008 | -4.13339 | 12.11389 | 6.90E-30 | 4.98E-28 | 57.07794 |
| SLCO2B1 | mRNA | UP | 1.419955 | 6.123005 | 16.63187 | 5.17E-50 | 1.02E-47 | 102.8224 |
| TNFSF8 | mRNA | UP | 1.417793 | 1.360394 | 17.87812 | 6.05E-56 | 1.66E-53 | 116.5238 |
| CCL8 | mRNA | UP | 1.41717 | 1.260174 | 9.882848 | 3.33E-21 | 1.68E-19 | 37.24379 |
| ABI3BP | mRNA | UP | 1.416972 | 4.149407 | 13.09261 | 5.27E-34 | 4.60E-32 | 66.27645 |
| TNIP3 | mRNA | UP | 1.41352 | 0.887744 | 10.1389 | 3.82E-22 | 2.02E-20 | 39.4024 |
| CST7 | mRNA | UP | 1.412201 | 2.973746 | 15.85744 | 2.17E-46 | 3.38E-44 | 94.6661 |
| PTAFR | mRNA | UP | 1.409663 | 4.773803 | 16.68981 | 2.75E-50 | 5.47E-48 | 103.5241 |
| PPP1R16B | mRNA | UP | 1.409513 | 3.649801 | 17.03537 | 6.38E-52 | 1.37E-49 | 107.3106 |
| KLRC1 | mRNA | UP | 1.409395 | -1.06672 | 10.62988 | 5.44E-24 | 3.09E-22 | 43.67245 |
| XXbac-BPG181M17.5 | mRNA | UP | 1.409287 | -4.02111 | 12.21787 | 2.57E-30 | 1.90E-28 | 58.05204 |
| HSF5 | mRNA | UP | 1.408779 | -3.58676 | 13.85019 | 2.69E-37 | 2.75E-35 | 73.90203 |
| LILRB2 | mRNA | UP | 1.406876 | 3.288701 | 17.44549 | 7.13E-54 | 1.75E-51 | 111.7885 |
| ALOX5AP | mRNA | UP | 1.405726 | 4.870301 | 14.73743 | 2.95E-41 | 3.59E-39 | 82.83107 |
| SIGLEC1 | mRNA | UP | 1.404952 | 4.48755 | 13.78175 | 5.37E-37 | 5.43E-35 | 73.10054 |
| KLHL6 | mRNA | UP | 1.403251 | 3.751464 | 17.2557 | 5.73E-53 | 1.34E-50 | 109.7063 |
| SIRPD | mRNA | UP | 1.403047 | -3.63286 | 12.79417 | 9.88E-33 | 8.09E-31 | 63.53318 |
| SIGLEC6 | mRNA | UP | 1.401947 | -0.80469 | 10.17582 | 2.79E-22 | 1.48E-20 | 39.78312 |
| CXorf65 | mRNA | UP | 1.401945 | -1.67681 | 13.10507 | 4.66E-34 | 4.07E-32 | 66.56081 |
| SPI1 | mRNA | UP | 1.401839 | 5.0177 | 18.93322 | 4.83E-61 | 1.84E-58 | 128.1735 |
| KLRC4 | mRNA | UP | 1.400015 | -3.73215 | 9.981383 | 1.45E-21 | 7.48E-20 | 38.20565 |
| CD86 | mRNA | UP | 1.3997 | 3.975144 | 20.1845 | 3.77E-67 | 2.36E-64 | 142.1965 |
| CIITA | mRNA | UP | 1.39889 | 5.331315 | 15.98492 | 5.54E-47 | 8.99E-45 | 95.92278 |
| FCER1G | mRNA | UP | 1.397886 | 5.403529 | 19.00833 | 2.08E-61 | 8.28E-59 | 128.9966 |
| FPR1 | mRNA | UP | 1.397815 | 3.249288 | 13.694 | 1.30E-36 | 1.29E-34 | 72.28734 |
| EXOC3L4 | mRNA | UP | 1.397443 | -0.18201 | 11.05095 | 1.28E-25 | 7.94E-24 | 47.34534 |
| CCL4L1 | mRNA | UP | 1.396268 | 1.765706 | 11.35408 | 8.20E-27 | 5.32E-25 | 49.98586 |
| C7 | mRNA | UP | 1.394988 | 5.278268 | 7.798888 | 3.50E-14 | 1.10E-12 | 20.98901 |
| LAPTM5 | mRNA | UP | 1.394525 | 7.965147 | 21.34904 | 7.12E-73 | 8.00E-70 | 155.1865 |
| MSR1 | mRNA | UP | 1.394111 | 5.368381 | 13.52974 | 6.80E-36 | 6.48E-34 | 70.51814 |
| COL6A6 | mRNA | UP | 1.392936 | 1.836501 | 8.532141 | 1.62E-16 | 6.16E-15 | 26.56454 |
| SAA4 | mRNA | UP | 1.390047 | -3.05409 | 6.976291 | 9.37E-12 | 2.30E-10 | 16.06493 |
| RASAL3 | mRNA | UP | 1.388989 | 3.637477 | 19.85371 | 1.57E-65 | 8.73E-63 | 138.4853 |
| SIGLEC7 | mRNA | UP | 1.387844 | 1.217967 | 16.32448 | 1.44E-48 | 2.51E-46 | 99.66668 |
| STAC | mRNA | UP | 1.387476 | 2.024828 | 9.698126 | 1.55E-20 | 7.56E-19 | 35.67732 |
| CYTIP | mRNA | UP | 1.387452 | 4.069103 | 19.54293 | 5.18E-64 | 2.54E-61 | 135.0025 |
| FCRL6 | mRNA | UP | 1.386566 | 0.595723 | 14.23128 | 5.51E-39 | 6.09E-37 | 77.78649 |
| SLA2 | mRNA | UP | 1.386392 | 1.823757 | 18.92478 | 5.31E-61 | 1.97E-58 | 128.0949 |
| SELPLG | mRNA | UP | 1.38631 | 4.962333 | 21.40293 | 3.86E-73 | 4.42E-70 | 155.9194 |
| RP11-812E19.9 | mRNA | UP | 1.385062 | -1.60224 | 7.62447 | 1.19E-13 | 3.58E-12 | 20.27884 |
| CD4 | mRNA | UP | 1.384682 | 6.516338 | 21.51937 | 1.03E-73 | 1.28E-70 | 157.1936 |
| HCST | mRNA | UP | 1.382821 | 2.21579 | 19.39419 | 2.76E-63 | 1.30E-60 | 133.3303 |
| GZMM | mRNA | UP | 1.382821 | 0.929057 | 12.91687 | 2.97E-33 | 2.48E-31 | 64.70435 |
| TYROBP | mRNA | UP | 1.379072 | 5.903455 | 17.19119 | 1.16E-52 | 2.68E-50 | 108.9103 |
| CSF1R | mRNA | UP | 1.378939 | 6.017411 | 17.34444 | 2.16E-53 | 5.18E-51 | 110.5773 |
| SUCNR1 | mRNA | UP | 1.378447 | 1.9772 | 10.93669 | 3.58E-25 | 2.16E-23 | 46.23759 |
| APOC2 | mRNA | UP | 1.376204 | -2.39523 | 11.075 | 1.03E-25 | 6.42E-24 | 47.59967 |
| KIR2DL4 | mRNA | UP | 1.375515 | -1.70196 | 7.926302 | 1.41E-14 | 4.61E-13 | 22.37173 |
| FLT3 | mRNA | UP | 1.375467 | -0.12706 | 14.68834 | 4.91E-41 | 5.90E-39 | 82.46192 |
| SLC1A3 | mRNA | UP | 1.374654 | 3.43522 | 15.09043 | 7.36E-43 | 9.74E-41 | 86.57195 |
| IL21 | mRNA | UP | 1.374225 | -4.70024 | 10.43998 | 2.86E-23 | 1.58E-21 | 42.05549 |
| PTGER4 | mRNA | UP | 1.373426 | 3.440622 | 18.26538 | 8.27E-58 | 2.54E-55 | 120.7989 |
| NLRP3 | mRNA | UP | 1.372672 | 2.012798 | 16.902 | 2.74E-51 | 5.71E-49 | 105.8871 |
| SLA | mRNA | UP | 1.371692 | 4.712483 | 20.62422 | 2.62E-69 | 1.91E-66 | 147.1367 |
| FAM216B | mRNA | UP | 1.370985 | 0.140287 | 5.614853 | 3.22E-08 | 4.84E-07 | 7.992387 |
| TIMD4 | mRNA | UP | 1.370824 | -1.00972 | 10.74888 | 1.90E-24 | 1.12E-22 | 44.70637 |
| GPR110 | mRNA | UP | 1.37043 | 4.50636 | 5.50529 | 5.83E-08 | 8.43E-07 | 7.047248 |
| GPR183 | mRNA | UP | 1.369863 | 3.903849 | 15.52213 | 7.72E-45 | 1.12E-42 | 91.08373 |
| SLC18A1 | mRNA | UP | 1.367249 | -3.56812 | 9.288215 | 4.42E-19 | 1.96E-17 | 32.58903 |
| ACAP1 | mRNA | UP | 1.36718 | 3.925581 | 19.50972 | 7.53E-64 | 3.66E-61 | 134.6325 |
| ITGAD | mRNA | UP | 1.366707 | -1.20865 | 10.72607 | 2.33E-24 | 1.36E-22 | 44.51189 |
| TNFRSF17 | mRNA | UP | 1.366606 | 1.071137 | 9.238969 | 6.56E-19 | 2.88E-17 | 32.04354 |
| FCRL5 | mRNA | UP | 1.365574 | 2.925236 | 8.980141 | 5.11E-18 | 2.13E-16 | 29.89398 |
| GPR171 | mRNA | UP | 1.364549 | 1.506339 | 12.11704 | 6.70E-30 | 4.84E-28 | 57.03859 |
| KCNJ10 | mRNA | UP | 1.363997 | -0.21403 | 10.23517 | 1.68E-22 | 9.00E-21 | 40.26414 |
| GPR141 | mRNA | UP | 1.363528 | -0.27852 | 14.45505 | 5.51E-40 | 6.39E-38 | 80.06794 |
| LAG3 | mRNA | UP | 1.362702 | 2.52715 | 12.67798 | 3.06E-32 | 2.44E-30 | 62.3329 |
| WAS | mRNA | UP | 1.362623 | 3.833977 | 23.02607 | 3.84E-81 | 1.18E-77 | 174.2594 |
| TLR4 | mRNA | UP | 1.362334 | 3.836471 | 17.10744 | 2.90E-52 | 6.45E-50 | 108.089 |
| TMEM150B | mRNA | UP | 1.362185 | 1.21623 | 14.64523 | 7.69E-41 | 9.16E-39 | 82.0148 |
| SIGLEC9 | mRNA | UP | 1.361551 | 2.006213 | 17.116 | 2.64E-52 | 5.96E-50 | 108.208 |
| CTSW | mRNA | UP | 1.358274 | 2.94198 | 12.63226 | 4.77E-32 | 3.77E-30 | 61.8721 |
| BCL11B | mRNA | UP | 1.357949 | 2.353534 | 13.85883 | 2.46E-37 | 2.52E-35 | 73.97943 |
| CYTH4 | mRNA | UP | 1.35747 | 4.285658 | 21.90059 | 1.36E-75 | 2.15E-72 | 161.5452 |
| OSCAR | mRNA | UP | 1.356673 | 3.086682 | 15.07877 | 8.32E-43 | 1.10E-40 | 86.46313 |
| APOBEC3G | mRNA | UP | 1.355831 | 3.937629 | 17.89815 | 4.84E-56 | 1.35E-53 | 116.7402 |
| NTNG1 | mRNA | UP | 1.355364 | -0.57095 | 6.954912 | 1.08E-11 | 2.62E-10 | 15.82558 |
| FGR | mRNA | UP | 1.354487 | 4.082591 | 18.83748 | 1.41E-60 | 5.08E-58 | 127.132 |
| FAIM3 | mRNA | UP | 1.354444 | 3.213963 | 16.71913 | 2.00E-50 | 4.03E-48 | 103.8945 |
| GIMAP7 | mRNA | UP | 1.354425 | 3.393681 | 21.09617 | 1.25E-71 | 1.20E-68 | 152.4536 |
| GIMAP4 | mRNA | UP | 1.353101 | 4.658219 | 23.94093 | 1.19E-85 | 8.67E-82 | 184.6144 |
| GFI1 | mRNA | UP | 1.35117 | 1.470158 | 16.87517 | 3.67E-51 | 7.56E-49 | 105.5949 |
| LILRB5 | mRNA | UP | 1.350492 | 2.28259 | 12.36698 | 6.17E-31 | 4.70E-29 | 59.36711 |
| MAP4K1 | mRNA | UP | 1.350249 | 3.413569 | 18.92956 | 5.03E-61 | 1.90E-58 | 128.1641 |
| MCOLN2 | mRNA | UP | 1.348578 | 2.0722 | 16.34137 | 1.20E-48 | 2.12E-46 | 99.84659 |
| LRMP | mRNA | UP | 1.347723 | 2.58112 | 15.34984 | 4.78E-44 | 6.63E-42 | 89.31532 |
| LGALS2 | mRNA | UP | 1.347671 | 1.046484 | 12.20195 | 2.99E-30 | 2.19E-28 | 57.85526 |
| CYP4F22 | mRNA | UP | 1.347164 | -2.01156 | 11.95263 | 3.17E-29 | 2.24E-27 | 55.57573 |
| GRAP2 | mRNA | UP | 1.346648 | 1.876398 | 17.16172 | 1.60E-52 | 3.66E-50 | 108.7045 |
| SIGLEC11 | mRNA | UP | 1.345328 | -0.62035 | 11.83294 | 9.75E-29 | 6.77E-27 | 54.45218 |
| FCRL2 | mRNA | UP | 1.345235 | 1.010253 | 8.850222 | 1.41E-17 | 5.69E-16 | 29.0226 |
| RCSD1 | mRNA | UP | 1.343731 | 3.981633 | 21.16107 | 6.00E-72 | 6.04E-69 | 153.1922 |
| ARHGAP15 | mRNA | UP | 1.343475 | 2.946776 | 21.95656 | 7.22E-76 | 1.24E-72 | 162.1481 |
| NELL2 | mRNA | UP | 1.343367 | 2.117109 | 11.16263 | 4.69E-26 | 2.95E-24 | 48.24258 |
| FERMT3 | mRNA | UP | 1.34277 | 5.014987 | 20.90556 | 1.08E-70 | 9.04E-68 | 150.3034 |
| CD180 | mRNA | UP | 1.341882 | 2.928176 | 16.38681 | 7.34E-49 | 1.33E-46 | 100.3213 |
| CTD-2207O23.3 | mRNA | UP | 1.341665 | -4.47404 | 10.17918 | 2.71E-22 | 1.44E-20 | 39.85144 |
| GPR34 | mRNA | UP | 1.341427 | 2.537562 | 13.73343 | 8.75E-37 | 8.71E-35 | 72.7131 |
| CCL3 | mRNA | UP | 1.339363 | 2.55282 | 13.39792 | 2.54E-35 | 2.36E-33 | 69.36875 |
| PARVG | mRNA | UP | 1.338293 | 4.071982 | 20.03047 | 2.14E-66 | 1.29E-63 | 140.4663 |
| CD5L | mRNA | UP | 1.337793 | -3.59708 | 8.442226 | 3.19E-16 | 1.19E-14 | 26.13008 |
| SIRPB2 | mRNA | UP | 1.336487 | 2.208359 | 16.37591 | 8.25E-49 | 1.48E-46 | 100.2157 |
| KCNA3 | mRNA | UP | 1.335467 | 1.391547 | 10.06541 | 7.14E-22 | 3.71E-20 | 38.75757 |
| CPA3 | mRNA | UP | 1.335148 | 3.732055 | 8.917662 | 8.34E-18 | 3.42E-16 | 29.34878 |
| IL10 | mRNA | UP | 1.333291 | -0.39373 | 14.82869 | 1.14E-41 | 1.42E-39 | 83.90606 |
| GPR114 | mRNA | UP | 1.333161 | 1.875681 | 12.59301 | 6.98E-32 | 5.46E-30 | 61.54437 |
| PTGDR | mRNA | UP | 1.332945 | -0.94826 | 14.56054 | 1.85E-40 | 2.17E-38 | 81.13959 |
| LY9 | mRNA | UP | 1.332537 | 2.062293 | 15.12724 | 5.00E-43 | 6.65E-41 | 86.99725 |
| CHRDL1 | mRNA | UP | 1.332474 | 3.195887 | 7.875923 | 2.03E-14 | 6.53E-13 | 21.71045 |
| MAP1LC3C | mRNA | UP | 1.331954 | 0.145474 | 8.670934 | 5.63E-17 | 2.19E-15 | 27.71055 |
| APOL3 | mRNA | UP | 1.330326 | 4.846117 | 19.06137 | 1.15E-61 | 4.63E-59 | 129.6071 |
| ZBP1 | mRNA | UP | 1.330192 | 2.062386 | 13.4213 | 2.01E-35 | 1.88E-33 | 69.61959 |
| IVL | mRNA | UP | 1.329896 | -0.41471 | 3.999635 | 7.28E-05 | 0.000561 | 0.63552 |
| CCDC141 | mRNA | UP | 1.328733 | -0.52361 | 10.34723 | 6.39E-23 | 3.48E-21 | 41.22705 |
| SLFN12L | mRNA | UP | 1.325501 | 0.940345 | 16.60274 | 7.09E-50 | 1.37E-47 | 102.6502 |
| DHRS9 | mRNA | UP | 1.322946 | 2.332067 | 8.851006 | 1.40E-17 | 5.66E-16 | 28.94115 |
| C10orf128 | mRNA | UP | 1.320804 | 3.182785 | 20.32004 | 8.15E-68 | 5.41E-65 | 143.7154 |
| IL27 | mRNA | UP | 1.320406 | -2.49104 | 12.56741 | 8.95E-32 | 6.98E-30 | 61.36625 |
| STX11 | mRNA | UP | 1.319094 | 2.441127 | 18.13165 | 3.65E-57 | 1.06E-54 | 119.3271 |
| RGS13 | mRNA | UP | 1.314895 | -0.47438 | 10.92678 | 3.91E-25 | 2.36E-23 | 46.25405 |
| FCGR2B | mRNA | UP | 1.314489 | 3.561064 | 14.90094 | 5.36E-42 | 6.80E-40 | 84.59357 |
| CEACAM4 | mRNA | UP | 1.312713 | -0.04303 | 10.70274 | 2.86E-24 | 1.66E-22 | 44.27518 |
| MMP7 | mRNA | UP | 1.312685 | 5.22698 | 6.886551 | 1.67E-11 | 3.97E-10 | 14.93292 |
| RASGRP2 | mRNA | UP | 1.312422 | 2.409074 | 15.29245 | 8.77E-44 | 1.20E-41 | 88.71744 |
| CADM3 | mRNA | UP | 1.30961 | 0.751076 | 9.285352 | 4.52E-19 | 2.00E-17 | 32.42889 |
| CHI3L1 | mRNA | UP | 1.309314 | 6.344418 | 8.602877 | 9.47E-17 | 3.64E-15 | 26.71394 |
| PPP2R2B | mRNA | UP | 1.309015 | 0.228778 | 14.83787 | 1.04E-41 | 1.30E-39 | 84.00642 |
| CLEC1B | mRNA | UP | 1.308828 | -2.91486 | 10.97941 | 2.44E-25 | 1.49E-23 | 46.75646 |
| HPGDS | mRNA | UP | 1.305796 | 1.381306 | 9.827151 | 5.31E-21 | 2.64E-19 | 36.7762 |
| CD209 | mRNA | UP | 1.305527 | 2.691917 | 11.95907 | 2.98E-29 | 2.12E-27 | 55.50211 |
| OR52N4 | mRNA | UP | 1.304458 | -3.65314 | 9.659971 | 2.13E-20 | 1.03E-18 | 35.56705 |
| RARRES3 | mRNA | UP | 1.304105 | 5.885149 | 11.90072 | 5.16E-29 | 3.63E-27 | 54.7344 |
| PI16 | mRNA | UP | 1.302471 | -1.33603 | 7.321555 | 9.52E-13 | 2.62E-11 | 18.23568 |
| SFTPA1 | mRNA | UP | 1.30205 | 8.986158 | 4.183055 | 3.38E-05 | 0.000283 | 0.421796 |
| GMFG | mRNA | UP | 1.301536 | 4.203379 | 22.09993 | 1.42E-76 | 2.67E-73 | 163.7976 |
| MYO1G | mRNA | UP | 1.30015 | 4.813913 | 11.13127 | 6.22E-26 | 3.89E-24 | 47.78211 |
| CLEC12B | mRNA | UP | 1.299821 | -3.03231 | 9.239161 | 6.55E-19 | 2.87E-17 | 32.1972 |
| L1CAM | mRNA | UP | 1.299264 | 0.254098 | 7.785958 | 3.84E-14 | 1.20E-12 | 21.29422 |
| CEACAM21 | mRNA | UP | 1.298351 | 1.049802 | 14.46372 | 5.04E-40 | 5.86E-38 | 80.15328 |
| C1orf162 | mRNA | UP | 1.297875 | 4.020011 | 19.20152 | 2.39E-62 | 1.05E-59 | 131.1884 |
| DAZL | mRNA | UP | 1.297719 | -4.29655 | 8.395322 | 4.54E-16 | 1.67E-14 | 25.77865 |
| CORO1A | mRNA | UP | 1.295983 | 6.022997 | 20.80238 | 3.49E-70 | 2.71E-67 | 149.1113 |
| NUGGC | mRNA | UP | 1.293114 | 0.654709 | 11.02788 | 1.58E-25 | 9.70E-24 | 47.11004 |
| APOC1 | mRNA | UP | 1.290488 | 6.078237 | 12.15976 | 4.46E-30 | 3.25E-28 | 57.14913 |
| APOBEC3H | mRNA | UP | 1.289948 | 0.113275 | 13.46997 | 1.24E-35 | 1.16E-33 | 70.14756 |
| APBB1IP | mRNA | UP | 1.289759 | 3.968971 | 18.33293 | 3.90E-58 | 1.22E-55 | 121.5371 |
| GLB1L3 | mRNA | UP | 1.289562 | 1.318329 | 4.161676 | 3.70E-05 | 0.000307 | 1.13454 |
| BIRC3 | mRNA | UP | 1.288275 | 6.028506 | 12.29125 | 1.27E-30 | 9.60E-29 | 58.39879 |
| MEOX1 | mRNA | UP | 1.288204 | -0.53241 | 10.51676 | 1.47E-23 | 8.16E-22 | 42.68007 |
| TREM2 | mRNA | UP | 1.28811 | 4.090404 | 12.68996 | 2.73E-32 | 2.18E-30 | 62.36173 |
| FAM92B | mRNA | UP | 1.286032 | 0.061734 | 6.741218 | 4.23E-11 | 9.58E-10 | 14.45055 |
| KCNK13 | mRNA | UP | 1.285045 | 0.323466 | 13.49839 | 9.31E-36 | 8.84E-34 | 70.42699 |
| CSF2 | mRNA | UP | 1.284502 | -0.24187 | 7.576538 | 1.66E-13 | 4.93E-12 | 19.8859 |
| LRRC25 | mRNA | UP | 1.284242 | 3.031514 | 16.62615 | 5.50E-50 | 1.07E-47 | 102.8946 |
| CXorf21 | mRNA | UP | 1.283344 | 1.679244 | 17.19114 | 1.16E-52 | 2.68E-50 | 109.0232 |
| PILRA | mRNA | UP | 1.281943 | 3.159546 | 17.17721 | 1.35E-52 | 3.11E-50 | 108.864 |
| PARP15 | mRNA | UP | 1.281377 | 2.429557 | 12.03328 | 1.48E-29 | 1.06E-27 | 56.20981 |
| LILRA1 | mRNA | UP | 1.281313 | -0.03138 | 15.88668 | 1.59E-46 | 2.50E-44 | 94.98549 |
| KLRC2 | mRNA | UP | 1.281017 | -2.79132 | 7.222233 | 1.85E-12 | 4.94E-11 | 17.63885 |
| MATK | mRNA | UP | 1.280532 | 1.889409 | 15.51486 | 8.33E-45 | 1.20E-42 | 91.06418 |
| PSMB9 | mRNA | UP | 1.279759 | 5.669656 | 14.49012 | 3.83E-40 | 4.48E-38 | 80.22645 |
| ZNF804A | mRNA | UP | 1.278248 | -1.05057 | 13.02341 | 1.04E-33 | 8.92E-32 | 65.76664 |
| KIR3DL2 | mRNA | UP | 1.278003 | -3.88661 | 8.091541 | 4.28E-15 | 1.46E-13 | 23.58927 |
| GIMAP1 | mRNA | UP | 1.276503 | 2.639073 | 20.26546 | 1.51E-67 | 9.79E-65 | 143.0921 |
| PIK3R5 | mRNA | UP | 1.275796 | 3.55936 | 18.69886 | 6.62E-60 | 2.27E-57 | 125.5989 |
| CPVL | mRNA | UP | 1.274746 | 4.715204 | 13.2449 | 1.17E-34 | 1.05E-32 | 67.73776 |
| GIMAP6 | mRNA | UP | 1.273566 | 4.184262 | 19.4702 | 1.17E-63 | 5.66E-61 | 134.1874 |
| MYO1F | mRNA | UP | 1.272923 | 4.863179 | 21.31102 | 1.10E-72 | 1.16E-69 | 154.8822 |
| TSPAN32 | mRNA | UP | 1.272437 | 0.467034 | 13.12015 | 4.02E-34 | 3.51E-32 | 66.69712 |
| KEL | mRNA | UP | 1.270247 | -1.37599 | 10.41201 | 3.65E-23 | 2.00E-21 | 41.80381 |
| MS4A7 | mRNA | UP | 1.269598 | 4.631635 | 15.20122 | 2.29E-43 | 3.09E-41 | 87.67427 |
| ACKR1 | mRNA | UP | 1.268608 | 2.727079 | 7.770624 | 4.28E-14 | 1.34E-12 | 21.01248 |
| LTF | mRNA | UP | 1.267189 | 5.080837 | 5.694152 | 2.09E-08 | 3.23E-07 | 7.987356 |
| CCL22 | mRNA | UP | 1.266558 | 3.136115 | 10.00575 | 1.18E-21 | 6.11E-20 | 38.14767 |
| GIMAP2 | mRNA | UP | 1.265706 | 3.286848 | 17.76235 | 2.17E-55 | 5.84E-53 | 115.2599 |
| CAMK4 | mRNA | UP | 1.26547 | 1.773845 | 15.81176 | 3.53E-46 | 5.40E-44 | 94.20282 |
| RUNX3 | mRNA | UP | 1.263409 | 4.00902 | 17.06393 | 4.67E-52 | 1.01E-49 | 107.6104 |
| CCR1 | mRNA | UP | 1.263014 | 3.953071 | 15.31812 | 6.69E-44 | 9.19E-42 | 88.93385 |
| ARHGAP30 | mRNA | UP | 1.262833 | 5.039074 | 21.53556 | 8.58E-74 | 1.09E-70 | 157.417 |
| TMEM156 | mRNA | UP | 1.261416 | 1.946703 | 9.788715 | 7.32E-21 | 3.63E-19 | 36.42527 |
| LILRA6 | mRNA | UP | 1.261199 | 1.526034 | 13.60122 | 3.32E-36 | 3.21E-34 | 71.4252 |
| BEND4 | mRNA | UP | 1.260649 | -2.15054 | 8.069076 | 5.04E-15 | 1.71E-13 | 23.39744 |
| FOXP3 | mRNA | UP | 1.260604 | 2.259668 | 16.82228 | 6.52E-51 | 1.33E-48 | 105.0235 |
| ALOX5 | mRNA | UP | 1.259466 | 5.268287 | 14.81793 | 1.27E-41 | 1.58E-39 | 83.63997 |
| RAC2 | mRNA | UP | 1.25945 | 5.89268 | 19.18326 | 2.94E-62 | 1.27E-59 | 130.9275 |
| CLNK | mRNA | UP | 1.259313 | 0.024259 | 12.23845 | 2.11E-30 | 1.57E-28 | 58.22865 |
| CLEC7A | mRNA | UP | 1.257556 | 4.209694 | 14.10722 | 1.96E-38 | 2.11E-36 | 76.40555 |
| ITGB7 | mRNA | UP | 1.257462 | 2.46462 | 16.3819 | 7.74E-49 | 1.39E-46 | 100.2768 |
| CASP1 | mRNA | UP | 1.256891 | 4.397279 | 18.75044 | 3.72E-60 | 1.29E-57 | 126.1573 |
| SLC7A7 | mRNA | UP | 1.255643 | 4.882571 | 18.45819 | 9.68E-59 | 3.16E-56 | 122.8988 |
| PLD4 | mRNA | UP | 1.255354 | 1.978944 | 11.14877 | 5.31E-26 | 3.34E-24 | 48.12561 |
| GPR82 | mRNA | UP | 1.255007 | -0.12893 | 13.12173 | 3.96E-34 | 3.47E-32 | 66.72129 |
| FAM78A | mRNA | UP | 1.254466 | 3.296042 | 21.00607 | 3.47E-71 | 3.12E-68 | 151.4364 |
| BRINP1 | mRNA | UP | 1.253673 | -0.17706 | 4.523139 | 7.57E-06 | 7.37E-05 | 2.765004 |
| MILR1 | mRNA | UP | 1.25313 | 2.487554 | 14.5881 | 1.39E-40 | 1.64E-38 | 81.39835 |
| CSF2RA | mRNA | UP | 1.250221 | 2.750779 | 13.90021 | 1.62E-37 | 1.67E-35 | 74.38093 |
| DNASE1L3 | mRNA | UP | 1.250182 | 0.197468 | 7.922717 | 1.45E-14 | 4.73E-13 | 22.25331 |
| FMO2 | mRNA | UP | 1.250144 | 4.342738 | 10.54109 | 1.18E-23 | 6.64E-22 | 42.61657 |
| VPREB3 | mRNA | UP | 1.25014 | 0.046024 | 9.484774 | 8.98E-20 | 4.15E-18 | 34.05738 |
| C1orf186 | mRNA | UP | 1.24808 | 1.084744 | 9.706395 | 1.45E-20 | 7.07E-19 | 35.80089 |
| IL26 | mRNA | UP | 1.246591 | -3.96199 | 9.337422 | 2.97E-19 | 1.33E-17 | 32.97921 |
| PNOC | mRNA | UP | 1.246496 | 0.049134 | 9.249189 | 6.04E-19 | 2.66E-17 | 32.17954 |
| PRR33 | mRNA | UP | 1.246279 | -0.86162 | 10.63575 | 5.17E-24 | 2.94E-22 | 43.71818 |
| RSPO1 | mRNA | UP | 1.245313 | -1.55132 | 8.423157 | 3.69E-16 | 1.36E-14 | 25.9415 |
| P2RX5 | mRNA | UP | 1.244596 | 0.664523 | 10.45802 | 2.44E-23 | 1.35E-21 | 42.12811 |
| IL1B | mRNA | UP | 1.243924 | 2.396903 | 11.0406 | 1.41E-25 | 8.70E-24 | 47.13619 |
| IL2 | mRNA | UP | 1.243604 | -4.13954 | 10.15349 | 3.37E-22 | 1.78E-20 | 39.64204 |
| MYBPC2 | mRNA | UP | 1.242576 | -0.13847 | 8.948719 | 6.54E-18 | 2.70E-16 | 29.84325 |
| MMP9 | mRNA | UP | 1.242029 | 5.568187 | 9.247707 | 6.11E-19 | 2.69E-17 | 31.7719 |
| TMEM236 | mRNA | UP | 1.24136 | 0.122491 | 10.10747 | 4.99E-22 | 2.62E-20 | 39.17369 |
| TREML1 | mRNA | UP | 1.240044 | -1.03671 | 12.77516 | 1.19E-32 | 9.70E-31 | 63.36156 |
| GAB3 | mRNA | UP | 1.239795 | 2.613024 | 19.77999 | 3.60E-65 | 1.96E-62 | 137.6502 |
| CD40 | mRNA | UP | 1.239342 | 4.801696 | 15.85165 | 2.31E-46 | 3.58E-44 | 94.53196 |
| TMEM52B | mRNA | UP | 1.239241 | 0.03244 | 13.74991 | 7.41E-37 | 7.44E-35 | 72.93551 |
| CCL21 | mRNA | UP | 1.23755 | 4.756085 | 8.959624 | 6.01E-18 | 2.49E-16 | 29.58721 |
| MZB1 | mRNA | UP | 1.237067 | 4.593297 | 8.308422 | 8.68E-16 | 3.12E-14 | 24.69396 |
| DPEP2 | mRNA | UP | 1.235724 | 2.01754 | 15.0608 | 1.00E-42 | 1.32E-40 | 86.30537 |
| CYSLTR2 | mRNA | UP | 1.235632 | 0.739856 | 15.74689 | 7.06E-46 | 1.06E-43 | 93.51704 |
| CCL25 | mRNA | UP | 1.235357 | -3.02144 | 7.890661 | 1.82E-14 | 5.91E-13 | 22.16184 |
| HTR7 | mRNA | UP | 1.234352 | -1.38133 | 11.4324 | 4.00E-27 | 2.62E-25 | 50.79583 |
| ENTHD1 | mRNA | UP | 1.234197 | -2.40679 | 8.45559 | 2.89E-16 | 1.08E-14 | 26.20748 |
| HAMP | mRNA | UP | 1.232735 | 0.066903 | 9.29644 | 4.13E-19 | 1.84E-17 | 32.55258 |
| POU2AF1 | mRNA | UP | 1.232572 | 3.882988 | 8.740277 | 3.31E-17 | 1.30E-15 | 27.97753 |
| MS4A4E | mRNA | UP | 1.230773 | -1.59907 | 10.7021 | 2.88E-24 | 1.67E-22 | 44.31089 |
| ITGAX | mRNA | UP | 1.230273 | 5.142485 | 16.00002 | 4.71E-47 | 7.69E-45 | 96.09418 |
| NCF4 | mRNA | UP | 1.228452 | 3.935481 | 17.66615 | 6.28E-55 | 1.66E-52 | 114.1909 |
| MS4A2 | mRNA | UP | 1.228239 | 1.286192 | 8.338674 | 6.93E-16 | 2.51E-14 | 25.17125 |
| NLRC3 | mRNA | UP | 1.228018 | 2.868471 | 18.11643 | 4.32E-57 | 1.25E-54 | 119.1594 |
| DOCK8 | mRNA | UP | 1.227902 | 5.294874 | 18.20189 | 1.67E-57 | 5.06E-55 | 120.045 |
| BFSP2 | mRNA | UP | 1.226836 | -2.22695 | 9.508469 | 7.40E-20 | 3.44E-18 | 34.32478 |
| RETN | mRNA | UP | 1.225564 | -0.51044 | 6.697689 | 5.57E-11 | 1.24E-09 | 14.21856 |
| RNASE6 | mRNA | UP | 1.225512 | 3.763135 | 17.57994 | 1.62E-54 | 4.16E-52 | 113.25 |
| MCEMP1 | mRNA | UP | 1.224102 | 1.945023 | 6.986615 | 8.76E-12 | 2.16E-10 | 15.85218 |
| KIAA0226L | mRNA | UP | 1.223186 | 1.940761 | 16.38154 | 7.77E-49 | 1.40E-46 | 100.2782 |
| PLA2G2A | mRNA | UP | 1.221537 | 0.670189 | 6.231353 | 9.64E-10 | 1.82E-08 | 11.36062 |
| RGS1 | mRNA | UP | 1.22042 | 6.035089 | 11.49832 | 2.18E-27 | 1.44E-25 | 51.0034 |
| CD72 | mRNA | UP | 1.219445 | 2.36368 | 17.03907 | 6.13E-52 | 1.32E-49 | 107.3717 |
| HDC | mRNA | UP | 1.219333 | 0.794748 | 9.083404 | 2.26E-18 | 9.56E-17 | 30.83784 |
| SAMD9L | mRNA | UP | 1.21691 | 5.113247 | 14.05086 | 3.49E-38 | 3.71E-36 | 75.77752 |
| NCF2 | mRNA | UP | 1.216327 | 5.069843 | 15.51779 | 8.08E-45 | 1.17E-42 | 90.97907 |
| TNF | mRNA | UP | 1.216193 | 0.317687 | 10.23766 | 1.64E-22 | 8.82E-21 | 40.26333 |
| GNGT2 | mRNA | UP | 1.215108 | 1.014349 | 18.13826 | 3.39E-57 | 1.00E-54 | 119.3736 |
| CCL11 | mRNA | UP | 1.2148 | 0.194633 | 7.829637 | 2.82E-14 | 8.97E-13 | 21.60154 |
| VNN2 | mRNA | UP | 1.214468 | 1.980139 | 11.03268 | 1.51E-25 | 9.31E-24 | 47.08894 |
| ANXA8L1 | mRNA | UP | 1.213783 | -0.91644 | 6.487293 | 2.06E-10 | 4.24E-09 | 12.96844 |
| HLA-F | mRNA | UP | 1.212804 | 6.498617 | 14.91173 | 4.79E-42 | 6.09E-40 | 84.52606 |
| ERICH3 | mRNA | UP | 1.212124 | -0.51647 | 4.676237 | 3.74E-06 | 3.89E-05 | 3.463321 |
| KCNJ5 | mRNA | UP | 1.211813 | 2.197202 | 9.690584 | 1.65E-20 | 8.03E-19 | 35.60409 |
| FAM159A | mRNA | UP | 1.211728 | -0.40974 | 14.69857 | 4.42E-41 | 5.34E-39 | 82.56437 |
| CHRNA6 | mRNA | UP | 1.211634 | -2.45022 | 11.01087 | 1.84E-25 | 1.13E-23 | 47.03158 |
| 1-Sep | mRNA | UP | 1.210879 | 3.403786 | 17.15026 | 1.82E-52 | 4.13E-50 | 108.5657 |
| PRAM1 | mRNA | UP | 1.210353 | 2.034348 | 13.72091 | 9.93E-37 | 9.85E-35 | 72.60557 |
| CCNA1 | mRNA | UP | 1.20822 | -1.11294 | 6.59948 | 1.03E-10 | 2.22E-09 | 13.65383 |
| CD14 | mRNA | UP | 1.208141 | 6.281314 | 14.54506 | 2.17E-40 | 2.55E-38 | 80.74587 |
| P2RY8 | mRNA | UP | 1.208071 | 1.807055 | 14.24309 | 4.88E-39 | 5.42E-37 | 77.8859 |
| PIK3R6 | mRNA | UP | 1.207188 | 1.691846 | 16.0731 | 2.15E-47 | 3.57E-45 | 96.98145 |
| ARHGEF6 | mRNA | UP | 1.20616 | 4.372848 | 20.25206 | 1.76E-67 | 1.13E-64 | 142.9527 |
| SLC37A2 | mRNA | UP | 1.204683 | 3.691203 | 13.88933 | 1.81E-37 | 1.86E-35 | 74.22802 |
| TPSAB1 | mRNA | UP | 1.204064 | 3.160662 | 8.740107 | 3.31E-17 | 1.31E-15 | 28.03322 |
| ATP6V0D2 | mRNA | UP | 1.203377 | 0.939381 | 8.714839 | 4.02E-17 | 1.57E-15 | 27.99575 |
| ANXA8 | mRNA | UP | 1.202027 | -0.96509 | 5.314233 | 1.60E-07 | 2.16E-06 | 6.519569 |
| IL5RA | mRNA | UP | 1.200535 | -0.1545 | 7.714786 | 6.34E-14 | 1.95E-12 | 20.8263 |
| CXCL14 | mRNA | UP | 1.19856 | 4.805184 | 4.963254 | 9.44E-07 | 1.11E-05 | 4.324453 |
| SUSD3 | mRNA | UP | 1.198003 | 2.122435 | 16.22108 | 4.38E-48 | 7.44E-46 | 98.55808 |
| SFTPA2 | mRNA | UP | 1.197618 | 9.433345 | 3.990283 | 7.56E-05 | 0.00058 | -0.40083 |
| CARD16 | mRNA | UP | 1.197253 | 2.665498 | 17.11488 | 2.68E-52 | 6.01E-50 | 108.1927 |
| LAT2 | mRNA | UP | 1.197201 | 4.024713 | 17.84081 | 9.13E-56 | 2.49E-53 | 116.1072 |
| SLC28A3 | mRNA | UP | 1.195066 | 1.596158 | 7.962281 | 1.09E-14 | 3.59E-13 | 22.43967 |
| LPXN | mRNA | UP | 1.194907 | 4.392342 | 24.79501 | 7.49E-90 | 8.75E-86 | 194.2383 |
| CCL1 | mRNA | UP | 1.194482 | -4.25082 | 8.181428 | 2.22E-15 | 7.76E-14 | 24.22922 |
| VSTM1 | mRNA | UP | 1.193878 | -2.93331 | 6.948455 | 1.12E-11 | 2.73E-10 | 15.88654 |
| XCR1 | mRNA | UP | 1.19314 | 0.291832 | 9.591965 | 3.73E-20 | 1.78E-18 | 34.91088 |
| LSP1 | mRNA | UP | 1.193063 | 5.973726 | 16.392 | 6.94E-49 | 1.26E-46 | 100.2444 |
| POU3F1 | mRNA | UP | 1.192756 | -1.89935 | 10.40722 | 3.80E-23 | 2.09E-21 | 41.77348 |
| COL19A1 | mRNA | UP | 1.192641 | -2.93033 | 7.820821 | 3.00E-14 | 9.51E-13 | 21.67318 |
| ANKRD22 | mRNA | UP | 1.191636 | 3.550965 | 9.067832 | 2.56E-18 | 1.08E-16 | 30.52811 |
| RNASE2 | mRNA | UP | 1.19152 | 0.032993 | 10.20737 | 2.13E-22 | 1.13E-20 | 40.01911 |
| KCNA2 | mRNA | UP | 1.191192 | -2.75678 | 8.930695 | 7.53E-18 | 3.10E-16 | 29.79387 |
| HLA-B | mRNA | UP | 1.191063 | 10.86326 | 16.26447 | 2.75E-48 | 4.74E-46 | 98.40232 |
| SLC15A3 | mRNA | UP | 1.19031 | 5.266028 | 16.97085 | 1.29E-51 | 2.72E-49 | 106.5458 |
| CD300LG | mRNA | UP | 1.188795 | -2.54039 | 5.95461 | 4.84E-09 | 8.30E-08 | 9.979207 |
| IGLL5 | mRNA | UP | 1.18852 | 6.081315 | 7.381644 | 6.34E-13 | 1.77E-11 | 18.06071 |
| RARRES1 | mRNA | UP | 1.187574 | 4.312683 | 9.859292 | 4.06E-21 | 2.04E-19 | 36.83989 |
| WNT7A | mRNA | UP | 1.187471 | -0.66088 | 4.919273 | 1.17E-06 | 1.35E-05 | 4.584976 |
| DOCK10 | mRNA | UP | 1.186573 | 4.344475 | 16.18328 | 6.58E-48 | 1.11E-45 | 98.09274 |
| IL24 | mRNA | UP | 1.186263 | 0.958466 | 14.24531 | 4.77E-39 | 5.31E-37 | 77.92516 |
| GPR15 | mRNA | UP | 1.185124 | -1.40495 | 6.511329 | 1.77E-10 | 3.69E-09 | 13.13808 |
| KBTBD8 | mRNA | UP | 1.185066 | 1.430319 | 16.69577 | 2.58E-50 | 5.14E-48 | 103.6573 |
| CLDN18 | mRNA | UP | 1.185038 | 3.055549 | 3.967269 | 8.30E-05 | 0.00063 | 0.214683 |
| GPR55 | mRNA | UP | 1.184843 | -0.77581 | 12.74702 | 1.56E-32 | 1.27E-30 | 63.08917 |
| SRGN | mRNA | UP | 1.184754 | 6.825265 | 13.92747 | 1.23E-37 | 1.28E-35 | 74.39515 |
| CLEC4G | mRNA | UP | 1.184179 | -2.04957 | 7.932375 | 1.35E-14 | 4.42E-13 | 22.42708 |
| TNFRSF8 | mRNA | UP | 1.18363 | 0.449221 | 14.20238 | 7.41E-39 | 8.18E-37 | 77.49388 |
| LRRC18 | mRNA | UP | 1.183565 | -2.34882 | 6.5851 | 1.13E-10 | 2.42E-09 | 13.62308 |
| ABI3 | mRNA | UP | 1.181764 | 3.687845 | 20.87968 | 1.45E-70 | 1.20E-67 | 150.0177 |
| CFP | mRNA | UP | 1.180459 | 1.406025 | 13.61081 | 3.01E-36 | 2.92E-34 | 71.52403 |
| PSMA8 | mRNA | UP | 1.179921 | -4.50797 | 9.114955 | 1.76E-18 | 7.51E-17 | 31.2209 |
| TRPV2 | mRNA | UP | 1.179054 | 4.398231 | 18.98248 | 2.78E-61 | 1.08E-58 | 128.7394 |
| NME8 | mRNA | UP | 1.176618 | -1.53914 | 13.16294 | 2.63E-34 | 2.32E-32 | 67.12695 |
| VCAM1 | mRNA | UP | 1.175261 | 4.824817 | 11.70817 | 3.13E-28 | 2.12E-26 | 53.03226 |
| ART4 | mRNA | UP | 1.174951 | -0.4966 | 8.564575 | 1.27E-16 | 4.85E-15 | 26.94555 |
| RUFY4 | mRNA | UP | 1.171739 | -0.19739 | 8.210251 | 1.79E-15 | 6.30E-14 | 24.32691 |
| CX3CR1 | mRNA | UP | 1.171713 | 1.487567 | 8.391613 | 4.67E-16 | 1.71E-14 | 25.54632 |
| GTSF1L | mRNA | UP | 1.171668 | -4.46698 | 9.615009 | 3.09E-20 | 1.48E-18 | 35.19628 |
| CD7 | mRNA | UP | 1.170969 | 3.274337 | 11.63913 | 5.94E-28 | 3.99E-26 | 52.50267 |
| 1-Mar | mRNA | UP | 1.169404 | 3.853625 | 19.67711 | 1.15E-64 | 5.97E-62 | 136.5062 |
| ABCA6 | mRNA | UP | 1.169336 | 2.120277 | 13.06688 | 6.80E-34 | 5.90E-32 | 66.12667 |
| SPRR1B | mRNA | UP | 1.167606 | -1.50166 | 3.519979 | 0.00047 | 0.002912 | -1.03922 |
| CTD-2370N5.3 | mRNA | UP | 1.167498 | -3.75069 | 11.613 | 7.56E-28 | 5.05E-26 | 52.4483 |
| TSPAN19 | mRNA | UP | 1.165735 | -2.20777 | 4.391125 | 1.37E-05 | 0.000126 | 2.326553 |
| SIRPA | mRNA | UP | 1.165119 | 5.567868 | 16.40155 | 6.26E-49 | 1.15E-46 | 100.3723 |
| CASS4 | mRNA | UP | 1.165001 | 2.569425 | 13.98934 | 6.53E-38 | 6.88E-36 | 75.28752 |
| GPNMB | mRNA | UP | 1.164889 | 7.690998 | 11.79533 | 1.39E-28 | 9.55E-27 | 53.58147 |
| HLA-DMA | mRNA | UP | 1.164603 | 7.066543 | 14.16765 | 1.06E-38 | 1.16E-36 | 76.81222 |
| CTSG | mRNA | UP | 1.164221 | -0.75503 | 6.52589 | 1.62E-10 | 3.40E-09 | 13.19032 |
| KLHDC7B | mRNA | UP | 1.163485 | 1.935332 | 9.565525 | 4.64E-20 | 2.20E-18 | 34.60279 |
| CCL14 | mRNA | UP | 1.161388 | -0.48114 | 7.695885 | 7.24E-14 | 2.22E-12 | 20.71449 |
| CCL3L3 | mRNA | UP | 1.161109 | 0.500642 | 8.652817 | 6.47E-17 | 2.51E-15 | 27.5538 |
| AGAP2 | mRNA | UP | 1.158972 | 2.464989 | 17.61709 | 1.08E-54 | 2.77E-52 | 113.6737 |
| TDO2 | mRNA | UP | 1.158364 | 1.89478 | 9.808593 | 6.20E-21 | 3.08E-19 | 36.59197 |
| PDE6G | mRNA | UP | 1.158225 | 0.276891 | 12.89705 | 3.61E-33 | 3.00E-31 | 64.52651 |
| HEATR9 | mRNA | UP | 1.156744 | -2.99564 | 8.851688 | 1.40E-17 | 5.63E-16 | 29.19349 |
| KCNMA1 | mRNA | UP | 1.156059 | 3.306054 | 11.52224 | 1.75E-27 | 1.16E-25 | 51.42871 |
| NAIP | mRNA | UP | 1.155905 | -0.06884 | 13.55261 | 5.41E-36 | 5.18E-34 | 70.96899 |
| AMPD1 | mRNA | UP | 1.155379 | -0.77493 | 7.718652 | 6.17E-14 | 1.90E-12 | 20.88647 |
| DRGX | mRNA | UP | 1.155295 | -1.56611 | 4.444876 | 1.08E-05 | 0.000102 | 2.520141 |
| INPP5D | mRNA | UP | 1.155227 | 5.015868 | 16.17294 | 7.36E-48 | 1.23E-45 | 97.94946 |
| RASGRF1 | mRNA | UP | 1.154788 | 2.65793 | 6.435789 | 2.82E-10 | 5.72E-09 | 12.40154 |
| ITGA4 | mRNA | UP | 1.154647 | 3.863098 | 15.0392 | 1.26E-42 | 1.65E-40 | 86.0189 |
| ADAMTS8 | mRNA | UP | 1.15453 | 1.144494 | 6.981939 | 9.03E-12 | 2.22E-10 | 15.88289 |
| MUSK | mRNA | UP | 1.1523 | -2.4519 | 8.06736 | 5.10E-15 | 1.73E-13 | 23.39448 |
| ARHGAP25 | mRNA | UP | 1.152181 | 4.078609 | 22.36263 | 7.18E-78 | 1.50E-74 | 166.7659 |
| VNN1 | mRNA | UP | 1.151558 | 1.593513 | 7.600871 | 1.41E-13 | 4.20E-12 | 19.92959 |
| HAPLN3 | mRNA | UP | 1.151459 | 3.894629 | 14.2741 | 3.55E-39 | 3.97E-37 | 78.12149 |
| DMBT1 | mRNA | UP | 1.149586 | 5.096665 | 4.112474 | 4.56E-05 | 0.000369 | 0.584165 |
| BHLHE41 | mRNA | UP | 1.148803 | 3.986943 | 12.25712 | 1.77E-30 | 1.32E-28 | 58.22829 |
| GPA33 | mRNA | UP | 1.147805 | -0.36022 | 6.977397 | 9.31E-12 | 2.28E-10 | 15.95574 |
| S100Z | mRNA | UP | 1.146636 | -2.45982 | 10.9077 | 4.64E-25 | 2.79E-23 | 46.12131 |
| ROS1 | mRNA | UP | 1.146621 | 5.157195 | 6.117557 | 1.88E-09 | 3.43E-08 | 10.32026 |
| GLIPR1 | mRNA | UP | 1.145388 | 4.273643 | 17.32458 | 2.69E-53 | 6.41E-51 | 110.4418 |
| NLRP7 | mRNA | UP | 1.140777 | -2.01634 | 8.839123 | 1.54E-17 | 6.18E-16 | 29.07506 |
| RHOH | mRNA | UP | 1.140348 | 3.132509 | 13.5001 | 9.15E-36 | 8.71E-34 | 70.35676 |
| TNR | mRNA | UP | 1.139661 | -2.02888 | 7.25327 | 1.51E-12 | 4.06E-11 | 17.8163 |
| PLA2G7 | mRNA | UP | 1.13947 | 3.593257 | 12.64971 | 4.03E-32 | 3.19E-30 | 62.00354 |
| PIGR | mRNA | UP | 1.139101 | 7.709598 | 4.35856 | 1.58E-05 | 0.000143 | 1.302081 |
| HEMGN | mRNA | UP | 1.137984 | -4.75842 | 9.899367 | 2.90E-21 | 1.47E-19 | 37.51156 |
| DDO | mRNA | UP | 1.137842 | 0.952003 | 10.65976 | 4.18E-24 | 2.40E-22 | 43.85904 |
| NDP | mRNA | UP | 1.137643 | -2.2044 | 5.114625 | 4.45E-07 | 5.54E-06 | 5.602588 |
| ADORA3 | mRNA | UP | 1.137173 | 2.6768 | 12.19159 | 3.30E-30 | 2.41E-28 | 57.68542 |
| TNFRSF13C | mRNA | UP | 1.135971 | 1.707202 | 8.830997 | 1.64E-17 | 6.58E-16 | 28.83013 |
| FFAR4 | mRNA | UP | 1.135556 | 0.661187 | 8.443837 | 3.16E-16 | 1.17E-14 | 25.98512 |
| TPSB2 | mRNA | UP | 1.134568 | 3.244319 | 6.936241 | 1.22E-11 | 2.94E-10 | 15.4265 |
| HHIP | mRNA | UP | 1.134421 | 1.866224 | 4.749741 | 2.65E-06 | 2.84E-05 | 3.604144 |
| SLC31A2 | mRNA | UP | 1.134297 | 0.920412 | 15.86204 | 2.07E-46 | 3.23E-44 | 94.73755 |
| MEFV | mRNA | UP | 1.134183 | 0.028164 | 12.27636 | 1.47E-30 | 1.10E-28 | 58.58651 |
| SHD | mRNA | UP | 1.134067 | -3.47354 | 7.782603 | 3.93E-14 | 1.23E-12 | 21.41859 |
| CPED1 | mRNA | UP | 1.134051 | 2.90059 | 12.45065 | 2.76E-31 | 2.13E-29 | 60.13292 |
| CACNG6 | mRNA | UP | 1.133251 | -1.90647 | 4.632236 | 4.59E-06 | 4.69E-05 | 3.352486 |
| DAPP1 | mRNA | UP | 1.132477 | 3.849071 | 13.29297 | 7.24E-35 | 6.59E-33 | 68.26573 |
| CH25H | mRNA | UP | 1.132463 | 2.673023 | 7.822993 | 2.95E-14 | 9.37E-13 | 21.38075 |
| CRLF2 | mRNA | UP | 1.13243 | -2.20998 | 6.830925 | 2.39E-11 | 5.59E-10 | 15.12575 |
| MAFB | mRNA | UP | 1.13127 | 5.212747 | 15.8393 | 2.63E-46 | 4.07E-44 | 94.37776 |
| WIPF1 | mRNA | UP | 1.130409 | 5.947554 | 20.99376 | 3.99E-71 | 3.49E-68 | 151.274 |
| MMP3 | mRNA | UP | 1.129916 | -0.68078 | 5.475354 | 6.84E-08 | 9.80E-07 | 7.321065 |
| SPOCK2 | mRNA | UP | 1.129732 | 5.72505 | 12.32652 | 9.09E-31 | 6.87E-29 | 58.75889 |
| CLEC5A | mRNA | UP | 1.127484 | 2.86035 | 9.31844 | 3.46E-19 | 1.55E-17 | 32.55563 |
| MEI1 | mRNA | UP | 1.127271 | 2.433365 | 11.31505 | 1.17E-26 | 7.56E-25 | 49.59668 |
| DPT | mRNA | UP | 1.126564 | 3.861688 | 8.808994 | 1.94E-17 | 7.76E-16 | 28.50289 |
| P2RY14 | mRNA | UP | 1.126023 | 1.513262 | 14.1791 | 9.41E-39 | 1.03E-36 | 77.24199 |
| C6orf222 | mRNA | UP | 1.125143 | -1.16903 | 5.050785 | 6.12E-07 | 7.45E-06 | 5.240065 |
| UTS2 | mRNA | UP | 1.124052 | -2.45344 | 7.406795 | 5.34E-13 | 1.50E-11 | 18.84444 |
| CILP | mRNA | UP | 1.123666 | 3.051632 | 6.300552 | 6.38E-10 | 1.24E-08 | 11.5709 |
| APOL4 | mRNA | UP | 1.123659 | 3.576576 | 12.57779 | 8.09E-32 | 6.32E-30 | 61.31271 |
| HCLS1 | mRNA | UP | 1.123558 | 5.799737 | 16.27273 | 2.51E-48 | 4.35E-46 | 98.97362 |
| LINGO3 | mRNA | UP | 1.12352 | -1.9344 | 9.74387 | 1.06E-20 | 5.22E-19 | 36.22682 |
| DNAH8 | mRNA | UP | 1.120482 | -2.01044 | 9.183536 | 1.02E-18 | 4.44E-17 | 31.73955 |
| CCRL2 | mRNA | UP | 1.119595 | 2.559988 | 13.17943 | 2.23E-34 | 1.97E-32 | 67.21191 |
| LILRA5 | mRNA | UP | 1.119435 | 1.40491 | 11.19456 | 3.51E-26 | 2.23E-24 | 48.56487 |
| CUX2 | mRNA | UP | 1.119168 | -2.75075 | 9.557584 | 4.95E-20 | 2.33E-18 | 34.73007 |
| FCER1A | mRNA | UP | 1.119158 | 1.122552 | 5.676656 | 2.30E-08 | 3.52E-07 | 8.244405 |
| LRRN3 | mRNA | UP | 1.11816 | 0.143336 | 9.18036 | 1.05E-18 | 4.55E-17 | 31.63243 |
| PLXNC1 | mRNA | UP | 1.117773 | 4.923461 | 16.95328 | 1.56E-51 | 3.28E-49 | 106.3716 |
| MMP12 | mRNA | UP | 1.117753 | 3.631511 | 5.45192 | 7.75E-08 | 1.10E-06 | 6.852275 |
| OSM | mRNA | UP | 1.116606 | 1.947032 | 9.037252 | 3.26E-18 | 1.37E-16 | 30.4055 |
| APOBEC3A | mRNA | UP | 1.116217 | 0.006923 | 8.454741 | 2.91E-16 | 1.08E-14 | 26.10371 |
| ANKRD33B | mRNA | UP | 1.115899 | 1.442382 | 10.58545 | 8.03E-24 | 4.54E-22 | 43.189 |
| ITM2A | mRNA | UP | 1.115019 | 3.810568 | 13.30671 | 6.31E-35 | 5.78E-33 | 68.40368 |
| P2RX1 | mRNA | UP | 1.113745 | 1.480525 | 11.78628 | 1.51E-28 | 1.04E-26 | 53.95482 |
| CR1L | mRNA | UP | 1.113421 | -2.242 | 9.288033 | 4.42E-19 | 1.96E-17 | 32.56805 |
| RAB33A | mRNA | UP | 1.111303 | -0.1167 | 15.54302 | 6.18E-45 | 9.02E-43 | 91.35614 |
| MFAP4 | mRNA | UP | 1.110817 | 5.769737 | 8.806831 | 1.98E-17 | 7.88E-16 | 28.31715 |
| CCDC170 | mRNA | UP | 1.109852 | 2.074536 | 8.578723 | 1.14E-16 | 4.36E-15 | 26.8951 |
| AQP9 | mRNA | UP | 1.109849 | 2.975528 | 8.672392 | 5.57E-17 | 2.16E-15 | 27.53391 |
| CXCL12 | mRNA | UP | 1.108893 | 4.749869 | 11.06216 | 1.16E-25 | 7.20E-24 | 47.16816 |
| TREML2 | mRNA | UP | 1.107951 | -0.73283 | 9.995184 | 1.29E-21 | 6.67E-20 | 38.2673 |
| ITIH1 | mRNA | UP | 1.106822 | -3.3493 | 8.154576 | 2.70E-15 | 9.40E-14 | 24.03772 |
| GIMAP8 | mRNA | UP | 1.106167 | 3.696533 | 15.74608 | 7.12E-46 | 1.07E-43 | 93.46042 |
| APOE | mRNA | UP | 1.105286 | 7.800108 | 10.44723 | 2.69E-23 | 1.48E-21 | 41.48315 |
| TNFSF14 | mRNA | UP | 1.105103 | 1.519999 | 10.38874 | 4.46E-23 | 2.44E-21 | 41.49 |
| RASGRP4 | mRNA | UP | 1.103698 | 1.239594 | 13.77026 | 6.03E-37 | 6.08E-35 | 73.12213 |
| IFI44L | mRNA | UP | 1.103667 | 4.646069 | 7.95407 | 1.16E-14 | 3.80E-13 | 22.13689 |
| ATP8B4 | mRNA | UP | 1.102554 | 1.913217 | 15.42123 | 2.25E-44 | 3.18E-42 | 90.07865 |
| NLRC4 | mRNA | UP | 1.09998 | 1.794485 | 16.71816 | 2.02E-50 | 4.06E-48 | 103.9002 |
| GPM6A | mRNA | UP | 1.098959 | -0.14926 | 5.919571 | 5.91E-09 | 1.00E-07 | 9.657331 |
| CDA | mRNA | UP | 1.098449 | 2.567805 | 5.725433 | 1.76E-08 | 2.75E-07 | 8.385742 |
| PKHD1L1 | mRNA | UP | 1.097913 | -0.54257 | 7.727034 | 5.82E-14 | 1.80E-12 | 20.9322 |
| SYNDIG1L | mRNA | UP | 1.097436 | -1.48502 | 6.595253 | 1.06E-10 | 2.27E-09 | 13.64722 |
| FABP4 | mRNA | UP | 1.096682 | 0.811198 | 6.107011 | 2.00E-09 | 3.64E-08 | 10.63747 |
| ANKRD55 | mRNA | UP | 1.096628 | -1.74804 | 9.904963 | 2.77E-21 | 1.41E-19 | 37.54726 |
| CSTA | mRNA | UP | 1.096098 | 2.421041 | 10.12366 | 4.35E-22 | 2.29E-20 | 39.18467 |
| GFRA2 | mRNA | UP | 1.095635 | -0.24537 | 9.783668 | 7.64E-21 | 3.78E-19 | 36.49947 |
| EPHB6 | mRNA | UP | 1.0954 | 1.969006 | 9.344446 | 2.81E-19 | 1.26E-17 | 32.82373 |
| GLIPR2 | mRNA | UP | 1.094985 | 4.816843 | 15.05522 | 1.06E-42 | 1.40E-40 | 86.13679 |
| CCR9 | mRNA | UP | 1.093611 | -3.34539 | 7.279341 | 1.26E-12 | 3.44E-11 | 18.02576 |
| KCNE1 | mRNA | UP | 1.092389 | -0.96708 | 8.733055 | 3.49E-17 | 1.37E-15 | 28.23282 |
| CYSLTR1 | mRNA | UP | 1.092122 | 2.391707 | 10.67767 | 3.57E-24 | 2.06E-22 | 43.937 |
| MYBPH | mRNA | UP | 1.089873 | -0.62403 | 4.720942 | 3.03E-06 | 3.22E-05 | 3.671375 |
| SAMHD1 | mRNA | UP | 1.08984 | 6.763001 | 18.93021 | 4.99E-61 | 1.89E-58 | 128.0554 |
| SCGB3A1 | mRNA | UP | 1.088121 | 4.873083 | 3.629154 | 0.000313 | 0.002041 | -1.21056 |
| LILRB3 | mRNA | UP | 1.086618 | 1.902023 | 15.31439 | 6.96E-44 | 9.51E-42 | 88.95746 |
| GNG2 | mRNA | UP | 1.083211 | 3.817575 | 19.46193 | 1.29E-63 | 6.16E-61 | 134.0989 |
| CD300A | mRNA | UP | 1.083103 | 3.695362 | 14.37141 | 1.30E-39 | 1.50E-37 | 79.1259 |
| GPR132 | mRNA | UP | 1.081848 | 3.134831 | 12.99244 | 1.42E-33 | 1.20E-31 | 65.35264 |
| C20orf85 | mRNA | UP | 1.080616 | -0.25449 | 3.451182 | 0.000604 | 0.003628 | -1.35895 |
| CDHR1 | mRNA | UP | 1.079325 | -1.41983 | 8.202623 | 1.90E-15 | 6.65E-14 | 24.32891 |
| CD1D | mRNA | UP | 1.079069 | 1.482686 | 14.17546 | 9.76E-39 | 1.07E-36 | 77.20565 |
| FAS | mRNA | UP | 1.079064 | 4.30953 | 12.75411 | 1.46E-32 | 1.18E-30 | 62.96709 |
| CLEC2B | mRNA | UP | 1.078503 | 3.579416 | 12.61395 | 5.70E-32 | 4.49E-30 | 61.65996 |
| KMO | mRNA | UP | 1.078108 | 1.839787 | 13.47687 | 1.16E-35 | 1.09E-33 | 70.17812 |
| CXCL6 | mRNA | UP | 1.077804 | 0.25264 | 4.6518 | 4.19E-06 | 4.31E-05 | 3.297365 |
| GAS7 | mRNA | UP | 1.077426 | 4.74032 | 13.48676 | 1.05E-35 | 9.92E-34 | 70.13243 |
| RASSF2 | mRNA | UP | 1.075516 | 4.864918 | 15.51024 | 8.75E-45 | 1.26E-42 | 90.91065 |
| SNX10 | mRNA | UP | 1.075433 | 4.63961 | 13.36561 | 3.51E-35 | 3.23E-33 | 68.93577 |
| VAV1 | mRNA | UP | 1.075341 | 4.349574 | 14.83797 | 1.03E-41 | 1.30E-39 | 83.90153 |
| MS4A14 | mRNA | UP | 1.074617 | 0.737538 | 10.78955 | 1.33E-24 | 7.85E-23 | 45.00244 |
| GYPE | mRNA | UP | 1.073584 | -1.86338 | 9.666985 | 2.01E-20 | 9.71E-19 | 35.59824 |
| CXCR4 | mRNA | UP | 1.073499 | 6.161968 | 15.00915 | 1.73E-42 | 2.23E-40 | 85.56674 |
| SCN2B | mRNA | UP | 1.073032 | -0.4569 | 7.457878 | 3.77E-13 | 1.08E-11 | 19.09754 |
| CTSS | mRNA | UP | 1.072884 | 7.824176 | 16.30174 | 1.84E-48 | 3.20E-46 | 99.13008 |
| CD101 | mRNA | UP | 1.072578 | 1.638072 | 14.03577 | 4.07E-38 | 4.31E-36 | 75.78612 |
| ZBTB32 | mRNA | UP | 1.07222 | -0.20331 | 12.33985 | 8.00E-31 | 6.07E-29 | 59.19187 |
| KIR2DL3 | mRNA | UP | 1.071846 | -4.09986 | 6.706759 | 5.26E-11 | 1.18E-09 | 14.39733 |
| HLA-G | mRNA | UP | 1.071796 | 2.528793 | 8.46385 | 2.71E-16 | 1.01E-14 | 26.00602 |
| PATL2 | mRNA | UP | 1.071677 | 0.878129 | 11.34883 | 8.61E-27 | 5.57E-25 | 49.97806 |
| IL37 | mRNA | UP | 1.071541 | -0.45547 | 3.513426 | 0.000481 | 0.002975 | -1.1327 |
| HS3ST3B1 | mRNA | UP | 1.070983 | 2.06738 | 13.48214 | 1.10E-35 | 1.04E-33 | 70.22265 |
| SGCG | mRNA | UP | 1.070683 | -2.19563 | 7.695478 | 7.26E-14 | 2.22E-12 | 20.78693 |
| EPYC | mRNA | UP | 1.070676 | -2.09891 | 4.456258 | 1.02E-05 | 9.73E-05 | 2.597454 |
| DPYS | mRNA | UP | 1.069959 | -2.15965 | 7.260473 | 1.44E-12 | 3.87E-11 | 17.86845 |
| ARRDC5 | mRNA | UP | 1.069057 | -1.58876 | 12.52972 | 1.29E-31 | 9.99E-30 | 61.00909 |
| ATP1A4 | mRNA | UP | 1.068397 | -3.06002 | 8.968294 | 5.61E-18 | 2.33E-16 | 30.08875 |
| GPR87 | mRNA | UP | 1.067967 | 1.049491 | 3.712633 | 0.000228 | 0.00154 | -0.55539 |
| TPSD1 | mRNA | UP | 1.067943 | -0.29718 | 4.719165 | 3.06E-06 | 3.24E-05 | 3.640396 |
| SLC8A1 | mRNA | UP | 1.067028 | 3.552422 | 14.65862 | 6.69E-41 | 7.99E-39 | 82.0845 |
| LIPM | mRNA | UP | 1.066642 | -0.31589 | 7.295865 | 1.13E-12 | 3.09E-11 | 18.01295 |
| KLRC3 | mRNA | UP | 1.066016 | -4.32142 | 7.313354 | 1.01E-12 | 2.76E-11 | 18.24804 |
| PM20D1 | mRNA | UP | 1.065969 | -2.22634 | 9.223514 | 7.42E-19 | 3.25E-17 | 32.05896 |
| GYPC | mRNA | UP | 1.065727 | 3.988339 | 16.98008 | 1.17E-51 | 2.47E-49 | 106.6995 |
| DPF3 | mRNA | UP | 1.065635 | -0.62082 | 11.91012 | 4.73E-29 | 3.33E-27 | 55.16771 |
| TM6SF1 | mRNA | UP | 1.065364 | 1.603962 | 13.19061 | 2.00E-34 | 1.78E-32 | 67.35844 |
| CR2 | mRNA | UP | 1.063832 | 2.240843 | 5.690033 | 2.14E-08 | 3.30E-07 | 8.223648 |
| IKZF3 | mRNA | UP | 1.062313 | 4.55821 | 12.70255 | 2.41E-32 | 1.93E-30 | 62.45177 |
| IL7 | mRNA | UP | 1.062009 | 1.780008 | 12.13607 | 5.59E-30 | 4.04E-28 | 57.20523 |
| SH2D1B | mRNA | UP | 1.060958 | -1.06783 | 9.508669 | 7.39E-20 | 3.44E-18 | 34.29468 |
| NRROS | mRNA | UP | 1.059793 | 2.87004 | 15.36227 | 4.19E-44 | 5.87E-42 | 89.43749 |
| DUSP2 | mRNA | UP | 1.058575 | 2.917733 | 12.8548 | 5.46E-33 | 4.51E-31 | 64.02355 |
| SPATC1 | mRNA | UP | 1.058121 | -1.79894 | 12.30797 | 1.09E-30 | 8.19E-29 | 58.90452 |
| ERAP2 | mRNA | UP | 1.057789 | 4.97911 | 7.133211 | 3.35E-12 | 8.67E-11 | 16.53365 |
| EPSTI1 | mRNA | UP | 1.056925 | 4.465052 | 12.7728 | 1.22E-32 | 9.91E-31 | 63.13761 |
| SLC2A5 | mRNA | UP | 1.05685 | 2.649261 | 8.676211 | 5.41E-17 | 2.11E-15 | 27.58694 |
| FLI1 | mRNA | UP | 1.056598 | 4.173689 | 17.76701 | 2.06E-55 | 5.58E-53 | 115.2917 |
| SOWAHD | mRNA | UP | 1.055901 | 0.311428 | 13.05708 | 7.49E-34 | 6.49E-32 | 66.0833 |
| ZEB2 | mRNA | UP | 1.054696 | 5.180289 | 16.22801 | 4.07E-48 | 6.94E-46 | 98.53041 |
| OMG | mRNA | UP | 1.05378 | 0.506645 | 7.006218 | 7.71E-12 | 1.91E-10 | 16.08286 |
| C19orf38 | mRNA | UP | 1.053771 | 1.164295 | 13.88801 | 1.83E-37 | 1.88E-35 | 74.30632 |
| MMP25 | mRNA | UP | 1.053097 | 1.545616 | 13.0241 | 1.04E-33 | 8.87E-32 | 65.72954 |
| ITPRIPL1 | mRNA | UP | 1.052524 | 1.176588 | 13.74099 | 8.11E-37 | 8.09E-35 | 72.83029 |
| CRHBP | mRNA | UP | 1.052355 | -2.4997 | 9.513586 | 7.10E-20 | 3.30E-18 | 34.37157 |
| RGS8 | mRNA | UP | 1.051562 | -3.86471 | 8.158656 | 2.62E-15 | 9.12E-14 | 24.07156 |
| POU2F2 | mRNA | UP | 1.051496 | 3.332967 | 12.23195 | 2.24E-30 | 1.67E-28 | 58.03014 |
| IL18R1 | mRNA | UP | 1.050215 | 1.931222 | 12.32772 | 8.99E-31 | 6.80E-29 | 59.0094 |
| CST6 | mRNA | UP | 1.04986 | 2.44356 | 5.718274 | 1.83E-08 | 2.85E-07 | 8.357671 |
| UNC45B | mRNA | UP | 1.048889 | -2.32073 | 9.322622 | 3.35E-19 | 1.50E-17 | 32.84365 |
| ADTRP | mRNA | UP | 1.048269 | 1.455094 | 8.665992 | 5.85E-17 | 2.27E-15 | 27.59318 |
| WDFY4 | mRNA | UP | 1.047712 | 4.230591 | 10.18293 | 2.62E-22 | 1.40E-20 | 39.55708 |
| FAM65B | mRNA | UP | 1.046683 | 3.680043 | 10.72558 | 2.34E-24 | 1.37E-22 | 44.27149 |
| ANKRD44 | mRNA | UP | 1.04661 | 4.22219 | 15.86057 | 2.10E-46 | 3.28E-44 | 94.65443 |
| CDX1 | mRNA | UP | 1.046394 | -1.81525 | 9.972312 | 1.57E-21 | 8.04E-20 | 38.10703 |
| S100A8 | mRNA | UP | 1.045391 | 3.651521 | 6.427595 | 2.96E-10 | 6.01E-09 | 12.26698 |
| RRAD | mRNA | UP | 1.045376 | 3.83076 | 6.82046 | 2.56E-11 | 5.95E-10 | 14.64689 |
| FCGR2A | mRNA | UP | 1.044676 | 5.730486 | 13.20755 | 1.69E-34 | 1.51E-32 | 67.29467 |
| COLEC12 | mRNA | UP | 1.044267 | 4.839738 | 8.955426 | 6.21E-18 | 2.57E-16 | 29.54669 |
| IL15 | mRNA | UP | 1.044146 | 2.421961 | 14.1249 | 1.64E-38 | 1.77E-36 | 76.66572 |
| SPRR2F | mRNA | UP | 1.044086 | -4.49286 | 4.577317 | 5.91E-06 | 5.90E-05 | 3.158034 |
| ASGR2 | mRNA | UP | 1.042632 | -0.54972 | 9.544053 | 5.53E-20 | 2.59E-18 | 34.56078 |
| NETO1 | mRNA | UP | 1.041183 | -1.15408 | 4.811639 | 1.97E-06 | 2.17E-05 | 4.118045 |
| IFFO1 | mRNA | UP | 1.040822 | 3.260941 | 15.63029 | 2.45E-45 | 3.61E-43 | 92.24971 |
| IL13RA2 | mRNA | UP | 1.040173 | -0.7651 | 5.695404 | 2.07E-08 | 3.21E-07 | 8.480511 |
| GCSAML | mRNA | UP | 1.039498 | -1.23857 | 6.929083 | 1.27E-11 | 3.07E-10 | 15.69826 |
| GRIP2 | mRNA | UP | 1.039375 | -0.66298 | 9.205605 | 8.56E-19 | 3.74E-17 | 31.86802 |
| GRIA1 | mRNA | UP | 1.039179 | -0.95778 | 4.991467 | 8.22E-07 | 9.77E-06 | 4.944381 |
| ZC3H12D | mRNA | UP | 1.038621 | 1.795032 | 12.74069 | 1.66E-32 | 1.35E-30 | 62.96866 |
| SLC9A9 | mRNA | UP | 1.038397 | 2.781706 | 14.97665 | 2.43E-42 | 3.11E-40 | 85.40904 |
| TMEM255A | mRNA | UP | 1.038049 | 1.066052 | 8.387185 | 4.83E-16 | 1.77E-14 | 25.54147 |
| KLRG1 | mRNA | UP | 1.036804 | 0.401066 | 13.81005 | 4.03E-37 | 4.10E-35 | 73.53488 |
| DOK3 | mRNA | UP | 1.036522 | 3.724312 | 15.83469 | 2.77E-46 | 4.25E-44 | 94.39978 |
| PLA2G4C | mRNA | UP | 1.035556 | 2.478191 | 13.57808 | 4.19E-36 | 4.03E-34 | 71.16187 |
| FGD3 | mRNA | UP | 1.034579 | 3.697151 | 15.8967 | 1.43E-46 | 2.26E-44 | 95.05994 |
| BTN1A1 | mRNA | UP | 1.034566 | -3.03251 | 8.831685 | 1.63E-17 | 6.54E-16 | 29.04183 |
| PIH1D3 | mRNA | UP | 1.033724 | -2.81874 | 4.576741 | 5.93E-06 | 5.91E-05 | 3.150411 |
| SYNE3 | mRNA | UP | 1.032349 | 2.870012 | 12.99439 | 1.39E-33 | 1.18E-31 | 65.38484 |
| EGR2 | mRNA | UP | 1.03203 | 3.421667 | 11.36191 | 7.64E-27 | 4.95E-25 | 49.96094 |
| GBP2 | mRNA | UP | 1.031324 | 6.36505 | 14.09571 | 2.21E-38 | 2.37E-36 | 76.13936 |
| GABRP | mRNA | UP | 1.030945 | 0.368017 | 4.258841 | 2.44E-05 | 0.000211 | 1.607582 |
| AKAP14 | mRNA | UP | 1.030028 | -2.58206 | 4.692673 | 3.46E-06 | 3.62E-05 | 3.653693 |
| DSE | mRNA | UP | 1.02926 | 4.729995 | 12.43985 | 3.06E-31 | 2.35E-29 | 59.91577 |
| C10orf67 | mRNA | UP | 1.027859 | -2.79132 | 5.887598 | 7.08E-09 | 1.18E-07 | 9.619378 |
| ST8SIA1 | mRNA | UP | 1.027697 | 1.697243 | 10.95912 | 2.93E-25 | 1.78E-23 | 46.45043 |
| TEKT1 | mRNA | UP | 1.027151 | -0.4108 | 3.925973 | 9.82E-05 | 0.000731 | 0.352906 |
| APOBEC3D | mRNA | UP | 1.026434 | 2.380221 | 13.74507 | 7.78E-37 | 7.79E-35 | 72.83517 |
| PTGS1 | mRNA | UP | 1.026347 | 4.138375 | 12.49206 | 1.85E-31 | 1.43E-29 | 60.45563 |
| GNG8 | mRNA | UP | 1.026309 | -3.47742 | 9.409888 | 1.65E-19 | 7.57E-18 | 33.55526 |
| TRIM22 | mRNA | UP | 1.026054 | 6.041834 | 13.95973 | 8.83E-38 | 9.27E-36 | 74.78596 |
| ADAM19 | mRNA | UP | 1.025176 | 4.57842 | 12.2122 | 2.71E-30 | 2.00E-28 | 57.76232 |
| CECR1 | mRNA | UP | 1.024772 | 6.232717 | 13.1892 | 2.03E-34 | 1.80E-32 | 67.07284 |
| NTRK1 | mRNA | UP | 1.024565 | -0.83258 | 10.64161 | 4.91E-24 | 2.80E-22 | 43.76828 |
| NTRK2 | mRNA | UP | 1.024466 | 1.181235 | 6.357295 | 4.54E-10 | 9.00E-09 | 12.05546 |
| LPAR5 | mRNA | UP | 1.024309 | 2.296097 | 11.81714 | 1.13E-28 | 7.83E-27 | 54.20163 |
| OVCH1 | mRNA | UP | 1.024181 | -2.96638 | 6.189835 | 1.23E-09 | 2.30E-08 | 11.31925 |
| IL1A | mRNA | UP | 1.023742 | -0.68811 | 6.096276 | 2.13E-09 | 3.87E-08 | 10.68048 |
| B2M | mRNA | UP | 1.023121 | 11.36402 | 17.22829 | 7.73E-53 | 1.81E-50 | 108.7912 |
| DBH | mRNA | UP | 1.022782 | -1.02084 | 9.388474 | 1.97E-19 | 8.96E-18 | 33.33025 |
| TMEM132C | mRNA | UP | 1.022321 | -2.86016 | 4.9538 | 9.89E-07 | 1.16E-05 | 4.862968 |
| RP1 | mRNA | UP | 1.022211 | -1.37611 | 5.189869 | 3.04E-07 | 3.89E-06 | 5.926837 |
| TTC16 | mRNA | UP | 1.022003 | -0.4246 | 7.970673 | 1.03E-14 | 3.39E-13 | 22.62653 |
| STAT4 | mRNA | UP | 1.021674 | 2.716575 | 14.73995 | 2.87E-41 | 3.50E-39 | 82.95672 |
| LGALS12 | mRNA | UP | 1.021566 | -1.81931 | 6.872529 | 1.83E-11 | 4.33E-10 | 15.36976 |
| RAB39A | mRNA | UP | 1.019243 | -0.94348 | 9.049722 | 2.96E-18 | 1.24E-16 | 30.66054 |
| LCNL1 | mRNA | UP | 1.018724 | -1.94709 | 7.184883 | 2.38E-12 | 6.26E-11 | 17.36743 |
| RLTPR | mRNA | UP | 1.018125 | 2.419963 | 9.719384 | 1.30E-20 | 6.36E-19 | 35.82467 |
| CPNE9 | mRNA | UP | 1.018086 | -2.45531 | 9.135983 | 1.49E-18 | 6.38E-17 | 31.37809 |
| VSIG1 | mRNA | UP | 1.017944 | 2.631221 | 3.915189 | 0.000103 | 0.000759 | 0.05401 |
| ANGPTL7 | mRNA | UP | 1.017791 | -3.61537 | 6.827975 | 2.44E-11 | 5.69E-10 | 15.14627 |
| C5orf58 | mRNA | UP | 1.016572 | -1.638 | 9.346415 | 2.76E-19 | 1.25E-17 | 33.01521 |
| KRT1 | mRNA | UP | 1.015819 | -2.91336 | 5.416588 | 9.35E-08 | 1.31E-06 | 7.131951 |
| NPL | mRNA | UP | 1.015278 | 4.482711 | 13.63207 | 2.43E-36 | 2.37E-34 | 71.59878 |
| S1PR4 | mRNA | UP | 1.014104 | 2.275308 | 12.56589 | 9.08E-32 | 7.07E-30 | 61.26623 |
| CDK15 | mRNA | UP | 1.012346 | -1.03614 | 8.911769 | 8.73E-18 | 3.58E-16 | 29.5984 |
| LRRK2 | mRNA | UP | 1.012099 | 5.365599 | 5.887747 | 7.07E-09 | 1.18E-07 | 9.009722 |
| CLIC5 | mRNA | UP | 1.011957 | 4.066442 | 6.737451 | 4.33E-11 | 9.80E-10 | 14.10944 |
| SLC6A12 | mRNA | UP | 1.011842 | 1.133035 | 9.779165 | 7.93E-21 | 3.92E-19 | 36.39438 |
| PRKCQ | mRNA | UP | 1.011553 | 1.813961 | 10.23321 | 1.71E-22 | 9.14E-21 | 40.14762 |
| IL1RL1 | mRNA | UP | 1.009985 | 1.055995 | 6.02868 | 3.16E-09 | 5.58E-08 | 10.17598 |
| CD300E | mRNA | UP | 1.009668 | 1.395591 | 8.697086 | 4.61E-17 | 1.80E-15 | 27.83181 |
| COTL1 | mRNA | UP | 1.009094 | 7.088973 | 14.855 | 8.66E-42 | 1.09E-39 | 83.88625 |
| NPY4R | mRNA | UP | 1.008142 | -3.69788 | 5.814605 | 1.07E-08 | 1.73E-07 | 9.243601 |
| TCF21 | mRNA | UP | 1.007843 | 1.815383 | 7.553328 | 1.95E-13 | 5.76E-12 | 19.58945 |
| SERPINB9 | mRNA | UP | 1.006741 | 4.569258 | 14.59525 | 1.29E-40 | 1.53E-38 | 81.37902 |
| OGN | mRNA | UP | 1.006654 | 1.210693 | 5.809345 | 1.10E-08 | 1.78E-07 | 8.951368 |
| CLEC2D | mRNA | UP | 1.006613 | 3.544513 | 14.87034 | 7.38E-42 | 9.32E-40 | 84.27594 |
| IL18BP | mRNA | UP | 1.006595 | 4.366745 | 16.18288 | 6.61E-48 | 1.11E-45 | 98.08714 |
| SLC24A4 | mRNA | UP | 1.005806 | -0.40263 | 9.738897 | 1.11E-20 | 5.44E-19 | 36.13875 |
| SHISA3 | mRNA | UP | 1.004479 | 1.247205 | 4.047415 | 5.98E-05 | 0.000471 | 0.686396 |
| EMP3 | mRNA | UP | 1.003319 | 5.027997 | 14.29583 | 2.84E-39 | 3.19E-37 | 78.27733 |
| C11orf88 | mRNA | UP | 1.003249 | -1.10027 | 4.608924 | 5.11E-06 | 5.17E-05 | 3.203306 |
| NIPAL4 | mRNA | UP | 1.00244 | -1.30611 | 6.654898 | 7.28E-11 | 1.60E-09 | 14.00078 |
| PREX1 | mRNA | UP | 1.002391 | 5.446722 | 16.12043 | 1.29E-47 | 2.16E-45 | 97.36272 |
| GATA3 | mRNA | UP | 1.002387 | 1.913967 | 10.53447 | 1.26E-23 | 7.03E-22 | 42.72145 |
| HVCN1 | mRNA | UP | 1.000925 | 2.992409 | 18.41637 | 1.54E-58 | 4.92E-56 | 122.4717 |
