# Supplementary material for: Screening of T Cell-Related Long Noncoding RNA-MicroRNA-mRNA Regulatory Networks in Non-Small-Cell Lung Cancer
Source: Biomed Res Int. 2020 Nov 14;2020:5816763. doi: 10.1155/2020/5816763 (PMC7684158; doi:10.1155/2020/5816763)
Supplement: Supplementary 3 — Supplementary Table 3: genes related with CD8 T cells. [file 5816763.f3.docx]

Supplementary Table 3 Genes related with CD8 T cells.

| Gene | Gene | Gene |
| --- | --- | --- |
| CRTAM | ITK | TRBV6-6 |
| TRBC2 | IL12RB1 | ANTXRLP1 |
| CD2 | TRBV14 | PTGDR |
| EOMES | TRBJ2-1 | IL21-AS1 |
| PYHIN1 | GPR174 | TRAV14DV4 |
| SH2D1A | TRAV4 | CD27 |
| FTH1P22 | TRAV3 | SLAMF1 |
| TRAC | FAM26F | TRAV25 |
| GZMK | TRBV18 | CXCR2P1 |
| THEMIS | TRBV5-4 | TRBV5-1 |
| TIGIT | ABCD2 | TRBV7-3 |
| CD3G | TRAV13-2 | TRAV8-6 |
| AC104820.2 | TRAV17 | TRAV35 |
| TRBV7-9 | TRBV7-6 | GIMAP4 |
| GBP5 | TRBV19 | TRBV15 |
| TRAV21 | TRAV8-3 | ICOS |
| SAMD3 | TRBV29-1 | PCED1B-AS1 |
| TRAT1 | ITGAL | TRBV6-1 |
| TRAV29DV5 | TRBV6-5 | TRBV12-4 |
| CXCL9 | TRBV21-1 | TRAV5 |
| TRBV25-1 | TRAV13-1 | TRAV8-1 |
| CCR5 | TRAV8-2 | TRAV16 |
| ZNF80 | CXCL10 | TRBV13 |
| TRAV12-2 | TRGV3 | CTLA4 |
| SLAMF6 | TRBV2 | RP11-10J5.1 |
| SIT1 | TRBV11-2 | SIRPG-AS1 |
| TRBV27 | TRBV5-6 | TRBV10-3 |
| TRBV3-1 | ZNF831 | GVINP1 |
| TRAV12-3 | TRAF3IP3 | CD226 |
| TOMM20P2 | AOAH | PLA2G2D |
