# Supplementary material for: Screening of T Cell-Related Long Noncoding RNA-MicroRNA-mRNA Regulatory Networks in Non-Small-Cell Lung Cancer
Source: Biomed Res Int. 2020 Nov 14;2020:5816763. doi: 10.1155/2020/5816763 (PMC7684158; doi:10.1155/2020/5816763)
Supplement: Supplementary 4 — Supplementary Table 4: genes related with memory-activated CD4 T cells. [file 5816763.f4.docx]

Supplementary Table 4 Genes related with memory activated CD4 T cells.

| Gene | Gene | Gene |
| --- | --- | --- |
| GBP5 | CD226 | TRBC2 |
| RP11-10J5.1 | CTLA4 | ITK |
| CXCL9 | CD3G | EOMES |
| CXCL10 | PDCD1LG2 | TNFRSF9 |
| FAM26F | CLEC4D | CCL8 |
| TIGIT | ADAMDEC1 | TRAC |
| TOMM20P2 | TRAV21 | RP11-81H14.2 |
| AC104820.2 | SIGLEC10 | CXCR2P1 |
| AIM2 | IL12RB1 | GPR174 |
| CRTAM | CXCL11 | CCR5 |
| THEMIS | FTH1P22 | LILRB2 |
| IL2RA | SLAMF7 | AC011893.3 |
| CD2 | TRDV1 | TRAT1 |
| IL21-AS1 | ABCD2 | TNFSF13B |
| SH2D1A | TRBV5-6 | TRBV7-9 |
| ICOS | PYHIN1 | AOAH |
